# Mixed effects approach to the analysis of the stepped wedge cluster randomised trial—Investigating the confounding effect of time through simulation

**DOI:** 10.1371/journal.pone.0208876

**Published:** 2018-12-13

**Authors:** Alecia Nickless, Merryn Voysey, John Geddes, Ly-Mee Yu, Thomas R. Fanshawe

**Affiliations:** 1 Nuffield Department of Primary Care Health Sciences, University of Oxford, Oxford, United Kingdom; 2 Oxford Vaccine Group, Department of Paediatrics, University of Oxford, Oxford, United Kingdom; 3 Department of Psychiatry, University of Oxford, Oxford, United Kingdom; Weill Cornell Medical College in Qatar, QATAR

## Abstract

**Background:**

A stepped wedge cluster randomised trial (SWCRT) is a multicentred study which allows an intervention to be rolled out at sites in a random order. Once the intervention is initiated at a site, all participants within that site remain exposed to the intervention for the remainder of the study.

The time since the start of the study (“calendar time”) may affect outcome measures through underlying time trends or periodicity. The time since the intervention was introduced to a site (“exposure time”) may also affect outcomes cumulatively for successful interventions, possibly in addition to a step change when the intervention began.

**Methods:**

Motivated by a SWCRT of self-monitoring for bipolar disorder, we conducted a simulation study to compare model formulations to analyse data from a SWCRT under 36 different scenarios in which time was related to the outcome (improvement in mood score). The aim was to find a model specification that would produce reliable estimates of intervention effects under different scenarios. Nine different formulations of a linear mixed effects model were fitted to these datasets. These models varied in the specification of calendar and exposure times.

**Results:**

Modelling the effects of the intervention was best accomplished by including terms for both calendar time and exposure time. Treating time as categorical (a separate parameter for each measurement time-step) achieved the best coverage probabilities and low bias, but at a cost of wider confidence intervals compared to simpler models for those scenarios which were sufficiently modelled by fewer parameters. Treating time as continuous and including a quadratic time term performed similarly well, with slightly larger variations in coverage probability, but narrower confidence intervals and in some cases lower bias. The impact of misspecifying the covariance structure was comparatively small.

**Conclusions:**

We recommend that unless there is a priori information to indicate the form of the relationship between time and outcomes, data from SWCRTs should be analysed with a linear mixed effects model that includes separate categorical terms for calendar time and exposure time. Prespecified sensitivity analyses should consider the different formulations of these time effects in the model, to assess their impact on estimates of intervention effects.

## Introduction

A stepped wedge cluster randomised trial (SWCRT) is a special case of a cross-over cluster randomised trial, in which the direction of cross-over is always from the control condition to the intervention condition [1]. Although the parallel cluster randomised trial is the gold standard, the SWCRT design is an appropriate option for large-scale intervention roll-outs when it is logistically infeasible to deploy the intervention at several clusters simultaneously. In SWCRTs, the intervention is rolled out at the cluster level, ensuring minimal risk of contamination between treatment and control subjects. One of the benefits of a SWCRT is that, at each time step, resources can be concentrated at the cluster where the intervention is being introduced, rather than resources spread across all intervention clusters simultaneously, as would be the case in a parallel cluster randomised trial design. A systematic review found that 21 SWCRT studies published between 2010 and 2014 listed logistical barriers to rolling out an intervention simultaneously at multiple centres as the reason for choosing the SWCRT design [2].

Particularly if an intervention has performed well during individual level trials, decision makers may view the intervention as doing more good than harm and may favour a design where all clusters will be exposed to the intervention at some point [2–4]. Under a parallel design, some clusters would not have the opportunity to be exposed to the intervention, which may be viewed as undesirable or unethical. If there is a strong view that the intervention works, clusters may be inclined to drop out of the study if not randomised to the intervention, and this has been used as justification for the selection of a SWCRT design in several studies [2]. The cross-over design is an alternative, but it may not be practical or possible to revert to “pre-intervention” conditions once the intervention has been introduced. Consequently, a SWCRT may be prescriptive rather than a preferred trial design, providing an option in which the intervention can still be tested at the cluster level without the encumbrances of a standard parallel cluster design.

The implementation of a community health insurance scheme in West Africa is an example where a SWCRT was used to assess the impact of a community-level intervention [5]. In this example, an SWCRT design was incorporated into the implementation of a scheme that had already been approved, allowing the impact on health resource utilisation and household protection to be assessed. The measurement units were individual households, located within 33 villages and towns (‘clusters’) to which the health insurance scheme was made available at a rate of 11 clusters per year. Another example of the use of the SWCRT design was a trial that assessed a feedback intervention aimed at producing sustained improvements in hand-hygiene compliance across 16 acute care hospitals in England and Wales [6]. The justification for the use of this design was a successful pilot, and a desire to reduce contamination and disappointment effects in hospitals not randomised to the intervention.

In a standard parallel cluster randomised trial, for a given intracluster correlation coefficient (ICC), it is most efficient to have many small clusters as opposed to a few large clusters [7]. When the clusters are limited in number, the cluster size needs to increase according to the ICC to acquire a required power, with larger ICC leading to larger required cluster sizes. When clusters are few and ICCs large, then the SWCRT design is more efficient than the parallel cluster randomised trial design, owing to each cluster having both non-exposure and exposure to the intervention at some point during the study period [1]. The number of clusters in SWCRT are usually smaller than typically expected for cluster randomised trials, consistent with the need to conserve or concentrate resources [1].

SWCRTs generally require data to be collected at each time step in all clusters both before and after the intervention is introduced. This can be burdensome to trial participants [4], unless long term monitoring is already in place or data acquisition is not intensive.

In SWCRTs, some clusters will be allocated to the intervention much earlier than others, and so there will be non-contemporaneous data from the intervention and the control periods. For this reason, differences in outcomes between the intervention and control periods may be confounded with “nuisance” factors associated with the outcome which influence how the outcome changes through time. Examples include changes in disease prevalence or measurement methods, or outcomes that demonstrate seasonality or a long-term temporal trend for reasons unrelated to the study. Consequently, this effect of time, which we refer to as “calendar time” in this study, may need to be accounted for when estimating the effectiveness of interventions in SWCRTs [8].

An additional time effect relates to the length of time that individuals in different clusters have been exposed to the effects of the intervention, which we term “exposure time”. In SWCRTs, exposure time varies by cluster, and as exposure to the intervention may have either an immediate or a cumulative effect on outcomes, both types of effect may need to be accounted for in the analysis. However, there has been limited exploration of the way either of these time effects should be modelled when analysing SWCRT data [4, 8], and between studies there is great inconsistency in the methods used [9].

The purpose of this simulation study is therefore to compare different formulations of the linear mixed effects (LME) model to account for time effects in stepped wedge cluster designs. LMEs account for both the correlation between repeated measurements from the same subject and the correlation between measurements from subjects in the same cluster, but methods for incorporating time effects to achieve correct inference about intervention effects are less clear. For example, time can be incorporated either as a continuous or categorical fixed effect, or via a random effect that allows for cluster specific intercepts and slopes in the outcome’s response over time [10].

It is recognised that trial statistical analysis plans may require a precise model formulation to be specified before any data analysis takes place. It is therefore desirable to identify a model formulation that performs well in estimating intervention effects in SWCRTs across a range of scenarios with differing calendar time and exposure time effects. We aim to identify such a model by fitting different variants of the basic LME to simulated data with known time and intervention effect parameters.

The paper is structured as follows. In the next section we introduce a motivating example relating to a trial (‘OXTEXT-7’) of an intervention for improving mood scores in individuals with bipolar disorder, which was expanded to include other patient groups with mental health disorders such as depression, substance abuse, anxiety and psychosis. After reviewing methodological considerations and a class of models for the analysis of SWCRTs, we perform a simulation study using a range of scenarios, with parameter values guided by the data obtained in the OXTEXT-7 trial. We assess the performance of the proposed models in estimating intervention effects under each of these scenarios to identify models that demonstrate the best performance overall and relate these findings to the trial results. The final section is a concluding discussion.

## Methods

This paper reports the results of the analysis of simulated datasets, and the secondary analysis of anonymised data from a previously published study. The University of Oxford does not require ethics approval for a secondary analysis of anonymised data. The study protocol of the original OXTEXT-7 trial was reviewed and approved by a UK NHS Ethics Committee.

### Motivating study

The simulation study was motivated by the OXTEXT-7 trial (ISRCTN16778756) [11]. This was a SWCRT run within eleven community mental health teams (CMHTs) in the Oxford Health NHS Foundation Trust. Each CMHT was randomised to a start date for “Feeling Well with TrueColours” (FWTC), which is an intervention originally aimed at individuals with bipolar disorder. The design of the study allowed for outcomes to be collected for three months under the control condition at the beginning of the study and for three months under the intervention condition at the end of the study over all CMHTs. This intervention makes use of technology that allows participants to text or email their responses to simple health-related questions with the aim of monitoring their mood prospectively. FWTC was offered to individuals with bipolar and other related mental health disorders whom the clinician (doctor, nurse, psychologist, other therapist) felt would benefit from developing self-monitoring and self-management skills. The intervention comprised two elements: a) self-monitoring of symptoms via the TrueColours system, and b) patient education about self-monitoring, via the ‘Feeling Well’ materials. The FWTC is a mood management approach built on the TrueColours platform, which aimed to help people through psychoeducation to learn about factors that could de-stabilise their mood and what steps the individual themselves could take to improve their mood stability. Central to such learning is accurate recording of, and feedback about, mood states.

The primary objective was to determine whether CMHTs which delivered the FWTC achieved better health outcomes for the participants in their care than teams that were not delivering the service, as determined by Health of the Nation Outcome Scales (HoNOS) total score [12]. The use of HoNOS is recommended by the English National Service Framework for Mental Health and by the UK Department of Health as an outcome to assess severe mental illness [13]. The instrument consists of 12 items, where each item is scored from 0 (no problem) to 4 (severe/very severe), and therefore the total score is out of 48. HoNOS total scores of 9 are typical of psychiatric out-patients [14]. The items cover four areas of mental health related to behaviour, impairment, symptoms and social functioning.

**Fig 1:**
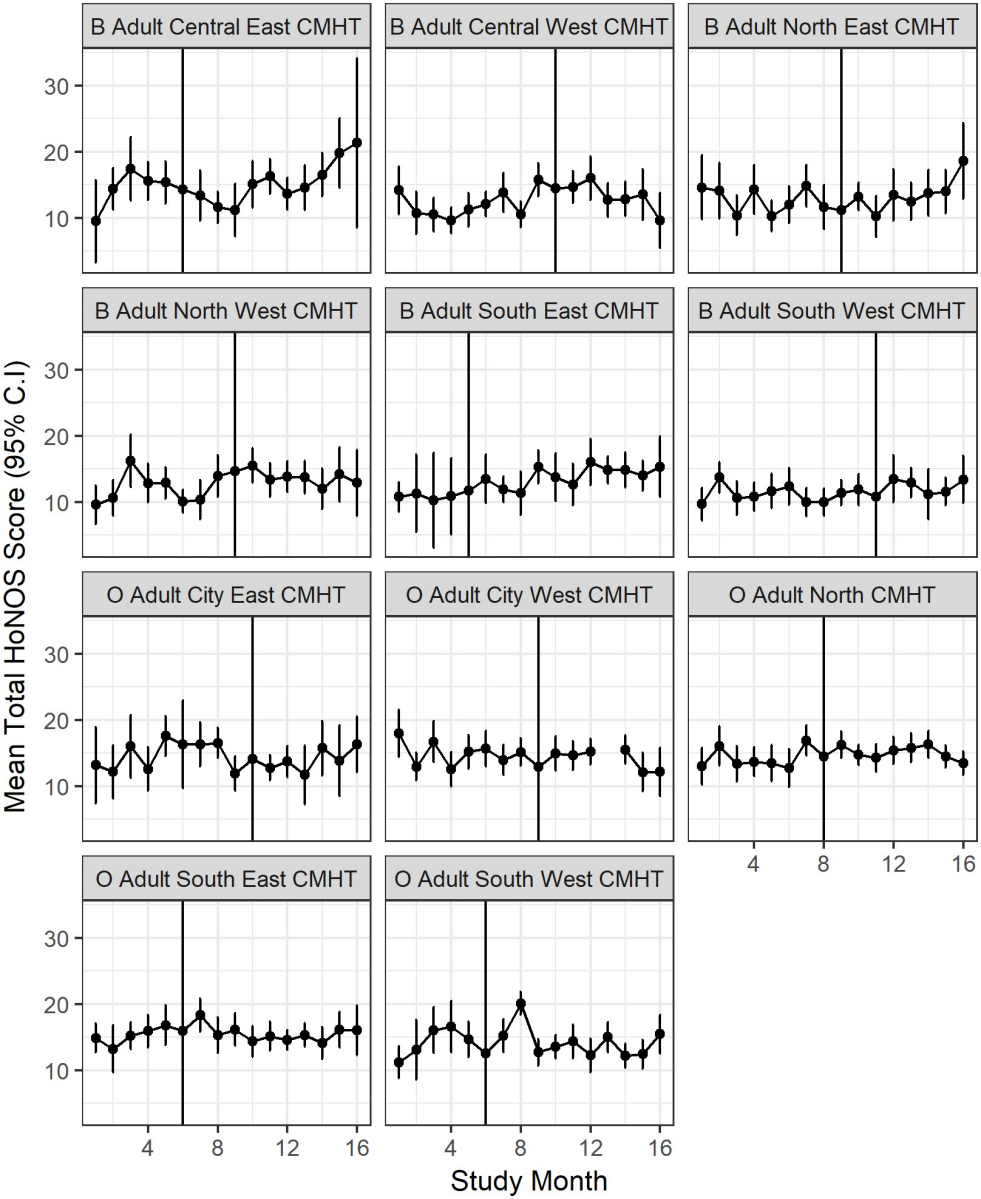
Mean plots with 95% confidence intervals (C.I.) of the total HoNOS scores plotted against the calendar time (study months) at each CMHT for the OXTEXT-7 study. Data to the left of the vertical line occurred before the intervention and data to the right after the intervention was introduced.

The mean Total HoNOS scores plotted against time since the introduction of the intervention for each CMHT during the OXTEXT-7 study period are presented in Fig 1. The mean plots suggest that across clusters the scores were lower at the start of the study period than at the end, with mean scores ranging between 10 and 14 between clusters at the beginning of the study, and between 10 and 21 at the end of the study with many clusters having means above 14. We used the characteristics of the HoNOS data collected from the OXTEXT-7 participants to create simulated datasets, as described in detail in the section ‘Simulations’.

### Stepped wedge design characteristics

**Fig 2:**
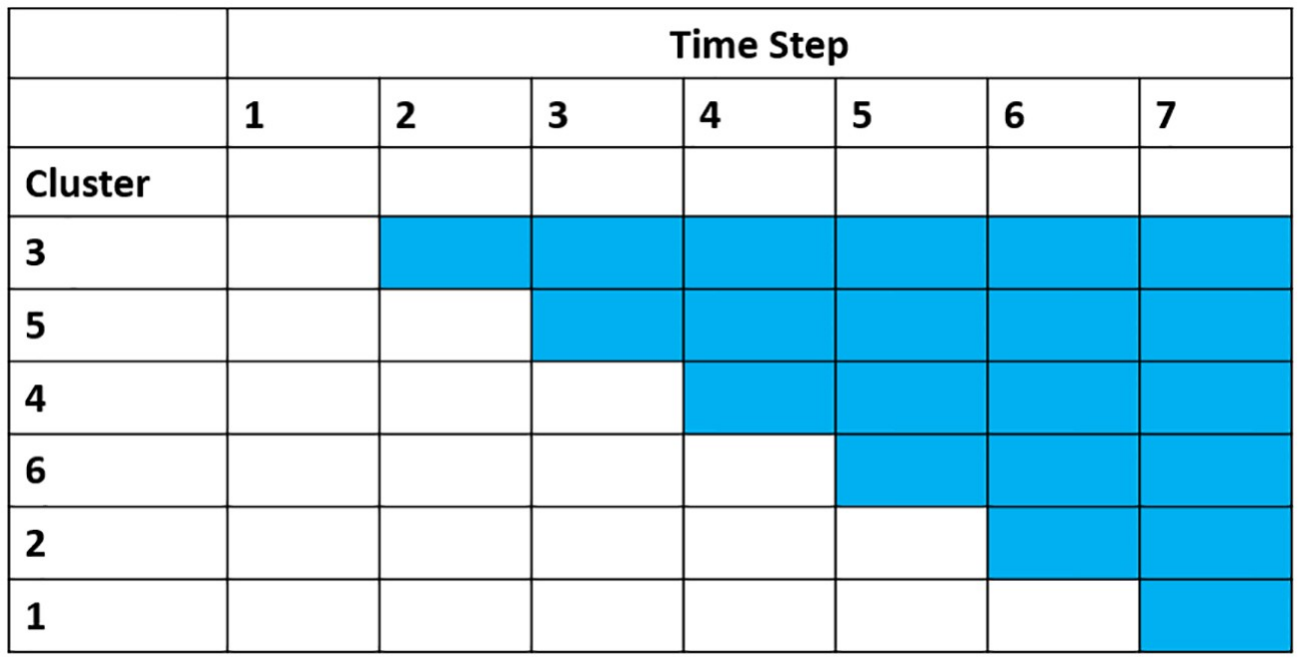
Graphical representation of the standard stepped wedge design intervention roll-out.

In the standard stepped wedge design there will be one more time step than there are clusters (Fig 2). For convenience, these time steps are assumed to be the same time points at which assessments are made. All clusters start under the control condition, and baseline assessments are performed on all clusters before the intervention is introduced. One cluster, selected at random, is then assigned to receive the intervention at the start of each subsequent time step. The outcome measures can either be obtained from new participants at each measurement occasion (cross-sectional SWCRT) or from the same participants at each measurement occasion (cohort SWCRT). In this study we consider only the closed cohort SWCRT, in which each participant will be exposed to both the control and intervention conditions at different times and each participant is present from the start to the end of the study period. The analysis of a cross-sectional SWCRT will be slightly easier as only correlation within the same cluster needs to be considered, as opposed to a cohort SWCRT which needs to account for both cluster and individual level correlation.

### Time

**Fig 3:**
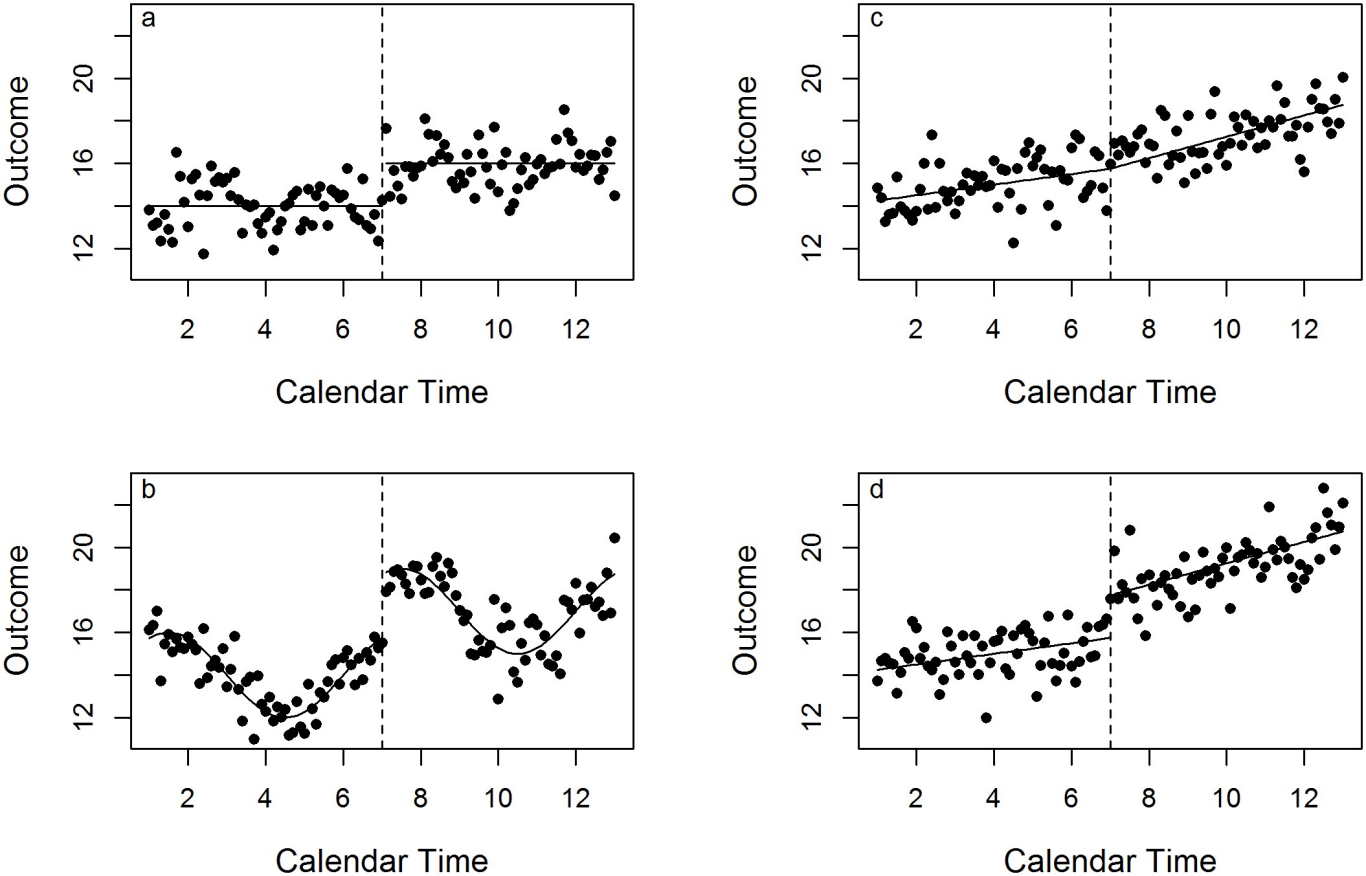
Four potential ways that an outcome can change as a function of time. The dashed line represents when the intervention is introduced. See text for further details. a.) Step change, no time trend, b.) Linear trends in calendar and exposure time, c.) Step change, non-linear trend in calendar time, d.) Step change, linear trends in calendar and exposure time.

In SWCRTs, outcomes may be related to calendar time and exposure time in different ways. The outcome may show no trend in relation to either calendar time or exposure time, but the intervention may cause a step change, represented by a higher mean value (Fig 3a). Alternatively, there could be a trend in relation to calendar time, either linear or non-linear (Fig 3b), allied to the step change. Additionally, there may be a trend in relation to exposure time, as illustrated by a change in the gradient, with or without a step change at the time the intervention is introduced (Fig 3c and 3d). The method of analysis needs to be flexible enough to be able to account for different types of responses over time. In the ‘Simulations’ section we describe how datasets were simulated with these different responses over time in mind.

### Models

In this section we describe the LME models which were considered as candidates for analysing data from a SWCRT. A summary of mathematical notation is provided in Table 1. We use the subscript *i* to denote participants where *i* = 1,…, *N*, *t* to denote time steps (calendar time) where *t* = 1,…, *n*, and *k* to denote clusters (mental health trusts) where *k* = 1,…, *K*. For the sake of reducing the amount of notation, we assume that the value of calendar time at the *t*th time step is equal to *t*. The notation *y*_*itk*_ represents the outcome (such as the HoNOS score) for participant *i* at time step *t* in cluster *k*, and *x*_*tk*_ is a binary indicator for whether the cluster *k* is in the intervention at time step *t*. When we treat time as continuous then *y*_*itk*_ is assumed to be a function of *t* and so *y*_*itk*_ = *y*_*itk*_ (*t*).

**Table 1:** Summary of mathematical notation.

*i*	Participant subscript *i* = 1, …, *N*
*t*	Time steps (calendar time) *t = 1*,…, *n*
*k*	Cluster subscript *k* = 1,…, *K*
*y*_*itk*_	Outcome (HoNOS score) for participant *i* at time step *t* in cluster *k*.
*y*_*itk*_(*t*)	Outcome (HoNOS score) for participant *i* in cluster *k* as a function of continuous calendar time equal to *t*.
*x*_*tk*_	Binary indicator for whether cluster *k* is under the intervention condition at time step *t*.
*β*_0_	Intercept of the LME model
*∂*	Intervention effect (coefficient of *x*_*tk*_ in the LME model)
*v*_0*ik*_	Random effect with mean zero and variance $\sigma_{\nu }^{2}$
*γ*_0*k*_	Cluster-specific random intercept with mean zero and variance $\sigma_{\gamma }^{2}$
*h*_0*i*_	Participant-specific random intercept with mean zero and variance $\sigma_{h}^{2}$
*ϵ*_*itk*_	Random error with mean zero and variance *σ*^2^ conditional on *v*_0*ik*_
*τ*	Slope of calendar time (coefficient of *t* in the LME model)
*κ*_*t*_	Coefficient of the binary indicator for categorical time *t* in the LME model
*ω*	Coefficient for the interaction between the binary indicator for the intervention *x*_*tk*_ and continuous calendar time *t*
*φ*_*t*_	Coefficient of binary indicator for interaction between intervention indicator *x*_*tk*_ and categorical calendar time *t*
*d*_*tk*_	Exposure time to intervention at calendar time *t* for cluster *k*
*ψ*	Coefficient of continuous exposure time *d*_*tk*_
*ξ*_*d*_	Coefficient of binary indicator for categorical exposure time *d*, where *d* = *d*|*k*, *t*
*ζ*	Coefficient of quadratic calendar time *t*^2^
*ρ*	Within-participant correlation
ICC	Intracluster correlation coefficient
*r*	Difference in calendar times between two measurements
*μ*_*it*_	Sum of fixed effects in LME model
*s*_*itk*_	Independent component of participant-level random error

The simplest LME model which can be used to analyse data from a SWCRT is:

$$y_{itk}=\beta_{0}+\partial x_{tk}+v_{0ik}+\epsilon_{itk}$$ (1)

where, *β*_0_ is the intercept, *∂* is the intervention effect, and *v*_0*ik*_ is a random effect term, which can be expressed as:

$$v_{0ik}=\gamma_{0k}+h_{0i}$$

where *γ*_0*k*_ is a cluster-specific random intercept, and *h*_0*i*_ is a random intercept for the *i*th participant. These random effect components are usually assumed to be normally distributed and mutually independent, such that *v*_0*ik*_ has zero mean and variance $\sigma_{\nu }^{2}=\sigma_{\gamma }^{2}+\sigma_{h}^{2}$. The random errors *ϵ*_*itk*_ are assumed to be normally and independently distributed, conditional on *v*_0*ik*_, with mean zero and variance *σ*^2^. The covariance matrix of the vector of responses for participant *i* then has a compound symmetrical (CS) structure, where the diagonal elements equal $\sigma^{2}+\sigma_{\nu }^{2}$ and the off-diagonal elements equal $\sigma_{\nu }^{2}$.

It is possible to impose a within-subject covariance matrix with an alternative structure [15]. We consider fitting an autoregressive AR(1) structure as an alternative to the CS structure.

An alternative approach, which explicitly accounts for a linear trend with respect to calendar time, is [10]:

$$y_{ik}(t)=\beta_{0}+\partial x_{tk}+\tau t+v_{0ik}+\epsilon_{ijk}$$ (2)

where *τ* is the slope over calendar time, and *t* is the calendar time, which is treated here as continuous.

The model proposed by Hussey and Hughes [8] and recommended by Hemming et al [1] and Barker et al [4] includes instead a categorical variable for calendar time as a fixed effect:

$$y_{itk}=\beta_{0}+\partial x_{tk}+\kappa_{t}+v_{0ik}+\epsilon_{itk}$$ (3)

where **κ** = (0, *κ*_2_, *κ*_3_, …, *κ*_*t*_, …, *κ*_*n*_) is a vector of parameters that allows a different calendar time effect, *κ*_*t*_, at each time step *t*. In Models 2 and 3, *∂* can be interpreted as a time-averaged intervention effect.

Models that account for both a step change in the outcome once the intervention is introduced and a change in the response over calendar time can be specified in a number of ways. A simple approach is to include the interaction between calendar time and intervention, treating time as either continuous or categorical:

$$y_{ik}(t)=\beta_{0}+\partial x_{tk}+\tau t+\omega x_{tk}t+v_{0ik}+\epsilon_{itk}$$ (4)

$$y_{itk}=\beta_{0}+\partial x_{tk}+\kappa_{t}+\varphi_{t}x_{tk}+v_{0ik}+\epsilon_{itk}$$ (5)

where *∂* is the estimate of the intervention effect at the first time step in which the intervention was available (*t* = 2), *ω* is the coefficient for the interaction between the binary indicator for the intervention *x*_*tk*_ and continuous calendar time *t*, and *φ*_*t*_ is an estimate of the additional effect of the intervention at categorical calendar time *t*, where *t* = 3,…, *n-1*. *φ*_*t*_ = 0 when *t* = 1, 2, *n*. Practically, this involves creating a set of *n-4* variables when *t* = 3,…, *n-1* that equals one when cluster *k* is under the intervention and zero otherwise. This is to ensure that all model parameters are identifiable. The intervention is in place for all clusters at time step *t = n*, and therefore the difference between the intervention and the control condition cannot be estimated at *t = n*. In a standard parallel cluster randomised trial, there would be a parameter *φ*_*n*_ which would have corresponded to the additional effect of the intervention due to *n*th calendar time. In the SWCRT there are no data at the *n*th calendar time to estimate the outcome under the control condition, and therefore the parameter *φ*_*n*_ is incomputable when the effect on the outcome due the *n*th calendar time (*κ*_*n*_) is estimated as well. As the data available at the *n*th calendar time are all under the intervention, *κ*_*n*_ is an estimate of the additional effect on the outcome due to calendar time *n* when under the intervention.

Alternatively, models might relate the exposure time, *d*, to the outcome, as in Fig 3d:

$$y_{ik}(t)=\beta_{0}+\partial x_{tk}+\tau t+\psi d_{tk}+v_{0ik}+\epsilon_{itk}.$$ (6)

Here, *d*_*k*_(*t*) = *d*_*tk*_ is the length of time the intervention has been in place in cluster *k* to which participant *i* belongs, between the time of its introduction and time step *t*. It is equal to zero while the cluster is under the control condition. The parameter *ψ* is the model coefficient for exposure time. The intervention term could be excluded if it is assumed that the intervention will not cause an immediate change to the outcome (Fig 3c):

$$y_{ik}(t)=\beta_{0}+\tau t+\psi d_{tk}+v_{0ik}+\epsilon_{itk}.$$ (7)

As in Model 3, calendar time and exposure time may be treated as categorical:

$$y_{itk}=\beta_{0}+\partial x_{tk}+\kappa_{t}+\xi_{d}+v_{0ik}+\epsilon_{itk}$$

where **ξ** = (0, *ξ*_1_, *ξ*_2_, …, *ξ*_*d*_, …, *ξ*_*n*−1_) is a vector of parameters where *ξ*_*d*_ = *ξ*_*d*|*k*,*t*_ is the specific effect of *d* time steps of exposure to the intervention, where *d* is determined by cluster *k* and time step *t*. If all clusters start on the control condition at time step 1 then there can be maximum *n-1* time steps under the intervention. When time is treated as categorical, including a term for the intervention is redundant because intervention is completely nested within exposure time [16]. Therefore, the model simplifies to:

$$y_{itk}=\beta_{0}+\kappa_{t}+\xi_{d}+v_{0ik}+\epsilon_{itk}.$$ (8)

Model 8 can be considered as a more general version of the Hussey and Hughes formulation [8], where instead of having a single time-averaged intervention effect, there is a different intervention effect for each level of exposure.

Finally, models might include non-linear time effects. For example, Model 6 might be extended to:

$$y_{ik}(t)=\beta_{0}+\partial x_{tk}+\tau t+\psi d_{tk}+\zeta t^{2}+v_{0ik}+\epsilon_{itk}$$ (9)

where *ζ* is the model coefficient for quadratic time.

### Simulations

We generated simulated datasets under 36 different scenarios, guided by the methods outlined in [17], using R statistical software. All scenarios considered a study conducted over 13 months (time steps) in 12 centres (clusters), where one centre was randomised to the intervention each month except during the first time step. A cluster-specific ICC of 0.03, as derived from the original data, was used to estimate the required sample size for the simulation study, which was 20 individuals per cluster if there were 12 clusters using the methods outlined in [18]. This would provide 80% power to detect a difference of 1.2 HoNOS units at an alpha of 5%, assuming a standard deviation of 6.94. To mimic the trial results, the total HoNOS score was simulated to increase through time for most of the scenarios. The repeated measurements from the same participant in the OXTEXT-7 trial produced an estimate of -0.5 for the correlation parameter, *ρ*. We considered both *ρ* = -0.5 and *ρ* = 0.5. Within cluster correlation was modelled by means of a random effect. To perform the simulations we used the patient-level and cluster-level variance components as estimated from the OXTEXT 7 trial data, rather than basing the simulation on the ICC of 0.03, which was calculated assuming a single clustering level as required by the sample size estimation method, due to the presence of both patient and cluster-level correlation.

Fixed effect parameters were simulated according to Table 2. Random effects and random errors were estimated in the same way for all simulated scenarios. The variance components and correlations are also provided in Table 2. It was assumed that the cluster-level random effect had distribution *γ*_0*k*_ ~ *N*(0, 0.96^2^) and the patient-level random effect had distribution *h*_0*i*_ ~ *N*(0, 4.42^2^). These terms were estimated from the variance components produced from a LME model fit to the original dataset, specifying nested random effects. We assumed a simple AR(1) structure for the within-subject covariance matrix, which assumes the same variance for each time step and that the correlation between measurements from the same individual *r* time steps apart equals *ρ*^*r*^, where *ρ* is a correlation parameter. The random error *ϵ*_*itk*_ was assumed to be normally distributed with zero mean and covariance matrix with diagonal elements equal to *σ*^2^ and off-diagonal elements equal to *σ*^2^*ρ*^*r*^, where *r* is the difference in calendar time steps and *σ*^2^ was estimated from the original data to be 5.44^2^ and *ρ* set to be either -0.5 or 0.5. To produce simulated data with this covariance structure, individual observations were simulated such that

$$y_{itk}=\mu_{it}+v_{0ik}+\rho \epsilon_{i,t-1,k}+s_{itk}$$

where *μ*_*it*_ represents the fixed effects, *ϵ*_*itk*_ = *ρϵ*_*i*,*t*−1,*k*_ + *s*_*itk*_ is the random error for *t* > 1, *ϵ*_*i*,1,*k*_ ~ *N*(0,*σ*^2^) and *s*_*itk*_ ~ *N*(0,(1 − *ρ*^2^) *σ*^2^) is the independent component of the random error. This rescaled variance for *s*_*itk*_ ensures that the total variance from the random error for subject *i* in cluster *k* is equal to *σ*^2^ for each *t* [17].

**Table 2:** Parameters used to simulate datasets.

**Simulation Parameters**	
*Mean Model*	
Intercept (*β*_0_) = 14.00 units	
Intervention effect (*∂*) = 2 OR -2 units	
Linear time trend (*τ*) = 0.25 units per month	
Intervention additional time trend (*ψ*) = 0.15 OR 0.25 OR -0.50 units per month	
Non-linear calendar time trend $2sin(\frac{(t-1)\pi }{12})$ {for scenarios D5, D6, D25-D30}	
such that **κ**_**1**_ ∈ (0, 0.52, 1.00, 1.41, 1.73, 1.98, 2.00, 1.93, 1.73, 1.41, 1.00, 0.52, 0)	
Non-linear calendar time trend $2sin(\frac{(t-1)\pi }{6})$ {for scenarios D7, D8, D31-D36}	
such that **κ**_**2**_ ∈ (0, 1.00, 1.73, 2.00, 1.73, 1.00, 0, -1.00, -1.73, -2.00, -1.73, -1.00, 0)	
where *t* ∈ (1, 2, 3, 4, 5, 6, 7, 8, 9, 10, 11, 12, 13)	
Non-linear exposure time trend $sin(\frac{(d-1)\pi }{12})$ {for scenarios D25-D30}	
such that ***ξ***_**1**_ ∈ (0, 0, 0.26, 0.50, 0.71, 0.87, 0.97, 1.00, 0.97, 0.87, 0.71, 0.50, 0.26)	
Non-linear exposure time trend $sin(\frac{(d-1)\pi }{6})$ {for scenarios D31-D36}	
such that ***ξ***_**2**_ ∈ (0, 0, 0.50, 0.87, 1.00, 0.87, 0.50, 0, -0.50, -0.87, -1.00, -0.87, -0.50)	
where *d* ∈ (0, 1, 2, 3, 4, 5, 6, 7, 8, 9, 10, 11, 12)	
**Fixed Effects Parameterisations for each Scenario**	
D1, D2: *y*_*itk*_ = 14	D19, D20: *y*_*ik*_(*t*) = 14 + 0.25*t* + 0.25*d*_*tk*_
D3, D4: *y*_*ik*_(*t*) = 14 + 0.25*t*	D21, D22: *y*_*ik*_(*t*) = 14 − 2*x*_*tk*_ + 0.25*t* − 0.50*d*_*k*_
D5, D6: *y*_*itk*_ = 14 + *κ*_1*t*_	D23, D24: *y*_*ik*_(*t*) = 14 + 0.25*t* − 0.50*d*_*tk*_
D7, D8: *y*_*itk*_ = 14 + *κ*_2*t*_	D25, D26: *y*_*itk*_ = 14 + 2*x*_*tk*_ + *κ*_1*t*_
D9, D10: *y*_*itk*_ = 14 + 2*x*_*tk*_	D27, D28: *y*_*itk*_ = 14 + 2*x*_*tk*_ + *κ*_1*t*_ + *ξ*_1*d*_
D11, D12: *y*_*ik*_(*t*) = 14 + 2*x*_*tk*_ + 0.25*t*	D29, D30: *y*_*itk*_ = 14 + *κ*_1*t*_ + *ξ*_1*d*_
D13, D14: *y*_*ik*_(*t*) = 14 + 2*x*_*tk*_ + 0.25*t* + 0.15*d*_*tk*_	D31, D32: *y*_*itk*_ = 14 + 2*x*_*tk*_ + *κ*_2*t*_
D15, D16: *y*_*ik*_(*t*) = 14 + 0.25*t* + 0.15*d*_*tk*_	D33, D34: *y*_*itk*_ = 14 + 2*x*_*tk*_ + *κ*_2*t*_ + *ξ*_2*d*_
D17, D18: *y*_*ik*_(*t*) = 14 + 2*x*_*tk*_ + 0.25*t* + 0.25*d*_*tk*_	D35, D36: *y*_*itk*_ = 14 + *κ*_2*t*_ + *ξ*_2*d*_

*y*_*itk*_ is the HoNOS score for participant *i* at time step *t* in cluster *k*, *x*_*tk*_ is an indicator variable for whether at time step *t* cluster *k* was under the control or intervention condition, *t* is the calendar time, *d*_*tk*_ is the exposure time to the intervention in cluster *k* at calendar time *t*, **κ**_**1**_ and **κ**_**2**_ are sets of parameters corresponding to the non-linear calendar time coefficients, **ξ**_**1**_ and **ξ**_**2**_ are sets of model parameters for the effects of different non-linear exposure times *d* to the intervention.

**Fig 4:**
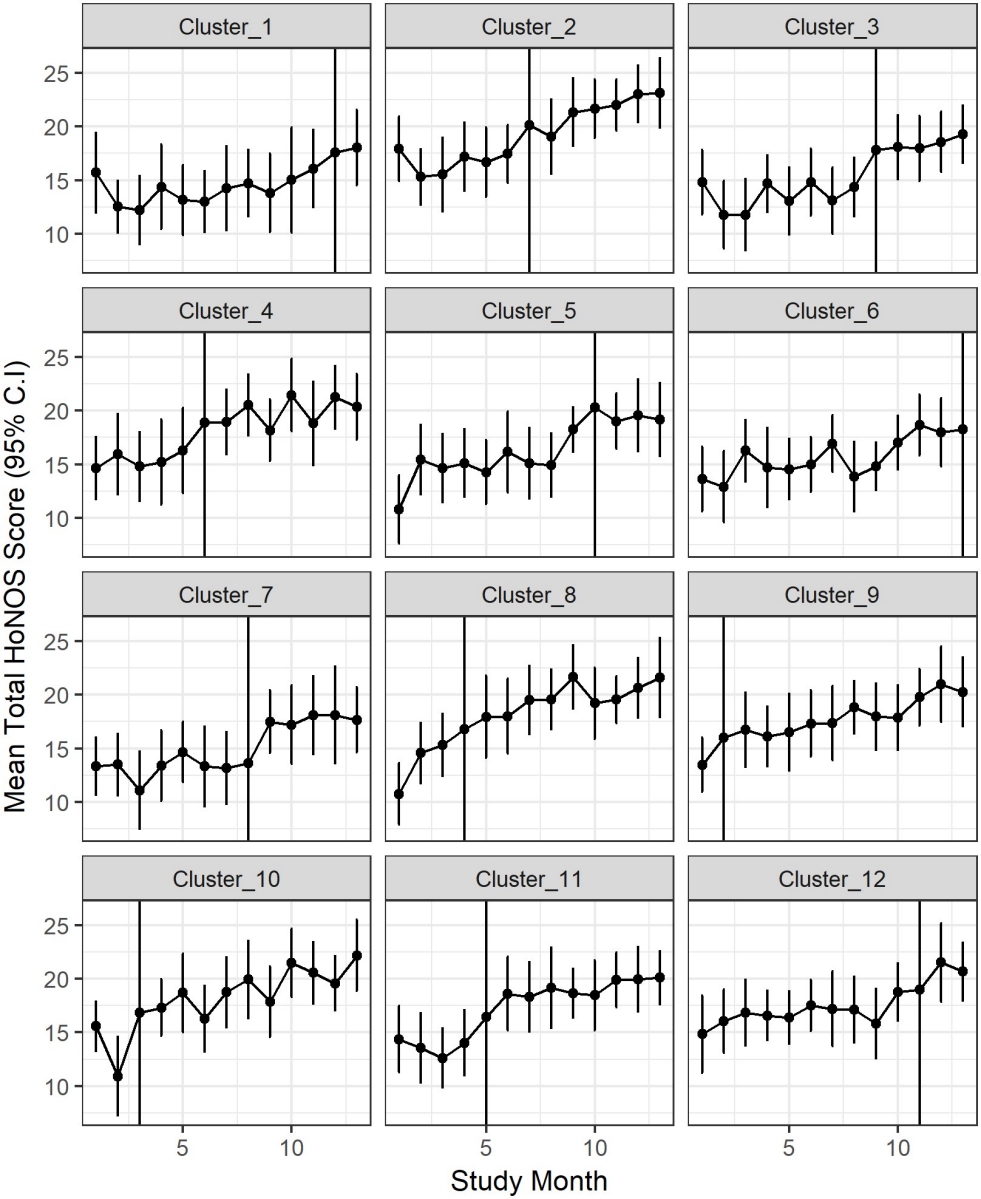
The simulated data under scenario D17: *y*_*ik*_(*t*) = 14 + 2*x*_*tk*_ + 0.25*t* + 0.25*d*_*tk*_. The means and 95% confidence intervals are plotted against the time since the intervention was introduced. Data to the left of the vertical line occurred before the intervention and data to the right after the intervention was introduced.

A full list of the models used to simulate the 36 different scenarios is provided in Table 2. An example where the simulated HoNOS scores have a linear time effect and both an immediate intervention effect on the HoNOS score and the time effect changes after the intervention is introduced is presented in Fig 4. The figure demonstrates that even when the intervention effect is prominent in the data, it is not easy to distinguish this effect from a plot of the data over time.

Simulations were performed using the R built-in package stats and parameter estimates from the original data were determined using the R nlme package. Code for simulating the data are provided in S1 Appendix.

### Analysis

The nine models listed in Table 3, as described in the section ‘Models’, were used to analyse each of the simulated datasets. The structure of the within-subject covariance matrix was specified as either CS or AR(1), so in total 18 candidate models were fitted for each of the 36 scenarios. Models were fit by means of maximum likelihood estimation to ensure the Bayesian information criterion (BIC) could be used for model comparison. The procedure lme from the nlme statistical software package for R statistical software was used to fit the linear mixed effects models and the package multcomp to obtain contrast estimates. Code is supplied in S2 Appendix.

**Table 3:** Model structures for linear mixed effects models fitted to simulated datasets.

*No time*:
*y*_*itk*_ = *β*_0_ + *∂x*_*tk*_ + *v*_0*ik*_ + *ϵ*_*itk*_ (Model 1)
Intervention effect at six months exposure: *∂*
Time-average intervention effect: *∂*
*Time Continuous*:
*y*_*ik*_(*t*) = *β*_0_ + *∂x*_*tk*_ + *τt* + *v*_0*ik*_ + *ϵ*_*ijk*_ (Model 2)
Intervention effect at six months exposure: *∂*
Time-average intervention effect: *∂*
*y*_*ik*_(*t*) = *β*_0_ + *∂x*_*tk*_ + *τt* + *ωx*_*tk*_*t* + *v*_0*ik*_ + *ϵ*_*itk*_ (Model 4)
Intervention effect at six months exposure: *∂* + 6*ω*
Time-average intervention effect: *∂* + 7*ω*
*y*_*ik*_(*t*) = *β*_0_ + *∂x*_*tk*_ + *τt* + *ψd*_*tk*_ + *v*_0*ik*_ + *ϵ*_*itk*_ (Model 6)
Intervention effect at six months exposure: *∂* + 6*ψ*
Time-average intervention effect: *∂* + 6*ψ*
*y*_*ik*_(*t*) = *β*_0_ + *τt* + *ψd*_*tk*_ + *v*_0*ik*_ + *ϵ*_*itk*_ (Model 7)
Intervention effect at six months exposure: 6*ψ*
Time-average intervention effect: 6*ψ*
*y*_*ik*_(*t*) = *β*_0_ + *∂x*_*tk*_ + *τt* + *ψd*_*tk*_ + *ζt*^2^ + *v*_0*ik*_ + *ϵ*_*itk*_ (Model 9)
Intervention effect at six months exposure: *∂* + 6*ψ*
Time-average intervention effect: *∂* + 6*ψ*
*Time Categorical*:
*y*_*itk*_ = *β*_0_ + *∂x*_*tk*_ + *κ*_*t*_ + *v*_0*ik*_ + *ϵ*_*itk*_ (Model 3) [8]
Intervention effect at six months exposure: *∂*
Time-average intervention effect: *∂*
*y*_*itk*_ = *β*_0_ + *∂x*_*tk*_ + *κ*_*t*_ + *φ*_*t*_*x*_*tk*_ + *v*_0*ik*_ + *ϵ*_*itk*_ (Model 5)
Intervention effect at six months exposure: *∂* + *φ*_6_
Time-average intervention effect: $\partial +\frac{1}{10}(\varphi_{3}+\varphi_{4}+\cdots +\varphi_{12})$
*y*_*itk*_ = *β*_0_ + *κ*_*t*_ + *ξ*_*d*_ + *v*_0*ik*_ + *ϵ*_*itk*_ (Model 8)
Intervention effect at six months exposure: *ξ*_6_
Time-average intervention effect: $\frac{1}{12}(\xi_{1}+\cdots +\xi_{12})$

The random effects, *v*_0*ik*_, and random errors, *ϵ*_*itk*_, are specified in the same way for each of the nine models, assuming either a CS structure for the within-subject variance-covariance matrix or an AR(1) structure. Therefore a total of 18 different model configurations were considered.

Two intervention effects were considered: the intervention effect at six months exposure to the intervention, and the time-averaged intervention effect over the whole study period. The estimated intervention effect was obtained by means of appropriate contrast statements, resulting in a linear combination of the model parameters corresponding to the intervention effect, together with the standard error and confidence interval for the estimate. Models were assessed based on the coverage probability of the 95% confidence interval of the intervention effect, the width of the 95% confidence interval of the intervention effect, the bias in estimating the intervention effect, the mean square error for the overall model fit to the simulate data, and the BIC assessing the overall fit.

If the intervention and time effects were assumed independent, such as in Models 1, 2 and 3, then the intervention effect at six months exposure and the time-averaged intervention effect would be equal to the parameter estimate for the intervention (*∂*). For those models with continuous exposure time (Models 6 and 9), the intervention effect after six months exposure would be equal to *∂* + 6*ψ* and equal to 6*ψ* for Model 7. As this corresponds to half of the total possible exposure time in the study (median of the set for *d* ∈ {0,2,…,12}), the time-averaged intervention effect is the same. For Model 8, where exposure time is categorical, the intervention effect at six months exposure would be the corresponding coefficient for exposure time *d* = 6 (*ξ*_6_), and the time-averaged intervention effect is the average of all the model coefficients for exposure time. The models with the interaction term (Models 4 and 5) provide a model for the outcome under the control condition over the whole study period, and likewise for the outcome under the intervention condition. This model implies that the intervention effect at a point in time is different to other times because the outcome responds to the intervention differently at each time point, and not because of a certain length of exposure to the intervention. To get the intervention effect after six months exposure, we have to assume that this would be the intervention effect as estimated for six months into the study period, and would be calculated as *∂* + 6*ω* for Model 4 and *∂* + *φ*_6_ for Model 5. The time-averaged intervention effect would be calculated half-way through the study period, which would be at a calendar time of 7 months (*∂* + 7*ω*) for Model 4 (median of the set for *j* ∈ {1,2,…,13}), and would be calculated as the sum of the intervention effect plus the mean of all the interaction coefficient terms for Model 5.

For each fitted model and for each simulated scenario, the coverage probability of the 95% confidence interval was calculated as the proportion of model fits where the confidence interval for the intervention effect contained the true value. Ideally, the coverage probability should be close to 95%. The confidence interval width, the MSE, and BIC were computed and their means obtained for each model fit over each set of 1000 simulations.

The nine candidate models were also fitted to the data from the OXTEXT-7 motivating example. The intervention effect after six months exposure time and time-averaged intervention effect were estimated from each model fit, and the overall model fit was assessed by means of the BIC statistic.

## Results

### Simulation study

**Fig 5:**
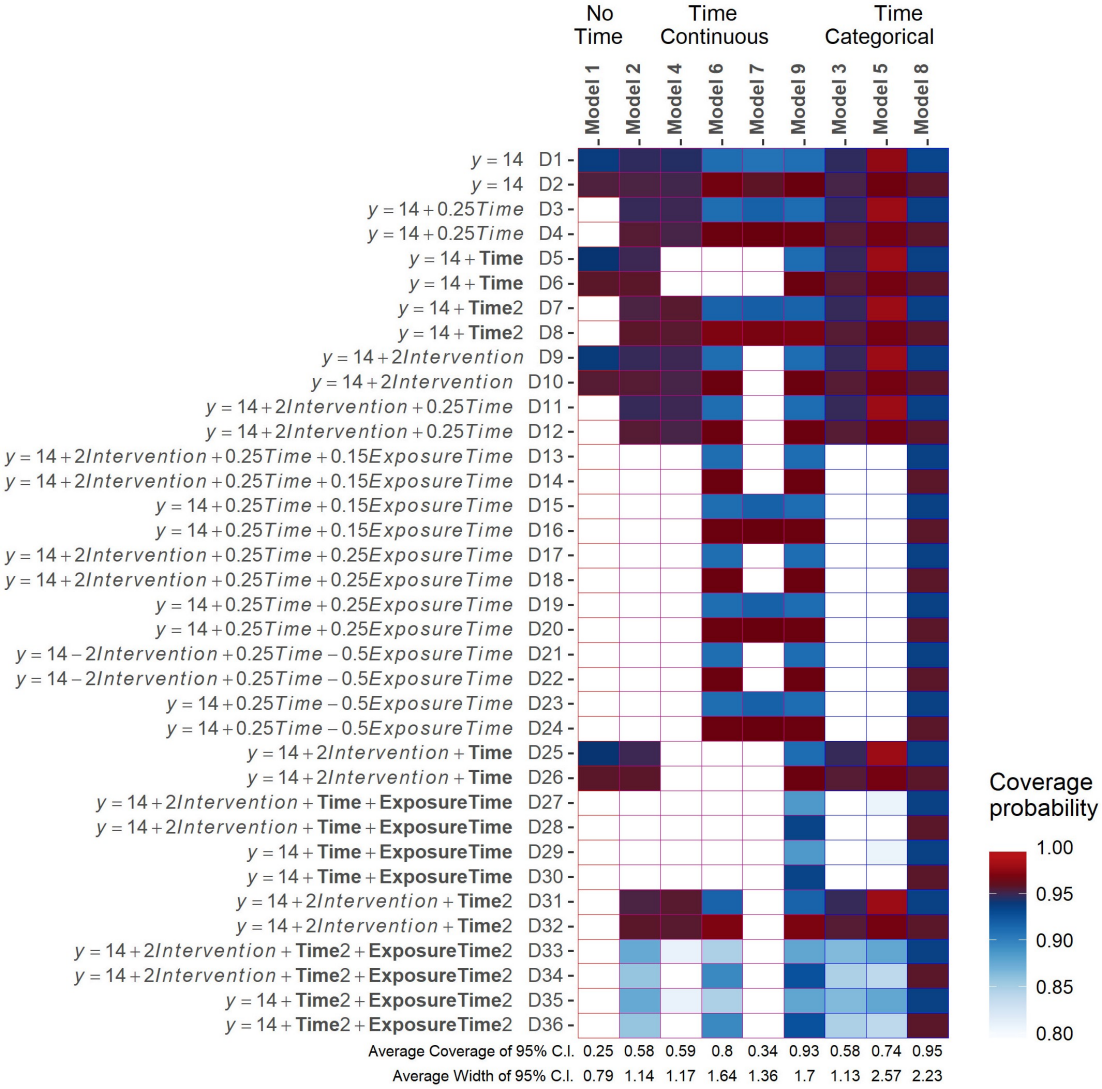
The heat map shows the coverage probability of the intervention effect at six months exposure for the nine fitted models with CS correlation structure. The heat map was very similar when the AR(1) structure was specified and for the time-averaged intervention effect. Values at the bottom of each column show the average coverage probability for each fitted model and the average width of the confidence interval for the intervention effect. Odd-numbered scenarios are simulated with *ρ* = -0.5 and even-numbered scenarios have *ρ* = 0.5.

The coverage probabilities for the intervention effect at six months exposure for each model fitted to each simulated scenario are provided in Fig 5, with the mean intervention effect estimates over all simulated datasets within each scenario provided in the tables in S3 Appendix. Model 8 had coverage probabilities close to 95% for all scenarios. Model 9 had coverage probabilities close to 95% except for those scenarios with a non-linear intervention effects (D27-D30 and D33-D36), where the coverage probabilities were lower than 95% but all close to or above 90%. Model 6 had similar coverage probabilities to Model 9, except for poor coverage for scenarios D5 and D6, and scenarios D25 to D30, therefore performing poorly for all scenarios where time was simulated as half a sinusoid cycle over the full study period. Models 2 and 3, which treated the intervention and calendar time independently, had coverage probabilities close to 95% only for those scenarios where time and the intervention effect were simulated as independent, and had poor coverage otherwise. Model 5 had poor coverage for all scenarios simulated with an effect of exposure time and had coverage probabilities that were higher than 95% for all other scenarios. Model 4, with continuous interaction term, had poorer coverage than Model 2, which treated time and the intervention effect independently. Model 7, which ignored the immediate effect of the intervention, and Model 1, which ignored time, had poor coverage probabilities for most scenarios.

When the structure of the covariance matrix of the within-subject observations was specified as AR(1), coverage probabilities were very similar and there were no differences in the estimates of the intervention effect after six months exposure, or any of the fixed effects model parameters, compared with those for the same mean model under the CS covariance structure (Tables J-R in S3 Appendix). Similar patterns in the coverage probabilities for the time-averaged intervention effect estimates were obtained. These plots are provided in S4 Appendix.

**Fig 6:**
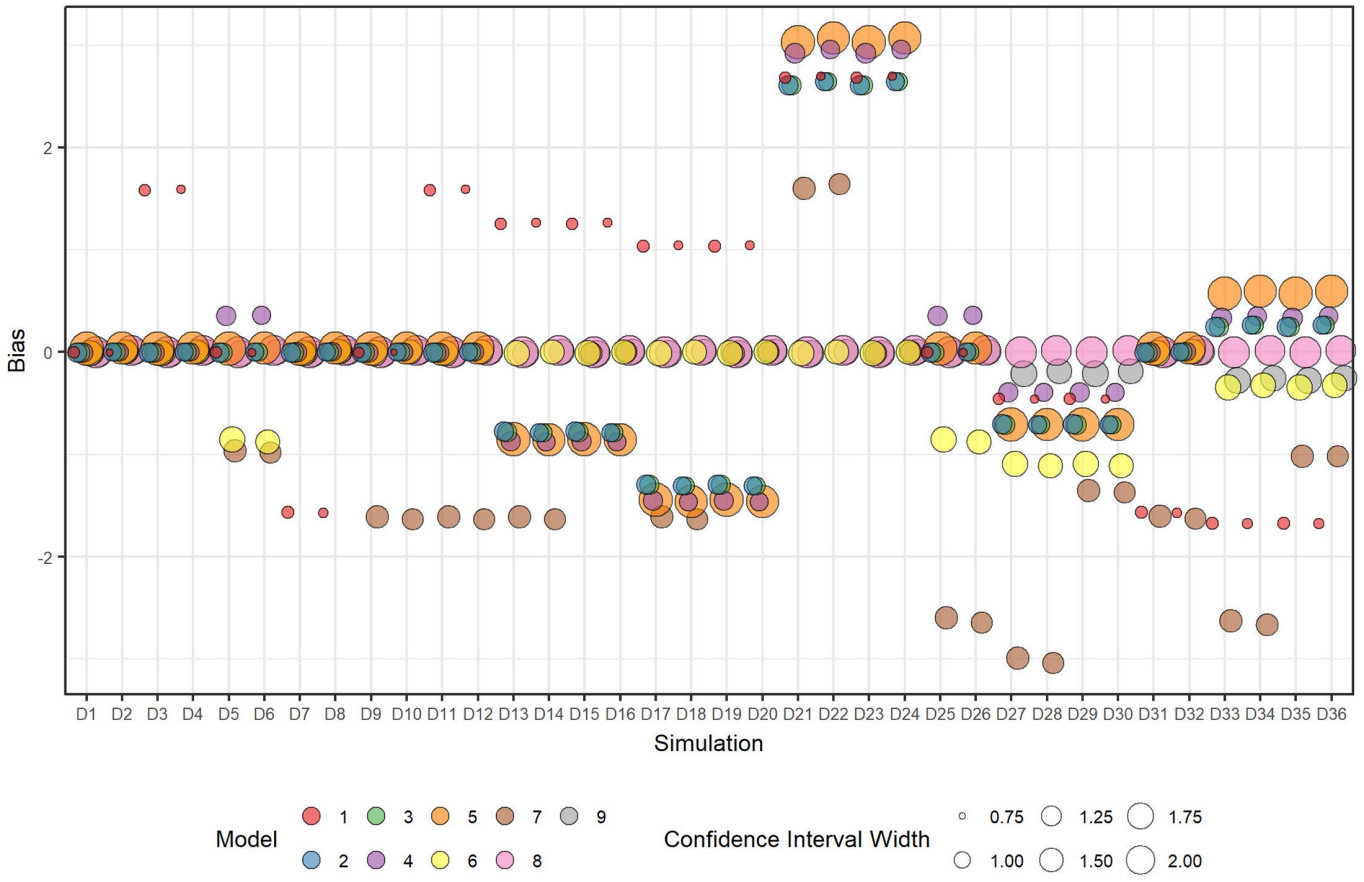
Plot of mean bias and mean 95% confidence interval width over all datasets within each scenario for each fitted model for the estimate of the intervention effect at six months exposure.

The biases in the intervention effect at six months exposure were close to zero across all scenarios for Models 8 and 9 (Fig 6), with Model 9 showing small biases for scenarios with non-linear intervention effects over time (D27-D30 and D33-D36). Model 6 achieved a similar bias close to zero for most scenarios, with exceptions for D5, D6, D25 to D26, as for the coverage probabilities. Models with interaction terms had relatively large biases for those scenarios with simulated exposure time effects. The width of the confidence intervals for Models 8 and 9 were similar, smaller than for Model 7, which had the widest intervals and relatively large biases, but wider than for Model 6. For each fitted model, the width of the confidence intervals did not differ between scenarios. As expected, models with fewer parameters, and therefore requiring fewer degrees of freedom to estimate parameters, had narrower confidence intervals for the intervention effects.

**Fig 7:**
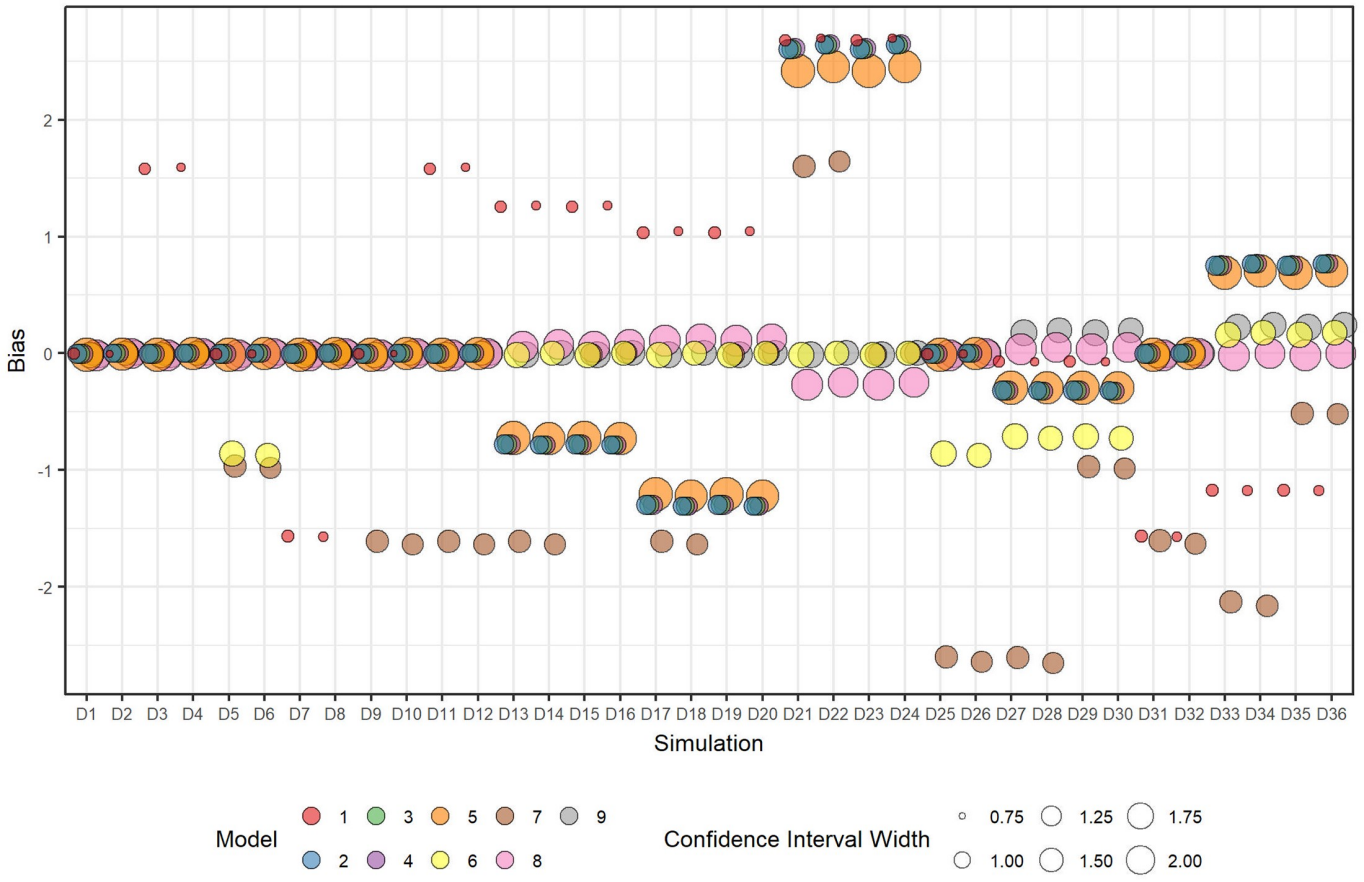
Plot of mean bias and mean 95% confidence interval width over all datasets within each scenario for each fitted model for the estimate of the time-averaged intervention effect.

Similar trends in bias and confidence interval widths were observed for the time-averaged intervention effect (Fig 7). A notable exception is the bias for Model 8, which had small, but non-zero, biases for scenarios D13 to D24, when exposure time was modelled as linear, whereas biases were still close to zero for Models 6 and 9. These non-zero biases were still smaller compared with Models 2 and 3, which attempt to estimate a single time-averaged intervention effect. Model 9 had small positive biases for scenarios D27 to D30 and D30 to D36 when the intervention effect was modelled as non-linear over time.

**Fig 8:**
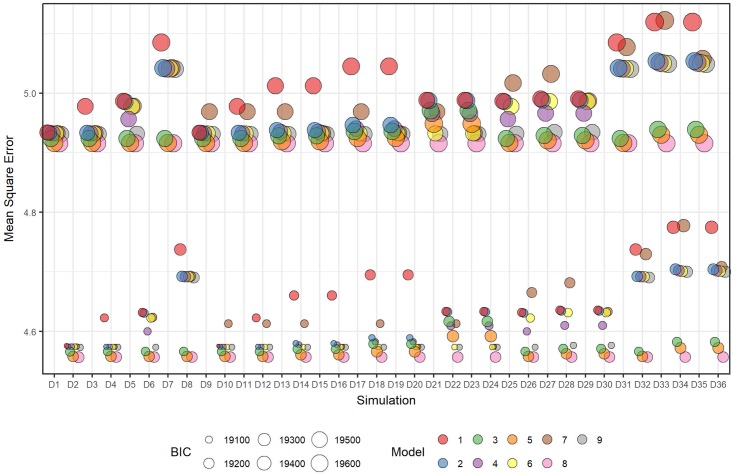
Plot of the mean of the MSE (mean square error) and mean BIC over all datasets within each scenario for each fitted model and where fitted models have assumed CS correlation structure.

When considering the overall fit of the model to the simulated data, across all models the MSEs were smaller for scenarios simulated with positive within-subject correlation compared with those with negative correlation (Fig 8). Model 8 consistently had the lowest mean MSEs across all scenarios (see Table I in S3 Appendix). Model 9 had similar MSEs, and smaller mean BICs, except for scenarios D31 to D36, which were simulated with a time effect described by a full sinusoid over the study period. Model 6 had larger MSEs compared with Model 9 across all scenarios, but smaller BIC values when time was linear and larger BIC values when time was sinusoidal. Compared to the variations in bias between fitted models, the variations in MSEs were smaller.

### Motivating example

The nine model formulations were fitted to the original OXTEXT-7 data. These results are presented in Table 4. Although not statistically significant, the intervention effects differ in sign and magnitude depending on the model selected to fit to the data.

**Table 4:** Estimates of the treatment effect after six months exposure time to the intervention for the original OXTEXT-7 SWCRT data.

Fitted Model	Intervention effect after six months exposure (SE)	p-value	Time-averaged intervention effect (SE)	p-value	BIC
*y*_*itk*_ = *β*_0_ + *∂x*_*tk*_ + *v*_0*ik*_ + *ϵ*_*itk*_ (Model 1)	0.33 (0.20)	0.096	0.33 (0.20)	0.096	30384.8
*y*_*ik*_(*t*) = *β*_0_ + *∂x*_*tk*_ + *τt* + *v*_0*ik*_ + *ϵ*_*ijk*_ (Model 2)	0.11 (0.33)	0.743	0.11 (0.33)	0.743	30376.5
*y*_*ik*_(*t*) = *β*_0_ + *∂x*_*tk*_ + *τt* + *ωx*_*tk*_*t* + *v*_0*ik*_ + *ϵ*_*itk*_ (Model 4)	0.22 (0.43)	0.611	0.19 (0.38)	0.625	30384.8
*y*_*ik*_(*t*) = *β*_0_ + *∂x*_*tk*_ + *τt* + *ψd*_*tk*_ + *v*_0*ik*_ + *ϵ*_*itk*_ (Model 6)	-0.08 (0.45)	0.855	-0.08 (0.45)	0.855	30384.6
*y*_*ik*_(*t*) = *β*_0_ + *τt* + *ψd*_*tk*_ + *v*_0*ik*_ + *ϵ*_*itk*_ (Model 7)	-0.19 (0.40)	0.632	-0.19 (0.40)	0.632	30376.4
*y*_*ik*_(*t*) = *β*_0_ + *∂x*_*tk*_ + *τt* + *ψd*_*tk*_ + *ζt*^2^ + *v*_0*ik*_ + *ϵ*_*itk*_ (Model 9)	0.09 (0.62)	0.147	0.09 (0.62)	0.147	30392.9
*y*_*itk*_ = *β*_0_ + *∂x*_*tk*_ + *κ*_*t*_ + *v*_0*ik*_ + *ϵ*_*itk*_ (Model 3) [8]	0.18 (0.37)	0.637	0.18 (0.37)	0.637	30483.7
*y*_*itk*_ = *β*_0_ + *∂x*_*tk*_ + *κ*_*t*_ + *φ*_*t*_*x*_*tk*_ + *v*_0*ik*_ + *ϵ*_*itk*_ (Model 5)	-0.28 (0.81)	0.731	0.62 (0.83)	0.454	30375.9
*y*_*itk*_ = *β*_0_ + *κ*_*t*_ + *ξ*_*d*_ + *v*_0*ik*_ + *ϵ*_*itk*_ (Model 8)	-0.59 (0.77)	0.446	0.24 (0.66)	0.717	30370.9

The model which performed the best in the simulation study (Model 8) provided a point estimate of the intervention effect at six months exposure of -0.59, with a large standard error of 0.77; almost four times the standard error of the simplest model. This is consistent with what was observed in the simulation study, where the standard error of the intervention effect depended primarily on the number of parameter estimates required. The BIC statistic for this model was the lowest, which is consistent with the model fits to the simulated data. The time-averaged treatment effect for Models 8 and 9 are similar to that obtained for Model 3, which is the analysis that had been specified in the protocol for this study.

## Discussion and conclusion

Our simulation study demonstrates that SWCRT scenarios exist such that when LMEs with simple formulations for time, which are typical for parallel cluster randomised controlled trials, are fitted to data, biased intervention effects with poor coverage of the true intervention effect result. Complex temporal trends in the outcome can arise due to factors outside of the trial, and for this reason the stepped wedge design should only be considered when the outcome is well understood and when the parallel CRT design is infeasible.

LMEs with complex terms for calendar and exposure time consistently obtained estimates that were less biased and had 95% confidence intervals with coverage close to 95%. The disadvantage is that where simpler formulations for time were sufficient, the confidence intervals for the treatment effect were wider.

Model 8 consistently had better coverage probabilities, low bias and better BIC statistics compared with other fitted models for all scenarios considered in this study. This model can be viewed as a modification of the Hussey and Hughes model [8] that allows the intervention effect to differ for each exposure time. The intervention effect at a specific amount of exposure time can easily be determined from the estimated parameters, as well as the time averaged intervention effect through the use of a linear combination of the parameter estimates. As avoiding biased estimates is a priority, we therefore recommend that in the absence of any information about the effect of time on the outcome, this model should be specified for the analysis of SWCRT data.

Model 9, which treated time as continuous with an additional term for quadratic time, obtained similar biases compared with Model 8, but tended to have coverage probabilities that were slightly further from the required 95% level. When non-linear effects of time were simulated, we considered a sine wave with a single peak during the study period and a sine wave with a peak and a trough. Model 9 with a quadratic term for time could approximate the scenarios with a single turning point, but performed less well when the effect over time had two turning points. The appropriateness for additional polynomial terms for time will be context-specific but should be considered if degrees of freedom are limited. We only considered simple quadratic function of time, but other polynomial functions for time could be fitted.

Our simulation study confirmed that a simple model which ignores the effect of time leads to confounding between the effect of time and the effect of the intervention and should therefore be avoided. Models treating time and the intervention as independent, such as the Hussey and Hughes model [8], consistently underestimated the effect of the intervention when the scenario had a simulated exposure time effect, even when the effect of interest was the time-averaged intervention effect.

Models including an interaction term between the intervention and calendar time misspecify the mean model for data under a SWCRT design and should also be avoided. Moreover, when calendar time is included as a categorical variable, the design matrix is rank-deficient, leading to some of the interaction terms being incomputable. Software such as SAS Proc Mixed will allow these models to be estimated and automatically discard redundant parameters, but when fitted with R, the user needs to carefully specify the interaction terms to be estimated to allow the model to converge. Although calendar time is a way of accounting for all known and unknown factors prevailing at the study centres, which may change during the study period, such as staffing levels or resource availability, modelling the intervention effect in such a way that it depends on these calendar time parameters limits its generalisability. Estimates related to calendar time should not be extrapolated beyond the trial.

Misspecification of the mean structure of the temporal effect had a much larger effect on the estimate of the intervention effect than did misspecification of the correlation structure. Statistical analysis plans should be flexible enough to allow for different formulations of time, which may be non-linear. Sensitivity analyses which allow the effect of time to be explored could be included in the statistical analysis plan, provided this is done in a way that avoids ‘cherry-picking’ the model that demonstrates the best intervention effect. Another way to proceed would be to first model data from the control condition only, so that the specification of a calendar time model can be obtained without knowledge of any intervention effects. A model with an appropriate parametric form could then be used in the full trial analysis. This approach would benefit from further research.

A limitation of our simulation study is that only a limited number of scenarios were considered. However, the scenarios are typical of what might be observed after an intervention is introduced to a new setting. We focused on linear terms for time, but more complex parameterisations of time could also be considered, such as the Ornstein–Uhlenbeck process for modelling the time effect [19]. This allows for correlated within-subject errors, allows the variance to change over time, and can be fitted to unbalanced datasets.

In this study we do not consider the effect of an imbalance in time-varying confounders between clusters randomised early to the intervention and those who start late. This could potentially lead to biased results, even though each cluster acts as its own control, and particularly when the number of clusters is small—a common issue in SWCRTs [20]. Research is in progress to inform the minimum number of clusters for SWCRTs [20].

Future work on the SWCRT study design should consider how to assess goodness-of-fit, particularly in relation to time effects. Sample size is another important consideration and should be large enough so that the model can untangle the effects of intervention and time [1, 21]. Not accounting for time effects when performing the sample size calculation will result in studies that are grossly underpowered [21–22]. This simulation study shows that statistical models alone cannot be used to determine intervention effects, as factors outside of the trial may lead to complex changes in the outcome over time, which may not always be resolved by the model. Rather these issues should be addressed in the design of the study as far as possible to ensure that a statistical model has the best chance of estimating the intervention effect of interest.

## Supporting information

S1 Appendix Simulation code. (PDF)

S2 Appendix Code to fit linear mixed effects models. (PDF)

S3 Appendix Model estimates for each scenario. (PDF)

S4 Appendix Additional plots. (PDF)

S1 Data OXTEXT-7 minimal dataset. (XLSX)

## References

[1] Hemming K, Haines TP, Chilton AJ, Lilford RJ. The stepped wedge cluster randomised trial: rationale, design, analysis, and report. BMJ. 2015;350: h391 10.1136/bmj.h391 25662947 10.1136/bmj.h391

[2] Beard E, Lewis JJ, Copas A, Davey C, Osrin D, Baio G, et al Stepped wedge randomised controlled trials: systematic review of studies published between 2010 and 2014. Trials. 2015;16: 353 10.1186/s13063-015-0839-2 26278881 10.1186/s13063-015-0839-2 PMC4538902

[3] Mdege ND, Man M, Taylor CA, Torgerson DJ. Systematic review of stepped wedge cluster randomized trials shows that design is particularly used to evaluate interventions during routine implementation. J Clin Epidemiol. 2011;64: 936–948. 10.1016/j.jclinepi.2010.12.003 21411284 10.1016/j.jclinepi.2010.12.003

[4] Barker D, McElduff P, D’Este C, Campbell MJ. Stepped wedge cluster randomised trials: a review of the statistical methodology used and available. BMC Med Res Methodol. 2016;16: 69 10.1186/s12874-016-0176-5 27267471 10.1186/s12874-016-0176-5 PMC4895892

[5] De Allegri M, Pokhrel S, Becher H, Dong H, Mansmann U, Kouyaté B, et al Step-wedge cluster-randomised community-based trials: An application to the study of the impact of community health insurance. Health Res Policy Syst. 2008;6: 10 10.1186/1478-4505-6-10 18945332 10.1186/1478-4505-6-10 PMC2583992

[6] Fuller C, Michie S, Savage J, McAteer J, Besser S, Charlett A, et al The Feedback Intervention Trial (FIT)—Improving hand-hygiene compliance in UK healthcare workers: A stepped wedge cluster randomised controlled trial. PLoS One. 2012;7(10): e41617 10.1371/journal.pone.0041617 BMJ. 2017; 23110040 10.1371/journal.pone.0041617 PMC3479093

[7] Hemming K, Eldridge S, Forbes G, Weijer C, Taljaard M. How to design efficient cluster randomised trials. 2017;358:j3064 10.1136/bmj.j3064 28710062 10.1136/bmj.j3064 PMC5508848

[8] Hussey MA, Hughes JP. Design and analysis of stepped wedge cluster randomized trials. Contemp Clin Trials. 2007;28: 182–91. 10.1016/j.cct.2006.05.007 16829207 10.1016/j.cct.2006.05.007

[9] Brown CA, Lilford RJ. The stepped wedge trial design: a systematic review. BMC Med Res Methodol. 2006;6: 65 10.1186/1471-2288-6-54 17092344 10.1186/1471-2288-6-54 PMC1636652

[10] Hedeker D, Gibbons RD. Longitudinal Data Analysis. John Wiley & Sons, New Jersey; 2006.

[11] Bilderbeck A, Price J, Hinds C, Voysey M, Nickless A, Geddes J, et al. OXTEXT: The development and evaluation of a remote monitoring and management service for people with bipolar disorder and other psychiatric disorders. NIHR Report for Programme Grants for Applied Research Programme (Reference Number RP-PG-0108-10087); 2015.

[12] Wing J, Curtis RH, Beevor A. Health of the Nation Outcome Scales (HoNOS). Glossary for HoNOS score sheet. Br J Psychiatry. 1999;174 (5): 432–434. 10.1192/bjp.174.5.432 10616611 10.1192/bjp.174.5.432

[13] Twomey C, Prina AM, Baldwin DS, Das-Munshi J, Kingdon D, Koeser L, et al Utility of the Health of the Nation Outcome Scales (HoNOS) in predicting mental health service costs for patients with common mental health problems: Historical cohort study. PLoS One. 2016;11(11): e0167103 10.1371/journal.pone.0167103 27902745 10.1371/journal.pone.0167103 PMC5130232

[14] Audin K, Margison FR, Clark JM, Barkham M. Value of HoNOS in assessing patient change in NHS psychotherapy and psychological treatment services. Br J Psychiatry. 2001;178, 561–566. 11388975 10.1192/bjp.178.6.561

[15] Diggle PJ, Heagerty PJ, Liang K, Zeger SL. Analysis of Longitudinal Data. 2nd Ed Oxford University Press, Oxford, UK; 2002.

[16] Fok CCT, Henry D, Allen J. Research designs for intervention research with small samples II: stepped wedge and interrupted time-series designs. Prev Sci. 2015;16: 967–977. 10.1007/s11121-015-0569-4 26017633 10.1007/s11121-015-0569-4 PMC4581909

[17] Song P, Xue J, Li Z. Simulation of longitudinal exposure data with variance-covariance structures based on mixed models. Risk Anal. 2013;33: 469–479. 10.1111/j.1539-6924.2012.01869.x 22817762 10.1111/j.1539-6924.2012.01869.x PMC3689546

[18] Hemming K, Girling A. A menu-driven facility for power and detectable-difference calculations in stepped-wedge cluster-randomized trials. The STATA J. 2014;14(2): 363–380.

[19] Hughes RA, Kenward MG, Sterne JAC, Tilling K. Estimation of the linear mixed integrated Ornstein–Uhlenbeck model. J Stat Comput Simul. 2017;87(8): 1541–1558. 10.1080/00949655.2016.1277425 28515536 10.1080/00949655.2016.1277425 PMC5407356

[20] Taljaard M, Teerenstra S, Ivers NM, Fergusson DA. Substantial risks associated with few clusters in cluster randomized and stepped wedge designs. Clinical Trials. 2016;13(4): 459–463. 10.1177/1740774516634316 26940696 10.1177/1740774516634316

[21] Hooper R, Teerenstra S, de Hoop E, Eldridge S. Sample size calculation for stepped wedge and other longitudinal cluster randomised trials. Stat Med. 2016;35(26): 4718–4728. 10.1002/sim.7028 27350420 10.1002/sim.7028

[22] Baio G, Copas A, Ambler G, Hargreaves J, Beard E, Omar RZ. Sample size calculation for a stepped wedge trial. Trials. 2015;16: 354 10.1186/s13063-015-0840-9 26282553 10.1186/s13063-015-0840-9 PMC4538764

