# Supplementary material for: Mixed effects approach to the analysis of the stepped wedge cluster randomised trial—Investigating the confounding effect of time through simulation
Source: PLoS One. 2018 Dec 13;13(12):e0208876. doi: 10.1371/journal.pone.0208876 (PMC6292598; doi:10.1371/journal.pone.0208876)
Supplement: S1 Appendix — (PDF) [file pone.0208876.s001.pdf]

S1 Appendix. Simulation Code

```
##Alecia Nickless
#28 September 2018
#Code to simulate datasets for HoNOS outcome
#Following procedure outlined in Song et al (2013)

#NULL models
##Simulation D1
nSim <- 1000
file_loc <- "C:/Users/aleci_000/Documents/Oxford Documents/Stepped Wedge Paper/Sim_Datasets/"

Sim0 <- function(nSim) {
for (N in 1:nSim){
sigmab = 4.415
sigmae = 5.442
sigmac = 0.9570
rho = -0.5

NumCentre = 12
NSteps = NumCentre + 1
AverageClusterSize = 20


centres = seq(1,NumCentre)
random_centres = sample(centres, NumCentre, replace = FALSE)

Beta0 <- 14.00 #Mean
Beta1 <- 0 #Time
Beta2 <- 0 #Intervention
Beta3 <- 0 #Intervention specific Time

Patient_ID = NULL
Time = NULL
TimeInt = NULL
Intervention = NULL
HONOS_scores = NULL
Cluster_ID = NULL
Random_Error = NULL
Random_Effect = NULL
for (i in 1:NumCentre) {
  random_effect_clust <- rnorm(1,0,sigmac)
```

```
  for (k in 1:AverageClusterSize) {
    random_effect = rnorm(1,0,sigmab)
    for (j in 1:NSteps) {
      randomstart = match(i, random_centres) + 1
      patientnumber = (i - 1)*20 + k
      Patient_ID = c(Patient_ID, paste("Patient_",patientnumber, sep=""))
      Cluster_ID = c(Cluster_ID, paste("Cluster_",i, sep=""))
      time = j
      Time = c(Time, j)
      random_error = rnorm(1,0,sqrt((1-rho^2)*sigmae^2))
      if (j==1) {
        random_error_corr = random_error
      }
      if (j > 1) {
        random_error_corr = rho*Random_Error[(patientnumber-1)*NSteps + 1] + random_error
      }
      Random_Error = c(Random_Error, random_error_corr)
      Random_Effect = c(Random_Effect, random_effect)
      if (j < randomstart) {
        timeint = 0
        intervention = 0
      }
      else {
        timeint = j - randomstart + 1
        intervention = 1}
      TimeInt = c(TimeInt, timeint)
      Intervention = c(Intervention, intervention)
      HONOS = Beta0 + Beta1*time + Beta2*intervention + Beta3*timeint + random_effect + random_effect_clust +
random_error_corr
      HONOS_scores <- c(HONOS_scores, HONOS)
    }
  }
}



dataset <- cbind(Patient_ID, Cluster_ID, Intervention, Time, TimeInt, HONOS_scores, Random_Effect, Random_Error)
dataset <- as.data.frame(dataset)
write.csv(dataset, file = paste(file_loc,"Sim0_",N,".csv", sep =""))
}
}
set.seed(12345)
Sim0(nSim)
```

```
##Simulation D2
Sim0000 <- function(nSim) {
  for (N in 1:nSim){
    sigmab = 4.415
    sigmae = 5.442
    sigmac = 0.9570
    rho = 0.5

    NumCentre = 12
    NSteps = NumCentre + 1
    AverageClusterSize = 20


    centres = seq(1,NumCentre)
    random_centres = sample(centres, NumCentre, replace = FALSE)

    Beta0 <- 14.00 #Mean
    Beta1 <- 0 #Time
    Beta2 <- 0 #Intervention
    Beta3 <- 0 #Intervention specific Time

    Patient_ID = NULL
    Time = NULL
    TimeInt = NULL
    Intervention = NULL
    HONOS_scores = NULL
    Cluster_ID = NULL
    Random_Error = NULL
    Random_Effect = NULL
    for (i in 1:NumCentre) {
      random_effect_clust <- rnorm(1,0,sigmac)
      for (k in 1:AverageClusterSize) {
        random_effect = rnorm(1,0,sigmab)
        for (j in 1:NSteps) {
          randomstart = match(i, random_centres) + 1
          patientnumber = (i - 1)*20 + k
          Patient_ID = c(Patient_ID, paste("Patient_",patientnumber, sep=""))
          Cluster_ID = c(Cluster_ID, paste("Cluster_",i, sep=""))
          time = j
          Time = c(Time, j)
          random_error = rnorm(1,0,sqrt((1-rho^2)*sigmae^2))
          if (j==1) {
```

```
            random_error_corr = random_error
          }
          if (j > 1) {
            random_error_corr = rho*Random_Error[(patientnumber-1)*NSteps + 1] + random_error
          }
          Random_Error = c(Random_Error, random_error_corr)
          Random_Effect = c(Random_Effect, random_effect)
          if (j < randomstart) {
            timeint = 0
            intervention = 0
          }
          else {
            timeint = j - randomstart + 1
            intervention = 1}
          TimeInt = c(TimeInt, timeint)
          Intervention = c(Intervention, intervention)
          HONOS = Beta0 + Beta1*time + Beta2*intervention + Beta3*timeint + random_effect + random_effect_clust +
random_error_corr
          HONOS_scores <- c(HONOS_scores, HONOS)
        }
      }
    }



    dataset <- cbind(Patient_ID, Cluster_ID, Intervention, Time, TimeInt, HONOS_scores, Random_Effect, Random_Error)
    dataset <- as.data.frame(dataset)
    write.csv(dataset, file = paste(file_loc,"Sim0000_",N,".csv", sep =""))
  }
}
set.seed(12345)
Sim0000(nSim)


##Simulation D3
Sim00 <- function(nSim) {
  for (N in 1:nSim){

sigmab = 4.415
sigmae = 5.442
sigmac = 0.9570
rho = -0.5
```

```
NumCentre = 12
NSteps = NumCentre + 1
AverageClusterSize = 20


centres = seq(1,NumCentre)
random_centres = sample(centres, NumCentre, replace = FALSE)

Beta0 <- 14.00 #Mean
Beta1 <- 0.25 #Time
Beta2 <- 0 #Intervention
Beta3 <- 0 #Intervention specific Time

Patient_ID = NULL
Time = NULL
TimeInt = NULL
Intervention = NULL
HONOS_scores = NULL
Cluster_ID = NULL
Random_Error = NULL
Random_Effect = NULL
for (i in 1:NumCentre) {
  random_effect_clust <- rnorm(1,0,sigmac)
  for (k in 1:AverageClusterSize) {
    random_effect = rnorm(1,0,sigmab)
    for (j in 1:NSteps) {
      randomstart = match(i, random_centres) + 1
      patientnumber = (i - 1)*20 + k
      Patient_ID = c(Patient_ID, paste("Patient_",patientnumber, sep=""))
      Cluster_ID = c(Cluster_ID, paste("Cluster_",i, sep=""))
      time = j
      Time = c(Time, j)
      random_error = rnorm(1,0,sqrt((1-rho^2)*sigmae^2))
      if (j==1) {
        random_error_corr = random_error
      }
      if (j > 1) {
        random_error_corr = rho*Random_Error[(patientnumber-1)*NSteps + 1] + random_error
      }
      Random_Error = c(Random_Error, random_error_corr)
      Random_Effect = c(Random_Effect, random_effect)
      if (j < randomstart) {
        timeint = 0
```

```
        intervention = 0
      }
      else {
        timeint = j - randomstart + 1
        intervention = 1}
      TimeInt = c(TimeInt, timeint)
      Intervention = c(Intervention, intervention)
      HONOS = Beta0 + Beta1*time + Beta2*intervention + Beta3*timeint + random_effect + random_error_corr
      HONOS_scores <- c(HONOS_scores, HONOS)
    }
  }
}



dataset <- cbind(Patient_ID, Cluster_ID, Intervention, Time, TimeInt, HONOS_scores, Random_Effect, Random_Error)
dataset <- as.data.frame(dataset)
write.csv(dataset, file = paste(file_loc,"Sim00_",N,".csv", sep =""))
  }
}
set.seed(12345)
Sim00(nSim)


##Simulation D4
Sim00000 <- function(nSim) {
  for (N in 1:nSim){

    sigmab = 4.415
    sigmae = 5.442
    sigmac = 0.9570
    rho = 0.5

    NumCentre = 12
    NSteps = NumCentre + 1
    AverageClusterSize = 20


    centres = seq(1,NumCentre)
    random_centres = sample(centres, NumCentre, replace = FALSE)

    Beta0 <- 14.00 #Mean
    Beta1 <- 0.25 #Time
```

```
Beta2 <- 0 #Intervention
Beta3 <- 0 #Intervention specific Time

Patient_ID = NULL
Time = NULL
TimeInt = NULL
Intervention = NULL
HONOS_scores = NULL
Cluster_ID = NULL
Random_Error = NULL
Random_Effect = NULL
for (i in 1:NumCentre) {
  random_effect_clust <- rnorm(1,0,sigmac)
  for (k in 1:AverageClusterSize) {
    random_effect = rnorm(1,0,sigmab)
    for (j in 1:NSteps) {
      randomstart = match(i, random_centres) + 1
      patientnumber = (i - 1)*20 + k
      Patient_ID = c(Patient_ID, paste("Patient_",patientnumber, sep=""))
      Cluster_ID = c(Cluster_ID, paste("Cluster_",i, sep=""))
      time = j
      Time = c(Time, j)
      random_error = rnorm(1,0,sqrt((1-rho^2)*sigmae^2))
      if (j==1) {
        random_error_corr = random_error
      }
      if (j > 1) {
        random_error_corr = rho*Random_Error[(patientnumber-1)*NSteps + 1] + random_error
      }
      Random_Error = c(Random_Error, random_error_corr)
      Random_Effect = c(Random_Effect, random_effect)
      if (j < randomstart) {
        timeint = 0
        intervention = 0
      }
      else {
        timeint = j - randomstart + 1
        intervention = 1}
      TimeInt = c(TimeInt, timeint)
      Intervention = c(Intervention, intervention)
      HONOS = Beta0 + Beta1*time + Beta2*intervention + Beta3*timeint + random_effect + random_error_corr
      HONOS_scores <- c(HONOS_scores, HONOS)
    }
```

```
      }
    }



    dataset <- cbind(Patient_ID, Cluster_ID, Intervention, Time, TimeInt, HONOS_scores, Random_Effect, Random_Error)
    dataset <- as.data.frame(dataset)
    write.csv(dataset, file = paste(file_loc,"Sim00000_",N,".csv", sep =""))
  }
}
set.seed(12345)
Sim00000(nSim)


##Simulation D5
Sim000 <- function(nSim) {
  for (N in 1:nSim){
sigmab = 4.415
sigmae = 5.442
sigmac = 0.9570
rho = -0.5

NumCentre = 12
NSteps = NumCentre + 1
AverageClusterSize = 20


centres = seq(1,NumCentre)
random_centres = sample(centres, NumCentre, replace = FALSE)

Beta0 <- 14.00 #Mean
Beta1 <- 1 #Time
Beta2 <- 0 #Intervention
Beta3 <- 0 #Intervention specific Time

x <- seq(0,12)
y <- sin(x*180/12*pi/180)
Beta1_time <- y*2*Beta1
Beta3_time <- y*1*Beta3
Time_factor <- seq(1,13)
Time_factor <- factor(Time_factor)

Patient_ID = NULL
```

```
Time = NULL
TimeInt = NULL
Intervention = NULL
HONOS_scores = NULL
Cluster_ID = NULL
Random_Error = NULL
Random_Effect = NULL
for (i in 1:NumCentre) {
  random_effect_clust <- rnorm(1,0,sigmac)
  for (k in 1:AverageClusterSize) {
    random_effect = rnorm(1,0,sigmab)
    for (j in 1:NSteps) {
      randomstart = match(i, random_centres) + 1
      patientnumber = (i - 1)*20 + k
      Patient_ID = c(Patient_ID, paste("Patient_",patientnumber, sep=""))
      Cluster_ID = c(Cluster_ID, paste("Cluster_",i, sep=""))
      time = j
      Time = c(Time, j)
      random_error = rnorm(1,0,sqrt((1-rho^2)*sigmae^2))
      if (j==1) {
        random_error_corr = random_error
      }
      if (j > 1) {
        random_error_corr = rho*Random_Error[(patientnumber-1)*NSteps + 1] + random_error
      }
      Random_Error = c(Random_Error, random_error_corr)
      Random_Effect = c(Random_Effect, random_effect)
      if (j < randomstart) {
        timeint = 0
        intervention = 0
      }
      else {
        timeint = j - randomstart + 1
        intervention = 1}
      TimeInt = c(TimeInt, timeint)
      Intervention = c(Intervention, intervention)
      HONOS = Beta0 + Beta1_time[match(factor(time),Time_factor)] + Beta2*intervention +
Beta3_time[match(factor(time),Time_factor)] + random_effect + random_error_corr
      HONOS_scores <- c(HONOS_scores, HONOS)
    }
  }
}
```

```
dataset <- cbind(Patient_ID, Cluster_ID, Intervention, Time, TimeInt, HONOS_scores, Random_Effect, Random_Error)
dataset <- as.data.frame(dataset)
write.csv(dataset, file = paste(file_loc,"Sim000_",N,".csv", sep =""))
  }
}
set.seed(12345)
Sim000(nSim)


##Simulation D6
Sim000000 <- function(nSim) {
  for (N in 1:nSim){
    sigmab = 4.415
    sigmae = 5.442
    sigmac = 0.9570
    rho = 0.5

    NumCentre = 12
    NSteps = NumCentre + 1
    AverageClusterSize = 20


    centres = seq(1,NumCentre)
    random_centres = sample(centres, NumCentre, replace = FALSE)

    Beta0 <- 14.00 #Mean
    Beta1 <- 1 #Time
    Beta2 <- 0 #Intervention
    Beta3 <- 0 #Intervention specific Time

    x <- seq(0,12)
    y <- sin(x*180/12*pi/180)
    Beta1_time <- y*2*Beta1
    Beta3_time <- y*1*Beta3
    Time_factor <- seq(1,13)
    Time_factor <- factor(Time_factor)

    Patient_ID = NULL
    Time = NULL
    TimeInt = NULL
    Intervention = NULL
```

```
HONOS_scores = NULL
Cluster_ID = NULL
Random_Error = NULL
Random_Effect = NULL
for (i in 1:NumCentre) {
  random_effect_clust <- rnorm(1,0,sigmac)
  for (k in 1:AverageClusterSize) {
    random_effect = rnorm(1,0,sigmab)
    for (j in 1:NSteps) {
      randomstart = match(i, random_centres) + 1
      patientnumber = (i - 1)*20 + k
      Patient_ID = c(Patient_ID, paste("Patient_",patientnumber, sep=""))
      Cluster_ID = c(Cluster_ID, paste("Cluster_",i, sep=""))
      time = j
      Time = c(Time, j)
      random_error = rnorm(1,0,sqrt((1-rho^2)*sigmae^2))
      if (j==1) {
        random_error_corr = random_error
      }
      if (j > 1) {
        random_error_corr = rho*Random_Error[(patientnumber-1)*NSteps + 1] + random_error
      }
      Random_Error = c(Random_Error, random_error_corr)
      Random_Effect = c(Random_Effect, random_effect)
      if (j < randomstart) {
        timeint = 0
        intervention = 0
      }
      else {
        timeint = j - randomstart + 1
        intervention = 1}
      TimeInt = c(TimeInt, timeint)
      Intervention = c(Intervention, intervention)
      HONOS = Beta0 + Beta1_time[match(factor(time),Time_factor)] + Beta2*intervention +
Beta3_time[match(factor(time),Time_factor)] + random_effect + random_error_corr
      HONOS_scores <- c(HONOS_scores, HONOS)
    }
  }
}



dataset <- cbind(Patient_ID, Cluster_ID, Intervention, Time, TimeInt, HONOS_scores, Random_Effect, Random_Error)
```

```
    dataset <- as.data.frame(dataset)
    write.csv(dataset, file = paste(file_loc,"Sim000000_",N,".csv", sep =""))
  }
}
set.seed(12345)
Sim000000(nSim)



##Simulation D7
Sim0000000 <- function(nSim) {
  for (N in 1:nSim){
    sigmab = 4.415
    sigmae = 5.442
    sigmac = 0.9570
    rho = -0.5

    NumCentre = 12
    NSteps = NumCentre + 1
    AverageClusterSize = 20


    centres = seq(1,NumCentre)
    random_centres = sample(centres, NumCentre, replace = FALSE)

    Beta0 <- 14.00 #Mean
    Beta1 <- 1 #Time
    Beta2 <- 0 #Intervention
    Beta3 <- 0 #Intervention specific Time

    x <- seq(0,12)
    y <- sin(x*180/6*pi/180)
    Beta1_time <- y*2*Beta1
    Beta3_time <- y*1*Beta3
    Time_factor <- seq(1,13)
    Time_factor <- factor(Time_factor)

    Patient_ID = NULL
    Time = NULL
    TimeInt = NULL
    Intervention = NULL
    HONOS_scores = NULL
    Cluster_ID = NULL
```

```
    Random_Error = NULL
    Random_Effect = NULL
    for (i in 1:NumCentre) {
      random_effect_clust <- rnorm(1,0,sigmac)
      for (k in 1:AverageClusterSize) {
        random_effect = rnorm(1,0,sigmab)
        for (j in 1:NSteps) {
          randomstart = match(i, random_centres) + 1
          patientnumber = (i - 1)*20 + k
          Patient_ID = c(Patient_ID, paste("Patient_",patientnumber, sep=""))
          Cluster_ID = c(Cluster_ID, paste("Cluster_",i, sep=""))
          time = j
          Time = c(Time, j)
          random_error = rnorm(1,0,sqrt((1-rho^2)*sigmae^2))
          if (j==1) {
            random_error_corr = random_error
          }
          if (j > 1) {
            random_error_corr = rho*Random_Error[(patientnumber-1)*NSteps + 1] + random_error
          }
          Random_Error = c(Random_Error, random_error_corr)
          Random_Effect = c(Random_Effect, random_effect)
          if (j < randomstart) {
            timeint = 0
            intervention = 0
          }
          else {
            timeint = j - randomstart + 1
            intervention = 1}
          TimeInt = c(TimeInt, timeint)
          Intervention = c(Intervention, intervention)
          HONOS = Beta0 + Beta1_time[match(factor(time),Time_factor)] + Beta2*intervention + 
Beta3_time[match(factor(time),Time_factor)] + random_effect + random_error_corr
          HONOS_scores <- c(HONOS_scores, HONOS)
        }
      }
    }



    dataset <- cbind(Patient_ID, Cluster_ID, Intervention, Time, TimeInt, HONOS_scores, Random_Effect, Random_Error)
    dataset <- as.data.frame(dataset)
    write.csv(dataset, file = paste(file_loc,"Sim0000000_",N,".csv", sep =""))
```

```
  }
}
set.seed(12345)
Sim0000000(nSim)


##Simulation D8
Sim00000000 <- function(nSim) {
  for (N in 1:nSim){
    sigmab = 4.415
    sigmae = 5.442
    sigmac = 0.9570
    rho = 0.5

    NumCentre = 12
    NSteps = NumCentre + 1
    AverageClusterSize = 20


    centres = seq(1,NumCentre)
    random_centres = sample(centres, NumCentre, replace = FALSE)

    Beta0 <- 14.00 #Mean
    Beta1 <- 1 #Time
    Beta2 <- 0 #Intervention
    Beta3 <- 0 #Intervention specific Time

    x <- seq(0,12)
    y <- sin(x*180/6*pi/180)
    Beta1_time <- y*2*Beta1
    Beta3_time <- y*1*Beta3
    Time_factor <- seq(1,13)
    Time_factor <- factor(Time_factor)

    Patient_ID = NULL
    Time = NULL
    TimeInt = NULL
    Intervention = NULL
    HONOS_scores = NULL
    Cluster_ID = NULL
    Random_Error = NULL
    Random_Effect = NULL
    for (i in 1:NumCentre) {
```

```
      random_effect_clust <- rnorm(1,0,sigmac)
      for (k in 1:AverageClusterSize) {
        random_effect = rnorm(1,0,sigmab)
        for (j in 1:NSteps) {
          randomstart = match(i, random_centres) + 1
          patientnumber = (i - 1)*20 + k
          Patient_ID = c(Patient_ID, paste("Patient_",patientnumber, sep=""))
          Cluster_ID = c(Cluster_ID, paste("Cluster_",i, sep=""))
          time = j
          Time = c(Time, j)
          random_error = rnorm(1,0,sqrt((1-rho^2)*sigmae^2))
          if (j==1) {
            random_error_corr = random_error
          }
          if (j > 1) {
            random_error_corr = rho*Random_Error[(patientnumber-1)*NSteps + 1] + random_error
          }
          Random_Error = c(Random_Error, random_error_corr)
          Random_Effect = c(Random_Effect, random_effect)
          if (j < randomstart) {
            timeint = 0
            intervention = 0
          }
          else {
            timeint = j - randomstart + 1
            intervention = 1}
          TimeInt = c(TimeInt, timeint)
          Intervention = c(Intervention, intervention)
          HONOS = Beta0 + Beta1_time[match(factor(time),Time_factor)] + Beta2*intervention +
Beta3_time[match(factor(time),Time_factor)] + random_effect + random_error_corr
          HONOS_scores <- c(HONOS_scores, HONOS)
        }
      }
    }



    dataset <- cbind(Patient_ID, Cluster_ID, Intervention, Time, TimeInt, HONOS_scores, Random_Effect, Random_Error)
    dataset <- as.data.frame(dataset)
    write.csv(dataset, file = paste(file_loc,"Sim00000000_",N,".csv", sep =""))
  }
}
set.seed(12345)
```

```
Sim00000000(nSim)


#Intervention Models
##Simulation D9
Sim1 <- function(nSim) {
  for (N in 1:nSim){
    sigmab = 4.415
    sigmae = 5.442
    sigmac = 0.9570
    rho = -0.5

NumCentre = 12
NSteps = NumCentre + 1
AverageClusterSize = 20


centres = seq(1,NumCentre)
random_centres = sample(centres, NumCentre, replace = FALSE)

Beta0 <- 14.00 #Mean
Beta1 <- 0 #Time
Beta2 <- 2 #Intervention
Beta3 <- 0 #Intervention specific Time

Patient_ID = NULL
Time = NULL
TimeInt = NULL
Intervention = NULL
HONOS_scores = NULL
Cluster_ID = NULL
Random_Error = NULL
Random_Effect = NULL
for (i in 1:NumCentre) {
  random_effect_clust <- rnorm(1,0,sigmac)
  for (k in 1:AverageClusterSize) {
    random_effect = rnorm(1,0,sigmab)
    for (j in 1:NSteps) {
      randomstart = match(i, random_centres) + 1
      patientnumber = (i - 1)*20 + k
      Patient_ID = c(Patient_ID, paste("Patient_",patientnumber, sep=""))
      Cluster_ID = c(Cluster_ID, paste("Cluster_",i, sep=""))
      time = j
```

```
      Time = c(Time, j)
      random_error = rnorm(1,0,sqrt((1-rho^2)*sigmae^2))
      if (j==1) {
        random_error_corr = random_error
      }
      if (j > 1) {
        random_error_corr = rho*Random_Error[(patientnumber-1)*NSteps + 1] + random_error
      }
      Random_Error = c(Random_Error, random_error_corr)
      Random_Effect = c(Random_Effect, random_effect)
      if (j < randomstart) {
        timeint = 0
        intervention = 0
      }
      else {
        timeint = j - randomstart + 1
        intervention = 1}
      TimeInt = c(TimeInt, timeint)
      Intervention = c(Intervention, intervention)
      HONOS = Beta0 + Beta1*time + Beta2*intervention + Beta3*timeint + random_effect + random_error_corr
      HONOS_scores <- c(HONOS_scores, HONOS)
    }
  }
}


dataset <- cbind(Patient_ID, Cluster_ID, Intervention, Time, TimeInt, HONOS_scores, Random_Effect, Random_Error)
dataset <- as.data.frame(dataset)
write.csv(dataset, file = paste(file_loc,"Sim1_",N,".csv", sep =""))
  }
}
set.seed(12345)
Sim1(nSim)


##Simulation D10
Sim5 <- function(nSim) {
  for (N in 1:nSim){
    sigmab = 4.415
    sigmae = 5.442
    sigmac = 0.9570
    rho = 0.5
```

```
NumCentre = 12
NSteps = NumCentre + 1
AverageClusterSize = 20


centres = seq(1,NumCentre)
random_centres = sample(centres, NumCentre, replace = FALSE)

Beta0 <- 14.00 #Mean
Beta1 <- 0 #Time
Beta2 <- 2 #Intervention
Beta3 <- 0 #Intervention specific Time

Patient_ID = NULL
Time = NULL
TimeInt = NULL
Intervention = NULL
HONOS_scores = NULL
Cluster_ID = NULL
Random_Error = NULL
Random_Effect = NULL
for (i in 1:NumCentre) {
  random_effect_clust <- rnorm(1,0,sigmac)
  for (k in 1:AverageClusterSize) {
    random_effect = rnorm(1,0,sigmab)
    for (j in 1:NSteps) {
      randomstart = match(i, random_centres) + 1
      patientnumber = (i - 1)*20 + k
      Patient_ID = c(Patient_ID, paste("Patient_",patientnumber, sep=""))
      Cluster_ID = c(Cluster_ID, paste("Cluster_",i, sep=""))
      time = j
      Time = c(Time, j)
      random_error = rnorm(1,0,sqrt((1-rho^2)*sigmae^2))
      if (j==1) {
        random_error_corr = random_error
      }
      if (j > 1) {
        random_error_corr = rho*Random_Error[(patientnumber-1)*NSteps + 1] + random_error
      }
      Random_Error = c(Random_Error, random_error_corr)
      Random_Effect = c(Random_Effect, random_effect)
      if (j < randomstart) {
        timeint = 0
```

```
          intervention = 0
        }
        else {
          timeint = j - randomstart + 1
          intervention = 1}
        TimeInt = c(TimeInt, timeint)
        Intervention = c(Intervention, intervention)
        HONOS = Beta0 + Beta1*time + Beta2*intervention + Beta3*timeint + random_effect + random_error_corr
        HONOS_scores <- c(HONOS_scores, HONOS)
      }
    }
  }


  dataset <- cbind(Patient_ID, Cluster_ID, Intervention, Time, TimeInt, HONOS_scores, Random_Effect, Random_Error)
  dataset <- as.data.frame(dataset)
  write.csv(dataset, file = paste(file_loc,"Sim5_",N,".csv", sep =""))
 }
}
set.seed(12345)
Sim5(nSim)


##Simulation D11
Sim2 <- function(nSim) {
  for (N in 1:nSim){
    sigmab = 4.415
    sigmae = 5.442
    sigmac = 0.9570
    rho = -0.5

NumCentre = 12
NSteps = NumCentre + 1
AverageClusterSize = 20

centres = seq(1,NumCentre)
random_centres = sample(centres, NumCentre, replace = FALSE)

Beta0 <- 14.00 #Mean
Beta1 <- 0.25 #Time
Beta2 <- 2 #Intervention
Beta3 <- 0 #Intervention specific Time
```

```
Patient_ID = NULL
Time = NULL
TimeInt = NULL
Intervention = NULL
HONOS_scores = NULL
Cluster_ID = NULL
Random_Error = NULL
Random_Effect = NULL
for (i in 1:NumCentre) {
  random_effect_clust <- rnorm(1,0,sigmac)
  for (k in 1:AverageClusterSize) {
    random_effect = rnorm(1,0,sigmab)
    for (j in 1:NSteps) {
      randomstart = match(i, random_centres) + 1
      patientnumber = (i - 1)*20 + k
      Patient_ID = c(Patient_ID, paste("Patient_",patientnumber, sep=""))
      Cluster_ID = c(Cluster_ID, paste("Cluster_",i, sep=""))
      time = j
      Time = c(Time, j)
      random_error = rnorm(1,0,sqrt((1-rho^2)*sigmae^2))
      if (j==1) {
        random_error_corr = random_error
      }
      if (j > 1) {
        random_error_corr = rho*Random_Error[(patientnumber-1)*NSteps + 1] + random_error
      }
      Random_Error = c(Random_Error, random_error_corr)
      Random_Effect = c(Random_Effect, random_effect)
      if (j < randomstart) {
        timeint = 0
        intervention = 0
      }
      else {
        timeint = j - randomstart + 1
        intervention = 1}
      TimeInt = c(TimeInt, timeint)
      Intervention = c(Intervention, intervention)
      HONOS = Beta0 + Beta1*time + Beta2*intervention + Beta3*timeint + random_effect + random_error_corr
      HONOS_scores <- c(HONOS_scores, HONOS)
    }
  }
}
```

```
dataset <- cbind(Patient_ID, Cluster_ID, Intervention, Time, TimeInt, HONOS_scores, Random_Effect, Random_Error)
dataset <- as.data.frame(dataset)
write.csv(dataset, file = paste(file_loc,"Sim2_",N,".csv", sep =""))
  }
}
set.seed(12345)
Sim2(nSim)


##Simulation D12
Sim6 <- function(nSim) {
  for (N in 1:nSim){
    sigmab = 4.415
    sigmae = 5.442
    sigmac = 0.9570
    rho = 0.5

    NumCentre = 12
    NSteps = NumCentre + 1
    AverageClusterSize = 20

    centres = seq(1,NumCentre)
    random_centres = sample(centres, NumCentre, replace = FALSE)

    Beta0 <- 14.00 #Mean
    Beta1 <- 0.25 #Time
    Beta2 <- 2 #Intervention
    Beta3 <- 0 #Intervention specific Time

    Patient_ID = NULL
    Time = NULL
    TimeInt = NULL
    Intervention = NULL
    HONOS_scores = NULL
    Cluster_ID = NULL
    Random_Error = NULL
    Random_Effect = NULL
    for (i in 1:NumCentre) {
      random_effect_clust <- rnorm(1,0,sigmac)
      for (k in 1:AverageClusterSize) {
        random_effect = rnorm(1,0,sigmab)
        for (j in 1:NSteps) {
```

```
          randomstart = match(i, random_centres) + 1
          patientnumber = (i - 1)*20 + k
          Patient_ID = c(Patient_ID, paste("Patient_",patientnumber, sep=""))
          Cluster_ID = c(Cluster_ID, paste("Cluster_",i, sep=""))
          time = j
          Time = c(Time, j)
          random_error = rnorm(1,0,sqrt((1-rho^2)*sigmae^2))
          if (j==1) {
            random_error_corr = random_error
          }
          if (j > 1) {
            random_error_corr = rho*Random_Error[(patientnumber-1)*NSteps + 1] + random_error
          }
          Random_Error = c(Random_Error, random_error_corr)
          Random_Effect = c(Random_Effect, random_effect)
          if (j < randomstart) {
            timeint = 0
            intervention = 0
          }
          else {
            timeint = j - randomstart + 1
            intervention = 1}
          TimeInt = c(TimeInt, timeint)
          Intervention = c(Intervention, intervention)
          HONOS = Beta0 + Beta1*time + Beta2*intervention + Beta3*timeint + random_effect + random_error_corr
          HONOS_scores <- c(HONOS_scores, HONOS)
        }
      }
    }


    dataset <- cbind(Patient_ID, Cluster_ID, Intervention, Time, TimeInt, HONOS_scores, Random_Effect, Random_Error)
    dataset <- as.data.frame(dataset)
    write.csv(dataset, file = paste(file_loc,"Sim6_",N,".csv", sep =""))
  }
}
set.seed(12345)
Sim6(nSim)


##Simulation D13
Sim3 <- function(nSim) {
  for (N in 1:nSim){
```

```
    sigmab = 4.415
    sigmae = 5.442
    sigmac = 0.9570
    rho = -0.5

NumCentre = 12
NSteps = NumCentre + 1
AverageClusterSize = 20

centres = seq(1,NumCentre)
random_centres = sample(centres, NumCentre, replace = FALSE)

Beta0 <- 14.00 #Mean
Beta1 <- 0.25 #Time
Beta2 <- 2 #Intervention
Beta3 <- 0.15 #Intervention specific Time

Patient_ID = NULL
Time = NULL
TimeInt = NULL
Intervention = NULL
HONOS_scores = NULL
Cluster_ID = NULL
Random_Error = NULL
Random_Effect = NULL
for (i in 1:NumCentre) {
  random_effect_clust <- rnorm(1,0,sigmac)
  for (k in 1:AverageClusterSize) {
    random_effect = rnorm(1,0,sigmab)
    for (j in 1:NSteps) {
      randomstart = match(i, random_centres) + 1
      patientnumber = (i - 1)*20 + k
      Patient_ID = c(Patient_ID, paste("Patient_",patientnumber, sep=""))
      Cluster_ID = c(Cluster_ID, paste("Cluster_",i, sep=""))
      time = j
      Time = c(Time, j)
      random_error = rnorm(1,0,sqrt((1-rho^2)*sigmae^2))
      if (j==1) {
        random_error_corr = random_error
      }
      if (j > 1) {
        random_error_corr = rho*Random_Error[(patientnumber-1)*NSteps + 1] + random_error
      }
```

```
      Random_Error = c(Random_Error, random_error_corr)
      Random_Effect = c(Random_Effect, random_effect)
      if (j < randomstart) {
        timeint = 0
        intervention = 0
      }
      else {
        timeint = j - randomstart + 1
        intervention = 1}
      TimeInt = c(TimeInt, timeint)
      Intervention = c(Intervention, intervention)
      HONOS = Beta0 + Beta1*time + Beta2*intervention + Beta3*timeint + random_effect + random_error_corr
      HONOS_scores <- c(HONOS_scores, HONOS)
    }
  }
}


dataset <- cbind(Patient_ID, Cluster_ID, Intervention, Time, TimeInt, HONOS_scores, Random_Effect, Random_Error)
dataset <- as.data.frame(dataset)
write.csv(dataset, file = paste(file_loc,"Sim3_",N,".csv", sep =""))
  }
}
set.seed(12345)
Sim3(nSim)

##Simulation D14
Sim7 <- function(nSim) {
  for (N in 1:nSim){
    sigmab = 4.415
    sigmae = 5.442
    sigmac = 0.9570
    rho = 0.5

    NumCentre = 12
    NSteps = NumCentre + 1
    AverageClusterSize = 20

    centres = seq(1,NumCentre)
    random_centres = sample(centres, NumCentre, replace = FALSE)

    Beta0 <- 14.00 #Mean
    Beta1 <- 0.25 #Time
```

```
Beta2 <- 2 #Intervention
Beta3 <- 0.15 #Intervention specific Time

Patient_ID = NULL
Time = NULL
TimeInt = NULL
Intervention = NULL
HONOS_scores = NULL
Cluster_ID = NULL
Random_Error = NULL
Random_Effect = NULL
for (i in 1:NumCentre) {
  random_effect_clust <- rnorm(1,0,sigmac)
  for (k in 1:AverageClusterSize) {
    random_effect = rnorm(1,0,sigmab)
    for (j in 1:NSteps) {
      randomstart = match(i, random_centres) + 1
      patientnumber = (i - 1)*20 + k
      Patient_ID = c(Patient_ID, paste("Patient_",patientnumber, sep=""))
      Cluster_ID = c(Cluster_ID, paste("Cluster_",i, sep=""))
      time = j
      Time = c(Time, j)
      random_error = rnorm(1,0,sqrt((1-rho^2)*sigmae^2))
      if (j==1) {
        random_error_corr = random_error
      }
      if (j > 1) {
        random_error_corr = rho*Random_Error[(patientnumber-1)*NSteps + 1] + random_error
      }
      Random_Error = c(Random_Error, random_error_corr)
      Random_Effect = c(Random_Effect, random_effect)
      if (j < randomstart) {
        timeint = 0
        intervention = 0
      }
      else {
        timeint = j - randomstart + 1
        intervention = 1}
      TimeInt = c(TimeInt, timeint)
      Intervention = c(Intervention, intervention)
      HONOS = Beta0 + Beta1*time + Beta2*intervention + Beta3*timeint + random_effect + random_error_corr
      HONOS_scores <- c(HONOS_scores, HONOS)
    }
```

```
      }
    }


    dataset <- cbind(Patient_ID, Cluster_ID, Intervention, Time, TimeInt, HONOS_scores, Random_Effect, Random_Error)
    dataset <- as.data.frame(dataset)
    write.csv(dataset, file = paste(file_loc,"Sim7_",N,".csv", sep =""))
  }
}
set.seed(12345)
Sim7(nSim)


##Simulation D15
Sim4 <- function(nSim) {
  for (N in 1:nSim){
    sigmab = 4.415
    sigmae = 5.442
    sigmac = 0.9570
    rho = -0.5

NumCentre = 12
NSteps = NumCentre + 1
AverageClusterSize = 20

centres = seq(1,NumCentre)
random_centres = sample(centres, NumCentre, replace = FALSE)

Beta0 <- 14.00 #Mean
Beta1 <- 0.25 #Time
Beta2 <- 0 #Intervention
Beta3 <- 0.15 #Intervention specific Time

Patient_ID = NULL
Time = NULL
TimeInt = NULL
Intervention = NULL
HONOS_scores = NULL
Cluster_ID = NULL
Random_Error = NULL
Random_Effect = NULL
for (i in 1:NumCentre) {
  random_effect_clust <- rnorm(1,0,sigmac)
```

```
  for (k in 1:AverageClusterSize) {
    random_effect = rnorm(1,0,sigmab)
    for (j in 1:NSteps) {
      randomstart = match(i, random_centres) + 1
      patientnumber = (i - 1)*20 + k
      Patient_ID = c(Patient_ID, paste("Patient_",patientnumber, sep=""))
      Cluster_ID = c(Cluster_ID, paste("Cluster_",i, sep=""))
      time = j
      Time = c(Time, j)
      random_error = rnorm(1,0,sqrt((1-rho^2)*sigmae^2))
      if (j==1) {
        random_error_corr = random_error
      }
      if (j > 1) {
        random_error_corr = rho*Random_Error[(patientnumber-1)*NSteps + 1] + random_error
      }
      Random_Error = c(Random_Error, random_error_corr)
      Random_Effect = c(Random_Effect, random_effect)
      if (j < randomstart) {
        timeint = 0
        intervention = 0
      }
      else {
        timeint = j - randomstart + 1
        intervention = 1}
      TimeInt = c(TimeInt, timeint)
      Intervention = c(Intervention, intervention)
      HONOS = Beta0 + Beta1*time + Beta2*intervention + Beta3*timeint + random_effect + random_error_corr
      HONOS_scores <- c(HONOS_scores, HONOS)
    }
  }
}


dataset <- cbind(Patient_ID, Cluster_ID, Intervention, Time, TimeInt, HONOS_scores, Random_Effect, Random_Error)
dataset <- as.data.frame(dataset)
write.csv(dataset, file = paste(file_loc,"Sim4_",N,".csv", sep =""))
  }
}
set.seed(12345)
Sim4(nSim)
```

```
##Simulation D16
Sim8 <- function(nSim) {
  for (N in 1:nSim){
    sigmab = 4.415
    sigmae = 5.442
    sigmac = 0.9570
    rho = 0.5

NumCentre = 12
NSteps = NumCentre + 1
AverageClusterSize = 20

centres = seq(1,NumCentre)
random_centres = sample(centres, NumCentre, replace = FALSE)

Beta0 <- 14.00 #Mean
Beta1 <- 0.25 #Time
Beta2 <- 0 #Intervention
Beta3 <- 0.15 #Intervention specific Time

Patient_ID = NULL
Time = NULL
TimeInt = NULL
Intervention = NULL
HONOS_scores = NULL
Cluster_ID = NULL
Random_Error = NULL
Random_Effect = NULL
for (i in 1:NumCentre) {
  random_effect_clust <- rnorm(1,0,sigmac)
  for (k in 1:AverageClusterSize) {
    random_effect = rnorm(1,0,sigmab)
    for (j in 1:NSteps) {
      randomstart = match(i, random_centres) + 1
      patientnumber = (i - 1)*20 + k
      Patient_ID = c(Patient_ID, paste("Patient_",patientnumber, sep=""))
      Cluster_ID = c(Cluster_ID, paste("Cluster_",i, sep=""))
      time = j
      Time = c(Time, j)
      random_error = rnorm(1,0,sqrt((1-rho^2)*sigmae^2))
      if (j==1) {
        random_error_corr = random_error
      }
```

```
      if (j > 1) {
        random_error_corr = rho*Random_Error[(patientnumber-1)*NSteps + 1] + random_error
      }
      Random_Error = c(Random_Error, random_error_corr)
      Random_Effect = c(Random_Effect, random_effect)
      if (j < randomstart) {
        timeint = 0
        intervention = 0
      }
      else {
        timeint = j - randomstart + 1
        intervention = 1}
      TimeInt = c(TimeInt, timeint)
      Intervention = c(Intervention, intervention)
      HONOS = Beta0 + Beta1*time + Beta2*intervention + Beta3*timeint + random_effect + random_error_corr
      HONOS_scores <- c(HONOS_scores, HONOS)
    }
  }
}


dataset <- cbind(Patient_ID, Cluster_ID, Intervention, Time, TimeInt, HONOS_scores, Random_Effect, Random_Error)
dataset <- as.data.frame(dataset)
write.csv(dataset, file = paste(file_loc,"Sim8_",N,".csv", sep =""))
  }
}
set.seed(12345)
Sim8(nSim)


##Simulation D17
Sim15 <- function(nSim) {
  for (N in 1:nSim){
    sigmab = 4.415
    sigmae = 5.442
    sigmac = 0.9570
    rho = -0.5

    NumCentre = 12
    NSteps = NumCentre + 1
    AverageClusterSize = 20

    centres = seq(1,NumCentre)
```

```
random_centres = sample(centres, NumCentre, replace = FALSE)

Beta0 <- 14.00 #Mean
Beta1 <- 0.25 #Time
Beta2 <- 2 #Intervention
Beta3 <- 0.25 #Intervention specific Time

Patient_ID = NULL
Time = NULL
TimeInt = NULL
Intervention = NULL
HONOS_scores = NULL
Cluster_ID = NULL
Random_Error = NULL
Random_Effect = NULL
for (i in 1:NumCentre) {
  random_effect_clust <- rnorm(1,0,sigmac)
  for (k in 1:AverageClusterSize) {
    random_effect = rnorm(1,0,sigmab)
    for (j in 1:NSteps) {
      randomstart = match(i, random_centres) + 1
      patientnumber = (i - 1)*20 + k
      Patient_ID = c(Patient_ID, paste("Patient_",patientnumber, sep=""))
      Cluster_ID = c(Cluster_ID, paste("Cluster_",i, sep=""))
      time = j
      Time = c(Time, j)
      random_error = rnorm(1,0,sqrt((1-rho^2)*sigmae^2))
      if (j==1) {
        random_error_corr = random_error
      }
      if (j > 1) {
        random_error_corr = rho*Random_Error[(patientnumber-1)*NSteps + 1] + random_error
      }
      Random_Error = c(Random_Error, random_error_corr)
      Random_Effect = c(Random_Effect, random_effect)
      if (j < randomstart) {
        timeint = 0
        intervention = 0
      }
      else {
        timeint = j - randomstart + 1
        intervention = 1}
      TimeInt = c(TimeInt, timeint)
```

```
          Intervention = c(Intervention, intervention)
          HONOS = Beta0 + Beta1*time + Beta2*intervention + Beta3*timeint + random_effect + random_error_corr
          HONOS_scores <- c(HONOS_scores, HONOS)
        }
      }
    }


    dataset <- cbind(Patient_ID, Cluster_ID, Intervention, Time, TimeInt, HONOS_scores, Random_Effect, Random_Error)
    dataset <- as.data.frame(dataset)
    write.csv(dataset, file = paste(file_loc,"Sim15_",N,".csv", sep =""))
  }
}
set.seed(12345)
Sim15(nSim)


##Simulation D18
Sim16 <- function(nSim) {
  for (N in 1:nSim){
    sigmab = 4.415
    sigmae = 5.442
    sigmac = 0.9570
    rho = 0.5

    NumCentre = 12
    NSteps = NumCentre + 1
    AverageClusterSize = 20

    centres = seq(1,NumCentre)
    random_centres = sample(centres, NumCentre, replace = FALSE)

    Beta0 <- 14.00 #Mean
    Beta1 <- 0.25 #Time
    Beta2 <- 2 #Intervention
    Beta3 <- 0.25 #Intervention specific Time

    Patient_ID = NULL
    Time = NULL
    TimeInt = NULL
    Intervention = NULL
    HONOS_scores = NULL
    Cluster_ID = NULL
```

```
    Random_Error = NULL
    Random_Effect = NULL
    for (i in 1:NumCentre) {
      random_effect_clust <- rnorm(1,0,sigmac)
      for (k in 1:AverageClusterSize) {
        random_effect = rnorm(1,0,sigmab)
        for (j in 1:NSteps) {
          randomstart = match(i, random_centres) + 1
          patientnumber = (i - 1)*20 + k
          Patient_ID = c(Patient_ID, paste("Patient_",patientnumber, sep=""))
          Cluster_ID = c(Cluster_ID, paste("Cluster_",i, sep=""))
          time = j
          Time = c(Time, j)
          random_error = rnorm(1,0,sqrt((1-rho^2)*sigmae^2))
          if (j==1) {
            random_error_corr = random_error
          }
          if (j > 1) {
            random_error_corr = rho*Random_Error[(patientnumber-1)*NSteps + 1] + random_error
          }
          Random_Error = c(Random_Error, random_error_corr)
          Random_Effect = c(Random_Effect, random_effect)
          if (j < randomstart) {
            timeint = 0
            intervention = 0
          }
          else {
            timeint = j - randomstart + 1
            intervention = 1}
          TimeInt = c(TimeInt, timeint)
          Intervention = c(Intervention, intervention)
          HONOS = Beta0 + Beta1*time + Beta2*intervention + Beta3*timeint + random_effect + random_error_corr
          HONOS_scores <- c(HONOS_scores, HONOS)
        }
      }
    }


    dataset <- cbind(Patient_ID, Cluster_ID, Intervention, Time, TimeInt, HONOS_scores, Random_Effect, Random_Error)
    dataset <- as.data.frame(dataset)
    write.csv(dataset, file = paste(file_loc,"Sim16_",N,".csv", sep =""))
  }
}
```

```
set.seed(12345)
Sim16(nSim)


##Simulation D19
Sim17 <- function(nSim) {
  for (N in 1:nSim){
    sigmab = 4.415
    sigmae = 5.442
    sigmac = 0.9570
    rho = -0.5

    NumCentre = 12
    NSteps = NumCentre + 1
    AverageClusterSize = 20

    centres = seq(1,NumCentre)
    random_centres = sample(centres, NumCentre, replace = FALSE)

    Beta0 <- 14.00 #Mean
    Beta1 <- 0.25 #Time
    Beta2 <- 0 #Intervention
    Beta3 <- 0.25 #Intervention specific Time

    Patient_ID = NULL
    Time = NULL
    TimeInt = NULL
    Intervention = NULL
    HONOS_scores = NULL
    Cluster_ID = NULL
    Random_Error = NULL
    Random_Effect = NULL
    for (i in 1:NumCentre) {
      random_effect_clust <- rnorm(1,0,sigmac)
      for (k in 1:AverageClusterSize) {
        random_effect = rnorm(1,0,sigmab)
        for (j in 1:NSteps) {
          randomstart = match(i, random_centres) + 1
          patientnumber = (i - 1)*20 + k
          Patient_ID = c(Patient_ID, paste("Patient_",patientnumber, sep=""))
          Cluster_ID = c(Cluster_ID, paste("Cluster_",i, sep=""))
          time = j
          Time = c(Time, j)
```

```
          random_error = rnorm(1,0,sqrt((1-rho^2)*sigmae^2))
          if (j==1) {
            random_error_corr = random_error
          }
          if (j > 1) {
            random_error_corr = rho*Random_Error[(patientnumber-1)*NSteps + 1] + random_error
          }
          Random_Error = c(Random_Error, random_error_corr)
          Random_Effect = c(Random_Effect, random_effect)
          if (j < randomstart) {
            timeint = 0
            intervention = 0
          }
          else {
            timeint = j - randomstart + 1
            intervention = 1}
          TimeInt = c(TimeInt, timeint)
          Intervention = c(Intervention, intervention)
          HONOS = Beta0 + Beta1*time + Beta2*intervention + Beta3*timeint + random_effect + random_error_corr
          HONOS_scores <- c(HONOS_scores, HONOS)
        }
      }
    }


    dataset <- cbind(Patient_ID, Cluster_ID, Intervention, Time, TimeInt, HONOS_scores, Random_Effect, Random_Error)
    dataset <- as.data.frame(dataset)
    write.csv(dataset, file = paste(file_loc,"Sim17_",N,".csv", sep =""))
  }
}
set.seed(12345)
Sim17(nSim)


##Simulation D20
Sim18 <- function(nSim) {
  for (N in 1:nSim){
    sigmab = 4.415
    sigmae = 5.442
    sigmac = 0.9570
    rho = 0.5

    NumCentre = 12
```

```
NSteps = NumCentre + 1
AverageClusterSize = 20

centres = seq(1,NumCentre)
random_centres = sample(centres, NumCentre, replace = FALSE)

Beta0 <- 14.00 #Mean
Beta1 <- 0.25 #Time
Beta2 <- 0 #Intervention
Beta3 <- 0.25 #Intervention specific Time

Patient_ID = NULL
Time = NULL
TimeInt = NULL
Intervention = NULL
HONOS_scores = NULL
Cluster_ID = NULL
Random_Error = NULL
Random_Effect = NULL
for (i in 1:NumCentre) {
  random_effect_clust <- rnorm(1,0,sigmac)
  for (k in 1:AverageClusterSize) {
    random_effect = rnorm(1,0,sigmab)
    for (j in 1:NSteps) {
      randomstart = match(i, random_centres) + 1
      patientnumber = (i - 1)*20 + k
      Patient_ID = c(Patient_ID, paste("Patient_",patientnumber, sep=""))
      Cluster_ID = c(Cluster_ID, paste("Cluster_",i, sep=""))
      time = j
      Time = c(Time, j)
      random_error = rnorm(1,0,sqrt((1-rho^2)*sigmae^2))
      if (j==1) {
        random_error_corr = random_error
      }
      if (j > 1) {
        random_error_corr = rho*Random_Error[(patientnumber-1)*NSteps + 1] + random_error
      }
      Random_Error = c(Random_Error, random_error_corr)
      Random_Effect = c(Random_Effect, random_effect)
      if (j < randomstart) {
        timeint = 0
        intervention = 0
      }
```

```
          else {
            timeint = j - randomstart + 1
            intervention = 1}
          TimeInt = c(TimeInt, timeint)
          Intervention = c(Intervention, intervention)
          HONOS = Beta0 + Beta1*time + Beta2*intervention + Beta3*timeint + random_effect + random_error_corr
          HONOS_scores <- c(HONOS_scores, HONOS)
        }
      }
    }


    dataset <- cbind(Patient_ID, Cluster_ID, Intervention, Time, TimeInt, HONOS_scores, Random_Effect, Random_Error)
    dataset <- as.data.frame(dataset)
    write.csv(dataset, file = paste(file_loc,"Sim18_",N,".csv", sep =""))
  }
}
set.seed(12345)
Sim18(nSim)


##Simulation D21
Sim19 <- function(nSim) {
  for (N in 1:nSim){
    sigmab = 4.415
    sigmae = 5.442
    sigmac = 0.9570
    rho = -0.5

    NumCentre = 12
    NSteps = NumCentre + 1
    AverageClusterSize = 20

    centres = seq(1,NumCentre)
    random_centres = sample(centres, NumCentre, replace = FALSE)

    Beta0 <- 14.00 #Mean
    Beta1 <- 0.25 #Time
    Beta2 <- -2 #Intervention
    Beta3 <- -0.5 #Intervention specific Time

    Patient_ID = NULL
    Time = NULL
```

```
TimeInt = NULL
Intervention = NULL
HONOS_scores = NULL
Cluster_ID = NULL
Random_Error = NULL
Random_Effect = NULL
for (i in 1:NumCentre) {
  random_effect_clust <- rnorm(1,0,sigmac)
  for (k in 1:AverageClusterSize) {
    random_effect = rnorm(1,0,sigmab)
    for (j in 1:NSteps) {
      randomstart = match(i, random_centres) + 1
      patientnumber = (i - 1)*20 + k
      Patient_ID = c(Patient_ID, paste("Patient_",patientnumber, sep=""))
      Cluster_ID = c(Cluster_ID, paste("Cluster_",i, sep=""))
      time = j
      Time = c(Time, j)
      random_error = rnorm(1,0,sqrt((1-rho^2)*sigmae^2))
      if (j==1) {
        random_error_corr = random_error
      }
      if (j > 1) {
        random_error_corr = rho*Random_Error[(patientnumber-1)*NSteps + 1] + random_error
      }
      Random_Error = c(Random_Error, random_error_corr)
      Random_Effect = c(Random_Effect, random_effect)
      if (j < randomstart) {
        timeint = 0
        intervention = 0
      }
      else {
        timeint = j - randomstart + 1
        intervention = 1}
      TimeInt = c(TimeInt, timeint)
      Intervention = c(Intervention, intervention)
      HONOS = Beta0 + Beta1*time + Beta2*intervention + Beta3*timeint + random_effect + random_error_corr
      HONOS_scores <- c(HONOS_scores, HONOS)
    }
  }
}


dataset <- cbind(Patient_ID, Cluster_ID, Intervention, Time, TimeInt, HONOS_scores, Random_Effect, Random_Error)
```

```
    dataset <- as.data.frame(dataset)
    write.csv(dataset, file = paste(file_loc,"Sim19_",N,".csv", sep =""))
  }
}
set.seed(12345)
Sim19(nSim)


##Simulation D22
Sim20 <- function(nSim) {
  for (N in 1:nSim){
    sigmab = 4.415
    sigmae = 5.442
    sigmac = 0.9570
    rho = 0.5

    NumCentre = 12
    NSteps = NumCentre + 1
    AverageClusterSize = 20

    centres = seq(1,NumCentre)
    random_centres = sample(centres, NumCentre, replace = FALSE)

    Beta0 <- 14.00 #Mean
    Beta1 <- 0.25 #Time
    Beta2 <- -2 #Intervention
    Beta3 <- -0.5 #Intervention specific Time

    Patient_ID = NULL
    Time = NULL
    TimeInt = NULL
    Intervention = NULL
    HONOS_scores = NULL
    Cluster_ID = NULL
    Random_Error = NULL
    Random_Effect = NULL
    for (i in 1:NumCentre) {
      random_effect_clust <- rnorm(1,0,sigmac)
      for (k in 1:AverageClusterSize) {
        random_effect = rnorm(1,0,sigmab)
        for (j in 1:NSteps) {
          randomstart = match(i, random_centres) + 1
          patientnumber = (i - 1)*20 + k
```

```
          Patient_ID = c(Patient_ID, paste("Patient_",patientnumber, sep=""))
          Cluster_ID = c(Cluster_ID, paste("Cluster_",i, sep=""))
          time = j
          Time = c(Time, j)
          random_error = rnorm(1,0,sqrt((1-rho^2)*sigmae^2))
          if (j==1) {
            random_error_corr = random_error
          }
          if (j > 1) {
            random_error_corr = rho*Random_Error[(patientnumber-1)*NSteps + 1] + random_error
          }
          Random_Error = c(Random_Error, random_error_corr)
          Random_Effect = c(Random_Effect, random_effect)
          if (j < randomstart) {
            timeint = 0
            intervention = 0
          }
          else {
            timeint = j - randomstart + 1
            intervention = 1}
          TimeInt = c(TimeInt, timeint)
          Intervention = c(Intervention, intervention)
          HONOS = Beta0 + Beta1*time + Beta2*intervention + Beta3*timeint + random_effect + random_error_corr
          HONOS_scores <- c(HONOS_scores, HONOS)
        }
      }
    }


    dataset <- cbind(Patient_ID, Cluster_ID, Intervention, Time, TimeInt, HONOS_scores, Random_Effect, Random_Error)
    dataset <- as.data.frame(dataset)
    write.csv(dataset, file = paste(file_loc,"Sim20_",N,".csv", sep =""))
  }
}
set.seed(12345)
Sim20(nSim)


##Simulation D23
Sim21 <- function(nSim) {
  for (N in 1:nSim){
    sigmab = 4.415
    sigmae = 5.442
```

```
sigmac = 0.9570
rho = -0.5

NumCentre = 12
NSteps = NumCentre + 1
AverageClusterSize = 20

centres = seq(1,NumCentre)
random_centres = sample(centres, NumCentre, replace = FALSE)

Beta0 <- 14.00 #Mean
Beta1 <- 0.25 #Time
Beta2 <- 0 #Intervention
Beta3 <- -0.5 #Intervention specific Time

Patient_ID = NULL
Time = NULL
TimeInt = NULL
Intervention = NULL
HONOS_scores = NULL
Cluster_ID = NULL
Random_Error = NULL
Random_Effect = NULL
for (i in 1:NumCentre) {
  random_effect_clust <- rnorm(1,0,sigmac)
  for (k in 1:AverageClusterSize) {
    random_effect = rnorm(1,0,sigmab)
    for (j in 1:NSteps) {
      randomstart = match(i, random_centres) + 1
      patientnumber = (i - 1)*20 + k
      Patient_ID = c(Patient_ID, paste("Patient_",patientnumber, sep=""))
      Cluster_ID = c(Cluster_ID, paste("Cluster_",i, sep=""))
      time = j
      Time = c(Time, j)
      random_error = rnorm(1,0,sqrt((1-rho^2)*sigmae^2))
      if (j==1) {
        random_error_corr = random_error
      }
      if (j > 1) {
        random_error_corr = rho*Random_Error[(patientnumber-1)*NSteps + 1] + random_error
      }
      Random_Error = c(Random_Error, random_error_corr)
      Random_Effect = c(Random_Effect, random_effect)
```

```
          if (j < randomstart) {
            timeint = 0
            intervention = 0
          }
          else {
            timeint = j - randomstart + 1
            intervention = 1}
          TimeInt = c(TimeInt, timeint)
          Intervention = c(Intervention, intervention)
          HONOS = Beta0 + Beta1*time + Beta2*intervention + Beta3*timeint + random_effect + random_error_corr
          HONOS_scores <- c(HONOS_scores, HONOS)
        }
      }
    }


    dataset <- cbind(Patient_ID, Cluster_ID, Intervention, Time, TimeInt, HONOS_scores, Random_Effect, Random_Error)
    dataset <- as.data.frame(dataset)
    write.csv(dataset, file = paste(file_loc,"Sim21_",N,".csv", sep =""))
  }
}
set.seed(12345)
Sim21(nSim)


##Simulation D24
Sim22 <- function(nSim) {
  for (N in 1:nSim){
    sigmab = 4.415
    sigmae = 5.442
    sigmac = 0.9570
    rho = 0.5

    NumCentre = 12
    NSteps = NumCentre + 1
    AverageClusterSize = 20

    centres = seq(1,NumCentre)
    random_centres = sample(centres, NumCentre, replace = FALSE)

    Beta0 <- 14.00 #Mean
    Beta1 <- 0.25 #Time
    Beta2 <- 0 #Intervention
```

```
Beta3 <- -0.5 #Intervention specific Time

Patient_ID = NULL
Time = NULL
TimeInt = NULL
Intervention = NULL
HONOS_scores = NULL
Cluster_ID = NULL
Random_Error = NULL
Random_Effect = NULL
for (i in 1:NumCentre) {
  random_effect_clust <- rnorm(1,0,sigmac)
  for (k in 1:AverageClusterSize) {
    random_effect = rnorm(1,0,sigmab)
    for (j in 1:NSteps) {
      randomstart = match(i, random_centres) + 1
      patientnumber = (i - 1)*20 + k
      Patient_ID = c(Patient_ID, paste("Patient_",patientnumber, sep=""))
      Cluster_ID = c(Cluster_ID, paste("Cluster_",i, sep=""))
      time = j
      Time = c(Time, j)
      random_error = rnorm(1,0,sqrt((1-rho^2)*sigmae^2))
      if (j==1) {
        random_error_corr = random_error
      }
      if (j > 1) {
        random_error_corr = rho*Random_Error[(patientnumber-1)*NSteps + 1] + random_error
      }
      Random_Error = c(Random_Error, random_error_corr)
      Random_Effect = c(Random_Effect, random_effect)
      if (j < randomstart) {
        timeint = 0
        intervention = 0
      }
      else {
        timeint = j - randomstart + 1
        intervention = 1}
      TimeInt = c(TimeInt, timeint)
      Intervention = c(Intervention, intervention)
      HONOS = Beta0 + Beta1*time + Beta2*intervention + Beta3*timeint + random_effect + random_error_corr
      HONOS_scores <- c(HONOS_scores, HONOS)
    }
  }
```

```
    }

    dataset <- cbind(Patient_ID, Cluster_ID, Intervention, Time, TimeInt, HONOS_scores, Random_Effect, Random_Error)
    dataset <- as.data.frame(dataset)
    write.csv(dataset, file = paste(file_loc,"Sim22_",N,".csv", sep =""))
  }
}
set.seed(12345)
Sim22(nSim)


##Simulation D25
Sim9 <- function(nSim) {
  for (N in 1:nSim){
    sigmab = 4.415
    sigmae = 5.442
    sigmac = 0.9570
    rho = -0.5

NumCentre = 12
NSteps = NumCentre + 1
AverageClusterSize = 20


centres = seq(1,NumCentre)
random_centres = sample(centres, NumCentre, replace = FALSE)

Beta0 <- 14.00 #Mean
Beta1 <- 1 #Time
Beta2 <- 2 #Intervention
Beta3 <- 0 #Intervention specific Time

x <- seq(0,12)
y <- sin(x*180/12*pi/180)
Beta1_time <- y*2*Beta1
Beta3_time <- y*1*Beta3
Time_factor <- seq(1,13)
Time_factor <- factor(Time_factor)

Patient_ID = NULL
Time = NULL
TimeInt = NULL
```

```
Intervention = NULL
HONOS_scores = NULL
Cluster_ID = NULL
Random_Error = NULL
Random_Effect = NULL
for (i in 1:NumCentre) {
  random_effect_clust <- rnorm(1,0,sigmac)
  for (k in 1:AverageClusterSize) {
    random_effect = rnorm(1,0,sigmab)
    for (j in 1:NSteps) {
      randomstart = match(i, random_centres) + 1
      patientnumber = (i - 1)*20 + k
      Patient_ID = c(Patient_ID, paste("Patient_",patientnumber, sep=""))
      Cluster_ID = c(Cluster_ID, paste("Cluster_",i, sep=""))
      time = j
      Time = c(Time, j)
      random_error = rnorm(1,0,sqrt((1-rho^2)*sigmae^2))
      if (j==1) {
        random_error_corr = random_error
      }
      if (j > 1) {
        random_error_corr = rho*Random_Error[(patientnumber-1)*NSteps + 1] + random_error
      }
      Random_Error = c(Random_Error, random_error_corr)
      Random_Effect = c(Random_Effect, random_effect)
      if (j < randomstart) {
        timeint = 0
        intervention = 0
      }
      else {
        timeint = j - randomstart + 1
        intervention = 1}
      TimeInt = c(TimeInt, timeint)
      Intervention = c(Intervention, intervention)
      HONOS = Beta0 + Beta1_time[match(factor(time),Time_factor)] + Beta2*intervention +
Beta3_time[match(factor(time),Time_factor)] + random_effect + random_error_corr
      HONOS_scores <- c(HONOS_scores, HONOS)
    }
  }
}


dataset <- cbind(Patient_ID, Cluster_ID, Intervention, Time, TimeInt, HONOS_scores, Random_Effect, Random_Error)
```

```
dataset <- as.data.frame(dataset)
write.csv(dataset, file = paste(file_loc,"Sim9_",N,".csv", sep =""))
  }
}
set.seed(12345)
Sim9(nSim)


##Simulation D26
Sim12 <- function(nSim) {
  for (N in 1:nSim){
    sigmab = 4.415
    sigmae = 5.442
    sigmac = 0.9570
    rho = 0.5

    NumCentre = 12
    NSteps = NumCentre + 1
    AverageClusterSize = 20


    centres = seq(1,NumCentre)
    random_centres = sample(centres, NumCentre, replace = FALSE)

    Beta0 <- 14.00 #Mean
    Beta1 <- 1 #Time
    Beta2 <- 2 #Intervention
    Beta3 <- 0 #Intervention specific Time

    x <- seq(0,12)
    y <- sin(x*180/12*pi/180)
    Beta1_time <- y*2*Beta1
    Beta3_time <- y*1*Beta3
    Time_factor <- seq(1,13)
    Time_factor <- factor(Time_factor)

    Patient_ID = NULL
    Time = NULL
    TimeInt = NULL
    Intervention = NULL
    HONOS_scores = NULL
    Cluster_ID = NULL
    Random_Error = NULL
```

```
    Random_Effect = NULL
    for (i in 1:NumCentre) {
      random_effect_clust <- rnorm(1,0,sigmac)
      for (k in 1:AverageClusterSize) {
        random_effect = rnorm(1,0,sigmab)
        for (j in 1:NSteps) {
          randomstart = match(i, random_centres) + 1
          patientnumber = (i - 1)*20 + k
          Patient_ID = c(Patient_ID, paste("Patient_",patientnumber, sep=""))
          Cluster_ID = c(Cluster_ID, paste("Cluster_",i, sep=""))
          time = j
          Time = c(Time, j)
          random_error = rnorm(1,0,sqrt((1-rho^2)*sigmae^2))
          if (j==1) {
            random_error_corr = random_error
          }
          if (j > 1) {
            random_error_corr = rho*Random_Error[(patientnumber-1)*NSteps + 1] + random_error
          }
          Random_Error = c(Random_Error, random_error_corr)
          Random_Effect = c(Random_Effect, random_effect)
          if (j < randomstart) {
            timeint = 0
            intervention = 0
          }
          else {
            timeint = j - randomstart + 1
            intervention = 1}
          TimeInt = c(TimeInt, timeint)
          Intervention = c(Intervention, intervention)
          HONOS = Beta0 + Beta1_time[match(factor(time),Time_factor)] + Beta2*intervention +
Beta3_time[match(factor(time),Time_factor)] + random_effect + random_error_corr
          HONOS_scores <- c(HONOS_scores, HONOS)
        }
      }
    }


    dataset <- cbind(Patient_ID, Cluster_ID, Intervention, Time, TimeInt, HONOS_scores, Random_Effect, Random_Error)
    dataset <- as.data.frame(dataset)
    write.csv(dataset, file = paste(file_loc,"Sim12_",N,".csv", sep =""))
  }
}
```

```
set.seed(12345)
Sim12(nSim)



##Simulation D27
Sim10 <- function(nSim) {
  for (N in 1:nSim){
    sigmab = 4.415
    sigmae = 5.442
    sigmac = 0.9570
    rho = -0.5

NumCentre = 12
NSteps = NumCentre + 1
AverageClusterSize = 20


centres = seq(1,NumCentre)
random_centres = sample(centres, NumCentre, replace = FALSE)

Beta0 <- 14.00 #Mean
Beta1 <- 1 #Time
Beta2 <- 2 #Intervention
Beta3 <- 1 #Intervention specific Time

x <- seq(0,12)
y <- sin(x*180/12*pi/180)
Beta1_time <- y*2*Beta1
Beta3_time <- y*1*Beta3
Time_factor <- seq(1,13)
Time_factor <- factor(Time_factor)

Patient_ID = NULL
Time = NULL
TimeInt = NULL
Intervention = NULL
HONOS_scores = NULL
Cluster_ID = NULL
Random_Error = NULL
Random_Effect = NULL
for (i in 1:NumCentre) {
  random_effect_clust <- rnorm(1,0,sigmac)
```

```
  for (k in 1:AverageClusterSize) {
    random_effect = rnorm(1,0,sigmab)
    for (j in 1:NSteps) {
      randomstart = match(i, random_centres) + 1
      patientnumber = (i - 1)*20 + k
      Patient_ID = c(Patient_ID, paste("Patient_",patientnumber, sep=""))
      Cluster_ID = c(Cluster_ID, paste("Cluster_",i, sep=""))
      time = j
      Time = c(Time, j)
      random_error = rnorm(1,0,sqrt((1-rho^2)*sigmae^2))
      if (j==1) {
        random_error_corr = random_error
      }
      if (j > 1) {
        random_error_corr = rho*Random_Error[(patientnumber-1)*NSteps + 1] + random_error
      }
      Random_Error = c(Random_Error, random_error_corr)
      Random_Effect = c(Random_Effect, random_effect)
      if (j < randomstart) {
        timeint = 0
        intervention = 0
      }
      else {
        timeint = j - randomstart + 1
        intervention = 1}
      TimeInt = c(TimeInt, timeint)
      Intervention = c(Intervention, intervention)
      HONOS = Beta0 + Beta1_time[match(factor(time),Time_factor)] + Beta2*intervention +
Beta3_time[match(factor(timeint),Time_factor)] + random_effect + random_error_corr
      HONOS_scores <- c(HONOS_scores, HONOS)
    }
  }
}


dataset <- cbind(Patient_ID, Cluster_ID, Intervention, Time, TimeInt, HONOS_scores, Random_Effect, Random_Error)
dataset <- as.data.frame(dataset)
write.csv(dataset, file = paste(file_loc,"Sim10_",N,".csv", sep =""))
  }
}
set.seed(12345)
Sim10(nSim)
```

```
##Simulation D28
Sim13 <- function(nSim) {
  for (N in 1:nSim){
    sigmab = 4.415
    sigmae = 5.442
    sigmac = 0.9570
    rho = 0.5

    NumCentre = 12
    NSteps = NumCentre + 1
    AverageClusterSize = 20


    centres = seq(1,NumCentre)
    random_centres = sample(centres, NumCentre, replace = FALSE)

    Beta0 <- 14.00 #Mean
    Beta1 <- 1 #Time
    Beta2 <- 2 #Intervention
    Beta3 <- 1 #Intervention specific Time

    x <- seq(0,12)
    y <- sin(x*180/12*pi/180)
    Beta1_time <- y*2*Beta1
    Beta3_time <- y*1*Beta3
    Time_factor <- seq(1,13)
    Time_factor <- factor(Time_factor)

    Patient_ID = NULL
    Time = NULL
    TimeInt = NULL
    Intervention = NULL
    HONOS_scores = NULL
    Cluster_ID = NULL
    Random_Error = NULL
    Random_Effect = NULL
    for (i in 1:NumCentre) {
      random_effect_clust <- rnorm(1,0,sigmac)
      for (k in 1:AverageClusterSize) {
        random_effect = rnorm(1,0,sigmab)
        for (j in 1:NSteps) {
          randomstart = match(i, random_centres) + 1
```

```
          patientnumber = (i - 1)*20 + k
          Patient_ID = c(Patient_ID, paste("Patient_",patientnumber, sep=""))
          Cluster_ID = c(Cluster_ID, paste("Cluster_",i, sep=""))
          time = j
          Time = c(Time, j)
          random_error = rnorm(1,0,sqrt((1-rho^2)*sigmae^2))
          if (j==1) {
            random_error_corr = random_error
          }
          if (j > 1) {
            random_error_corr = rho*Random_Error[(patientnumber-1)*NSteps + 1] + random_error
          }
          Random_Error = c(Random_Error, random_error_corr)
          Random_Effect = c(Random_Effect, random_effect)
          if (j < randomstart) {
            timeint = 0
            intervention = 0
          }
          else {
            timeint = j - randomstart + 1
            intervention = 1}
          TimeInt = c(TimeInt, timeint)
          Intervention = c(Intervention, intervention)
          HONOS = Beta0 + Beta1_time[match(factor(time),Time_factor)] + Beta2*intervention +
Beta3_time[match(factor(timeint),Time_factor)] + random_effect + random_error_corr
          HONOS_scores <- c(HONOS_scores, HONOS)
        }
      }
    }


    dataset <- cbind(Patient_ID, Cluster_ID, Intervention, Time, TimeInt, HONOS_scores, Random_Effect, Random_Error)
    dataset <- as.data.frame(dataset)
    write.csv(dataset, file = paste(file_loc,"Sim13_",N,".csv", sep =""))
  }
}
set.seed(12345)
Sim13(nSim)


##Simulation D29
Sim11 <- function(nSim) {
  for (N in 1:nSim){
```

```
    sigmab = 4.415
    sigmae = 5.442
    sigmac = 0.9570
    rho = -0.5

NumCentre = 12
NSteps = NumCentre + 1
AverageClusterSize = 20


centres = seq(1,NumCentre)
random_centres = sample(centres, NumCentre, replace = FALSE)

Beta0 <- 14.00 #Mean
Beta1 <- 1 #Time
Beta2 <- 0 #Intervention
Beta3 <- 1 #Intervention specific Time

x <- seq(0,12)
y <- sin(x*180/12*pi/180)
Beta1_time <- y*2*Beta1
Beta3_time <- y*1*Beta3
Time_factor <- seq(1,13)
Time_factor <- factor(Time_factor)

Patient_ID = NULL
Time = NULL
TimeInt = NULL
Intervention = NULL
HONOS_scores = NULL
Cluster_ID = NULL
Random_Error = NULL
Random_Effect = NULL
for (i in 1:NumCentre) {
  random_effect_clust <- rnorm(1,0,sigmac)
  for (k in 1:AverageClusterSize) {
    random_effect = rnorm(1,0,sigmab)
    for (j in 1:NSteps) {
      randomstart = match(i, random_centres) + 1
      patientnumber = (i - 1)*20 + k
      Patient_ID = c(Patient_ID, paste("Patient_",patientnumber, sep=""))
      Cluster_ID = c(Cluster_ID, paste("Cluster_",i, sep=""))
      time = j
```

```
      Time = c(Time, j)
      random_error = rnorm(1,0,sqrt((1-rho^2)*sigmae^2))
      if (j==1) {
        random_error_corr = random_error
      }
      if (j > 1) {
        random_error_corr = rho*Random_Error[(patientnumber-1)*NSteps + 1] + random_error
      }
      Random_Error = c(Random_Error, random_error_corr)
      Random_Effect = c(Random_Effect, random_effect)
      if (j < randomstart) {
        timeint = 0
        intervention = 0
      }
      else {
        timeint = j - randomstart + 1
        intervention = 1}
      TimeInt = c(TimeInt, timeint)
      Intervention = c(Intervention, intervention)
      HONOS = Beta0 + Beta1_time[match(factor(time),Time_factor)] + Beta2*intervention +
Beta3_time[match(factor(timeint),Time_factor)] + random_effect + random_error_corr
      HONOS_scores <- c(HONOS_scores, HONOS)
    }
  }
}


dataset <- cbind(Patient_ID, Cluster_ID, Intervention, Time, TimeInt, HONOS_scores, Random_Effect, Random_Error)
dataset <- as.data.frame(dataset)
write.csv(dataset, file = paste(file_loc,"Sim11_",N,".csv", sep =""))
  }
}
set.seed(12345)
Sim11(nSim)


##Simulation D30
Sim14 <- function(nSim) {
  for (N in 1:nSim){
    sigmab = 4.415
    sigmae = 5.442
    sigmac = 0.9570
    rho = 0.5
```

```
NumCentre = 12
NSteps = NumCentre + 1
AverageClusterSize = 20


centres = seq(1,NumCentre)
random_centres = sample(centres, NumCentre, replace = FALSE)

Beta0 <- 14.00 #Mean
Beta1 <- 1 #Time
Beta2 <- 0 #Intervention
Beta3 <- 1 #Intervention specific Time

x <- seq(0,12)
y <- sin(x*180/12*pi/180)
Beta1_time <- y*2*Beta1
Beta3_time <- y*1*Beta3
Time_factor <- seq(1,13)
Time_factor <- factor(Time_factor)

Patient_ID = NULL
Time = NULL
TimeInt = NULL
Intervention = NULL
HONOS_scores = NULL
Cluster_ID = NULL
Random_Error = NULL
Random_Effect = NULL
for (i in 1:NumCentre) {
  random_effect_clust <- rnorm(1,0,sigmac)
  for (k in 1:AverageClusterSize) {
    random_effect = rnorm(1,0,sigmab)
    for (j in 1:NSteps) {
      randomstart = match(i, random_centres) + 1
      patientnumber = (i - 1)*20 + k
      Patient_ID = c(Patient_ID, paste("Patient_",patientnumber, sep=""))
      Cluster_ID = c(Cluster_ID, paste("Cluster_",i, sep=""))
      time = j
      Time = c(Time, j)
      random_error = rnorm(1,0,sqrt((1-rho^2)*sigmae^2))
      if (j==1) {
        random_error_corr = random_error
```

```
      }
      if (j > 1) {
        random_error_corr = rho*Random_Error[(patientnumber-1)*NSteps + 1] + random_error
      }
      Random_Error = c(Random_Error, random_error_corr)
      Random_Effect = c(Random_Effect, random_effect)
      if (j < randomstart) {
        timeint = 0
        intervention = 0
      }
      else {
        timeint = j - randomstart + 1
        intervention = 1}
      TimeInt = c(TimeInt, timeint)
      Intervention = c(Intervention, intervention)
      HONOS = Beta0 + Beta1_time[match(factor(time),Time_factor)] + Beta2*intervention +
Beta3_time[match(factor(timeint),Time_factor)] + random_effect + random_error_corr
      HONOS_scores <- c(HONOS_scores, HONOS)
    }
  }
}


dataset <- cbind(Patient_ID, Cluster_ID, Intervention, Time, TimeInt, HONOS_scores, Random_Effect, Random_Error)
dataset <- as.data.frame(dataset)
write.csv(dataset, file = paste(file_loc,"Sim14_",N,".csv", sep =""))
  }
}
set.seed(12345)
Sim14(nSim)


##Simulation D31
Sim23 <- function(nSim) {
  for (N in 1:nSim){
    sigmab = 4.415
    sigmae = 5.442
    sigmac = 0.9570
    rho = -0.5

    NumCentre = 12
    NSteps = NumCentre + 1
    AverageClusterSize = 20
```

```
centres = seq(1,NumCentre)
random_centres = sample(centres, NumCentre, replace = FALSE)

Beta0 <- 14.00 #Mean
Beta1 <- 1 #Time
Beta2 <- 2 #Intervention
Beta3 <- 0 #Intervention specific Time

x <- seq(0,12)
y <- sin(x*180/6*pi/180)
Beta1_time <- y*2*Beta1
Beta3_time <- y*1*Beta3
Time_factor <- seq(1,13)
Time_factor <- factor(Time_factor)

Patient_ID = NULL
Time = NULL
TimeInt = NULL
Intervention = NULL
HONOS_scores = NULL
Cluster_ID = NULL
Random_Error = NULL
Random_Effect = NULL
for (i in 1:NumCentre) {
  random_effect_clust <- rnorm(1,0,sigmac)
  for (k in 1:AverageClusterSize) {
    random_effect = rnorm(1,0,sigmab)
    for (j in 1:NSteps) {
      randomstart = match(i, random_centres) + 1
      patientnumber = (i - 1)*20 + k
      Patient_ID = c(Patient_ID, paste("Patient_",patientnumber, sep=""))
      Cluster_ID = c(Cluster_ID, paste("Cluster_",i, sep=""))
      time = j
      Time = c(Time, j)
      random_error = rnorm(1,0,sqrt((1-rho^2)*sigmae^2))
      if (j==1) {
        random_error_corr = random_error
      }
      if (j > 1) {
        random_error_corr = rho*Random_Error[(patientnumber-1)*NSteps + 1] + random_error
      }
```

```
          Random_Error = c(Random_Error, random_error_corr)
          Random_Effect = c(Random_Effect, random_effect)
          if (j < randomstart) {
            timeint = 0
            intervention = 0
          }
          else {
            timeint = j - randomstart + 1
            intervention = 1}
          TimeInt = c(TimeInt, timeint)
          Intervention = c(Intervention, intervention)
          HONOS = Beta0 + Beta1_time[match(factor(time),Time_factor)] + Beta2*intervention +
Beta3_time[match(factor(time),Time_factor)] + random_effect + random_error_corr
          HONOS_scores <- c(HONOS_scores, HONOS)
        }
      }
    }


    dataset <- cbind(Patient_ID, Cluster_ID, Intervention, Time, TimeInt, HONOS_scores, Random_Effect, Random_Error)
    dataset <- as.data.frame(dataset)
    write.csv(dataset, file = paste(file_loc,"Sim23_",N,".csv", sep =""))
  }
}
set.seed(12345)
Sim23(nSim)


##Simulation D32
Sim26 <- function(nSim) {
  for (N in 1:nSim){
    sigmab = 4.415
    sigmae = 5.442
    sigmac = 0.9570
    rho = 0.5

    NumCentre = 12
    NSteps = NumCentre + 1
    AverageClusterSize = 20


    centres = seq(1,NumCentre)
    random_centres = sample(centres, NumCentre, replace = FALSE)
```

```
Beta0 <- 14.00 #Mean
Beta1 <- 1 #Time
Beta2 <- 2 #Intervention
Beta3 <- 0 #Intervention specific Time

x <- seq(0,12)
y <- sin(x*180/6*pi/180)
Beta1_time <- y*2*Beta1
Beta3_time <- y*1*Beta3
Time_factor <- seq(1,13)
Time_factor <- factor(Time_factor)

Patient_ID = NULL
Time = NULL
TimeInt = NULL
Intervention = NULL
HONOS_scores = NULL
Cluster_ID = NULL
Random_Error = NULL
Random_Effect = NULL
for (i in 1:NumCentre) {
  random_effect_clust <- rnorm(1,0,sigmac)
  for (k in 1:AverageClusterSize) {
    random_effect = rnorm(1,0,sigmab)
    for (j in 1:NSteps) {
      randomstart = match(i, random_centres) + 1
      patientnumber = (i - 1)*20 + k
      Patient_ID = c(Patient_ID, paste("Patient_",patientnumber, sep=""))
      Cluster_ID = c(Cluster_ID, paste("Cluster_",i, sep=""))
      time = j
      Time = c(Time, j)
      random_error = rnorm(1,0,sqrt((1-rho^2)*sigmae^2))
      if (j==1) {
        random_error_corr = random_error
      }
      if (j > 1) {
        random_error_corr = rho*Random_Error[(patientnumber-1)*NSteps + 1] + random_error
      }
      Random_Error = c(Random_Error, random_error_corr)
      Random_Effect = c(Random_Effect, random_effect)
      if (j < randomstart) {
        timeint = 0
```

```
          intervention = 0
        }
        else {
          timeint = j - randomstart + 1
          intervention = 1}
        TimeInt = c(TimeInt, timeint)
        Intervention = c(Intervention, intervention)
        HONOS = Beta0 + Beta1_time[match(factor(time),Time_factor)] + Beta2*intervention +
Beta3_time[match(factor(time),Time_factor)] + random_effect + random_error_corr
        HONOS_scores <- c(HONOS_scores, HONOS)
      }
    }
  }


    dataset <- cbind(Patient_ID, Cluster_ID, Intervention, Time, TimeInt, HONOS_scores, Random_Effect, Random_Error)
    dataset <- as.data.frame(dataset)
    write.csv(dataset, file = paste(file_loc,"Sim26_",N,".csv", sep =""))
  }
}
set.seed(12345)
Sim26(nSim)


##Simulation D33
Sim24 <- function(nSim) {
  for (N in 1:nSim){
    sigmab = 4.415
    sigmae = 5.442
    sigmac = 0.9570
    rho = -0.5

    NumCentre = 12
    NSteps = NumCentre + 1
    AverageClusterSize = 20


    centres = seq(1,NumCentre)
    random_centres = sample(centres, NumCentre, replace = FALSE)

    Beta0 <- 14.00 #Mean
    Beta1 <- 1 #Time
    Beta2 <- 2 #Intervention
```

```
Beta3 <- 1 #Intervention specific Time

x <- seq(0,12)
y <- sin(x*180/6*pi/180)
Beta1_time <- y*2*Beta1
Beta3_time <- y*1*Beta3
Time_factor <- seq(1,13)
Time_factor <- factor(Time_factor)

Patient_ID = NULL
Time = NULL
TimeInt = NULL
Intervention = NULL
HONOS_scores = NULL
Cluster_ID = NULL
Random_Error = NULL
Random_Effect = NULL
for (i in 1:NumCentre) {
  random_effect_clust <- rnorm(1,0,sigmac)
  for (k in 1:AverageClusterSize) {
    random_effect = rnorm(1,0,sigmab)
    for (j in 1:NSteps) {
      randomstart = match(i, random_centres) + 1
      patientnumber = (i - 1)*20 + k
      Patient_ID = c(Patient_ID, paste("Patient_",patientnumber, sep=""))
      Cluster_ID = c(Cluster_ID, paste("Cluster_",i, sep=""))
      time = j
      Time = c(Time, j)
      random_error = rnorm(1,0,sqrt((1-rho^2)*sigmae^2))
      if (j==1) {
        random_error_corr = random_error
      }
      if (j > 1) {
        random_error_corr = rho*Random_Error[(patientnumber-1)*NSteps + 1] + random_error
      }
      Random_Error = c(Random_Error, random_error_corr)
      Random_Effect = c(Random_Effect, random_effect)
      if (j < randomstart) {
        timeint = 0
        intervention = 0
      }
      else {
        timeint = j - randomstart + 1
```

```
            intervention = 1}
          TimeInt = c(TimeInt, timeint)
          Intervention = c(Intervention, intervention)
          HONOS = Beta0 + Beta1_time[match(factor(time),Time_factor)] + Beta2*intervention +
Beta3_time[match(factor(timeint),Time_factor)] + random_effect + random_error_corr
          HONOS_scores <- c(HONOS_scores, HONOS)
        }
      }
    }


    dataset <- cbind(Patient_ID, Cluster_ID, Intervention, Time, TimeInt, HONOS_scores, Random_Effect, Random_Error)
    dataset <- as.data.frame(dataset)
    write.csv(dataset, file = paste(file_loc,"Sim24_",N,".csv", sep =""))
  }
}
set.seed(12345)
Sim24(nSim)


##Simulation D34
Sim27 <- function(nSim) {
  for (N in 1:nSim){
    sigmab = 4.415
    sigmae = 5.442
    sigmac = 0.9570
    rho = 0.5

    NumCentre = 12
    NSteps = NumCentre + 1
    AverageClusterSize = 20


    centres = seq(1,NumCentre)
    random_centres = sample(centres, NumCentre, replace = FALSE)

    Beta0 <- 14.00 #Mean
    Beta1 <- 1 #Time
    Beta2 <- 2 #Intervention
    Beta3 <- 1 #Intervention specific Time

    x <- seq(0,12)
    y <- sin(x*180/6*pi/180)
```

```
Beta1_time <- y*2*Beta1
Beta3_time <- y*1*Beta3
Time_factor <- seq(1,13)
Time_factor <- factor(Time_factor)

Patient_ID = NULL
Time = NULL
TimeInt = NULL
Intervention = NULL
HONOS_scores = NULL
Cluster_ID = NULL
Random_Error = NULL
Random_Effect = NULL
for (i in 1:NumCentre) {
  random_effect_clust <- rnorm(1,0,sigmac)
  for (k in 1:AverageClusterSize) {
    random_effect = rnorm(1,0,sigmab)
    for (j in 1:NSteps) {
      randomstart = match(i, random_centres) + 1
      patientnumber = (i - 1)*20 + k
      Patient_ID = c(Patient_ID, paste("Patient_",patientnumber, sep=""))
      Cluster_ID = c(Cluster_ID, paste("Cluster_",i, sep=""))
      time = j
      Time = c(Time, j)
      random_error = rnorm(1,0,sqrt((1-rho^2)*sigmae^2))
      if (j==1) {
        random_error_corr = random_error
      }
      if (j > 1) {
        random_error_corr = rho*Random_Error[(patientnumber-1)*NSteps + 1] + random_error
      }
      Random_Error = c(Random_Error, random_error_corr)
      Random_Effect = c(Random_Effect, random_effect)
      if (j < randomstart) {
        timeint = 0
        intervention = 0
      }
      else {
        timeint = j - randomstart + 1
        intervention = 1}
      TimeInt = c(TimeInt, timeint)
      Intervention = c(Intervention, intervention)
```

```
          HONOS = Beta0 + Beta1_time[match(factor(time),Time_factor)] + Beta2*intervention +
Beta3_time[match(factor(timeint),Time_factor)] + random_effect + random_error_corr
          HONOS_scores <- c(HONOS_scores, HONOS)
        }
      }
    }


    dataset <- cbind(Patient_ID, Cluster_ID, Intervention, Time, TimeInt, HONOS_scores, Random_Effect, Random_Error)
    dataset <- as.data.frame(dataset)
    write.csv(dataset, file = paste(file_loc,"Sim27_",N,".csv", sep =""))
  }
}
set.seed(12345)
Sim27(nSim)


##Simulation D35
Sim25 <- function(nSim) {
  for (N in 1:nSim){
    sigmab = 4.415
    sigmae = 5.442
    sigmac = 0.9570
    rho = -0.5

    NumCentre = 12
    NSteps = NumCentre + 1
    AverageClusterSize = 20


    centres = seq(1,NumCentre)
    random_centres = sample(centres, NumCentre, replace = FALSE)

    Beta0 <- 14.00 #Mean
    Beta1 <- 1 #Time
    Beta2 <- 0 #Intervention
    Beta3 <- 1 #Intervention specific Time

    x <- seq(0,12)
    y <- sin(x*180/6*pi/180)
    Beta1_time <- y*2*Beta1
    Beta3_time <- y*1*Beta3
    Time_factor <- seq(1,13)
```

```
    Time_factor <- factor(Time_factor)

    Patient_ID = NULL
    Time = NULL
    TimeInt = NULL
    Intervention = NULL
    HONOS_scores = NULL
    Cluster_ID = NULL
    Random_Error = NULL
    Random_Effect = NULL
    for (i in 1:NumCentre) {
      random_effect_clust <- rnorm(1,0,sigmac)
      for (k in 1:AverageClusterSize) {
        random_effect = rnorm(1,0,sigmab)
        for (j in 1:NSteps) {
          randomstart = match(i, random_centres) + 1
          patientnumber = (i - 1)*20 + k
          Patient_ID = c(Patient_ID, paste("Patient_",patientnumber, sep=""))
          Cluster_ID = c(Cluster_ID, paste("Cluster_",i, sep=""))
          time = j
          Time = c(Time, j)
          random_error = rnorm(1,0,sqrt((1-rho^2)*sigmae^2))
          if (j==1) {
            random_error_corr = random_error
          }
          if (j > 1) {
            random_error_corr = rho*Random_Error[(patientnumber-1)*NSteps + 1] + random_error
          }
          Random_Error = c(Random_Error, random_error_corr)
          Random_Effect = c(Random_Effect, random_effect)
          if (j < randomstart) {
            timeint = 0
            intervention = 0
          }
          else {
            timeint = j - randomstart + 1
            intervention = 1}
          TimeInt = c(TimeInt, timeint)
          Intervention = c(Intervention, intervention)
          HONOS = Beta0 + Beta1_time[match(factor(time),Time_factor)] + Beta2*intervention +
Beta3_time[match(factor(timeint),Time_factor)] + random_effect + random_error_corr
          HONOS_scores <- c(HONOS_scores, HONOS)
        }
```

```
      }
    }


    dataset <- cbind(Patient_ID, Cluster_ID, Intervention, Time, TimeInt, HONOS_scores, Random_Effect, Random_Error)
    dataset <- as.data.frame(dataset)
    write.csv(dataset, file = paste(file_loc,"Sim25_",N,".csv", sep =""))
  }
}
set.seed(12345)
Sim25(nSim)


##Simulation D36
Sim28 <- function(nSim) {
  for (N in 1:nSim){
    sigmab = 4.415
    sigmae = 5.442
    sigmac = 0.9570
    rho = 0.5

    NumCentre = 12
    NSteps = NumCentre + 1
    AverageClusterSize = 20


    centres = seq(1,NumCentre)
    random_centres = sample(centres, NumCentre, replace = FALSE)

    Beta0 <- 14.00 #Mean
    Beta1 <- 1 #Time
    Beta2 <- 0 #Intervention
    Beta3 <- 1 #Intervention specific Time

    x <- seq(0,12)
    y <- sin(x*180/6*pi/180)
    Beta1_time <- y*2*Beta1
    Beta3_time <- y*1*Beta3
    Time_factor <- seq(1,13)
    Time_factor <- factor(Time_factor)

    Patient_ID = NULL
    Time = NULL
```

```
TimeInt = NULL
Intervention = NULL
HONOS_scores = NULL
Cluster_ID = NULL
Random_Error = NULL
Random_Effect = NULL
for (i in 1:NumCentre) {
  random_effect_clust <- rnorm(1,0,sigmac)
  for (k in 1:AverageClusterSize) {
    random_effect = rnorm(1,0,sigmab)
    for (j in 1:NSteps) {
      randomstart = match(i, random_centres) + 1
      patientnumber = (i - 1)*20 + k
      Patient_ID = c(Patient_ID, paste("Patient_",patientnumber, sep=""))
      Cluster_ID = c(Cluster_ID, paste("Cluster_",i, sep=""))
      time = j
      Time = c(Time, j)
      random_error = rnorm(1,0,sqrt((1-rho^2)*sigmae^2))
      if (j==1) {
        random_error_corr = random_error
      }
      if (j > 1) {
        random_error_corr = rho*Random_Error[(patientnumber-1)*NSteps + 1] + random_error
      }
      Random_Error = c(Random_Error, random_error_corr)
      Random_Effect = c(Random_Effect, random_effect)
      if (j < randomstart) {
        timeint = 0
        intervention = 0
      }
      else {
        timeint = j - randomstart + 1
        intervention = 1}
      TimeInt = c(TimeInt, timeint)
      Intervention = c(Intervention, intervention)
      HONOS = Beta0 + Beta1_time[match(factor(time),Time_factor)] + Beta2*intervention +
Beta3_time[match(factor(timeint),Time_factor)] + random_effect + random_error_corr
      HONOS_scores <- c(HONOS_scores, HONOS)
    }
  }
}
```

```
    dataset <- cbind(Patient_ID, Cluster_ID, Intervention, Time, TimeInt, HONOS_scores, Random_Effect, Random_Error)
    dataset <- as.data.frame(dataset)
    write.csv(dataset, file = paste(file_loc,"Sim28_",N,".csv", sep =""))
  }
}
set.seed(12345)
Sim28(nSim)
```
