# Supplementary material for: Mixed effects approach to the analysis of the stepped wedge cluster randomised trial—Investigating the confounding effect of time through simulation
Source: PLoS One. 2018 Dec 13;13(12):e0208876. doi: 10.1371/journal.pone.0208876 (PMC6292598; doi:10.1371/journal.pone.0208876)
Supplement: S2 Appendix — (PDF) [file pone.0208876.s002.pdf]

**S2 Appendix. Code to fit linear mixed effects models**

```
##Alecia Nickless
#29 May 2018
#Code to fit linear mixed effects models to simulated datasets

require(nlme)
require(MASS)
require(multcomp)

#Code to fit models in R and collect data
setwd("file path")
files <- list.files(path="file path/Sim_Datasets/", pattern="*.csv", full.names=F, recursive=FALSE)

Simulations <- c("0", "00", "000", "0000", "00000", "000000", "0000000", "00000000", seq(1,28,1))
Repeats <- 1000

nSim <- 36 #24 #This is the number of different models used to simulate data
nModels <- 18 #18 #This is the number of different models fitted to the data
Sim_fit_results <- matrix(rep(NA, length = nModels*nSim*Repeats*36), nrow = nModels*nSim*Repeats, ncol = 36)
Sim_fit_results <- as.data.frame(Sim_fit_results)
names(Sim_fit_results) <- c("Model", "Simulation", "Repeat", "AIC", "BIC", "MSE", "Intercept", "Intercep_SE",
"Intercept_p", "Intercep_t3p",
                            "Time", "Time_SE", "Time_p", "Time_t3p", "Intervention", "Intervention_SE",
"Intervention_p",
                            "Intervention_t3p", "Interven_time", "Interven_time_SE", "Interven_time_p",
"Intver_time_t3p",
                            "Exptime", "Exptime_SE", "Exptime_p", "Exptime_t3p", "Timesq", "Timesq_SE", "Timesq_p",
"Estimate", "Estimate_SE", "Estimate_p", "Estimate2", "Estimate2_SE", "Estimate2_p", "Cov_var")
#i = repeated dataset 1 to 1000
#j = model fitted 1 to 20 (1-10 is CS and 11-20 is AR(1))
#Looping through the different repeats

for (k in 1:length(Simulations)) {
  print(k)
  #Fitted Model 1
  for (i in 1:Repeats) {
    j=1
    cov_var <- "cs"
```

```
    data <- read.csv(paste("Sim",Simulations[k],"_",i,".csv", sep =""))
    data$Intervention <- as.factor(data$Intervention)
    fm1 <- try(lme(HONOS_scores ~ Intervention, random = ~ 1 | Cluster_ID/Patient_ID, data = data, method = "ML",
control=lmeControl(returnObject=TRUE, maxIter=100)), TRUE)
    if(isTRUE(class(fm1)=="try-error")) { next } else { fm1=fm1 }
    K <- matrix(c(0,1), 1)
    t <- glht(fm1, linfct = K)
    K2 <- matrix(c(0,1), 1)
    t2 <- glht(fm1, linfct = K2)
    #Getting AIC
    AIC <- AIC(fm1)
    BIC <- BIC(fm1)
    MSE <- fm1$sigma
    #Intercept
    intercept <- summary(fm1)$tTable[1,1]
    inter_SE <- summary(fm1)$tTable[1,2]
    inter_p <- summary(fm1)$tTable[1,5]
    inter_t3p <- anova(fm1)[1,4]
    #Intervention
    interven <- summary(fm1)$tTable[2,1]
    interven_SE <- summary(fm1)$tTable[2,2]
    interven_p <- summary(fm1)$tTable[2,5]
    interven_t3p <- anova(fm1)[2,4]
    #Estimate of effect at 6 months
    estimate <- summary(t)$test$coefficients[1]
    estimate_se <- summary(t)$test$sigma[1]
    estimate_p <- summary(t)$test$pvalues[1]
    #Estimate of time-averaged effect
    estimate2 <- t2[1,2]
    estimate2_se <- t2[1,3]
    estimate2_p <- t2[1,5]
    #Writing out results
    Sim_fit_results[(j-1)*nSim*Repeats + (k-1)*Repeats + i,c(1,2,3,4,5,6,7,8,9,10,15,16,17,18,30,31,32,33,34,35,36)]
<- c(j,Simulations[k],i,AIC,BIC,MSE, intercept, inter_SE, inter_p, inter_t3p, interven, interven_SE, interven_p,
interven_t3p, estimate, estimate_se, estimate_p, estimate2, estimate2_se, estimate2_p, cov_var)
  }
  #Fitted Model 2
  for (i in 1:Repeats) {
    j=2
```

```
    cov_var <- "cs"
    data <- read.csv(paste("Sim",Simulations[k],"_",i,".csv", sep =""))
    data$Intervention <- as.factor(data$Intervention)
    fm1 <- try(lme(HONOS_scores ~ Time + Intervention, random = ~ 1 | Cluster_ID/Patient_ID, data = data, method =
"ML", control=lmeControl(returnObject=TRUE, maxIter=100)), TRUE)
    if(isTRUE(class(fm1)=="try-error")) { next } else { fm1=fm1 }
    K <- matrix(c(0,0,1), 1)
    t <- glht(fm1, linfct = K)
    K2 <- matrix(c(0,0,1), 1)
    t2 <- glht(fm1, linfct = K2)
    #Getting AIC
    AIC <- AIC(fm1)
    BIC <- BIC(fm1)
    MSE <- fm1$sigma
    #Intercept
    intercept <- summary(fm1)$tTable[1,1]
    inter_SE <- summary(fm1)$tTable[1,2]
    inter_p <- summary(fm1)$tTable[1,5]
    inter_t3p <- anova(fm1)[1,4]
    #Time
    time <- summary(fm1)$tTable[2,1]
    time_SE <- summary(fm1)$tTable[2,2]
    time_p <- summary(fm1)$tTable[2,5]
    time_t3p <- anova(fm1)[2,4]
    #Intervention
    interven <- summary(fm1)$tTable[3,1]
    interven_SE <- summary(fm1)$tTable[3,2]
    interven_p <- summary(fm1)$tTable[3,5]
    interven_t3p <- anova(fm1)[3,4]
    #Estimate of effect at 6 months
    estimate <- summary(t)$test$coefficients[1]
    estimate_se <- summary(t)$test$sigma[1]
    estimate_p <- summary(t)$test$pvalues[1]
    #Estimate of time-averaged effect
    estimate2 <- summary(t2)$test$coefficients[1]
    estimate2_se <- summary(t2)$test$sigma[1]
    estimate2_p <- summary(t2)$test$pvalues[1]
    #Writing out results
```

```
    Sim_fit_results[(j-1)*nSim*Repeats + (k-1)*Repeats +
i,c(1,2,3,4,5,6,7,8,9,10,11,12,13,14,15,16,17,18,30,31,32,33,34,35,36)] <- c(j,Simulations[k],i,AIC,BIC,MSE,
intercept, inter_SE, inter_p, inter_t3p, time, time_SE, time_p, time_t3p, interven, interven_SE, interven_p,
interven_t3p, estimate, estimate_se, estimate_p, estimate2, estimate2_se, estimate2_p, cov_var)
  }
  #Fitted Model 3
  for (i in 1:Repeats) {
    j=3
    cov_var <- "cs"
    data <- read.csv(paste("Sim",Simulations[k],"_",i,".csv", sep =""))
    data$Intervention <- as.factor(data$Intervention)
    data$Time <- as.factor(data$Time)
    fm1 <- try(lme(HONOS_scores ~ Time + Intervention, random = ~ 1 | Cluster_ID/Patient_ID, data = data, method =
"ML", control=lmeControl(returnObject=TRUE, maxIter=100)), TRUE)
    if(isTRUE(class(fm1)=="try-error")) { next } else { fm1=fm1 }
    K <- matrix(c(0,0,0,0,0,0,0,0,0,0,0,0,0,1), 1)
    t <- glht(fm1, linfct = K)
    K2 <- matrix(c(0,0,0,0,0,0,0,0,0,0,0,0,0,1), 1)
    t2 <- glht(fm1, linfct = K2)
    #Getting AIC
    AIC <- AIC(fm1)
    BIC <- BIC(fm1)
    MSE <- fm1$sigma
    #Intercept
    intercept <- summary(fm1)$tTable[1,1]
    inter_SE <- summary(fm1)$tTable[1,2]
    inter_p <- summary(fm1)$tTable[1,5]
    inter_t3p <- anova(fm1)[1,4]
    #Time
    time <- NA
    time_SE <- NA
    time_p <- NA
    time_t3p <- anova(fm1)[2,4]
    #Intervention
    interven <- summary(fm1)$tTable[14,1]
    interven_SE <- summary(fm1)$tTable[14,2]
    interven_p <- summary(fm1)$tTable[14,5]
    interven_t3p <- anova(fm1)[3,4]
    #Estimate of effect at 6 months
```

```
    estimate <- summary(t)$test$coefficients[1]
    estimate_se <- summary(t)$test$sigma[1]
    estimate_p <- summary(t)$test$pvalues[1]
    #Estimate of time-averaged effect
    estimate2 <- summary(t2)$test$coefficients[1]
    estimate2_se <- summary(t2)$test$sigma[1]
    estimate2_p <- summary(t2)$test$pvalues[1]
    #Writing out results
    Sim_fit_results[(j-1)*nSim*Repeats + (k-1)*Repeats +
i,c(1,2,3,4,5,6,7,8,9,10,11,12,13,14,15,16,17,18,30,31,32,33,34,35,36)] <- c(j,Simulations[k],i,AIC,BIC,MSE,
intercept, inter_SE, inter_p, inter_t3p, time, time_SE, time_p, time_t3p, interven, interven_SE, interven_p,
interven_t3p, estimate, estimate_se, estimate_p, estimate2, estimate2_se, estimate2_p, cov_var)
  }
  #Fitted Model 4
  for (i in 1:Repeats) {
    j=4
    cov_var <- "cs"
    data <- read.csv(paste("Sim",Simulations[k],"_",i,".csv", sep =""))
    data$Intervention <- as.factor(data$Intervention)
    fm1 <- try(lme(HONOS_scores ~ Time + Intervention + Time*Intervention, random = ~ 1 | Cluster_ID/Patient_ID,
data = data, method = "ML", control=lmeControl(returnObject=TRUE, maxIter=100)), TRUE)
    if(isTRUE(class(fm1)=="try-error")) { next } else { fm1=fm1 }
    K <- matrix(c(0,0,1,6), 1)
    t <- glht(fm1, linfct = K)
    K2 <- matrix(c(0,0,1,7), 1)
    t2 <- glht(fm1, linfct = K2)
    #Getting AIC
    AIC <- AIC(fm1)
    BIC <- BIC(fm1)
    MSE <- fm1$sigma
    #Intercept
    intercept <- summary(fm1)$tTable[1,1]
    inter_SE <- summary(fm1)$tTable[1,2]
    inter_p <- summary(fm1)$tTable[1,5]
    inter_t3p <- anova(fm1)[1,4]
    #Time
    time <- summary(fm1)$tTable[2,1]
    time_SE <- summary(fm1)$tTable[2,2]
    time_p <- summary(fm1)$tTable[2,5]
```

```
    time_t3p <- anova(fm1)[2,4]
    #Intervention
    interven <- summary(fm1)$tTable[3,1]
    interven_SE <- summary(fm1)$tTable[3,2]
    interven_p <- summary(fm1)$tTable[3,5]
    interven_t3p <- anova(fm1)[3,4]
    #Time*Intervention
    time_int <- summary(fm1)$tTable[4,1]
    time_int_SE <- summary(fm1)$tTable[4,2]
    time_int_p <- summary(fm1)$tTable[4,5]
    time_int_t3p <- anova(fm1)[4,4]
    #Estimate of effect at 6 months
    estimate <- summary(t)$test$coefficients[1]
    estimate_se <- summary(t)$test$sigma[1]
    estimate_p <- summary(t)$test$pvalues[1]
    #Estimate of time-averaged effect
    estimate2 <- summary(t2)$test$coefficients[1]
    estimate2_se <- summary(t2)$test$sigma[1]
    estimate2_p <- summary(t2)$test$pvalues[1]
    #Writing out results
    Sim_fit_results[(j-1)*nSim*Repeats + (k-1)*Repeats + 
i,c(1,2,3,4,5,6,7,8,9,10,11,12,13,14,15,16,17,18,19,20,21,22,30,31,32,33,34,35,36)] <- 
c(j,Simulations[k],i,AIC,BIC,MSE, intercept, inter_SE, inter_p, inter_t3p, time, time_SE, time_p, time_t3p, 
interven, interven_SE, interven_p, interven_t3p, time_int, time_int_SE, time_int_p, time_int_t3p, estimate, 
estimate_se, estimate_p, estimate2, estimate2_se, estimate2_p, cov_var)
  }
  #Fitted Model 5
  for (i in 1:Repeats) {
    j=5
    cov_var <- "cs"
    data <- read.csv(paste("Sim",Simulations[k],"_",i,".csv", sep =""))
    data$Intervention <- as.factor(data$Intervention)
    data$Time <- as.factor(data$Time)
    data$Time_Int <- "IntTime0"
    data$Time_Int[data$Time == "3" & data$Intervention == "1"] <- "IntTime3"
    data$Time_Int[data$Time == "4" & data$Intervention == "1"] <- "IntTime4"
    data$Time_Int[data$Time == "5" & data$Intervention == "1"] <- "IntTime5"
    data$Time_Int[data$Time == "6" & data$Intervention == "1"] <- "IntTime6"
    data$Time_Int[data$Time == "7" & data$Intervention == "1"] <- "IntTime7"
```

```
    data$Time_Int[data$Time == "8" & data$Intervention == "1"] <- "IntTime8"
    data$Time_Int[data$Time == "9" & data$Intervention == "1"] <- "IntTime9"
    data$Time_Int[data$Time == "10" & data$Intervention == "1"] <- "IntTime10"
    data$Time_Int[data$Time == "11" & data$Intervention == "1"] <- "IntTime11"
    data$Time_Int[data$Time == "12" & data$Intervention == "1"] <- "IntTime12"
    data$Time_Int <- as.factor(data$Time_Int)
    fm1 <- try(lme(HONOS_scores ~ Time + Intervention + Time_Int, random = ~ 1 | Cluster_ID/Patient_ID, data = data, 
method = "ML", control=lmeControl(returnObject=TRUE, maxIter=100)), TRUE)
    if(isTRUE(class(fm1)=="try-error")) { next } else { fm1=fm1 }
    K <- matrix(c(0,0,0,0,0,0,0,0,0,0,0,0,0,1,0,0,0,0,0,0,1,0,0,0), 1)
    t <- glht(fm1, linfct = K)
    K2 <- matrix(c(0,0,0,0,0,0,0,0,0,0,0,0,0,1,0.1,0.1,0.1,0.1,0.1,0.1,0.1,0.1,0.1,0.1), 1)
    t2 <- glht(fm1, linfct = K2)
    #Getting AIC
    AIC <- AIC(fm1)
    BIC <- BIC(fm1)
    MSE <- fm1$sigma
    #Intercept
    intercept <- summary(fm1)$tTable[1,1]
    inter_SE <- summary(fm1)$tTable[1,2]
    inter_p <- summary(fm1)$tTable[1,5]
    inter_t3p <- anova(fm1)[1,4]
    #Time
    time <- NA
    time_SE <- NA
    time_p <- NA
    time_t3p <- anova(fm1)[2,4]
    #Intervention
    interven <- summary(fm1)$tTable[14,1]
    interven_SE <- summary(fm1)$tTable[14,2]
    interven_p <- summary(fm1)$tTable[14,5]
    interven_t3p <- anova(fm1)[3,4]
    #Time*Intervention
    time_int <- NA
    time_int_SE <- NA
    time_int_p <- NA
    time_int_t3p <- anova(fm1)[4,4]
    #Estimate of effect at 6 months
    estimate <- summary(t)$test$coefficients[1]
```

```
    estimate_se <- summary(t)$test$sigma[1]
    estimate_p <- summary(t)$test$pvalues[1]
    #Estimate of time-averaged effect
    estimate2 <- summary(t2)$test$coefficients[1]
    estimate2_se <- summary(t2)$test$sigma[1]
    estimate2_p <- summary(t2)$test$pvalues[1]
    #Writing out results
    Sim_fit_results[(j-1)*nSim*Repeats + (k-1)*Repeats +
i,c(1,2,3,4,5,6,7,8,9,10,11,12,13,14,15,16,17,18,19,20,21,22,30,31,32,33,34,35,36)] <-
c(j,Simulations[k],i,AIC,BIC,MSE, intercept, inter_SE, inter_p, inter_t3p, time, time_SE, time_p, time_t3p,
interven, interven_SE, interven_p, interven_t3p, time_int, time_int_SE, time_int_p, time_int_t3p, estimate,
estimate_se, estimate_p, estimate2, estimate2_se, estimate2_p, cov_var)
  }
  #Fitted Model 6
  for (i in 1:Repeats) {
    j=6
    cov_var <- "cs"
    data <- read.csv(paste("Sim",Simulations[k],"_",i,".csv", sep =""))
    data$Intervention <- as.factor(data$Intervention)
    fm1 <- try(lme(HONOS_scores ~ Time + Intervention + TimeInt, random = ~ 1 | Cluster_ID/Patient_ID, data = data,
method = "ML", control=lmeControl(returnObject=TRUE, maxIter=100)), TRUE)
    if(isTRUE(class(fm1)=="try-error")) { next } else { fm1=fm1 }
    K <- matrix(c(0, 0, 1, 6), 1)
    t <- glht(fm1, linfct = K)
    K2 <- matrix(c(0, 0, 1, 6), 1)
    t2 <- glht(fm1, linfct = K2)
    #Getting AIC
    AIC <- AIC(fm1)
    BIC <- BIC(fm1)
    MSE <- fm1$sigma
    #Intercept
    intercept <- summary(fm1)$tTable[1,1]
    inter_SE <- summary(fm1)$tTable[1,2]
    inter_p <- summary(fm1)$tTable[1,5]
    inter_t3p <- anova(fm1)[1,4]
    #Time
    time <- summary(fm1)$tTable[2,1]
    time_SE <- summary(fm1)$tTable[2,2]
    time_p <- summary(fm1)$tTable[2,5]
```

```
    time_t3p <- anova(fm1)[2,4]
    #Intervention
    interven <- summary(fm1)$tTable[3,1]
    interven_SE <- summary(fm1)$tTable[3,2]
    interven_p <- summary(fm1)$tTable[3,5]
    interven_t3p <- anova(fm1)[3,4]
    #Time*Intervention
    timeint <- summary(fm1)$tTable[4,1]
    timeint_SE <- summary(fm1)$tTable[4,2]
    timeint_p <- summary(fm1)$tTable[4,5]
    timeint_t3p <- anova(fm1)[4,4]
    #Estimate of effect at 6 months
    estimate <- summary(t)$test$coefficients[1]
    estimate_se <- summary(t)$test$sigma[1]
    estimate_p <- summary(t)$test$pvalues[1]
    #Estimate of time-averaged effect
    estimate2 <- summary(t2)$test$coefficients[1]
    estimate2_se <- summary(t2)$test$sigma[1]
    estimate2_p <- summary(t2)$test$pvalues[1]
    #Writing out results
    Sim_fit_results[(j-1)*nSim*Repeats + (k-1)*Repeats +
i,c(1,2,3,4,5,6,7,8,9,10,11,12,13,14,15,16,17,18,23,24,25,26,30,31,32,33,34,35,36)] <-
c(j,Simulations[k],i,AIC,BIC,MSE, intercept, inter_SE, inter_p, inter_t3p, time, time_SE, time_p, time_t3p,
interven, interven_SE, interven_p, interven_t3p, timeint, timeint_SE, timeint_p, timeint_t3p, estimate, estimate_se,
estimate_p, estimate2, estimate2_se, estimate2_p, cov_var)
  }
  #Fitted Model 7
  for (i in 1:Repeats) {
    j=7
    cov_var <- "cs"
    data <- read.csv(paste("Sim",Simulations[k],"_",i,".csv", sep =""))
    data$Intervention <- as.factor(data$Intervention)
    fm1 <- try(lme(HONOS_scores ~ Time + TimeInt, random = ~ 1 | Cluster_ID/Patient_ID, data = data, method = "ML",
control=lmeControl(returnObject=TRUE, maxIter=100)), TRUE)
    if(isTRUE(class(fm1)=="try-error")) { next } else { fm1=fm1 }
    K <- matrix(c(0, 0, 6), 1)
    t <- glht(fm1, linfct = K)
    K2 <- matrix(c(0, 0, 6), 1)
    t2 <- glht(fm1, linfct = K2)
```

```
    #Getting AIC
     AIC <- AIC(fm1)
     BIC <- BIC(fm1)
     MSE <- fm1$sigma
     #Intercept
     intercept <- summary(fm1)$tTable[1,1]
     inter_SE <- summary(fm1)$tTable[1,2]
     inter_p <- summary(fm1)$tTable[1,5]
     inter_t3p <- anova(fm1)[1,4]
     #Time
     time <- summary(fm1)$tTable[2,1]
     time_SE <- summary(fm1)$tTable[2,2]
     time_p <- summary(fm1)$tTable[2,5]
     time_t3p <- anova(fm1)[2,4]
     #Exposure Time
     timeint <- summary(fm1)$tTable[3,1]
     timeint_SE <- summary(fm1)$tTable[3,2]
     timeint_p <- summary(fm1)$tTable[3,5]
     timeint_t3p <- anova(fm1)[3,4]
     #Estimate of effect at 6 months
     estimate <- summary(t)$test$coefficients[1]
     estimate_se <- summary(t)$test$sigma[1]
     estimate_p <- summary(t)$test$pvalues[1]
     #Estimate of time-averaged effect
     estimate2 <- summary(t2)$test$coefficients[1]
     estimate2_se <- summary(t2)$test$sigma[1]
     estimate2_p <- summary(t2)$test$pvalues[1]
     #Writing out results
     Sim_fit_results[(j-1)*nSim*Repeats + (k-1)*Repeats +
i,c(1,2,3,4,5,6,7,8,9,10,11,12,13,14,23,24,25,26,30,31,32,33,34,35,36)] <- c(j,Simulations[k],i,AIC,BIC,MSE,
intercept, inter_SE, inter_p, inter_t3p, time, time_SE, time_p, time_t3p, timeint, timeint_SE, timeint_p,
timeint_t3p, estimate, estimate_se, estimate_p, estimate2, estimate2_se, estimate2_p, cov_var)
   }
   #Fitted Model 8
   for (i in 1:Repeats) {
     j=8
     cov_var <- "cs"
     data <- read.csv(paste("Sim",Simulations[k],"_",i,".csv", sep =""))
     data$Intervention <- as.factor(data$Intervention)
```

```
    data$Time <- as.factor(data$Time)
    data$TimeInt <- as.factor(data$TimeInt)
    fm1 <- try(lme(HONOS_scores ~ Time + TimeInt, random = ~ 1 | Cluster_ID/Patient_ID, data = data, method = "ML",
control=lmeControl(returnObject=TRUE, maxIter=100)), TRUE)
    if(isTRUE(class(fm1)=="try-error")) { next } else { fm1=fm1 }
    K <- matrix(c(0,0,0,0,0,0,0,0,0,0,0,0,0,0,0,0,0,0,1,0,0,0,0,0,0), 1)
    t <- glht(fm1, linfct = K)
    K2 <-
matrix(c(0,0,0,0,0,0,0,0,0,0,0,0,0,0.08333333,0.08333333,0.08333333,0.08333333,0.08333333,0.08333333,0.08333333,0.08
333333,0.08333333,0.08333333,0.08333333,0.08333333), 1)
    t2 <- glht(fm1, linfct = K2)
    #Getting AIC
    AIC <- AIC(fm1)
    BIC <- BIC(fm1)
    MSE <- fm1$sigma
    #Intercept
    intercept <- summary(fm1)$tTable[1,1]
    inter_SE <- summary(fm1)$tTable[1,2]
    inter_p <- summary(fm1)$tTable[1,5]
    inter_t3p <- anova(fm1)[1,4]
    #Time
    time <- NA
    time_SE <- NA
    time_p <- NA
    time_t3p <- anova(fm1)[1,4]
    #Exposure Time
    timeint <- NA
    timeint_SE <- NA
    timeint_p <- NA
    timeint_t3p <- anova(fm1)[2,4]
    #Estimate of effect at 6 months
    estimate <- summary(t)$test$coefficients[1]
    estimate_se <- summary(t)$test$sigma[1]
    estimate_p <- summary(t)$test$pvalues[1]
    #Estimate of time-averaged effect
    estimate2 <- summary(t2)$test$coefficients[1]
    estimate2_se <- summary(t2)$test$sigma[1]
    estimate2_p <- summary(t2)$test$pvalues[1]
    #Writing out results
```

```
    Sim_fit_results[(j-1)*nSim*Repeats + (k-1)*Repeats +
i,c(1,2,3,4,5,6,7,8,9,10,11,12,13,14,23,24,25,26,30,31,32,33,34,35,36)] <- c(j,Simulations[k],i,AIC,BIC,MSE,
intercept, inter_SE, inter_p, inter_t3p, time, time_SE, time_p, time_t3p, timeint, timeint_SE, timeint_p,
timeint_t3p, estimate, estimate_se, estimate_p, estimate2, estimate2_se, estimate2_p, cov_var)
  }
  #Fitted Model 9
  for (i in 1:Repeats) {
    j=9
    cov_var <- "cs"
    data <- read.csv(paste("Sim",Simulations[k],"_",i,".csv", sep =""))
    data$Intervention <- as.factor(data$Intervention)
    fm1 <- try(lme(HONOS_scores ~ Time + Intervention + TimeInt + I(Time^2), random = ~ 1 | Cluster_ID/Patient_ID,
data = data, method = "ML", control=lmeControl(returnObject=TRUE, maxIter=100)), TRUE)
    if(isTRUE(class(fm1)=="try-error")) { next } else { fm1=fm1 }
    K <- matrix(c(0, 0, 1, 6, 0), 1)
    t <- glht(fm1, linfct = K)
    K2 <- matrix(c(0, 0, 1, 6, 0), 1)
    t2 <- glht(fm1, linfct = K2)
    #Getting AIC
    AIC <- AIC(fm1)
    BIC <- BIC(fm1)
    MSE <- fm1$sigma
    #Intercept
    intercept <- summary(fm1)$tTable[1,1]
    inter_SE <- summary(fm1)$tTable[1,2]
    inter_p <- summary(fm1)$tTable[1,5]
    inter_t3p <- anova(fm1)[1,4]
    #Time
    time <- summary(fm1)$tTable[2,1]
    time_SE <- summary(fm1)$tTable[2,2]
    time_p <- summary(fm1)$tTable[2,5]
    time_t3p <- anova(fm1)[2,4]
    #Intervention
    interven <- summary(fm1)$tTable[3,1]
    interven_SE <- summary(fm1)$tTable[3,2]
    interven_p <- summary(fm1)$tTable[3,5]
    interven_t3p <- anova(fm1)[3,4]
    #Time*Intervention
    timeint <- summary(fm1)$tTable[4,1]
```

```
    timeint_SE <- summary(fm1)$tTable[4,2]
    timeint_p <- summary(fm1)$tTable[4,5]
    timeint_t3p <- anova(fm1)[4,4]
    #Timesq
    timesq <- summary(fm1)$tTable[5,1]
    timesq_SE <- summary(fm1)$tTable[5,2]
    timesq_p <- summary(fm1)$tTable[5,5]
    timesq_t3p <- anova(fm1)[5,4]
    #Estimate of effect at 6 months
    estimate <- summary(t)$test$coefficients[1]
    estimate_se <- summary(t)$test$sigma[1]
    estimate_p <- summary(t)$test$pvalues[1]
    #Estimate of time-averaged effect
    estimate2 <- summary(t2)$test$coefficients[1]
    estimate2_se <- summary(t2)$test$sigma[1]
    estimate2_p <- summary(t2)$test$pvalues[1]
    #Writing out results
    Sim_fit_results[(j-1)*nSim*Repeats + (k-1)*Repeats +
i,c(1,2,3,4,5,6,7,8,9,10,11,12,13,14,15,16,17,18,23,24,25,26,27,28,29,30,31,32,33,34,35,36)] <-
c(j,Simulations[k],i,AIC,BIC,MSE, intercept, inter_SE, inter_p, inter_t3p, time, time_SE, time_p, time_t3p,
interven, interven_SE, interven_p, interven_t3p, timeint, timeint_SE, timeint_p, timeint_t3p, timesq, timesq_SE,
timesq_p, estimate, estimate_se, estimate_p, estimate2, estimate2_se, estimate2_p, cov_var)
  }
##AR(1)
  #Fitted Model 1
  for (i in 1:Repeats) {
    j=10
    cov_var <- "ar1"
    data <- read.csv(paste("Sim",Simulations[k],"_",i,".csv", sep =""))
    data$Intervention <- as.factor(data$Intervention)
    fm1 <- try(lme(HONOS_scores ~ Intervention, random = ~ 1 | Cluster_ID/Patient_ID, data = data, method = "ML",
correlation = corAR1(), control=lmeControl(returnObject=TRUE, maxIter=100)), TRUE)
    if(isTRUE(class(fm1)=="try-error")) { next } else { fm1=fm1 }
    K <- matrix(c(0,1), 1)
    t <- glht(fm1, linfct = K)
    K2 <- matrix(c(0,1), 1)
    t2 <- glht(fm1, linfct = K2)
    #Getting AIC
    AIC <- AIC(fm1)
```

```
    BIC <- BIC(fm1)
    MSE <- fm1$sigma
    #Intercept
    intercept <- summary(fm1)$tTable[1,1]
    inter_SE <- summary(fm1)$tTable[1,2]
    inter_p <- summary(fm1)$tTable[1,5]
    inter_t3p <- anova(fm1)[1,4]
    #Intervention
    interven <- summary(fm1)$tTable[2,1]
    interven_SE <- summary(fm1)$tTable[2,2]
    interven_p <- summary(fm1)$tTable[2,5]
    interven_t3p <- anova(fm1)[2,4]
    #Estimate of effect at 6 months
    estimate <- summary(t)$test$coefficients[1]
    estimate_se <- summary(t)$test$sigma[1]
    estimate_p <- summary(t)$test$pvalues[1]
    #Estimate of time-averaged effect
    estimate2 <- summary(t2)$test$coefficients[1]
    estimate2_se <- summary(t2)$test$sigma[1]
    estimate2_p <- summary(t2)$test$pvalues[1]
    #Writing out results
    Sim_fit_results[(j-1)*nSim*Repeats + (k-1)*Repeats + i,c(1,2,3,4,5,6,7,8,9,10,15,16,17,18,30,31,32,33,34,35,36)]
<- c(j,Simulations[k],i,AIC,BIC,MSE, intercept, inter_SE, inter_p, inter_t3p, interven, interven_SE, interven_p,
interven_t3p, estimate, estimate_se, estimate_p, estimate2, estimate2_se, estimate2_p, cov_var)
  }
  #Fitted Model 2
  for (i in 1:Repeats) {
    j=11
    cov_var <- "ar1"
    data <- read.csv(paste("Sim",Simulations[k],"_",i,".csv", sep =""))
    data$Intervention <- as.factor(data$Intervention)
    fm1 <- try(lme(HONOS_scores ~ Time + Intervention, random = ~ 1 | Cluster_ID/Patient_ID, data = data, method =
"ML", correlation = corAR1(), control=lmeControl(returnObject=TRUE, maxIter=100)), TRUE)
    if(isTRUE(class(fm1)=="try-error")) { next } else { fm1=fm1 }
    K <- matrix(c(0,0,1), 1)
    t <- glht(fm1, linfct = K)
    K2 <- matrix(c(0,0,1), 1)
    t2 <- glht(fm1, linfct = K2)
    #Getting AIC
```

```
    AIC <- AIC(fm1)
    BIC <- BIC(fm1)
    MSE <- fm1$sigma
    #Intercept
    intercept <- summary(fm1)$tTable[1,1]
    inter_SE <- summary(fm1)$tTable[1,2]
    inter_p <- summary(fm1)$tTable[1,5]
    inter_t3p <- anova(fm1)[1,4]
    #Time
    time <- summary(fm1)$tTable[2,1]
    time_SE <- summary(fm1)$tTable[2,2]
    time_p <- summary(fm1)$tTable[2,5]
    time_t3p <- anova(fm1)[2,4]
    #Intervention
    interven <- summary(fm1)$tTable[3,1]
    interven_SE <- summary(fm1)$tTable[3,2]
    interven_p <- summary(fm1)$tTable[3,5]
    interven_t3p <- anova(fm1)[3,4]
    #Estimate of effect at 6 months
    estimate <- summary(t)$test$coefficients[1]
    estimate_se <- summary(t)$test$sigma[1]
    estimate_p <- summary(t)$test$pvalues[1]
    #Estimate of time-averaged effect
    estimate2 <- summary(t2)$test$coefficients[1]
    estimate2_se <- summary(t2)$test$sigma[1]
    estimate2_p <- summary(t2)$test$pvalues[1]
    #Writing out results
    Sim_fit_results[(j-1)*nSim*Repeats + (k-1)*Repeats +
i,c(1,2,3,4,5,6,7,8,9,10,11,12,13,14,15,16,17,18,30,31,32,33,34,35,36)] <- c(j,Simulations[k],i,AIC,BIC,MSE,
intercept, inter_SE, inter_p, inter_t3p, time, time_SE, time_p, time_t3p, interven, interven_SE, interven_p,
interven_t3p, estimate, estimate_se, estimate_p, estimate2, estimate2_se, estimate2_p, cov_var)
  }
  #Fitted Model 3
  for (i in 1:Repeats) {
    j=12
    cov_var <- "ar1"
    data <- read.csv(paste("Sim",Simulations[k],"_",i,".csv", sep =""))
    data$Intervention <- as.factor(data$Intervention)
    data$Time <- as.factor(data$Time)
```

```
    fm1 <- try(lme(HONOS_scores ~ Time + Intervention, random = ~ 1 | Cluster_ID/Patient_ID, data = data, method = 
"ML", correlation = corAR1(), control=lmeControl(returnObject=TRUE, maxIter=100)), TRUE)
    if(isTRUE(class(fm1)=="try-error")) { next } else { fm1=fm1 }
    K <- matrix(c(0,0,0,0,0,0,0,0,0,0,0,0,0,1), 1)
    t <- glht(fm1, linfct = K)
    K2 <- matrix(c(0,0,0,0,0,0,0,0,0,0,0,0,0,1), 1)
    t2 <- glht(fm1, linfct = K2)
    #Getting AIC
    AIC <- AIC(fm1)
    BIC <- BIC(fm1)
    MSE <- fm1$sigma
    #Intercept
    intercept <- summary(fm1)$tTable[1,1]
    inter_SE <- summary(fm1)$tTable[1,2]
    inter_p <- summary(fm1)$tTable[1,5]
    inter_t3p <- anova(fm1)[1,4]
    #Time
    time <- NA
    time_SE <- NA
    time_p <- NA
    time_t3p <- anova(fm1)[2,4]
    #Intervention
    interven <- summary(fm1)$tTable[14,1]
    interven_SE <- summary(fm1)$tTable[14,2]
    interven_p <- summary(fm1)$tTable[14,5]
    interven_t3p <- anova(fm1)[3,4]
    #Estimate of effect at 6 months
    estimate <- summary(t)$test$coefficients[1]
    estimate_se <- summary(t)$test$sigma[1]
    estimate_p <- summary(t)$test$pvalues[1]
    #Estimate of time-averaged effect
    estimate2 <- summary(t2)$test$coefficients[1]
    estimate2_se <- summary(t2)$test$sigma[1]
    estimate2_p <- summary(t2)$test$pvalues[1]
    #Writing out results
    Sim_fit_results[(j-1)*nSim*Repeats + (k-1)*Repeats + 
i,c(1,2,3,4,5,6,7,8,9,10,11,12,13,14,15,16,17,18,30,31,32,33,34,35,36)] <- c(j,Simulations[k],i,AIC,BIC,MSE, 
intercept, inter_SE, inter_p, inter_t3p, time, time_SE, time_p, time_t3p, interven, interven_SE, interven_p, 
interven_t3p, estimate, estimate_se, estimate_p, estimate2, estimate2_se, estimate2_p, cov_var)
```

```
  }
  #Fitted Model 4
  for (i in 1:Repeats) {
    j=13
    cov_var <- "ar1"
    data <- read.csv(paste("Sim",Simulations[k],"_",i,".csv", sep =""))
    data$Intervention <- as.factor(data$Intervention)
    fm1 <- try(lme(HONOS_scores ~ Time + Intervention + Time*Intervention, random = ~ 1 | Cluster_ID/Patient_ID,
data = data, method = "ML", correlation = corAR1(), control=lmeControl(returnObject=TRUE, maxIter=100)), TRUE)
    if(isTRUE(class(fm1)=="try-error")) { next } else { fm1=fm1 }
    K <- matrix(c(0,0,1,6), 1)
    t <- glht(fm1, linfct = K)
    K2 <- matrix(c(0,0,1,7), 1)
    t2 <- glht(fm1, linfct = K2)
    #Getting AIC
    AIC <- AIC(fm1)
    BIC <- BIC(fm1)
    MSE <- fm1$sigma
    #Intercept
    intercept <- summary(fm1)$tTable[1,1]
    inter_SE <- summary(fm1)$tTable[1,2]
    inter_p <- summary(fm1)$tTable[1,5]
    inter_t3p <- anova(fm1)[1,4]
    #Time
    time <- summary(fm1)$tTable[2,1]
    time_SE <- summary(fm1)$tTable[2,2]
    time_p <- summary(fm1)$tTable[2,5]
    time_t3p <- anova(fm1)[2,4]
    #Intervention
    interven <- summary(fm1)$tTable[3,1]
    interven_SE <- summary(fm1)$tTable[3,2]
    interven_p <- summary(fm1)$tTable[3,5]
    interven_t3p <- anova(fm1)[3,4]
    #Time*Intervention
    time_int <- summary(fm1)$tTable[4,1]
    time_int_SE <- summary(fm1)$tTable[4,2]
    time_int_p <- summary(fm1)$tTable[4,5]
    time_int_t3p <- anova(fm1)[4,4]
    #Estimate of effect at 6 months
```

```
    estimate <- summary(t)$test$coefficients[1]
    estimate_se <- summary(t)$test$sigma[1]
    estimate_p <- summary(t)$test$pvalues[1]
    #Estimate of time-averaged effect
    estimate2 <- summary(t2)$test$coefficients[1]
    estimate2_se <- summary(t2)$test$sigma[1]
    estimate2_p <- summary(t2)$test$pvalues[1]
    #Writing out results
    Sim_fit_results[(j-1)*nSim*Repeats + (k-1)*Repeats +
i,c(1,2,3,4,5,6,7,8,9,10,11,12,13,14,15,16,17,18,19,20,21,22,30,31,32,33,34,35,36)] <-
c(j,Simulations[k],i,AIC,BIC,MSE, intercept, inter_SE, inter_p, inter_t3p, time, time_SE, time_p, time_t3p,
interven, interven_SE, interven_p, interven_t3p, time_int, time_int_SE, time_int_p, time_int_t3p, estimate,
estimate_se, estimate_p, estimate2, estimate2_se, estimate2_p, cov_var)
  }
  #Fitted Model 5
  for (i in 1:Repeats) {
    j=14
    cov_var <- "ar1"
    data <- read.csv(paste("Sim",Simulations[k],"_",i,".csv", sep =""))
    data$Intervention <- as.factor(data$Intervention)
    data$Time <- as.factor(data$Time)
    data$Time_Int <- "IntTime0"
    data$Time_Int[data$Time == "3" & data$Intervention == "1"] <- "IntTime3"
    data$Time_Int[data$Time == "4" & data$Intervention == "1"] <- "IntTime4"
    data$Time_Int[data$Time == "5" & data$Intervention == "1"] <- "IntTime5"
    data$Time_Int[data$Time == "6" & data$Intervention == "1"] <- "IntTime6"
    data$Time_Int[data$Time == "7" & data$Intervention == "1"] <- "IntTime7"
    data$Time_Int[data$Time == "8" & data$Intervention == "1"] <- "IntTime8"
    data$Time_Int[data$Time == "9" & data$Intervention == "1"] <- "IntTime9"
    data$Time_Int[data$Time == "10" & data$Intervention == "1"] <- "IntTime10"
    data$Time_Int[data$Time == "11" & data$Intervention == "1"] <- "IntTime11"
    data$Time_Int[data$Time == "12" & data$Intervention == "1"] <- "IntTime12"
    data$Time_Int <- as.factor(data$Time_Int)
    fm1 <- try(lme(HONOS_scores ~ Time + Intervention + Time_Int, random = ~ 1 | Cluster_ID/Patient_ID, data = data,
method = "ML", correlation = corAR1(), control=lmeControl(returnObject=TRUE, maxIter=100)), TRUE)
    if(isTRUE(class(fm1)=="try-error")) { next } else { fm1=fm1 }
    K <- matrix(c(0,0,0,0,0,0,0,0,0,0,0,0,1,0,0,0,0,0,0,1,0,0,0), 1)
    t <- glht(fm1, linfct = K)
    K2 <- matrix(c(0,0,0,0,0,0,0,0,0,0,0,0,1,0.1,0.1,0.1,0.1,0.1,0.1,0.1,0.1,0.1,0.1), 1)
```

```
    t2 <- glht(fm1, linfct = K2)
    #Getting AIC
    AIC <- AIC(fm1)
    BIC <- BIC(fm1)
    MSE <- fm1$sigma
    #Intercept
    intercept <- summary(fm1)$tTable[1,1]
    inter_SE <- summary(fm1)$tTable[1,2]
    inter_p <- summary(fm1)$tTable[1,5]
    inter_t3p <- anova(fm1)[1,4]
    #Time
    time <- NA
    time_SE <- NA
    time_p <- NA
    time_t3p <- anova(fm1)[2,4]
    #Intervention
    interven <- summary(fm1)$tTable[14,1]
    interven_SE <- summary(fm1)$tTable[14,2]
    interven_p <- summary(fm1)$tTable[14,5]
    interven_t3p <- anova(fm1)[3,4]
    #Time*Intervention
    time_int <- NA
    time_int_SE <- NA
    time_int_p <- NA
    time_int_t3p <- anova(fm1)[4,4]
    #Estimate of effect at 6 months
    estimate <- summary(t)$test$coefficients[1]
    estimate_se <- summary(t)$test$sigma[1]
    estimate_p <- summary(t)$test$pvalues[1]
    #Estimate of time-averaged effect
    estimate2 <- summary(t2)$test$coefficients[1]
    estimate2_se <- summary(t2)$test$sigma[1]
    estimate2_p <- summary(t2)$test$pvalues[1]
    #Writing out results
    Sim_fit_results[(j-1)*nSim*Repeats + (k-1)*Repeats +
i,c(1,2,3,4,5,6,7,8,9,10,11,12,13,14,15,16,17,18,19,20,21,22,30,31,32,33,34,35,36)] <-
c(j,Simulations[k],i,AIC,BIC,MSE, intercept, inter_SE, inter_p, inter_t3p, time, time_SE, time_p, time_t3p,
interven, interven_SE, interven_p, interven_t3p, time_int, time_int_SE, time_int_p, time_int_t3p, estimate,
estimate_se, estimate_p, estimate2, estimate2_se, estimate2_p, cov_var)
```

```
  }
  #Fitted Model 6
  for (i in 1:Repeats) {
    j=15
    cov_var <- "ar1"
    data <- read.csv(paste("Sim",Simulations[k],"_",i,".csv", sep =""))
    data$Intervention <- as.factor(data$Intervention)
    fm1 <- try(lme(HONOS_scores ~ Time + Intervention + TimeInt, random = ~ 1 | Cluster_ID/Patient_ID, data = data,
method = "ML", correlation = corAR1(), control=lmeControl(returnObject=TRUE, maxIter=100)), TRUE)
    if(isTRUE(class(fm1)=="try-error")) { next } else { fm1=fm1 }
    K <- matrix(c(0, 0, 1, 6), 1)
    t <- glht(fm1, linfct = K)
    K2 <- matrix(c(0, 0, 1, 6), 1)
    t2 <- glht(fm1, linfct = K2)
    #Getting AIC
    AIC <- AIC(fm1)
    BIC <- BIC(fm1)
    MSE <- fm1$sigma
    #Intercept
    intercept <- summary(fm1)$tTable[1,1]
    inter_SE <- summary(fm1)$tTable[1,2]
    inter_p <- summary(fm1)$tTable[1,5]
    inter_t3p <- anova(fm1)[1,4]
    #Time
    time <- summary(fm1)$tTable[2,1]
    time_SE <- summary(fm1)$tTable[2,2]
    time_p <- summary(fm1)$tTable[2,5]
    time_t3p <- anova(fm1)[2,4]
    #Intervention
    interven <- summary(fm1)$tTable[3,1]
    interven_SE <- summary(fm1)$tTable[3,2]
    interven_p <- summary(fm1)$tTable[3,5]
    interven_t3p <- anova(fm1)[3,4]
    #Time*Intervention
    timeint <- summary(fm1)$tTable[4,1]
    timeint_SE <- summary(fm1)$tTable[4,2]
    timeint_p <- summary(fm1)$tTable[4,5]
    timeint_t3p <- anova(fm1)[4,4]
    #Estimate of effect at 6 months
```

```
    estimate <- summary(t)$test$coefficients[1]
    estimate_se <- summary(t)$test$sigma[1]
    estimate_p <- summary(t)$test$pvalues[1]
    #Estimate of time-averaged effect
    estimate2 <- summary(t2)$test$coefficients[1]
    estimate2_se <- summary(t2)$test$sigma[1]
    estimate2_p <- summary(t2)$test$pvalues[1]
    #Writing out results
    Sim_fit_results[(j-1)*nSim*Repeats + (k-1)*Repeats +
i,c(1,2,3,4,5,6,7,8,9,10,11,12,13,14,15,16,17,18,23,24,25,26,30,31,32,33,34,35,36)] <-
c(j,Simulations[k],i,AIC,BIC,MSE, intercept, inter_SE, inter_p, inter_t3p, time, time_SE, time_p, time_t3p,
interven, interven_SE, interven_p, interven_t3p, timeint, timeint_SE, timeint_p, timeint_t3p, estimate, estimate_se,
estimate_p, estimate2, estimate2_se, estimate2_p, cov_var)
  }
  #Fitted Model 7
  for (i in 1:Repeats) {
    j=16
    cov_var <- "ar1"
    data <- read.csv(paste("Sim",Simulations[k],"_",i,".csv", sep =""))
    data$Intervention <- as.factor(data$Intervention)
    fm1 <- try(lme(HONOS_scores ~ Time + TimeInt, random = ~ 1 | Cluster_ID/Patient_ID, data = data, method = "ML",
correlation = corAR1(), control=lmeControl(returnObject=TRUE, maxIter=100)), TRUE)
    if(isTRUE(class(fm1)=="try-error")) { next } else { fm1=fm1 }
    K <- matrix(c(0, 0, 6), 1)
    t <- glht(fm1, linfct = K)
    K2 <- matrix(c(0, 0, 6), 1)
    t2 <- glht(fm1, linfct = K2)
    #Getting AIC
    AIC <- AIC(fm1)
    BIC <- BIC(fm1)
    MSE <- fm1$sigma
    #Intercept
    intercept <- summary(fm1)$tTable[1,1]
    inter_SE <- summary(fm1)$tTable[1,2]
    inter_p <- summary(fm1)$tTable[1,5]
    inter_t3p <- anova(fm1)[1,4]
    #Time
    time <- summary(fm1)$tTable[2,1]
    time_SE <- summary(fm1)$tTable[2,2]
```

```
    time_p <- summary(fm1)$tTable[2,5]
    time_t3p <- anova(fm1)[2,4]
    #Exposure Time
    timeint <- summary(fm1)$tTable[3,1]
    timeint_SE <- summary(fm1)$tTable[3,2]
    timeint_p <- summary(fm1)$tTable[3,5]
    timeint_t3p <- anova(fm1)[3,4]
    #Estimate of effect at 6 months
    estimate <- summary(t)$test$coefficients[1]
    estimate_se <- summary(t)$test$sigma[1]
    estimate_p <- summary(t)$test$pvalues[1]
    #Estimate of time-averaged effect
    estimate2 <- summary(t2)$test$coefficients[1]
    estimate2_se <- summary(t2)$test$sigma[1]
    estimate2_p <- summary(t2)$test$pvalues[1]
    #Writing out results
    Sim_fit_results[(j-1)*nSim*Repeats + (k-1)*Repeats +
i,c(1,2,3,4,5,6,7,8,9,10,11,12,13,14,23,24,25,26,30,31,32,33,34,35,36)] <- c(j,Simulations[k],i,AIC,BIC,MSE,
intercept, inter_SE, inter_p, inter_t3p, time, time_SE, time_p, time_t3p, timeint, timeint_SE, timeint_p,
timeint_t3p, estimate, estimate_se, estimate_p, estimate2, estimate2_se, estimate2_p, cov_var)
  }
  #Fitted Model 8
  for (i in 1:Repeats) {
    j=17
    cov_var <- "ar1"
    data <- read.csv(paste("Sim",Simulations[k],"_",i,".csv", sep =""))
    data$Intervention <- as.factor(data$Intervention)
    data$Time <- as.factor(data$Time)
    data$TimeInt <- as.factor(data$TimeInt)
    fm1 <- try(lme(HONOS_scores ~ Time + TimeInt, random = ~ 1 | Cluster_ID/Patient_ID, data = data, method = "ML",
correlation = corAR1(), control=lmeControl(returnObject=TRUE, maxIter=100)), TRUE)
    if(isTRUE(class(fm1)=="try-error")) { next } else { fm1=fm1 }
    K <- matrix(c(0,0,0,0,0,0,0,0,0,0,0,0,0,0,0,0,0,1,0,0,0,0,0,0), 1)
    t <- glht(fm1, linfct = K)
    K2 <-
matrix(c(0,0,0,0,0,0,0,0,0,0,0,0,0.08333333,0.08333333,0.08333333,0.08333333,0.08333333,0.08333333,0.08333333,0.08
333333,0.08333333,0.08333333,0.08333333,0.08333333), 1)
    t2 <- glht(fm1, linfct = K2)
    #Getting AIC
```

```
    AIC <- AIC(fm1)
    BIC <- BIC(fm1)
    MSE <- fm1$sigma
    #Intercept
    intercept <- summary(fm1)$tTable[1,1]
    inter_SE <- summary(fm1)$tTable[1,2]
    inter_p <- summary(fm1)$tTable[1,5]
    inter_t3p <- anova(fm1)[1,4]
    #Time
    time <- NA
    time_SE <- NA
    time_p <- NA
    time_t3p <- anova(fm1)[1,4]
    #Exposure Time
    timeint <- NA
    timeint_SE <- NA
    timeint_p <- NA
    timeint_t3p <- anova(fm1)[2,4]
    #Estimate of effect at 6 months
    estimate <- summary(t)$test$coefficients[1]
    estimate_se <- summary(t)$test$sigma[1]
    estimate_p <- summary(t)$test$pvalues[1]
    #Estimate of time-averaged effect
    estimate2 <- summary(t2)$test$coefficients[1]
    estimate2_se <- summary(t2)$test$sigma[1]
    estimate2_p <- summary(t2)$test$pvalues[1]
    #Writing out results
    Sim_fit_results[(j-1)*nSim*Repeats + (k-1)*Repeats +
i,c(1,2,3,4,5,6,7,8,9,10,11,12,13,14,23,24,25,26,30,31,32,33,34,35,36)] <- c(j,Simulations[k],i,AIC,BIC,MSE,
intercept, inter_SE, inter_p, inter_t3p, time, time_SE, time_p, time_t3p, timeint, timeint_SE, timeint_p,
timeint_t3p, estimate, estimate_se, estimate_p, estimate2, estimate2_se, estimate2_p, cov_var)
  }
  #Fitted Model 9
  for (i in 1:Repeats) {
    j=18
    cov_var <- "ar1"
    data <- read.csv(paste("Sim",Simulations[k],"_",i,".csv", sep =""))
    data$Intervention <- as.factor(data$Intervention)
```

```
    fm1 <- try(lme(HONOS_scores ~ Time + Intervention + TimeInt + I(Time^2), random = ~ 1 | Cluster_ID/Patient_ID,
data = data, method = "ML", correlation = corAR1(), control=lmeControl(returnObject=TRUE, maxIter=100)), TRUE)
    if(isTRUE(class(fm1)=="try-error")) { next } else { fm1=fm1 }
    K <- matrix(c(0, 0, 1, 6, 0), 1)
    t <- glht(fm1, linfct = K)
    K2 <- matrix(c(0, 0, 1, 6, 0), 1)
    t2 <- glht(fm1, linfct = K2)
    #Getting AIC
    AIC <- AIC(fm1)
    BIC <- BIC(fm1)
    MSE <- fm1$sigma
    #Intercept
    intercept <- summary(fm1)$tTable[1,1]
    inter_SE <- summary(fm1)$tTable[1,2]
    inter_p <- summary(fm1)$tTable[1,5]
    inter_t3p <- anova(fm1)[1,4]
    #Time
    time <- summary(fm1)$tTable[2,1]
    time_SE <- summary(fm1)$tTable[2,2]
    time_p <- summary(fm1)$tTable[2,5]
    time_t3p <- anova(fm1)[2,4]
    #Intervention
    interven <- summary(fm1)$tTable[3,1]
    interven_SE <- summary(fm1)$tTable[3,2]
    interven_p <- summary(fm1)$tTable[3,5]
    interven_t3p <- anova(fm1)[3,4]
    #Time*Intervention
    timeint <- summary(fm1)$tTable[4,1]
    timeint_SE <- summary(fm1)$tTable[4,2]
    timeint_p <- summary(fm1)$tTable[4,5]
    timeint_t3p <- anova(fm1)[4,4]
    #Timesq
    timesq <- summary(fm1)$tTable[5,1]
    timesq_SE <- summary(fm1)$tTable[5,2]
    timesq_p <- summary(fm1)$tTable[5,5]
    timesq_t3p <- anova(fm1)[5,4]
    #Estimate of effect at 6 months
    estimate <- summary(t)$test$coefficients[1]
    estimate_se <- summary(t)$test$sigma[1]
```

```
    estimate_p <- summary(t)$test$pvalues[1]
    #Estimate of time-averaged effect
    estimate2 <- summary(t2)$test$coefficients[1]
    estimate2_se <- summary(t2)$test$sigma[1]
    estimate2_p <- summary(t2)$test$pvalues[1]
    #Writing out results
    Sim_fit_results[(j-1)*nSim*Repeats + (k-1)*Repeats +
i,c(1,2,3,4,5,6,7,8,9,10,11,12,13,14,15,16,17,18,23,24,25,26,27,28,29,30,31,32,33,34,35,36)] <-
c(j,Simulations[k],i,AIC,BIC,MSE, intercept, inter_SE, inter_p, inter_t3p, time, time_SE, time_p, time_t3p,
interven, interven_SE, interven_p, interven_t3p, timeint, timeint_SE, timeint_p, timeint_t3p, timesq, timesq_SE,
timesq_p, estimate, estimate_se, estimate_p, estimate2, estimate2_se, estimate2_p, cov_var)
  }
}
```
