# Supplementary material for: Mixed effects approach to the analysis of the stepped wedge cluster randomised trial—Investigating the confounding effect of time through simulation
Source: PLoS One. 2018 Dec 13;13(12):e0208876. doi: 10.1371/journal.pone.0208876 (PMC6292598; doi:10.1371/journal.pone.0208876)
Supplement: S3 Appendix — (PDF) [file pone.0208876.s003.pdf]

S3 Appendix. Model estimates for each scenario

S3 Table A: The mean treatment effect estimates, their standard errors, and goodness-of-fit statistics, for each of the 36 simulated scenarios assuming the mean structure from Model 1 ($y_{itk} = \beta_0 + \partial x_{tk} + v_{0ik} + \epsilon_{itk}$) with compound symmetric covariance structure between within subject measurement, together with the Monte Carlo standard error of each estimate in brackets. Each scenario was simulated 1000 times.

				Treatment Effect at 6 months						Time Averaged Treatment Effect					
Scenario	BIC	MSE	Proportion Significant Intervention Parameter	True Value	Estimate	Estimate SE	Coverage of 95% C.I.	Bias	Confidence Interval Width	True Value	Estimate	Estimate SE	Coverage of 95% C.I.	Bias	Confidence Interval Width
D1	19472.8 (2.7)	4.9 (0)	0.06	0	-0.01 (0.062)	0.21 (0.0001)	93.8%	-0.01 (0.0068)	0.81 (0.0004)	0	-0.01 (0.0068)	0.21 (0.0001)	93.8%	-0.01 (0.0068)	0.81 (0.0004)
D2	19067.8 (2.6)	4.6 (0)	0.05	0	0 (0.046)	0.19 (0.0001)	95.4%	0 (0.0059)	0.75 (0.0003)	0	0 (0.0059)	0.19 (0.0001)	95.4%	0 (0.0059)	0.75 (0.0003)
D3	19519.2 (2.6)	5 (0)	1	0	1.58 (1)	0.21 (0.0001)	0%	1.58 (0.0068)	0.81 (0.0004)	0	1.58 (0.0068)	0.21 (0.0001)	0%	1.58 (0.0068)	0.81 (0.0004)
D4	19122.8 (2.5)	4.6 (0)	1	0	1.59 (1)	0.19 (0.0001)	0%	1.59 (0.0059)	0.76 (0.0003)	0	1.59 (0.0059)	0.19 (0.0001)	0%	1.59 (0.0059)	0.76 (0.0003)
D5	19527.4 (2.7)	5 (0)	0.06	0	-0.01 (0.057)	0.21 (0.0001)	94.3%	-0.01 (0.0068)	0.81 (0.0004)	0	-0.01 (0.0068)	0.21 (0.0001)	94.3%	-0.01 (0.0068)	0.81 (0.0004)
D6	19133 (2.6)	4.6 (0)	0.04	0	0 (0.042)	0.19 (0.0001)	95.8%	0 (0.0059)	0.76 (0.0003)	0	0 (0.0059)	0.19 (0.0001)	95.8%	0 (0.0059)	0.76 (0.0003)
D7	19641.4 (2.6)	5.1 (0)	1	0	-1.56 (1)	0.21 (0.0001)	0%	-1.56 (0.0068)	0.83 (0.0004)	0	-1.56 (0.0068)	0.21 (0.0001)	0%	-1.56 (0.0068)	0.83 (0.0004)
D8	19264.4 (2.6)	4.7 (0)	1	0	-1.57 (1)	0.2 (0.0001)	0%	-1.57 (0.0059)	0.78 (0.0003)	0	-1.57 (0.0059)	0.2 (0.0001)	0%	-1.57 (0.0059)	0.78 (0.0003)
D9	19466.7 (2.7)	4.9 (0)	1	2	1.99 (1)	0.21 (0.0001)	94%	-0.01 (0.0068)	0.81 (0.0003)	2	1.99 (0.0068)	0.21 (0.0001)	94%	-0.01 (0.0068)	0.81 (0.0003)
D10	19062.5 (2.6)	4.6 (0)	1	2	2 (1)	0.19 (0.0001)	95.6%	0 (0.0059)	0.75 (0.0003)	2	2 (0.0059)	0.19 (0.0001)	95.6%	0 (0.0059)	0.75 (0.0003)
D11	19519.2 (2.6)	5 (0)	1	2	3.58 (1)	0.21 (0.0001)	0%	1.58 (0.0068)	0.81 (0.0004)	2	3.58 (0.0068)	0.21 (0.0001)	0%	1.58 (0.0068)	0.81 (0.0004)
D12	19122.8 (2.5)	4.6 (0)	1	2	3.59 (1)	0.19 (0.0001)	0%	1.59 (0.0059)	0.76 (0.0003)	2	3.59 (0.0059)	0.19 (0.0001)	0%	1.59 (0.0059)	0.76 (0.0003)
D13	19558.5 (2.6)	5 (0)	1	2.9	4.15 (1)	0.21 (0.0001)	0%	1.25 (0.0068)	0.82 (0.0004)	2.9	4.15 (0.0068)	0.21 (0.0001)	0%	1.25 (0.0068)	0.82 (0.0004)
D14	19168.6 (2.5)	4.7 (0)	1	2.9	4.16 (1)	0.19 (0.0001)	0%	1.26 (0.0059)	0.76 (0.0003)	2.9	4.16 (0.0059)	0.19 (0.0001)	0%	1.26 (0.0059)	0.76 (0.0003)
D15	19558.5 (2.6)	5 (0)	1	0.9	2.15 (1)	0.21 (0.0001)	0%	1.25 (0.0068)	0.82 (0.0004)	0.9	2.15 (0.0068)	0.21 (0.0001)	0%	1.25 (0.0068)	0.82 (0.0004)
D16	19168.6 (2.5)	4.7 (0)	1	0.9	2.16 (1)	0.19 (0.0001)	0%	1.26 (0.0059)	0.76 (0.0003)	0.9	2.16 (0.0059)	0.19 (0.0001)	0%	1.26 (0.0059)	0.76 (0.0003)
D17	19595.6 (2.6)	5 (0)	1	3.5	4.54 (1)	0.21 (0.0001)	0.1%	1.04 (0.0068)	0.82 (0.0004)	3.5	4.54 (0.0068)	0.21 (0.0001)	0.1%	1.04 (0.0068)	0.82 (0.0004)
D18	19211.6 (2.5)	4.7 (0)	1	3.5	4.54 (1)	0.2 (0.0001)	0%	1.04 (0.0059)	0.77 (0.0003)	3.5	4.54 (0.0059)	0.2 (0.0001)	0%	1.04 (0.0059)	0.77 (0.0003)
D19	19595.6 (2.6)	5 (0)	1	1.5	2.54 (1)	0.21 (0.0001)	0.1%	1.04 (0.0068)	0.82 (0.0004)	1.5	2.54 (0.0068)	0.21 (0.0001)	0.1%	1.04 (0.0068)	0.82 (0.0004)
D20	19211.6 (2.5)	4.7 (0)	1	1.5	2.54 (1)	0.2 (0.0001)	0%	1.04 (0.0059)	0.77 (0.0003)	1.5	2.54 (0.0059)	0.2 (0.0001)	0%	1.04 (0.0059)	0.77 (0.0003)
D21	19535.3 (2.7)	5 (0)	1	-5	-2.32 (1)	0.21 (0.0001)	0%	2.68 (0.0068)	0.82 (0.0004)	-5	-2.32 (0.0068)	0.21 (0.0001)	0%	2.68 (0.0068)	0.82 (0.0004)
D22	19140.1 (2.6)	4.6 (0)	1	-5	-2.3 (1)	0.19 (0.0001)	0%	2.7 (0.006)	0.76 (0.0003)	-5	-2.3 (0.006)	0.19 (0.0001)	0%	2.7 (0.006)	0.76 (0.0003)
D23	19535.3 (2.7)	5 (0)	0.34	-3	-0.32 (0.335)	0.21 (0.0001)	0%	2.68 (0.0068)	0.82 (0.0004)	-3	-0.32 (0.0068)	0.21 (0.0001)	0%	2.68 (0.0068)	0.82 (0.0004)
D24	19140.1 (2.6)	4.6 (0)	0.33	-3	-0.3 (0.329)	0.19 (0.0001)	0%	2.7 (0.006)	0.76 (0.0003)	-3	-0.3 (0.006)	0.19 (0.0001)	0%	2.7 (0.006)	0.76 (0.0003)
D25	19527.4 (2.7)	5 (0)	1	2	1.99 (1)	0.21 (0.0001)	94.3%	-0.01 (0.0068)	0.81 (0.0004)	2	1.99 (0.0068)	0.21 (0.0001)	94.3%	-0.01 (0.0068)	0.81 (0.0004)
D26	19133 (2.6)	4.6 (0)	1	2	2 (1)	0.19 (0.0001)	95.8%	0 (0.0059)	0.76 (0.0003)	2	2 (0.0059)	0.19 (0.0001)	95.8%	0 (0.0059)	0.76 (0.0003)
D27	19532 (2.7)	5 (0)	1	2.966	2.51 (1)	0.21 (0.0001)	41.5%	-0.46 (0.0068)	0.81 (0.0004)	2.58	2.51 (0.0068)	0.21 (0.0001)	92.1%	-0.07 (0.0068)	0.81 (0.0004)
D28	19138.4 (2.6)	4.6 (0)	1	2.966	2.51 (1)	0.19 (0.0001)	35.6%	-0.45 (0.0059)	0.76 (0.0003)	2.58	2.51 (0.0059)	0.19 (0.0001)	93.4%	-0.07 (0.0059)	0.76 (0.0003)
D29	19532 (2.7)	5 (0)	0.68	0.966	0.51 (0.682)	0.21 (0.0001)	41.5%	-0.46 (0.0068)	0.81 (0.0004)	0.58	0.51 (0.0068)	0.21 (0.0001)	92.1%	-0.07 (0.0068)	0.81 (0.0004)
D30	19138.4 (2.6)	4.6 (0)	0.76	0.966	0.51 (0.757)	0.19 (0.0001)	35.6%	-0.45 (0.0059)	0.76 (0.0003)	0.58	0.51 (0.0059)	0.19 (0.0001)	93.4%	-0.07 (0.0059)	0.76 (0.0003)
D31	19641.4 (2.6)	5.1 (0)	0.54	2	0.44 (0.54)	0.21 (0.0001)	0%	-1.56 (0.0068)	0.83 (0.0004)	2	0.44 (0.0068)	0.21 (0.0001)	0%	-1.56 (0.0068)	0.83 (0.0004)
D32	19264.4 (2.6)	4.7 (0)	0.59	2	0.43 (0.593)	0.2 (0.0001)	0%	-1.57 (0.0059)	0.78 (0.0003)	2	0.43 (0.0059)	0.2 (0.0001)	0%	-1.57 (0.0059)	0.78 (0.0003)
D33	19679.8 (2.6)	5.1 (0)	0.98	2.5	0.83 (0.975)	0.21 (0.0001)	0%	-1.67 (0.0068)	0.84 (0.0004)	2	0.83 (0.0068)	0.21 (0.0001)	0%	-1.17 (0.0068)	0.84 (0.0004)
D34	19308.8 (2.6)	4.8 (0)	0.99	2.5	0.82 (0.988)	0.2 (0.0001)	0%	-1.68 (0.0059)	0.78 (0.0003)	2	0.82 (0.0059)	0.2 (0.0001)	0%	-1.18 (0.0059)	0.78 (0.0003)
D35	19679.8 (2.6)	5.1 (0)	1	0.5	-1.17 (1)	0.21 (0.0001)	0%	-1.67 (0.0068)	0.84 (0.0004)	0	-1.17 (0.0068)	0.21 (0.0001)	0%	-1.17 (0.0068)	0.84 (0.0004)
D36	19308.8 (2.6)	4.8 (0)	1	0.5	-1.18 (1)	0.2 (0.0001)	0%	-1.68 (0.0059)	0.78 (0.0003)	0	-1.18 (0.0059)	0.2 (0.0001)	0%	-1.18 (0.0059)	0.78 (0.0003)

S3 Table B: The mean treatment effect estimates, their standard errors, and goodness-of-fit statistics, for each of the 36 simulated scenarios assuming the mean structure from Model 2 ($y_{itk} = \beta_0 + \partial x_{tk} + \tau t + v_{0ik} + \epsilon_{ijk}$) with compound symmetric covariance structure between within subject measurement, together with the Monte Carlo standard error of each estimate in brackets. Each scenario was simulated 1000 times.

				Treatment Effect at 6 months						Time Averaged Treatment Effect					
Scenario	BIC	MSE	Proportion Significant Intervention Parameter	True Value	Estimate	Estimate SE	Coverage of 95% C.I.	Bias	Confidence Interval Width	True Value	Estimate	Estimate SE	Coverage of 95% C.I.	Bias	Confidence Interval Width
D1	19479.8 (2.7)	4.9 (0)	0.05	0	-0.01 (0.052)	0.3 (0.0001)	94.8%	-0.01 (0.0096)	1.18 (0.0006)	0	-0.01 (0.0096)	0.3 (0.0001)	94.8%	-0.01 (0.0096)	1.18 (0.0006)
D2	19074.9 (2.6)	4.6 (0)	0.05	0	0 (0.047)	0.28 (0.0001)	95.3%	0 (0.0087)	1.1 (0.0005)	0	0 (0.0087)	0.28 (0.0001)	95.3%	0 (0.0087)	1.1 (0.0005)
D3	19473.7 (2.7)	4.9 (0)	0.05	0	-0.01 (0.051)	0.3 (0.0001)	94.9%	-0.01 (0.0096)	1.17 (0.0005)	0	-0.01 (0.0096)	0.3 (0.0001)	94.9%	-0.01 (0.0096)	1.17 (0.0005)
D4	19069.6 (2.6)	4.6 (0)	0.04	0	0 (0.044)	0.28 (0.0001)	95.6%	0 (0.0087)	1.09 (0.0005)	0	0 (0.0087)	0.28 (0.0001)	95.6%	0 (0.0087)	1.09 (0.0005)
D5	19534.4 (2.7)	5 (0)	0.05	0	-0.01 (0.05)	0.3 (0.0001)	95%	-0.01 (0.0096)	1.18 (0.0005)	0	-0.01 (0.0096)	0.3 (0.0001)	95%	-0.01 (0.0096)	1.18 (0.0005)
D6	19140.1 (2.6)	4.6 (0)	0.04	0	0 (0.042)	0.28 (0.0001)	95.8%	0 (0.0087)	1.11 (0.0005)	0	0 (0.0087)	0.28 (0.0001)	95.8%	0 (0.0087)	1.11 (0.0005)
D7	19599.6 (2.6)	5 (0)	0.05	0	-0.01 (0.047)	0.31 (0.0001)	95.3%	-0.01 (0.0096)	1.2 (0.0005)	0	-0.01 (0.0096)	0.31 (0.0001)	95.3%	-0.01 (0.0096)	1.2 (0.0005)
D8	19215.6 (2.5)	4.7 (0)	0.04	0	0 (0.042)	0.29 (0.0001)	95.8%	0 (0.0087)	1.12 (0.0005)	0	0 (0.0087)	0.29 (0.0001)	95.8%	0 (0.0087)	1.12 (0.0005)
D9	19473.7 (2.7)	4.9 (0)	1	2	1.99 (1)	0.3 (0.0001)	94.9%	-0.01 (0.0096)	1.17 (0.0005)	2	1.99 (0.0096)	0.3 (0.0001)	94.9%	-0.01 (0.0096)	1.17 (0.0005)
D10	19069.6 (2.6)	4.6 (0)	1	2	2 (1)	0.28 (0.0001)	95.6%	0 (0.0087)	1.09 (0.0005)	2	2 (0.0087)	0.28 (0.0001)	95.6%	0 (0.0087)	1.09 (0.0005)
D11	19473.7 (2.7)	4.9 (0)	1	2	1.99 (1)	0.3 (0.0001)	94.9%	-0.01 (0.0096)	1.17 (0.0005)	2	1.99 (0.0096)	0.3 (0.0001)	94.9%	-0.01 (0.0096)	1.17 (0.0005)
D12	19069.6 (2.6)	4.6 (0)	1	2	2 (1)	0.28 (0.0001)	95.6%	0 (0.0087)	1.09 (0.0005)	2	2 (0.0087)	0.28 (0.0001)	95.6%	0 (0.0087)	1.09 (0.0005)
D13	19479.9 (2.7)	4.9 (0)	1	2.9	2.12 (1)	0.3 (0.0001)	26.3%	-0.78 (0.0096)	1.17 (0.0005)	2.9	2.12 (0.0096)	0.3 (0.0001)	26.3%	-0.78 (0.0096)	1.17 (0.0005)
D14	19076.8 (2.6)	4.6 (0)	1	2.9	2.12 (1)	0.28 (0.0001)	19.8%	-0.78 (0.0087)	1.1 (0.0005)	2.9	2.12 (0.0087)	0.28 (0.0001)	19.8%	-0.78 (0.0087)	1.1 (0.0005)
D15	19479.9 (2.7)	4.9 (0)	0.07	0.9	0.12 (0.07)	0.3 (0.0001)	26.3%	-0.78 (0.0096)	1.17 (0.0005)	0.9	0.12 (0.0096)	0.3 (0.0001)	26.3%	-0.78 (0.0096)	1.17 (0.0005)
D16	19076.8 (2.6)	4.6 (0)	0.07	0.9	0.12 (0.068)	0.28 (0.0001)	19.8%	-0.78 (0.0087)	1.1 (0.0005)	0.9	0.12 (0.0087)	0.28 (0.0001)	19.8%	-0.78 (0.0087)	1.1 (0.0005)
D17	19490.9 (2.7)	4.9 (0)	1	3.5	2.21 (1)	0.3 (0.0001)	1.1%	-1.29 (0.0096)	1.18 (0.0005)	3.5	2.21 (0.0096)	0.3 (0.0001)	1.1%	-1.29 (0.0096)	1.18 (0.0005)
D18	19089.4 (2.6)	4.6 (0)	1	3.5	2.19 (1)	0.28 (0.0001)	0.3%	-1.31 (0.0087)	1.1 (0.0005)	3.5	2.19 (0.0087)	0.28 (0.0001)	0.3%	-1.31 (0.0087)	1.1 (0.0005)
D19	19490.9 (2.7)	4.9 (0)	0.11	1.5	0.21 (0.11)	0.3 (0.0001)	1.1%	-1.29 (0.0096)	1.18 (0.0005)	1.5	0.21 (0.0096)	0.3 (0.0001)	1.1%	-1.29 (0.0096)	1.18 (0.0005)
D20	19089.4 (2.6)	4.6 (0)	0.11	1.5	0.19 (0.107)	0.28 (0.0001)	0.3%	-1.31 (0.0087)	1.1 (0.0005)	1.5	0.19 (0.0087)	0.28 (0.0001)	0.3%	-1.31 (0.0087)	1.1 (0.0005)
D21	19542.2 (2.7)	5 (0)	1	-5	-2.39 (1)	0.3 (0.0001)	0%	2.61 (0.0095)	1.19 (0.0006)	-5	-2.39 (0.0095)	0.3 (0.0001)	0%	2.61 (0.0095)	1.19 (0.0006)
D22	19147.2 (2.6)	4.6 (0)	1	-5	-2.36 (1)	0.28 (0.0001)	0%	2.64 (0.0089)	1.11 (0.0005)	-5	-2.36 (0.0089)	0.28 (0.0001)	0%	2.64 (0.0089)	1.11 (0.0005)
D23	19542.2 (2.7)	5 (0)	0.27	-3	-0.39 (0.268)	0.3 (0.0001)	0%	2.61 (0.0095)	1.19 (0.0006)	-3	-0.39 (0.0095)	0.3 (0.0001)	0%	2.61 (0.0095)	1.19 (0.0006)
D24	19147.2 (2.6)	4.6 (0)	0.26	-3	-0.36 (0.259)	0.28 (0.0001)	0%	2.64 (0.0089)	1.11 (0.0005)	-3	-0.36 (0.0089)	0.28 (0.0001)	0%	2.64 (0.0089)	1.11 (0.0005)
D25	19534.4 (2.7)	5 (0)	1	2	1.99 (1)	0.3 (0.0001)	95%	-0.01 (0.0096)	1.18 (0.0005)	2	1.99 (0.0096)	0.3 (0.0001)	95%	-0.01 (0.0096)	1.18 (0.0005)
D26	19140.1 (2.6)	4.6 (0)	1	2	2 (1)	0.28 (0.0001)	95.8%	0 (0.0087)	1.11 (0.0005)	2	2 (0.0087)	0.28 (0.0001)	95.8%	0 (0.0087)	1.11 (0.0005)
D27	19537.7 (2.7)	5 (0)	1	2.966	2.26 (1)	0.3 (0.0001)	35%	-0.7 (0.0096)	1.18 (0.0005)	2.58	2.26 (0.0096)	0.3 (0.0001)	80.6%	-0.32 (0.0096)	1.18 (0.0005)
D28	19144 (2.6)	4.6 (0)	1	2.966	2.26 (1)	0.28 (0.0001)	28.2%	-0.71 (0.0087)	1.11 (0.0005)	2.58	2.26 (0.0087)	0.28 (0.0001)	79%	-0.32 (0.0087)	1.11 (0.0005)
D29	19537.7 (2.7)	5 (0)	0.14	0.966	0.26 (0.137)	0.3 (0.0001)	35%	-0.7 (0.0096)	1.18 (0.0005)	0.58	0.26 (0.0096)	0.3 (0.0001)	80.6%	-0.32 (0.0096)	1.18 (0.0005)
D30	19144 (2.6)	4.6 (0)	0.14	0.966	0.26 (0.144)	0.28 (0.0001)	28.2%	-0.71 (0.0087)	1.11 (0.0005)	0.58	0.26 (0.0087)	0.28 (0.0001)	79%	-0.32 (0.0087)	1.11 (0.0005)
D31	19599.6 (2.6)	5 (0)	1	2	1.99 (1)	0.31 (0.0001)	95.3%	-0.01 (0.0096)	1.2 (0.0005)	2	1.99 (0.0096)	0.31 (0.0001)	95.3%	-0.01 (0.0096)	1.2 (0.0005)
D32	19215.6 (2.5)	4.7 (0)	1	2	2 (1)	0.29 (0.0001)	95.8%	0 (0.0087)	1.12 (0.0005)	2	2 (0.0087)	0.29 (0.0001)	95.8%	0 (0.0087)	1.12 (0.0005)
D33	19613.2 (2.6)	5.1 (0)	1	2.5	2.75 (1)	0.31 (0.0001)	87.5%	0.25 (0.0096)	1.2 (0.0005)	2	2.75 (0.0096)	0.31 (0.0001)	32.7%	0.75 (0.0096)	1.2 (0.0005)
D34	19231 (2.6)	4.7 (0)	1	2.5	2.77 (1)	0.29 (0.0001)	85.8%	0.27 (0.0087)	1.12 (0.0005)	2	2.77 (0.0087)	0.29 (0.0001)	24.1%	0.77 (0.0087)	1.12 (0.0005)
D35	19613.2 (2.6)	5.1 (0)	0.67	0.5	0.75 (0.673)	0.31 (0.0001)	87.5%	0.25 (0.0096)	1.2 (0.0005)	0	0.75 (0.0096)	0.31 (0.0001)	32.7%	0.75 (0.0096)	1.2 (0.0005)
D36	19231 (2.6)	4.7 (0)	0.76	0.5	0.77 (0.759)	0.29 (0.0001)	85.8%	0.27 (0.0087)	1.12 (0.0005)	0	0.77 (0.0087)	0.29 (0.0001)	24.1%	0.77 (0.0087)	1.12 (0.0005)

S3 Table C: The mean treatment effect estimates, their standard errors, and goodness-of-fit statistics, for each of the 36 simulated scenarios assuming the mean structure from Model 3 ($y_{itk} = \beta_0 + \partial x_{tk} + \kappa_t + v_{0ik} + \epsilon_{itk}$) with compound symmetric covariance structure between within subject measurement, together with the Monte Carlo standard error of each estimate in brackets. Each scenario was simulated 1000 times.

				Treatment Effect at 6 months						Time Averaged Treatment Effect					
Scenario	BIC	MSE	Proportion Significant Intervention Parameter	True Value	Estimate	Estimate SE	Coverage of 95% C.I.	Bias	Confidence Interval Width	True Value	Estimate	Estimate SE	Coverage of 95% C.I.	Bias	Confidence Interval Width
D1	19557.6 (2.7)	4.9 (0)	0.05	0	-0.01 (0.052)	0.3 (0.0001)	94.8%	-0.01 (0.0096)	1.18 (0.0006)	0	-0.01 (0.0096)	0.3 (0.0001)	94.8%	-0.01 (0.0096)	1.18 (0.0006)
D2	19152.5 (2.6)	4.6 (0)	0.05	0	0 (0.047)	0.28 (0.0001)	95.2%	0 (0.0087)	1.1 (0.0005)	0	0 (0.0087)	0.28 (0.0001)	95.2%	0 (0.0087)	1.1 (0.0005)
D3	19551.6 (2.7)	4.9 (0)	0.05	0	-0.01 (0.051)	0.3 (0.0001)	94.9%	-0.01 (0.0096)	1.17 (0.0005)	0	-0.01 (0.0096)	0.3 (0.0001)	94.9%	-0.01 (0.0096)	1.17 (0.0005)
D4	19147.2 (2.6)	4.6 (0)	0.04	0	0 (0.044)	0.28 (0.0001)	95.6%	0 (0.0087)	1.09 (0.0005)	0	0 (0.0087)	0.28 (0.0001)	95.6%	0 (0.0087)	1.09 (0.0005)
D5	19551.6 (2.7)	4.9 (0)	0.05	0	-0.01 (0.051)	0.3 (0.0001)	94.9%	-0.01 (0.0096)	1.17 (0.0005)	0	-0.01 (0.0096)	0.3 (0.0001)	94.9%	-0.01 (0.0096)	1.17 (0.0005)
D6	19147.2 (2.6)	4.6 (0)	0.04	0	0 (0.044)	0.28 (0.0001)	95.6%	0 (0.0087)	1.09 (0.0005)	0	0 (0.0087)	0.28 (0.0001)	95.6%	0 (0.0087)	1.09 (0.0005)
D7	19551.6 (2.7)	4.9 (0)	0.05	0	-0.01 (0.051)	0.3 (0.0001)	94.9%	-0.01 (0.0096)	1.17 (0.0005)	0	-0.01 (0.0096)	0.3 (0.0001)	94.9%	-0.01 (0.0096)	1.17 (0.0005)
D8	19147.2 (2.6)	4.6 (0)	0.04	0	0 (0.044)	0.28 (0.0001)	95.6%	0 (0.0087)	1.09 (0.0005)	0	0 (0.0087)	0.28 (0.0001)	95.6%	0 (0.0087)	1.09 (0.0005)
D9	19551.6 (2.7)	4.9 (0)	1	2	1.99 (1)	0.3 (0.0001)	94.9%	-0.01 (0.0096)	1.17 (0.0005)	2	1.99 (0.0096)	0.3 (0.0001)	94.9%	-0.01 (0.0096)	1.17 (0.0005)
D10	19147.2 (2.6)	4.6 (0)	1	2	2 (1)	0.28 (0.0001)	95.6%	0 (0.0087)	1.09 (0.0005)	2	2 (0.0087)	0.28 (0.0001)	95.6%	0 (0.0087)	1.09 (0.0005)
D11	19551.6 (2.7)	4.9 (0)	1	2	1.99 (1)	0.3 (0.0001)	94.9%	-0.01 (0.0096)	1.17 (0.0005)	2	1.99 (0.0096)	0.3 (0.0001)	94.9%	-0.01 (0.0096)	1.17 (0.0005)
D12	19147.2 (2.6)	4.6 (0)	1	2	2 (1)	0.28 (0.0001)	95.6%	0 (0.0087)	1.09 (0.0005)	2	2 (0.0087)	0.28 (0.0001)	95.6%	0 (0.0087)	1.09 (0.0005)
D13	19557 (2.7)	4.9 (0)	1	2.9	2.12 (1)	0.3 (0.0001)	26.1%	-0.78 (0.0096)	1.17 (0.0005)	2.9	2.12 (0.0096)	0.3 (0.0001)	26.1%	-0.78 (0.0096)	1.17 (0.0005)
D14	19153.5 (2.6)	4.6 (0)	1	2.9	2.12 (1)	0.28 (0.0001)	19.7%	-0.78 (0.0087)	1.09 (0.0005)	2.9	2.12 (0.0087)	0.28 (0.0001)	19.7%	-0.78 (0.0087)	1.09 (0.0005)
D15	19557 (2.7)	4.9 (0)	0.07	0.9	0.12 (0.071)	0.3 (0.0001)	26.1%	-0.78 (0.0096)	1.17 (0.0005)	0.9	0.12 (0.0096)	0.3 (0.0001)	26.1%	-0.78 (0.0096)	1.17 (0.0005)
D16	19153.5 (2.6)	4.6 (0)	0.07	0.9	0.12 (0.068)	0.28 (0.0001)	19.7%	-0.78 (0.0087)	1.09 (0.0005)	0.9	0.12 (0.0087)	0.28 (0.0001)	19.7%	-0.78 (0.0087)	1.09 (0.0005)
D17	19566.7 (2.7)	4.9 (0)	1	3.5	2.21 (1)	0.3 (0.0001)	1.1%	-1.29 (0.0096)	1.17 (0.0005)	3.5	2.21 (0.0096)	0.3 (0.0001)	1.1%	-1.29 (0.0096)	1.17 (0.0005)
D18	19164.6 (2.6)	4.6 (0)	1	3.5	2.19 (1)	0.28 (0.0001)	0.3%	-1.31 (0.0087)	1.1 (0.0005)	3.5	2.19 (0.0087)	0.28 (0.0001)	0.3%	-1.31 (0.0087)	1.1 (0.0005)
D19	19566.7 (2.7)	4.9 (0)	0.11	1.5	0.21 (0.11)	0.3 (0.0001)	1.1%	-1.29 (0.0096)	1.17 (0.0005)	1.5	0.21 (0.0096)	0.3 (0.0001)	1.1%	-1.29 (0.0096)	1.17 (0.0005)
D20	19164.6 (2.6)	4.6 (0)	0.11	1.5	0.19 (0.107)	0.28 (0.0001)	0.3%	-1.31 (0.0087)	1.1 (0.0005)	1.5	0.19 (0.0087)	0.28 (0.0001)	0.3%	-1.31 (0.0087)	1.1 (0.0005)
D21	19612 (2.6)	5 (0)	1	-5	-2.39 (1)	0.3 (0.0001)	0%	2.61 (0.0095)	1.19 (0.0006)	-5	-2.39 (0.0095)	0.3 (0.0001)	0%	2.61 (0.0095)	1.19 (0.0006)
D22	19215.4 (2.6)	4.6 (0)	1	-5	-2.36 (1)	0.28 (0.0001)	0%	2.64 (0.0089)	1.11 (0.0005)	-5	-2.36 (0.0089)	0.28 (0.0001)	0%	2.64 (0.0089)	1.11 (0.0005)
D23	19612 (2.6)	5 (0)	0.27	-3	-0.39 (0.268)	0.3 (0.0001)	0%	2.61 (0.0095)	1.19 (0.0006)	-3	-0.39 (0.0095)	0.3 (0.0001)	0%	2.61 (0.0095)	1.19 (0.0006)
D24	19215.4 (2.6)	4.6 (0)	0.26	-3	-0.36 (0.259)	0.28 (0.0001)	0%	2.64 (0.0089)	1.11 (0.0005)	-3	-0.36 (0.0089)	0.28 (0.0001)	0%	2.64 (0.0089)	1.11 (0.0005)
D25	19551.6 (2.7)	4.9 (0)	1	2	1.99 (1)	0.3 (0.0001)	94.9%	-0.01 (0.0096)	1.17 (0.0005)	2	1.99 (0.0096)	0.3 (0.0001)	94.9%	-0.01 (0.0096)	1.17 (0.0005)
D26	19147.2 (2.6)	4.6 (0)	1	2	2 (1)	0.28 (0.0001)	95.6%	0 (0.0087)	1.09 (0.0005)	2	2 (0.0087)	0.28 (0.0001)	95.6%	0 (0.0087)	1.09 (0.0005)
D27	19557.4 (2.7)	4.9 (0)	1	2.966	2.26 (1)	0.3 (0.0001)	34%	-0.71 (0.0096)	1.17 (0.0005)	2.58	2.26 (0.0096)	0.3 (0.0001)	80%	-0.32 (0.0096)	1.17 (0.0005)
D28	19154.1 (2.6)	4.6 (0)	1	2.966	2.26 (1)	0.28 (0.0001)	27.2%	-0.71 (0.0087)	1.09 (0.0005)	2.58	2.26 (0.0087)	0.28 (0.0001)	78.5%	-0.32 (0.0087)	1.09 (0.0005)
D29	19557.4 (2.7)	4.9 (0)	0.14	0.966	0.26 (0.141)	0.3 (0.0001)	34%	-0.71 (0.0096)	1.17 (0.0005)	0.58	0.26 (0.0096)	0.3 (0.0001)	80%	-0.32 (0.0096)	1.17 (0.0005)
D30	19154.1 (2.6)	4.6 (0)	0.15	0.966	0.26 (0.147)	0.28 (0.0001)	27.2%	-0.71 (0.0087)	1.09 (0.0005)	0.58	0.26 (0.0087)	0.28 (0.0001)	78.5%	-0.32 (0.0087)	1.09 (0.0005)
D31	19551.6 (2.7)	4.9 (0)	1	2	1.99 (1)	0.3 (0.0001)	94.9%	-0.01 (0.0096)	1.17 (0.0005)	2	1.99 (0.0096)	0.3 (0.0001)	94.9%	-0.01 (0.0096)	1.17 (0.0005)
D32	19147.2 (2.6)	4.6 (0)	1	2	2 (1)	0.28 (0.0001)	95.6%	0 (0.0087)	1.09 (0.0005)	2	2 (0.0087)	0.28 (0.0001)	95.6%	0 (0.0087)	1.09 (0.0005)
D33	19569.5 (2.7)	4.9 (0)	1	2.5	2.75 (1)	0.3 (0.0001)	86.5%	0.25 (0.0095)	1.17 (0.0005)	2	2.75 (0.0095)	0.3 (0.0001)	31.3%	0.75 (0.0095)	1.17 (0.0005)
D34	19167.7 (2.6)	4.6 (0)	1	2.5	2.77 (1)	0.28 (0.0001)	84.8%	0.27 (0.0087)	1.1 (0.0005)	2	2.77 (0.0087)	0.28 (0.0001)	22.7%	0.77 (0.0087)	1.1 (0.0005)
D35	19569.5 (2.7)	4.9 (0)	0.69	0.5	0.75 (0.687)	0.3 (0.0001)	86.5%	0.25 (0.0095)	1.17 (0.0005)	0	0.75 (0.0095)	0.3 (0.0001)	31.3%	0.75 (0.0095)	1.17 (0.0005)
D36	19167.7 (2.6)	4.6 (0)	0.77	0.5	0.77 (0.772)	0.28 (0.0001)	84.8%	0.27 (0.0087)	1.1 (0.0005)	0	0.77 (0.0087)	0.28 (0.0001)	22.7%	0.77 (0.0087)	1.1 (0.0005)

S3 Table D: The mean treatment effect estimates, their standard errors, and goodness-of-fit statistics, for each of the 36 simulated scenarios assuming the mean structure from Model 4 ($y_{itk} = \beta_0 + \partial x_{tk} + \tau t + \omega x_{tk} t + v_{0ik} + \epsilon_{itk}$) with compound symmetric covariance structure between within subject measurement, together with the Monte Carlo standard error of each estimate in brackets. Each scenario was simulated 1000 times.

				Treatment Effect at 6 months						Time Averaged Treatment Effect					
Scenario	BIC	MSE	Proportion Significant Intervention Parameter	True Value	Estimate	Estimate SE	Coverage of 95% C.I.	Bias	Confidence Interval Width	True Value	Estimate	Estimate SE	Coverage of 95% C.I.	Bias	Confidence Interval Width
D1	19486.8 (2.7)	4.9 (0)	0.07	0	-0.01 (0.073)	0.31 (0.0001)	94.7%	-0.01 (0.0097)	1.21 (0.0006)	0	-0.01 (0.0096)	0.3 (0.0001)	94.8%	-0.01 (0.0096)	1.18 (0.0006)
D2	19082.1 (2.6)	4.6 (0)	0.07	0	0 (0.065)	0.29 (0.0001)	95.1%	0 (0.0089)	1.12 (0.0005)	0	0 (0.0087)	0.28 (0.0001)	95.3%	0 (0.0087)	1.1 (0.0005)
D3	19480.8 (2.7)	4.9 (0)	0.08	0	-0.01 (0.075)	0.31 (0.0001)	95%	-0.01 (0.0097)	1.2 (0.0005)	0	-0.01 (0.0096)	0.3 (0.0001)	94.9%	-0.01 (0.0096)	1.17 (0.0005)
D4	19076.8 (2.6)	4.6 (0)	0.07	0	0 (0.067)	0.29 (0.0001)	95.2%	0 (0.0089)	1.12 (0.0005)	0	0 (0.0087)	0.28 (0.0001)	95.6%	0 (0.0087)	1.09 (0.0005)
D5	19508.9 (2.7)	5 (0)	1	0	0.35 (1)	0.31 (0.0001)	78.7%	0.35 (0.0097)	1.2 (0.0005)	0	-0.01 (0.0095)	0.3 (0.0001)	95%	-0.01 (0.0095)	1.18 (0.0005)
D6	19109.5 (2.6)	4.6 (0)	1	0	0.36 (1)	0.29 (0.0001)	76%	0.36 (0.0089)	1.12 (0.0005)	0	0 (0.0087)	0.28 (0.0001)	95.6%	0 (0.0087)	1.1 (0.0005)
D7	19606.7 (2.6)	5 (0)	0.06	0	-0.01 (0.064)	0.31 (0.0001)	95.7%	-0.01 (0.0097)	1.22 (0.0005)	0	-0.01 (0.0096)	0.3 (0.0001)	95.3%	-0.01 (0.0096)	1.2 (0.0005)
D8	19222.8 (2.5)	4.7 (0)	0.06	0	0 (0.058)	0.29 (0.0001)	95.7%	0 (0.0089)	1.14 (0.0005)	0	0 (0.0087)	0.29 (0.0001)	95.8%	0 (0.0087)	1.12 (0.0005)
D9	19480.8 (2.7)	4.9 (0)	0.99	2	1.99 (0.985)	0.31 (0.0001)	95%	-0.01 (0.0097)	1.2 (0.0005)	2	1.99 (0.0096)	0.3 (0.0001)	94.9%	-0.01 (0.0096)	1.17 (0.0005)
D10	19076.8 (2.6)	4.6 (0)	1	2	2 (0.995)	0.29 (0.0001)	95.2%	0 (0.0089)	1.12 (0.0005)	2	2 (0.0087)	0.28 (0.0001)	95.6%	0 (0.0087)	1.09 (0.0005)
D11	19480.8 (2.7)	4.9 (0)	0.99	2	1.99 (0.985)	0.31 (0.0001)	95%	-0.01 (0.0097)	1.2 (0.0005)	2	1.99 (0.0096)	0.3 (0.0001)	94.9%	-0.01 (0.0096)	1.17 (0.0005)
D12	19076.8 (2.6)	4.6 (0)	1	2	2 (0.995)	0.29 (0.0001)	95.2%	0 (0.0089)	1.12 (0.0005)	2	2 (0.0087)	0.28 (0.0001)	95.6%	0 (0.0087)	1.09 (0.0005)
D13	19484.8 (2.7)	4.9 (0)	0.97	2.9	2.03 (0.968)	0.31 (0.0001)	19.5%	-0.87 (0.0097)	1.2 (0.0005)	2.9	2.12 (0.0096)	0.3 (0.0001)	26.2%	-0.78 (0.0096)	1.17 (0.0005)
D14	19081.4 (2.6)	4.6 (0)	0.98	2.9	2.02 (0.984)	0.29 (0.0001)	13.4%	-0.88 (0.0089)	1.12 (0.0005)	2.9	2.12 (0.0087)	0.28 (0.0001)	19.7%	-0.78 (0.0087)	1.1 (0.0005)
D15	19484.8 (2.7)	4.9 (0)	0.33	0.9	0.03 (0.33)	0.31 (0.0001)	19.5%	-0.87 (0.0097)	1.2 (0.0005)	0.9	0.12 (0.0096)	0.3 (0.0001)	26.2%	-0.78 (0.0096)	1.17 (0.0005)
D16	19081.4 (2.6)	4.6 (0)	0.36	0.9	0.02 (0.355)	0.29 (0.0001)	13.4%	-0.88 (0.0089)	1.12 (0.0005)	0.9	0.12 (0.0087)	0.28 (0.0001)	19.7%	-0.78 (0.0087)	1.1 (0.0005)
D17	19492 (2.7)	4.9 (0)	0.98	3.5	2.05 (0.98)	0.31 (0.0001)	0.1%	-1.45 (0.0097)	1.2 (0.0005)	3.5	2.2 (0.0096)	0.3 (0.0001)	1.1%	-1.3 (0.0096)	1.18 (0.0005)
D18	19089.6 (2.6)	4.6 (0)	1	3.5	2.04 (0.996)	0.29 (0.0001)	0.1%	-1.46 (0.0089)	1.12 (0.0005)	3.5	2.19 (0.0087)	0.28 (0.0001)	0.3%	-1.31 (0.0087)	1.1 (0.0005)
D19	19492 (2.7)	4.9 (0)	0.7	1.5	0.05 (0.703)	0.31 (0.0001)	0.1%	-1.45 (0.0097)	1.2 (0.0005)	1.5	0.2 (0.0096)	0.3 (0.0001)	1.1%	-1.3 (0.0096)	1.18 (0.0005)
D20	19089.6 (2.6)	4.6 (0)	0.78	1.5	0.04 (0.775)	0.29 (0.0001)	0.1%	-1.46 (0.0089)	1.12 (0.0005)	1.5	0.19 (0.0087)	0.28 (0.0001)	0.3%	-1.31 (0.0087)	1.1 (0.0005)
D21	19525.5 (2.7)	5 (0)	1	-5	-2.08 (1)	0.31 (0.0001)	0%	2.92 (0.0097)	1.21 (0.0006)	-5	-2.39 (0.0095)	0.3 (0.0001)	0%	2.61 (0.0095)	1.19 (0.0006)
D22	19126.6 (2.6)	4.6 (0)	1	-5	-2.04 (1)	0.29 (0.0001)	0%	2.96 (0.0091)	1.13 (0.0005)	-5	-2.35 (0.0089)	0.28 (0.0001)	0%	2.65 (0.0089)	1.11 (0.0005)
D23	19525.5 (2.7)	5 (0)	1	-3	-0.08 (0.996)	0.31 (0.0001)	0%	2.92 (0.0097)	1.21 (0.0006)	-3	-0.39 (0.0095)	0.3 (0.0001)	0%	2.61 (0.0095)	1.19 (0.0006)
D24	19126.6 (2.6)	4.6 (0)	1	-3	-0.04 (1)	0.29 (0.0001)	0%	2.96 (0.0091)	1.13 (0.0005)	-3	-0.35 (0.0089)	0.28 (0.0001)	0%	2.65 (0.0089)	1.11 (0.0005)
D25	19508.9 (2.7)	5 (0)	1	2	2.35 (1)	0.31 (0.0001)	78.7%	0.35 (0.0097)	1.2 (0.0005)	2	1.99 (0.0095)	0.3 (0.0001)	95%	-0.01 (0.0095)	1.18 (0.0005)
D26	19109.5 (2.6)	4.6 (0)	1	2	2.36 (1)	0.29 (0.0001)	76%	0.36 (0.0089)	1.12 (0.0005)	2	2 (0.0087)	0.28 (0.0001)	95.6%	0 (0.0087)	1.1 (0.0005)
D27	19520 (2.7)	5 (0)	1	2.966	2.58 (1)	0.31 (0.0001)	74.4%	-0.39 (0.0097)	1.2 (0.0005)	2.58	2.26 (0.0096)	0.3 (0.0001)	80.3%	-0.32 (0.0096)	1.18 (0.0005)
D28	19122.3 (2.6)	4.6 (0)	1	2.966	2.58 (1)	0.29 (0.0001)	72.5%	-0.39 (0.0089)	1.13 (0.0005)	2.58	2.26 (0.0087)	0.28 (0.0001)	78.7%	-0.32 (0.0087)	1.1 (0.0005)
D29	19520 (2.7)	5 (0)	1	0.966	0.58 (0.999)	0.31 (0.0001)	74.4%	-0.39 (0.0097)	1.2 (0.0005)	0.58	0.26 (0.0096)	0.3 (0.0001)	80.3%	-0.32 (0.0096)	1.18 (0.0005)
D30	19122.3 (2.6)	4.6 (0)	1	0.966	0.58 (1)	0.29 (0.0001)	72.5%	-0.39 (0.0089)	1.13 (0.0005)	0.58	0.26 (0.0087)	0.28 (0.0001)	78.7%	-0.32 (0.0087)	1.1 (0.0005)
D31	19606.7 (2.6)	5 (0)	0.98	2	1.99 (0.977)	0.31 (0.0001)	95.7%	-0.01 (0.0097)	1.22 (0.0005)	2	1.99 (0.0096)	0.3 (0.0001)	95.3%	-0.01 (0.0096)	1.2 (0.0005)
D32	19222.8 (2.5)	4.7 (0)	0.99	2	2 (0.993)	0.29 (0.0001)	95.7%	0 (0.0089)	1.14 (0.0005)	2	2 (0.0087)	0.29 (0.0001)	95.8%	0 (0.0087)	1.12 (0.0005)
D33	19618.5 (2.6)	5.1 (0)	1	2.5	2.83 (1)	0.31 (0.0001)	81%	0.33 (0.0097)	1.22 (0.0005)	2	2.75 (0.0095)	0.31 (0.0001)	32.6%	0.75 (0.0095)	1.2 (0.0005)
D34	19236.2 (2.6)	4.7 (0)	1	2.5	2.85 (1)	0.29 (0.0001)	78%	0.35 (0.0089)	1.15 (0.0005)	2	2.77 (0.0087)	0.29 (0.0001)	24.1%	0.77 (0.0087)	1.12 (0.0005)
D35	19618.5 (2.6)	5.1 (0)	0.7	0.5	0.83 (0.704)	0.31 (0.0001)	81%	0.33 (0.0097)	1.22 (0.0005)	0	0.75 (0.0095)	0.31 (0.0001)	32.6%	0.75 (0.0095)	1.2 (0.0005)
D36	19236.2 (2.6)	4.7 (0)	0.78	0.5	0.85 (0.784)	0.29 (0.0001)	78%	0.35 (0.0089)	1.15 (0.0005)	0	0.77 (0.0087)	0.29 (0.0001)	24.1%	0.77 (0.0087)	1.12 (0.0005)

S3 Table E: The mean treatment effect estimates, their standard errors, and goodness-of-fit statistics, for each of the 36 simulated scenarios assuming the mean structure from Model 5 ($y_{itk} = \beta_0 + \partial x_{tk} + \kappa_t + \varphi_t + v_{0ik} + \epsilon_{itk}$) with compound symmetric covariance structure between within subject measurement, together with the Monte Carlo standard error of each estimate in brackets. Each scenario was simulated 1000 times.

				Treatment Effect at 6 months						Time Averaged Treatment Effect					
Scenario	BIC	MSE	Proportion Significant Intervention Parameter	True Value	Estimate	Estimate SE	Coverage of 95% C.I.	Bias	Confidence Interval Width	True Value	Estimate	Estimate SE	Coverage of 95% C.I.	Bias	Confidence Interval Width
D1	19629 (2.7)	4.9 (0)	0.06	0	0.03 (0.055)	0.68 (0.0003)	97.6%	0.03 (0.0201)	2.67 (0.0012)	0	-0.01 (0.0101)	0.32 (0.0002)	95.4%	-0.01 (0.0101)	1.24 (0.0006)
D2	19222.5 (2.6)	4.6 (0)	0.11	0	0.04 (0.105)	0.63 (0.0003)	96.6%	0.04 (0.0197)	2.47 (0.0011)	0	-0.01 (0.0093)	0.29 (0.0001)	95.3%	-0.01 (0.0093)	1.15 (0.0005)
D3	19622.9 (2.7)	4.9 (0)	0.06	0	0.03 (0.055)	0.68 (0.0003)	97.9%	0.03 (0.0201)	2.66 (0.0012)	0	-0.01 (0.0101)	0.31 (0.0001)	95.2%	-0.01 (0.0101)	1.23 (0.0005)
D4	19217.2 (2.6)	4.6 (0)	0.1	0	0.04 (0.101)	0.63 (0.0003)	96.8%	0.04 (0.0197)	2.47 (0.0011)	0	-0.01 (0.0092)	0.29 (0.0001)	95.3%	-0.01 (0.0092)	1.15 (0.0005)
D5	19622.9 (2.7)	4.9 (0)	0.06	0	0.03 (0.055)	0.68 (0.0003)	97.9%	0.03 (0.0201)	2.66 (0.0012)	0	-0.01 (0.0101)	0.31 (0.0001)	95.2%	-0.01 (0.0101)	1.23 (0.0005)
D6	19217.2 (2.6)	4.6 (0)	0.1	0	0.04 (0.101)	0.63 (0.0003)	96.8%	0.04 (0.0197)	2.47 (0.0011)	0	-0.01 (0.0092)	0.29 (0.0001)	95.3%	-0.01 (0.0092)	1.15 (0.0005)
D7	19622.9 (2.7)	4.9 (0)	0.06	0	0.03 (0.055)	0.68 (0.0003)	97.9%	0.03 (0.0201)	2.66 (0.0012)	0	-0.01 (0.0101)	0.31 (0.0001)	95.2%	-0.01 (0.0101)	1.23 (0.0005)
D8	19217.2 (2.6)	4.6 (0)	0.1	0	0.04 (0.101)	0.63 (0.0003)	96.8%	0.04 (0.0197)	2.47 (0.0011)	0	-0.01 (0.0092)	0.29 (0.0001)	95.3%	-0.01 (0.0092)	1.15 (0.0005)
D9	19622.9 (2.7)	4.9 (0)	0.39	2	2.03 (0.387)	0.68 (0.0003)	97.9%	0.03 (0.0201)	2.66 (0.0012)	2	1.99 (0.0101)	0.31 (0.0001)	95.2%	-0.01 (0.0101)	1.23 (0.0005)
D10	19217.2 (2.6)	4.6 (0)	0.46	2	2.04 (0.462)	0.63 (0.0003)	96.8%	0.04 (0.0197)	2.47 (0.0011)	2	1.99 (0.0092)	0.29 (0.0001)	95.3%	-0.01 (0.0092)	1.15 (0.0005)
D11	19622.9 (2.7)	4.9 (0)	0.39	2	2.03 (0.387)	0.68 (0.0003)	97.9%	0.03 (0.0201)	2.66 (0.0012)	2	1.99 (0.0101)	0.31 (0.0001)	95.2%	-0.01 (0.0101)	1.23 (0.0005)
D12	19217.2 (2.6)	4.6 (0)	0.46	2	2.04 (0.462)	0.63 (0.0003)	96.8%	0.04 (0.0197)	2.47 (0.0011)	2	1.99 (0.0092)	0.29 (0.0001)	95.3%	-0.01 (0.0092)	1.15 (0.0005)
D13	19626.9 (2.7)	4.9 (0)	0.29	2.9	2.05 (0.286)	0.68 (0.0003)	75.1%	-0.85 (0.0201)	2.66 (0.0012)	2.9	2.17 (0.0101)	0.31 (0.0001)	36.8%	-0.73 (0.0101)	1.23 (0.0006)
D14	19221.8 (2.6)	4.6 (0)	0.37	2.9	2.04 (0.374)	0.63 (0.0003)	71%	-0.86 (0.0197)	2.47 (0.0011)	2.9	2.17 (0.0092)	0.29 (0.0001)	29.7%	-0.73 (0.0092)	1.15 (0.0005)
D15	19626.9 (2.7)	4.9 (0)	0.1	0.9	0.05 (0.095)	0.68 (0.0003)	75.1%	-0.85 (0.0201)	2.66 (0.0012)	0.9	0.17 (0.0101)	0.31 (0.0001)	36.8%	-0.73 (0.0101)	1.23 (0.0006)
D16	19221.8 (2.6)	4.6 (0)	0.17	0.9	0.04 (0.165)	0.63 (0.0003)	71%	-0.86 (0.0197)	2.47 (0.0011)	0.9	0.17 (0.0092)	0.29 (0.0001)	29.7%	-0.73 (0.0092)	1.15 (0.0005)
D17	19634 (2.7)	4.9 (0)	0.32	3.5	2.05 (0.315)	0.68 (0.0003)	42.8%	-1.45 (0.0201)	2.67 (0.0012)	3.5	2.29 (0.0101)	0.31 (0.0001)	3.4%	-1.21 (0.0101)	1.23 (0.0006)
D18	19229.8 (2.6)	4.6 (0)	0.43	3.5	2.04 (0.434)	0.63 (0.0003)	37.4%	-1.46 (0.0197)	2.48 (0.0011)	3.5	2.28 (0.0093)	0.29 (0.0001)	1.5%	-1.22 (0.0093)	1.15 (0.0005)
D19	19634 (2.7)	4.9 (0)	0.21	1.5	0.05 (0.208)	0.68 (0.0003)	42.8%	-1.45 (0.0201)	2.67 (0.0012)	1.5	0.29 (0.0101)	0.31 (0.0001)	3.4%	-1.21 (0.0101)	1.23 (0.0006)
D20	19229.8 (2.6)	4.6 (0)	0.32	1.5	0.04 (0.321)	0.63 (0.0003)	37.4%	-1.46 (0.0197)	2.48 (0.0011)	1.5	0.28 (0.0093)	0.29 (0.0001)	1.5%	-1.22 (0.0093)	1.15 (0.0005)
D21	19666.8 (2.7)	4.9 (0)	0.8	-5	-1.97 (0.802)	0.68 (0.0003)	0.3%	3.03 (0.02)	2.68 (0.0012)	-5	-2.58 (0.0101)	0.32 (0.0002)	0%	2.42 (0.0101)	1.25 (0.0006)
D22	19266 (2.6)	4.6 (0)	0.9	-5	-1.93 (0.898)	0.64 (0.0003)	0.2%	3.07 (0.0197)	2.49 (0.0011)	-5	-2.55 (0.0094)	0.3 (0.0001)	0%	2.45 (0.0094)	1.16 (0.0005)
D23	19666.8 (2.7)	4.9 (0)	0.81	-3	0.03 (0.805)	0.68 (0.0003)	0.3%	3.03 (0.02)	2.68 (0.0012)	-3	-0.58 (0.0101)	0.32 (0.0002)	0%	2.42 (0.0101)	1.25 (0.0006)
D24	19266 (2.6)	4.6 (0)	0.9	-3	0.07 (0.897)	0.64 (0.0003)	0.2%	3.07 (0.0197)	2.49 (0.0011)	-3	-0.55 (0.0094)	0.3 (0.0001)	0%	2.45 (0.0094)	1.16 (0.0005)
D25	19622.9 (2.7)	4.9 (0)	0.39	2	2.03 (0.387)	0.68 (0.0003)	97.9%	0.03 (0.0201)	2.66 (0.0012)	2	1.99 (0.0101)	0.31 (0.0001)	95.2%	-0.01 (0.0101)	1.23 (0.0005)
D26	19217.2 (2.6)	4.6 (0)	0.46	2	2.04 (0.462)	0.63 (0.0003)	96.8%	0.04 (0.0197)	2.47 (0.0011)	2	1.99 (0.0092)	0.29 (0.0001)	95.3%	-0.01 (0.0092)	1.15 (0.0005)
D27	19628 (2.7)	4.9 (0)	0.32	2.966	2.26 (0.315)	0.68 (0.0003)	80.9%	-0.71 (0.0201)	2.66 (0.0012)	2.58	2.28 (0.0101)	0.31 (0.0001)	84.2%	-0.3 (0.0101)	1.23 (0.0005)
D28	19223.1 (2.6)	4.6 (0)	0.41	2.966	2.26 (0.412)	0.63 (0.0003)	77.3%	-0.71 (0.0197)	2.47 (0.0011)	2.58	2.28 (0.0092)	0.29 (0.0001)	83.2%	-0.3 (0.0092)	1.15 (0.0005)
D29	19628 (2.7)	4.9 (0)	0.07	0.966	0.26 (0.068)	0.68 (0.0003)	80.9%	-0.71 (0.0201)	2.66 (0.0012)	0.58	0.28 (0.0101)	0.31 (0.0001)	84.2%	-0.3 (0.0101)	1.23 (0.0005)
D30	19223.1 (2.6)	4.6 (0)	0.13	0.966	0.26 (0.128)	0.63 (0.0003)	77.3%	-0.71 (0.0197)	2.47 (0.0011)	0.58	0.28 (0.0092)	0.29 (0.0001)	83.2%	-0.3 (0.0092)	1.15 (0.0005)
D31	19622.9 (2.7)	4.9 (0)	0.39	2	2.03 (0.387)	0.68 (0.0003)	97.9%	0.03 (0.0201)	2.66 (0.0012)	2	1.99 (0.0101)	0.31 (0.0001)	95.2%	-0.01 (0.0101)	1.23 (0.0005)
D32	19217.2 (2.6)	4.6 (0)	0.46	2	2.04 (0.462)	0.63 (0.0003)	96.8%	0.04 (0.0197)	2.47 (0.0011)	2	1.99 (0.0092)	0.29 (0.0001)	95.3%	-0.01 (0.0092)	1.15 (0.0005)
D33	19639.5 (2.7)	4.9 (0)	0.57	2.5	3.07 (0.573)	0.68 (0.0003)	87.7%	0.57 (0.0201)	2.67 (0.0012)	2	2.69 (0.0101)	0.31 (0.0001)	41.8%	0.69 (0.0101)	1.23 (0.0006)
D34	19236.2 (2.6)	4.6 (0)	0.66	2.5	3.09 (0.66)	0.63 (0.0003)	84%	0.59 (0.0197)	2.48 (0.0011)	2	2.7 (0.0093)	0.29 (0.0001)	34.9%	0.7 (0.0093)	1.15 (0.0005)
D35	19639.5 (2.7)	4.9 (0)	0.11	0.5	1.07 (0.112)	0.68 (0.0003)	87.7%	0.57 (0.0201)	2.67 (0.0012)	0	0.69 (0.0101)	0.31 (0.0001)	41.8%	0.69 (0.0101)	1.23 (0.0006)
D36	19236.2 (2.6)	4.6 (0)	0.19	0.5	1.09 (0.185)	0.63 (0.0003)	84%	0.59 (0.0197)	2.48 (0.0011)	0	0.7 (0.0093)	0.29 (0.0001)	34.9%	0.7 (0.0093)	1.15 (0.0005)

S3 Table F: The mean treatment effect estimates, their standard errors, and goodness-of-fit statistics, for each of the 36 simulated scenarios assuming the mean structure from Model 6 ($y_{itk} = \beta_0 + \partial x_{tk} + \tau t + \psi d_{tk} + v_{0ik} + \epsilon_{itk}$) with compound symmetric covariance structure between within subject measurement, together with the Monte Carlo standard error of each estimate in brackets. Each scenario was simulated 1000 times.

				Treatment Effect at 6 months						Time Averaged Treatment Effect					
Scenario	BIC	MSE	Proportion Significant Intervention Parameter	True Value	Estimate	Estimate SE	Coverage of 95% C.I.	Bias	Confidence Interval Width	True Value	Estimate	Estimate SE	Coverage of 95% C.I.	Bias	Confidence Interval Width
D1	19486.6 (2.7)	4.9 (0)	0.12	0	-0.01 (0.122)	0.44 (0.0003)	91.1%	-0.01 (0.0156)	1.71 (0.0012)	0	-0.01 (0.0156)	0.44 (0.0003)	91.1%	-0.01 (0.0156)	1.71 (0.0012)
D2	19082 (2.6)	4.6 (0)	0.09	0	0 (0.091)	0.41 (0.0002)	96.7%	0 (0.0121)	1.6 (0.001)	0	0 (0.0121)	0.41 (0.0002)	96.7%	0 (0.0121)	1.6 (0.001)
D3	19480.6 (2.7)	4.9 (0)	0.12	0	-0.01 (0.12)	0.43 (0.0002)	91.1%	-0.01 (0.0154)	1.68 (0.0009)	0	-0.01 (0.0154)	0.43 (0.0002)	91.1%	-0.01 (0.0154)	1.68 (0.0009)
D4	19076.7 (2.6)	4.6 (0)	0.09	0	0 (0.093)	0.4 (0.0002)	96.5%	0 (0.0119)	1.58 (0.0008)	0	0 (0.0119)	0.4 (0.0002)	96.5%	0 (0.0119)	1.58 (0.0008)
D5	19533.8 (2.7)	5 (0)	0.79	0	-0.85 (0.785)	0.43 (0.0003)	50.1%	-0.85 (0.0154)	1.69 (0.001)	0	-0.85 (0.0154)	0.43 (0.0003)	50.1%	-0.85 (0.0154)	1.69 (0.001)
D6	19138.6 (2.6)	4.6 (0)	0.85	0	-0.87 (0.848)	0.41 (0.0002)	42.7%	-0.87 (0.0119)	1.6 (0.0008)	0	-0.87 (0.0119)	0.41 (0.0002)	42.7%	-0.87 (0.0119)	1.6 (0.0008)
D7	19606.5 (2.6)	5 (0)	0.11	0	-0.01 (0.113)	0.44 (0.0002)	91.7%	-0.01 (0.0154)	1.71 (0.001)	0	-0.01 (0.0154)	0.44 (0.0002)	91.7%	-0.01 (0.0154)	1.71 (0.001)
D8	19222.8 (2.5)	4.7 (0)	0.08	0	0 (0.08)	0.41 (0.0002)	97.1%	0 (0.0119)	1.62 (0.0008)	0	0 (0.0119)	0.41 (0.0002)	97.1%	0 (0.0119)	1.62 (0.0008)
D9	19480.6 (2.7)	4.9 (0)	1	2	1.99 (1)	0.43 (0.0002)	91.1%	-0.01 (0.0154)	1.68 (0.0009)	2	1.99 (0.0154)	0.43 (0.0002)	91.1%	-0.01 (0.0154)	1.68 (0.0009)
D10	19076.7 (2.6)	4.6 (0)	1	2	2 (1)	0.4 (0.0002)	96.5%	0 (0.0119)	1.58 (0.0008)	2	2 (0.0119)	0.4 (0.0002)	96.5%	0 (0.0119)	1.58 (0.0008)
D11	19480.6 (2.7)	4.9 (0)	1	2	1.99 (1)	0.43 (0.0002)	91.1%	-0.01 (0.0154)	1.68 (0.0009)	2	1.99 (0.0154)	0.43 (0.0002)	91.1%	-0.01 (0.0154)	1.68 (0.0009)
D12	19076.7 (2.6)	4.6 (0)	1	2	2 (1)	0.4 (0.0002)	96.5%	0 (0.0119)	1.58 (0.0008)	2	2 (0.0119)	0.4 (0.0002)	96.5%	0 (0.0119)	1.58 (0.0008)
D13	19480.6 (2.7)	4.9 (0)	1	2.9	2.89 (1)	0.43 (0.0002)	91.1%	-0.01 (0.0154)	1.68 (0.0009)	2.9	2.89 (0.0154)	0.43 (0.0002)	91.1%	-0.01 (0.0154)	1.68 (0.0009)
D14	19076.7 (2.6)	4.6 (0)	1	2.9	2.9 (1)	0.4 (0.0002)	96.5%	0 (0.0119)	1.58 (0.0008)	2.9	2.9 (0.0119)	0.4 (0.0002)	96.5%	0 (0.0119)	1.58 (0.0008)
D15	19480.6 (2.7)	4.9 (0)	0.71	0.9	0.89 (0.706)	0.43 (0.0002)	91.1%	-0.01 (0.0154)	1.68 (0.0009)	0.9	0.89 (0.0154)	0.43 (0.0002)	91.1%	-0.01 (0.0154)	1.68 (0.0009)
D16	19076.7 (2.6)	4.6 (0)	0.78	0.9	0.9 (0.782)	0.4 (0.0002)	96.5%	0 (0.0119)	1.58 (0.0008)	0.9	0.9 (0.0119)	0.4 (0.0002)	96.5%	0 (0.0119)	1.58 (0.0008)
D17	19480.6 (2.7)	4.9 (0)	1	3.5	3.49 (1)	0.43 (0.0002)	91.1%	-0.01 (0.0154)	1.68 (0.0009)	3.5	3.49 (0.0154)	0.43 (0.0002)	91.1%	-0.01 (0.0154)	1.68 (0.0009)
D18	19076.7 (2.6)	4.6 (0)	1	3.5	3.5 (1)	0.4 (0.0002)	96.5%	0 (0.0119)	1.58 (0.0008)	3.5	3.5 (0.0119)	0.4 (0.0002)	96.5%	0 (0.0119)	1.58 (0.0008)
D19	19480.6 (2.7)	4.9 (0)	0.97	1.5	1.49 (0.974)	0.43 (0.0002)	91.1%	-0.01 (0.0154)	1.68 (0.0009)	1.5	1.49 (0.0154)	0.43 (0.0002)	91.1%	-0.01 (0.0154)	1.68 (0.0009)
D20	19076.7 (2.6)	4.6 (0)	1	1.5	1.5 (0.999)	0.4 (0.0002)	96.5%	0 (0.0119)	1.58 (0.0008)	1.5	1.5 (0.0119)	0.4 (0.0002)	96.5%	0 (0.0119)	1.58 (0.0008)
D21	19480.6 (2.7)	4.9 (0)	1	-5	-5.01 (1)	0.43 (0.0002)	91.1%	-0.01 (0.0154)	1.68 (0.0009)	-5	-5.01 (0.0154)	0.43 (0.0002)	91.1%	-0.01 (0.0154)	1.68 (0.0009)
D22	19076.7 (2.6)	4.6 (0)	1	-5	-5 (1)	0.4 (0.0002)	96.5%	0 (0.0119)	1.58 (0.0008)	-5	-5 (0.0119)	0.4 (0.0002)	96.5%	0 (0.0119)	1.58 (0.0008)
D23	19480.6 (2.7)	4.9 (0)	1	-3	-3.01 (1)	0.43 (0.0002)	91.1%	-0.01 (0.0154)	1.68 (0.0009)	-3	-3.01 (0.0154)	0.43 (0.0002)	91.1%	-0.01 (0.0154)	1.68 (0.0009)
D24	19076.7 (2.6)	4.6 (0)	1	-3	-3 (1)	0.4 (0.0002)	96.5%	0 (0.0119)	1.58 (0.0008)	-3	-3 (0.0119)	0.4 (0.0002)	96.5%	0 (0.0119)	1.58 (0.0008)
D25	19533.8 (2.7)	5 (0)	1	2	1.15 (1)	0.43 (0.0003)	50.1%	-0.85 (0.0154)	1.69 (0.001)	2	1.15 (0.0154)	0.43 (0.0003)	50.1%	-0.85 (0.0154)	1.69 (0.001)
D26	19138.6 (2.6)	4.6 (0)	1	2	1.13 (1)	0.41 (0.0002)	42.7%	-0.87 (0.0119)	1.6 (0.0008)	2	1.13 (0.0119)	0.41 (0.0002)	42.7%	-0.87 (0.0119)	1.6 (0.0008)
D27	19543.1 (2.7)	5 (0)	1	2.966	1.87 (1)	0.43 (0.0003)	30.6%	-1.09 (0.0154)	1.69 (0.001)	2.58	1.87 (0.0154)	0.43 (0.0003)	61.2%	-0.71 (0.0154)	1.69 (0.001)
D28	19149.3 (2.6)	4.6 (0)	1	2.966	1.85 (1)	0.41 (0.0002)	21.1%	-1.11 (0.0119)	1.6 (0.0008)	2.58	1.85 (0.0119)	0.41 (0.0002)	57.1%	-0.73 (0.0119)	1.6 (0.0008)
D29	19543.1 (2.7)	5 (0)	0.39	0.966	-0.13 (0.389)	0.43 (0.0003)	30.6%	-1.09 (0.0154)	1.69 (0.001)	0.58	-0.13 (0.0154)	0.43 (0.0003)	61.2%	-0.71 (0.0154)	1.69 (0.001)
D30	19149.3 (2.6)	4.6 (0)	0.4	0.966	-0.15 (0.395)	0.41 (0.0002)	21.1%	-1.11 (0.0119)	1.6 (0.0008)	0.58	-0.15 (0.0119)	0.41 (0.0002)	57.1%	-0.73 (0.0119)	1.6 (0.0008)
D31	19606.5 (2.6)	5 (0)	1	2	1.99 (1)	0.44 (0.0002)	91.7%	-0.01 (0.0154)	1.71 (0.001)	2	1.99 (0.0154)	0.44 (0.0002)	91.7%	-0.01 (0.0154)	1.71 (0.001)
D32	19222.8 (2.5)	4.7 (0)	1	2	2 (1)	0.41 (0.0002)	97.1%	0 (0.0119)	1.62 (0.0008)	2	2 (0.0119)	0.41 (0.0002)	97.1%	0 (0.0119)	1.62 (0.0008)
D33	19616.5 (2.6)	5.1 (0)	1	2.5	2.16 (1)	0.44 (0.0002)	84.8%	-0.34 (0.0154)	1.71 (0.001)	2	2.16 (0.0154)	0.44 (0.0002)	91.6%	0.16 (0.0154)	1.71 (0.001)
D34	19234.3 (2.6)	4.7 (0)	1	2.5	2.18 (1)	0.41 (0.0002)	89.6%	-0.32 (0.0119)	1.62 (0.0008)	2	2.18 (0.0119)	0.41 (0.0002)	95.3%	0.18 (0.0119)	1.62 (0.0008)
D35	19616.5 (2.6)	5.1 (0)	0.88	0.5	0.16 (0.881)	0.44 (0.0002)	84.8%	-0.34 (0.0154)	1.71 (0.001)	0	0.16 (0.0154)	0.44 (0.0002)	91.6%	0.16 (0.0154)	1.71 (0.001)
D36	19234.3 (2.6)	4.7 (0)	0.9	0.5	0.18 (0.895)	0.41 (0.0002)	89.6%	-0.32 (0.0119)	1.62 (0.0008)	0	0.18 (0.0119)	0.41 (0.0002)	95.3%	0.18 (0.0119)	1.62 (0.0008)

S3 Table G: The mean treatment effect estimates, their standard errors, and goodness-of-fit statistics, for each of the 36 simulated scenarios assuming the mean structure from Model 7 ($y_{itk} = \beta_0 + \tau t + \psi d_{tk} + v_{0ik} + \epsilon_{itk}$) with compound symmetric covariance structure between within subject measurement, together with the Monte Carlo standard error of each estimate in brackets. Each scenario was simulated 1000 times.

				Treatment Effect at 6 months						Time Averaged Treatment Effect					
Scenario	BIC	MSE	Proportion Significant Intervention Parameter	True Value	Estimate	Estimate SE	Coverage of 95% C.I.	Bias	Confidence Interval Width	True Value	Estimate	Estimate SE	Coverage of 95% C.I.	Bias	Confidence Interval Width
D1	19479.6 (2.7)	4.9 (0)	0.09	0	-0.01 (0.092)	0.36 (0.0002)	90.8%	-0.01 (0.0126)	1.4 (0.0009)	0	-0.01 (0.0126)	0.36 (0.0002)	90.8%	-0.01 (0.0126)	1.4 (0.0009)
D2	19075 (2.6)	4.6 (0)	0.04	0	0 (0.041)	0.34 (0.0002)	95.9%	0 (0.0101)	1.31 (0.0007)	0	0 (0.0101)	0.34 (0.0002)	95.9%	0 (0.0101)	1.31 (0.0007)
D3	19473.5 (2.7)	4.9 (0)	0.08	0	-0.01 (0.082)	0.35 (0.0002)	91.8%	-0.01 (0.0124)	1.38 (0.0007)	0	-0.01 (0.0124)	0.35 (0.0002)	91.8%	-0.01 (0.0124)	1.38 (0.0007)
D4	19069.7 (2.6)	4.6 (0)	0.04	0	0 (0.035)	0.33 (0.0002)	96.4%	0 (0.0099)	1.3 (0.0006)	0	0 (0.0099)	0.33 (0.0002)	96.4%	0 (0.0099)	1.3 (0.0006)
D5	19526.9 (2.7)	5 (0)	0.76	0	-0.96 (0.761)	0.36 (0.0002)	23.8%	-0.96 (0.0124)	1.4 (0.0007)	0	-0.96 (0.0124)	0.36 (0.0002)	23.8%	-0.96 (0.0124)	1.4 (0.0007)
D6	19131.7 (2.6)	4.6 (0)	0.84	0	-0.98 (0.844)	0.34 (0.0002)	15.6%	-0.98 (0.01)	1.32 (0.0006)	0	-0.98 (0.01)	0.34 (0.0002)	15.6%	-0.98 (0.01)	1.32 (0.0006)
D7	19599.4 (2.6)	5 (0)	0.08	0	-0.01 (0.081)	0.36 (0.0002)	91.9%	-0.01 (0.0124)	1.41 (0.0007)	0	-0.01 (0.0124)	0.36 (0.0002)	91.9%	-0.01 (0.0124)	1.41 (0.0007)
D8	19215.7 (2.5)	4.7 (0)	0.03	0	0 (0.031)	0.34 (0.0002)	96.9%	0 (0.0099)	1.33 (0.0006)	0	0 (0.0099)	0.34 (0.0002)	96.9%	0 (0.0099)	1.33 (0.0006)
D9	19516.3 (2.6)	5 (0)	0.21	2	0.39 (0.208)	0.36 (0.0002)	1.1%	-1.61 (0.0125)	1.4 (0.0008)	2	0.39 (0.0125)	0.36 (0.0002)	1.1%	-1.61 (0.0125)	1.4 (0.0008)
D10	19119.3 (2.6)	4.6 (0)	0.19	2	0.36 (0.187)	0.34 (0.0002)	0.1%	-1.64 (0.01)	1.31 (0.0006)	2	0.36 (0.01)	0.34 (0.0002)	0.1%	-1.64 (0.01)	1.31 (0.0006)
D11	19516.3 (2.6)	5 (0)	0.21	2	0.39 (0.208)	0.36 (0.0002)	1.1%	-1.61 (0.0125)	1.4 (0.0008)	2	0.39 (0.0125)	0.36 (0.0002)	1.1%	-1.61 (0.0125)	1.4 (0.0008)
D12	19119.3 (2.6)	4.6 (0)	0.19	2	0.36 (0.187)	0.34 (0.0002)	0.1%	-1.64 (0.01)	1.31 (0.0006)	2	0.36 (0.01)	0.34 (0.0002)	0.1%	-1.64 (0.01)	1.31 (0.0006)
D13	19516.3 (2.6)	5 (0)	0.93	2.9	1.29 (0.931)	0.36 (0.0002)	1.1%	-1.61 (0.0125)	1.4 (0.0008)	2.9	1.29 (0.0125)	0.36 (0.0002)	1.1%	-1.61 (0.0125)	1.4 (0.0008)
D14	19119.3 (2.6)	4.6 (0)	0.97	2.9	1.26 (0.971)	0.34 (0.0002)	0.1%	-1.64 (0.01)	1.31 (0.0006)	2.9	1.26 (0.01)	0.34 (0.0002)	0.1%	-1.64 (0.01)	1.31 (0.0006)
D15	19473.5 (2.7)	4.9 (0)	0.71	0.9	0.89 (0.707)	0.35 (0.0002)	91.8%	-0.01 (0.0124)	1.38 (0.0007)	0.9	0.89 (0.0124)	0.35 (0.0002)	91.8%	-0.01 (0.0124)	1.38 (0.0007)
D16	19069.7 (2.6)	4.6 (0)	0.79	0.9	0.9 (0.787)	0.33 (0.0002)	96.4%	0 (0.0099)	1.3 (0.0006)	0.9	0.9 (0.0099)	0.33 (0.0002)	96.4%	0 (0.0099)	1.3 (0.0006)
D17	19516.3 (2.6)	5 (0)	1	3.5	1.89 (0.997)	0.36 (0.0002)	1.1%	-1.61 (0.0125)	1.4 (0.0008)	3.5	1.89 (0.0125)	0.36 (0.0002)	1.1%	-1.61 (0.0125)	1.4 (0.0008)
D18	19119.3 (2.6)	4.6 (0)	1	3.5	1.86 (1)	0.34 (0.0002)	0.1%	-1.64 (0.01)	1.31 (0.0006)	3.5	1.86 (0.01)	0.34 (0.0002)	0.1%	-1.64 (0.01)	1.31 (0.0006)
D19	19473.5 (2.7)	4.9 (0)	0.97	1.5	1.49 (0.973)	0.35 (0.0002)	91.8%	-0.01 (0.0124)	1.38 (0.0007)	1.5	1.49 (0.0124)	0.35 (0.0002)	91.8%	-0.01 (0.0124)	1.38 (0.0007)
D20	19069.7 (2.6)	4.6 (0)	1	1.5	1.5 (0.999)	0.33 (0.0002)	96.4%	0 (0.0099)	1.3 (0.0006)	1.5	1.5 (0.0099)	0.33 (0.0002)	96.4%	0 (0.0099)	1.3 (0.0006)
D21	19516.7 (2.7)	5 (0)	1	-5	-3.4 (1)	0.36 (0.0002)	1.4%	1.6 (0.0124)	1.4 (0.0008)	-5	-3.4 (0.0124)	0.36 (0.0002)	1.4%	1.6 (0.0124)	1.4 (0.0008)
D22	19119.2 (2.6)	4.6 (0)	1	-5	-3.36 (1)	0.34 (0.0002)	0%	1.64 (0.01)	1.31 (0.0006)	-5	-3.36 (0.01)	0.34 (0.0002)	0%	1.64 (0.01)	1.31 (0.0006)
D23	19473.5 (2.7)	4.9 (0)	1	-3	-3.01 (1)	0.35 (0.0002)	91.8%	-0.01 (0.0124)	1.38 (0.0007)	-3	-3.01 (0.0124)	0.35 (0.0002)	91.8%	-0.01 (0.0124)	1.38 (0.0007)
D24	19069.7 (2.6)	4.6 (0)	1	-3	-3 (1)	0.33 (0.0002)	96.4%	0 (0.0099)	1.3 (0.0006)	-3	-3 (0.0099)	0.33 (0.0002)	96.4%	0 (0.0099)	1.3 (0.0006)
D25	19574.7 (2.7)	5 (0)	0.38	2	-0.6 (0.383)	0.36 (0.0002)	0%	-2.6 (0.0126)	1.42 (0.0008)	2	-0.6 (0.0126)	0.36 (0.0002)	0%	-2.6 (0.0126)	1.42 (0.0008)
D26	19186.3 (2.6)	4.7 (0)	0.47	2	-0.64 (0.47)	0.34 (0.0002)	0%	-2.64 (0.0102)	1.33 (0.0007)	2	-0.64 (0.0102)	0.34 (0.0002)	0%	-2.64 (0.0102)	1.33 (0.0007)
D27	19592.6 (2.6)	5 (0)	0.09	2.966	-0.03 (0.086)	0.36 (0.0002)	0%	-2.99 (0.0126)	1.42 (0.0009)	2.58	-0.03 (0.0126)	0.36 (0.0002)	0%	-2.61 (0.0126)	1.42 (0.0009)
D28	19206.9 (2.6)	4.7 (0)	0.04	2.966	-0.07 (0.04)	0.34 (0.0002)	0%	-3.04 (0.0102)	1.34 (0.0007)	2.58	-0.07 (0.0102)	0.34 (0.0002)	0%	-2.65 (0.0102)	1.34 (0.0007)
D29	19537.1 (2.7)	5 (0)	0.22	0.966	-0.39 (0.217)	0.36 (0.0002)	5.8%	-1.36 (0.0124)	1.4 (0.0007)	0.58	-0.39 (0.0124)	0.36 (0.0002)	23.4%	-0.97 (0.0124)	1.4 (0.0007)
D30	19143.5 (2.6)	4.6 (0)	0.22	0.966	-0.41 (0.215)	0.34 (0.0002)	1%	-1.37 (0.01)	1.32 (0.0006)	0.58	-0.41 (0.01)	0.34 (0.0002)	15.2%	-0.99 (0.01)	1.32 (0.0006)
D31	19640.5 (2.6)	5.1 (0)	0.2	2	0.39 (0.202)	0.36 (0.0002)	1.1%	-1.61 (0.0125)	1.42 (0.0008)	2	0.39 (0.0125)	0.36 (0.0002)	1.1%	-1.61 (0.0125)	1.42 (0.0008)
D32	19262.9 (2.5)	4.7 (0)	0.17	2	0.37 (0.174)	0.34 (0.0002)	0.1%	-1.63 (0.01)	1.34 (0.0007)	2	0.37 (0.01)	0.34 (0.0002)	0.1%	-1.63 (0.01)	1.34 (0.0007)
D33	19692.3 (2.6)	5.1 (0)	0.09	2.5	-0.13 (0.085)	0.37 (0.0002)	0%	-2.63 (0.0125)	1.44 (0.0008)	2	-0.13 (0.0125)	0.37 (0.0002)	0%	-2.13 (0.0125)	1.44 (0.0008)
D34	19322.2 (2.5)	4.8 (0)	0.06	2.5	-0.16 (0.06)	0.35 (0.0002)	0%	-2.66 (0.0101)	1.36 (0.0007)	2	-0.16 (0.0101)	0.35 (0.0002)	0%	-2.16 (0.0101)	1.36 (0.0007)
D35	19616.9 (2.6)	5.1 (0)	0.3	0.5	-0.52 (0.304)	0.36 (0.0002)	20.5%	-1.02 (0.0124)	1.41 (0.0007)	0	-0.52 (0.0124)	0.36 (0.0002)	69.6%	-0.52 (0.0124)	1.41 (0.0007)
D36	19235.9 (2.6)	4.7 (0)	0.32	0.5	-0.52 (0.324)	0.34 (0.0002)	13.8%	-1.02 (0.0099)	1.34 (0.0006)	0	-0.52 (0.0099)	0.34 (0.0002)	67.5%	-0.52 (0.0099)	1.34 (0.0006)

S3 Table H: The mean treatment effect estimates, their standard errors, and goodness-of-fit statistics, for each of the 36 simulated scenarios assuming the mean structure from Model 8 ($y_{itk} = \beta_0 + \kappa_t + \xi_d + v_{0ik} + \epsilon_{itk}$) with compound symmetric covariance structure between within subject measurement, together with the Monte Carlo standard error of each estimate in brackets. Each scenario was simulated 1000 times.

				Treatment Effect at 6 months						Time Averaged Treatment Effect					
Scenario	BIC	MSE	Proportion Significant Intervention Parameter	True Value	Estimate	Estimate SE	Coverage of 95% C.I.	Bias	Confidence Interval Width	True Value	Estimate	Estimate SE	Coverage of 95% C.I.	Bias	Confidence Interval Width
D1	19635.9 (2.7)	4.9 (0)	0.04	0	0 (0.043)	0.59 (0.0003)	93.3%	0 (0.0204)	2.32 (0.0013)	0	-0.02 (0.0174)	0.49 (0.0004)	91.4%	-0.02 (0.0174)	1.91 (0.0015)
D2	19229.6 (2.6)	4.6 (0)	0.04	0	0.01 (0.044)	0.55 (0.0003)	95.8%	0.01 (0.0175)	2.17 (0.0011)	0	0 (0.0135)	0.46 (0.0003)	96.9%	0 (0.0135)	1.8 (0.0011)
D3	19629.8 (2.7)	4.9 (0)	1	0	0 (1)	0.59 (0.0003)	93.6%	0 (0.0203)	2.3 (0.0011)	0	-0.02 (0.0171)	0.48 (0.0003)	91.4%	-0.02 (0.0171)	1.87 (0.0011)
D4	19224.3 (2.6)	4.6 (0)	1	0	0.02 (1)	0.55 (0.0002)	95.8%	0.02 (0.0174)	2.15 (0.0009)	0	0 (0.0131)	0.45 (0.0002)	96.9%	0 (0.0131)	1.77 (0.0009)
D5	19629.8 (2.7)	4.9 (0)	1	0	0 (1)	0.59 (0.0003)	93.6%	0 (0.0203)	2.3 (0.0011)	0	-0.02 (0.0171)	0.48 (0.0003)	91.4%	-0.02 (0.0171)	1.87 (0.0011)
D6	19224.3 (2.6)	4.6 (0)	1	0	0.02 (1)	0.55 (0.0002)	95.8%	0.02 (0.0174)	2.15 (0.0009)	0	0 (0.0131)	0.45 (0.0002)	96.9%	0 (0.0131)	1.77 (0.0009)
D7	19629.8 (2.7)	4.9 (0)	1	0	0 (1)	0.59 (0.0003)	93.6%	0 (0.0203)	2.3 (0.0011)	0	-0.02 (0.0171)	0.48 (0.0003)	91.4%	-0.02 (0.0171)	1.87 (0.0011)
D8	19224.3 (2.6)	4.6 (0)	1	0	0.02 (1)	0.55 (0.0002)	95.8%	0.02 (0.0174)	2.15 (0.0009)	0	0 (0.0131)	0.45 (0.0002)	96.9%	0 (0.0131)	1.77 (0.0009)
D9	19629.8 (2.7)	4.9 (0)	1	2	2 (1)	0.59 (0.0003)	93.6%	0 (0.0203)	2.3 (0.0011)	2	1.98 (0.0171)	0.48 (0.0003)	91.4%	-0.02 (0.0171)	1.87 (0.0011)
D10	19224.3 (2.6)	4.6 (0)	1	2	2.02 (1)	0.55 (0.0002)	95.8%	0.02 (0.0174)	2.15 (0.0009)	2	2 (0.0131)	0.45 (0.0002)	96.9%	0 (0.0131)	1.77 (0.0009)
D11	19629.8 (2.7)	4.9 (0)	1	2	2 (1)	0.59 (0.0003)	93.6%	0 (0.0203)	2.3 (0.0011)	2	1.98 (0.0171)	0.48 (0.0003)	91.4%	-0.02 (0.0171)	1.87 (0.0011)
D12	19224.3 (2.6)	4.6 (0)	1	2	2.02 (1)	0.55 (0.0002)	95.8%	0.02 (0.0174)	2.15 (0.0009)	2	2 (0.0131)	0.45 (0.0002)	96.9%	0 (0.0131)	1.77 (0.0009)
D13	19629.8 (2.7)	4.9 (0)	1	2.9	2.9 (1)	0.59 (0.0003)	93.6%	0 (0.0203)	2.3 (0.0011)	2.9	2.96 (0.0171)	0.48 (0.0003)	91.4%	0.06 (0.0171)	1.87 (0.0011)
D14	19224.3 (2.6)	4.6 (0)	1	2.9	2.92 (1)	0.55 (0.0002)	95.8%	0.02 (0.0174)	2.15 (0.0009)	2.9	2.97 (0.0131)	0.45 (0.0002)	96.9%	0.07 (0.0131)	1.77 (0.0009)
D15	19629.8 (2.7)	4.9 (0)	1	0.9	0.9 (1)	0.59 (0.0003)	93.6%	0 (0.0203)	2.3 (0.0011)	0.9	0.96 (0.0171)	0.48 (0.0003)	91.4%	0.06 (0.0171)	1.87 (0.0011)
D16	19224.3 (2.6)	4.6 (0)	1	0.9	0.92 (1)	0.55 (0.0002)	95.8%	0.02 (0.0174)	2.15 (0.0009)	0.9	0.97 (0.0131)	0.45 (0.0002)	96.9%	0.07 (0.0131)	1.77 (0.0009)
D17	19629.8 (2.7)	4.9 (0)	1	3.5	3.5 (1)	0.59 (0.0003)	93.6%	0 (0.0203)	2.3 (0.0011)	3.5	3.61 (0.0171)	0.48 (0.0003)	91.2%	0.11 (0.0171)	1.87 (0.0011)
D18	19224.3 (2.6)	4.6 (0)	1	3.5	3.52 (1)	0.55 (0.0002)	95.8%	0.02 (0.0174)	2.15 (0.0009)	3.5	3.62 (0.0131)	0.45 (0.0002)	96.5%	0.12 (0.0131)	1.77 (0.0009)
D19	19629.8 (2.7)	4.9 (0)	1	1.5	1.5 (1)	0.59 (0.0003)	93.6%	0 (0.0203)	2.3 (0.0011)	1.5	1.61 (0.0171)	0.48 (0.0003)	91.2%	0.11 (0.0171)	1.87 (0.0011)
D20	19224.3 (2.6)	4.6 (0)	1	1.5	1.52 (1)	0.55 (0.0002)	95.8%	0.02 (0.0174)	2.15 (0.0009)	1.5	1.62 (0.0131)	0.45 (0.0002)	96.5%	0.12 (0.0131)	1.77 (0.0009)
D21	19629.8 (2.7)	4.9 (0)	1	-5	-5 (1)	0.59 (0.0003)	93.6%	0 (0.0203)	2.3 (0.0011)	-5	-5.27 (0.0171)	0.48 (0.0003)	87.8%	-0.27 (0.0171)	1.87 (0.0011)
D22	19224.3 (2.6)	4.6 (0)	1	-5	-4.98 (1)	0.55 (0.0002)	95.8%	0.02 (0.0174)	2.15 (0.0009)	-5	-5.25 (0.0131)	0.45 (0.0002)	93%	-0.25 (0.0131)	1.77 (0.0009)
D23	19629.8 (2.7)	4.9 (0)	0.44	-3	-3 (0.442)	0.59 (0.0003)	93.6%	0 (0.0203)	2.3 (0.0011)	-3	-3.27 (0.0171)	0.48 (0.0003)	87.8%	-0.27 (0.0171)	1.87 (0.0011)
D24	19224.3 (2.6)	4.6 (0)	0.53	-3	-2.98 (0.525)	0.55 (0.0002)	95.8%	0.02 (0.0174)	2.15 (0.0009)	-3	-3.25 (0.0131)	0.45 (0.0002)	93%	-0.25 (0.0131)	1.77 (0.0009)
D25	19629.8 (2.7)	4.9 (0)	1	2	2 (1)	0.59 (0.0003)	93.6%	0 (0.0203)	2.3 (0.0011)	2	1.98 (0.0171)	0.48 (0.0003)	91.4%	-0.02 (0.0171)	1.87 (0.0011)
D26	19224.3 (2.6)	4.6 (0)	1	2	2.02 (1)	0.55 (0.0002)	95.8%	0.02 (0.0174)	2.15 (0.0009)	2	2 (0.0131)	0.45 (0.0002)	96.9%	0 (0.0131)	1.77 (0.0009)
D27	19629.8 (2.7)	4.9 (0)	1	2.966	2.97 (1)	0.59 (0.0003)	93.6%	0 (0.0203)	2.3 (0.0011)	2.58	2.61 (0.0171)	0.48 (0.0003)	91.3%	0.03 (0.0171)	1.87 (0.0011)
D28	19224.3 (2.6)	4.6 (0)	1	2.966	2.98 (1)	0.55 (0.0002)	95.8%	0.02 (0.0174)	2.15 (0.0009)	2.58	2.63 (0.0131)	0.45 (0.0002)	96.8%	0.05 (0.0131)	1.77 (0.0009)
D29	19629.8 (2.7)	4.9 (0)	1	0.966	0.97 (1)	0.59 (0.0003)	93.6%	0 (0.0203)	2.3 (0.0011)	0.58	0.61 (0.0171)	0.48 (0.0003)	91.3%	0.03 (0.0171)	1.87 (0.0011)
D30	19224.3 (2.6)	4.6 (0)	1	0.966	0.98 (1)	0.55 (0.0002)	95.8%	0.02 (0.0174)	2.15 (0.0009)	0.58	0.63 (0.0131)	0.45 (0.0002)	96.8%	0.05 (0.0131)	1.77 (0.0009)
D31	19629.8 (2.7)	4.9 (0)	1	2	2 (1)	0.59 (0.0003)	93.6%	0 (0.0203)	2.3 (0.0011)	2	1.98 (0.0171)	0.48 (0.0003)	91.4%	-0.02 (0.0171)	1.87 (0.0011)
D32	19224.3 (2.6)	4.6 (0)	1	2	2.02 (1)	0.55 (0.0002)	95.8%	0.02 (0.0174)	2.15 (0.0009)	2	2 (0.0131)	0.45 (0.0002)	96.9%	0 (0.0131)	1.77 (0.0009)
D33	19629.8 (2.7)	4.9 (0)	1	2.5	2.5 (1)	0.59 (0.0003)	93.6%	0 (0.0203)	2.3 (0.0011)	2	1.98 (0.0171)	0.48 (0.0003)	91.4%	-0.02 (0.0171)	1.87 (0.0011)
D34	19224.3 (2.6)	4.6 (0)	1	2.5	2.52 (1)	0.55 (0.0002)	95.8%	0.02 (0.0174)	2.15 (0.0009)	2	2 (0.0131)	0.45 (0.0002)	96.9%	0 (0.0131)	1.77 (0.0009)
D35	19629.8 (2.7)	4.9 (0)	1	0.5	0.5 (1)	0.59 (0.0003)	93.6%	0 (0.0203)	2.3 (0.0011)	0	-0.02 (0.0171)	0.48 (0.0003)	91.4%	-0.02 (0.0171)	1.87 (0.0011)
D36	19224.3 (2.6)	4.6 (0)	1	0.5	0.52 (1)	0.55 (0.0002)	95.8%	0.02 (0.0174)	2.15 (0.0009)	0	0 (0.0131)	0.45 (0.0002)	96.9%	0 (0.0131)	1.77 (0.0009)

S3 Table I: The mean treatment effect estimates, their standard errors, and goodness-of-fit statistics, for each of the 36 simulated scenarios assuming the mean structure from Model 9 ($y_{itk} = \beta_0 + \partial x_{tk} + \tau t + \psi d_{tk} + \zeta t^2 + v_{0ik} + \epsilon_{itk}$) with compound symmetric covariance structure between within subject measurement, together with the Monte Carlo standard error of each estimate in brackets. Each scenario was simulated 1000 times.

				Treatment Effect at 6 months						Time Averaged Treatment Effect					
Scenario	BIC	MSE	Proportion Significant Intervention Parameter	True Value	Estimate	Estimate SE	Coverage of 95% C.I.	Bias	Confidence Interval Width	True Value	Estimate	Estimate SE	Coverage of 95% C.I.	Bias	Confidence Interval Width
D1	19493.6 (2.7)	4.9 (0)	0.13	0	-0.01 (0.129)	0.45 (0.0003)	91%	-0.01 (0.0162)	1.77 (0.0014)	0	-0.01 (0.0162)	0.45 (0.0003)	91%	-0.01 (0.0162)	1.77 (0.0014)
D2	19089.2 (2.6)	4.6 (0)	0.09	0	0 (0.093)	0.42 (0.0003)	96.4%	0 (0.0126)	1.67 (0.0011)	0	0 (0.0126)	0.42 (0.0003)	96.4%	0 (0.0126)	1.67 (0.0011)
D3	19487.5 (2.7)	4.9 (0)	0.12	0	-0.01 (0.12)	0.44 (0.0003)	91.2%	-0.01 (0.0159)	1.73 (0.001)	0	-0.01 (0.0159)	0.44 (0.0003)	91.2%	-0.01 (0.0159)	1.73 (0.001)
D4	19083.9 (2.6)	4.6 (0)	0.09	0	0 (0.089)	0.42 (0.0002)	96.5%	0 (0.0123)	1.64 (0.0008)	0	0 (0.0123)	0.42 (0.0002)	96.5%	0 (0.0123)	1.64 (0.0008)
D5	19487.8 (2.7)	4.9 (0)	0.12	0	-0.01 (0.12)	0.44 (0.0003)	91.2%	-0.01 (0.0159)	1.73 (0.001)	0	-0.01 (0.0159)	0.44 (0.0003)	91.2%	-0.01 (0.0159)	1.73 (0.001)
D6	19084.2 (2.6)	4.6 (0)	0.09	0	0 (0.089)	0.42 (0.0002)	96.5%	0 (0.0123)	1.64 (0.0008)	0	0 (0.0123)	0.42 (0.0002)	96.5%	0 (0.0123)	1.64 (0.0008)
D7	19613.5 (2.6)	5 (0)	0.11	0	-0.01 (0.114)	0.45 (0.0003)	91.7%	-0.01 (0.0159)	1.77 (0.001)	0	-0.01 (0.0159)	0.45 (0.0003)	91.7%	-0.01 (0.0159)	1.77 (0.001)
D8	19230 (2.5)	4.7 (0)	0.08	0	0 (0.077)	0.43 (0.0002)	97%	0 (0.0122)	1.68 (0.0009)	0	0 (0.0122)	0.43 (0.0002)	97%	0 (0.0122)	1.68 (0.0009)
D9	19487.5 (2.7)	4.9 (0)	1	2	1.99 (1)	0.44 (0.0003)	91.2%	-0.01 (0.0159)	1.73 (0.001)	2	1.99 (0.0159)	0.44 (0.0003)	91.2%	-0.01 (0.0159)	1.73 (0.001)
D10	19083.9 (2.6)	4.6 (0)	1	2	2 (1)	0.42 (0.0002)	96.5%	0 (0.0123)	1.64 (0.0008)	2	2 (0.0123)	0.42 (0.0002)	96.5%	0 (0.0123)	1.64 (0.0008)
D11	19487.5 (2.7)	4.9 (0)	1	2	1.99 (1)	0.44 (0.0003)	91.2%	-0.01 (0.0159)	1.73 (0.001)	2	1.99 (0.0159)	0.44 (0.0003)	91.2%	-0.01 (0.0159)	1.73 (0.001)
D12	19083.9 (2.6)	4.6 (0)	1	2	2 (1)	0.42 (0.0002)	96.5%	0 (0.0123)	1.64 (0.0008)	2	2 (0.0123)	0.42 (0.0002)	96.5%	0 (0.0123)	1.64 (0.0008)
D13	19487.5 (2.7)	4.9 (0)	1	2.9	2.89 (1)	0.44 (0.0003)	91.2%	-0.01 (0.0159)	1.73 (0.001)	2.9	2.89 (0.0159)	0.44 (0.0003)	91.2%	-0.01 (0.0159)	1.73 (0.001)
D14	19083.9 (2.6)	4.6 (0)	1	2.9	2.9 (1)	0.42 (0.0002)	96.5%	0 (0.0123)	1.64 (0.0008)	2.9	2.9 (0.0123)	0.42 (0.0002)	96.5%	0 (0.0123)	1.64 (0.0008)
D15	19487.5 (2.7)	4.9 (0)	0.65	0.9	0.89 (0.649)	0.44 (0.0003)	91.2%	-0.01 (0.0159)	1.73 (0.001)	0.9	0.89 (0.0159)	0.44 (0.0003)	91.2%	-0.01 (0.0159)	1.73 (0.001)
D16	19083.9 (2.6)	4.6 (0)	0.73	0.9	0.9 (0.734)	0.42 (0.0002)	96.5%	0 (0.0123)	1.64 (0.0008)	0.9	0.9 (0.0123)	0.42 (0.0002)	96.5%	0 (0.0123)	1.64 (0.0008)
D17	19487.5 (2.7)	4.9 (0)	1	3.5	3.49 (1)	0.44 (0.0003)	91.2%	-0.01 (0.0159)	1.73 (0.001)	3.5	3.49 (0.0159)	0.44 (0.0003)	91.2%	-0.01 (0.0159)	1.73 (0.001)
D18	19083.9 (2.6)	4.6 (0)	1	3.5	3.5 (1)	0.42 (0.0002)	96.5%	0 (0.0123)	1.64 (0.0008)	3.5	3.5 (0.0123)	0.42 (0.0002)	96.5%	0 (0.0123)	1.64 (0.0008)
D19	19487.5 (2.7)	4.9 (0)	0.96	1.5	1.49 (0.958)	0.44 (0.0003)	91.2%	-0.01 (0.0159)	1.73 (0.001)	1.5	1.49 (0.0159)	0.44 (0.0003)	91.2%	-0.01 (0.0159)	1.73 (0.001)
D20	19083.9 (2.6)	4.6 (0)	0.99	1.5	1.5 (0.992)	0.42 (0.0002)	96.5%	0 (0.0123)	1.64 (0.0008)	1.5	1.5 (0.0123)	0.42 (0.0002)	96.5%	0 (0.0123)	1.64 (0.0008)
D21	19487.5 (2.7)	4.9 (0)	1	-5	-5.01 (1)	0.44 (0.0003)	91.2%	-0.01 (0.0159)	1.73 (0.001)	-5	-5.01 (0.0159)	0.44 (0.0003)	91.2%	-0.01 (0.0159)	1.73 (0.001)
D22	19083.9 (2.6)	4.6 (0)	1	-5	-5 (1)	0.42 (0.0002)	96.5%	0 (0.0123)	1.64 (0.0008)	-5	-5 (0.0123)	0.42 (0.0002)	96.5%	0 (0.0123)	1.64 (0.0008)
D23	19487.5 (2.7)	4.9 (0)	1	-3	-3.01 (1)	0.44 (0.0003)	91.2%	-0.01 (0.0159)	1.73 (0.001)	-3	-3.01 (0.0159)	0.44 (0.0003)	91.2%	-0.01 (0.0159)	1.73 (0.001)
D24	19083.9 (2.6)	4.6 (0)	1	-3	-3 (1)	0.42 (0.0002)	96.5%	0 (0.0123)	1.64 (0.0008)	-3	-3 (0.0123)	0.42 (0.0002)	96.5%	0 (0.0123)	1.64 (0.0008)
D25	19487.8 (2.7)	4.9 (0)	1	2	1.99 (1)	0.44 (0.0003)	91.2%	-0.01 (0.0159)	1.73 (0.001)	2	1.99 (0.0159)	0.44 (0.0003)	91.2%	-0.01 (0.0159)	1.73 (0.001)
D26	19084.2 (2.6)	4.6 (0)	1	2	2 (1)	0.42 (0.0002)	96.5%	0 (0.0123)	1.64 (0.0008)	2	2 (0.0123)	0.42 (0.0002)	96.5%	0 (0.0123)	1.64 (0.0008)
D27	19491.4 (2.7)	4.9 (0)	1	2.966	2.76 (1)	0.44 (0.0003)	88.6%	-0.21 (0.0159)	1.73 (0.001)	2.58	2.76 (0.0159)	0.44 (0.0003)	89.8%	0.18 (0.0159)	1.73 (0.001)
D28	19088.5 (2.6)	4.6 (0)	1	2.966	2.78 (1)	0.42 (0.0002)	93.4%	-0.19 (0.0123)	1.64 (0.0008)	2.58	2.78 (0.0123)	0.42 (0.0002)	93.5%	0.2 (0.0123)	1.64 (0.0008)
D29	19491.4 (2.7)	4.9 (0)	0.4	0.966	0.76 (0.404)	0.44 (0.0003)	88.6%	-0.21 (0.0159)	1.73 (0.001)	0.58	0.76 (0.0159)	0.44 (0.0003)	89.8%	0.18 (0.0159)	1.73 (0.001)
D30	19088.5 (2.6)	4.6 (0)	0.45	0.966	0.78 (0.451)	0.42 (0.0002)	93.4%	-0.19 (0.0123)	1.64 (0.0008)	0.58	0.78 (0.0123)	0.42 (0.0002)	93.5%	0.2 (0.0123)	1.64 (0.0008)
D31	19613.5 (2.6)	5 (0)	1	2	1.99 (1)	0.45 (0.0003)	91.7%	-0.01 (0.0159)	1.77 (0.001)	2	1.99 (0.0159)	0.45 (0.0003)	91.7%	-0.01 (0.0159)	1.77 (0.001)
D32	19230 (2.5)	4.7 (0)	1	2	2 (1)	0.43 (0.0002)	97%	0 (0.0122)	1.68 (0.0009)	2	2 (0.0122)	0.43 (0.0002)	97%	0 (0.0122)	1.68 (0.0009)
D33	19623.2 (2.6)	5 (0)	1	2.5	2.22 (1)	0.45 (0.0003)	87.8%	-0.28 (0.0159)	1.77 (0.001)	2	2.22 (0.0159)	0.45 (0.0003)	88.9%	0.22 (0.0159)	1.77 (0.001)
D34	19241.2 (2.6)	4.7 (0)	1	2.5	2.24 (1)	0.43 (0.0002)	92.7%	-0.26 (0.0122)	1.68 (0.0009)	2	2.24 (0.0122)	0.43 (0.0002)	93%	0.24 (0.0122)	1.68 (0.0009)
D35	19623.2 (2.6)	5 (0)	0.85	0.5	0.22 (0.849)	0.45 (0.0003)	87.8%	-0.28 (0.0159)	1.77 (0.001)	0	0.22 (0.0159)	0.45 (0.0003)	88.9%	0.22 (0.0159)	1.77 (0.001)
D36	19241.2 (2.6)	4.7 (0)	0.87	0.5	0.24 (0.869)	0.43 (0.0002)	92.7%	-0.26 (0.0122)	1.68 (0.0009)	0	0.24 (0.0122)	0.43 (0.0002)	93%	0.24 (0.0122)	1.68 (0.0009)

S3 Table J: The mean treatment effect estimates, their standard errors, and goodness-of-fit statistics, for each of the 36 simulated scenarios assuming the mean structure from Model 1 ($y_{itk} = \beta_0 + \partial x_{tk} + v_{0ik} + \epsilon_{itk}$) with autoregressive (AR(1)) covariance structure between within subject measurement, together with the Monte Carlo standard error of each estimate in brackets. Each scenario was simulated 1000 times.

				Treatment Effect at 6 months						Time Averaged Treatment Effect					
Scenario	BIC	MSE	Proportion Significant Intervention Parameter	True Value	Estimate	Estimate SE	Coverage of 95% C.I.	Bias	Confidence Interval Width	True Value	Estimate	Estimate SE	Coverage of 95% C.I.	Bias	Confidence Interval Width
D1	19479.6 (2.7)	4.9 (0)	0.06	0	-0.01 (0.059)	0.21 (0.0001)	94%	-0.01 (0.0068)	0.82 (0.0005)	0	-0.01 (0.0068)	0.21 (0.0001)	94%	-0.01 (0.0068)	0.82 (0.0005)
D2	19074.8 (2.6)	4.6 (0)	0.05	0	0 (0.047)	0.19 (0.0001)	95.3%	0 (0.0059)	0.75 (0.0005)	0	0 (0.0059)	0.19 (0.0001)	95.3%	0 (0.0059)	0.75 (0.0005)
D3	19524.6 (2.6)	5 (0)	1	0	1.57 (1)	0.21 (0.0001)	0%	1.57 (0.0068)	0.83 (0.0005)	0	1.57 (0.0068)	0.21 (0.0001)	0%	1.57 (0.0068)	0.83 (0.0005)
D4	19129.6 (2.5)	4.6 (0)	1	0	1.59 (1)	0.19 (0.0001)	0%	1.59 (0.006)	0.76 (0.0005)	0	1.59 (0.006)	0.19 (0.0001)	0%	1.59 (0.006)	0.76 (0.0005)
D5	19532.3 (2.7)	5 (0)	0.06	0	-0.01 (0.055)	0.21 (0.0001)	94.5%	-0.01 (0.0068)	0.83 (0.0005)	0	-0.01 (0.0068)	0.21 (0.0001)	94.5%	-0.01 (0.0068)	0.83 (0.0005)
D6	19139.6 (2.6)	4.6 (0)	0.04	0	0 (0.039)	0.2 (0.0001)	96.1%	0 (0.0059)	0.77 (0.0005)	0	0 (0.0059)	0.2 (0.0001)	96.1%	0 (0.0059)	0.77 (0.0005)
D7	19637.2 (2.6)	5.1 (0)	1	0	-1.47 (1)	0.22 (0.0001)	0%	-1.47 (0.0069)	0.87 (0.0006)	0	-1.47 (0.0069)	0.22 (0.0001)	0%	-1.47 (0.0069)	0.87 (0.0006)
D8	19264.2 (2.6)	4.8 (0)	1	0	-1.5 (1)	0.21 (0.0001)	0%	-1.5 (0.006)	0.81 (0.0005)	0	-1.5 (0.006)	0.21 (0.0001)	0%	-1.5 (0.006)	0.81 (0.0005)
D9	19473.5 (2.7)	4.9 (0)	1	2	1.99 (1)	0.21 (0.0001)	94.1%	-0.01 (0.0068)	0.81 (0.0005)	2	1.99 (0.0068)	0.21 (0.0001)	94.1%	-0.01 (0.0068)	0.81 (0.0005)
D10	19069.4 (2.6)	4.6 (0)	1	2	2 (1)	0.19 (0.0001)	95.4%	0 (0.0059)	0.75 (0.0005)	2	2 (0.0059)	0.19 (0.0001)	95.4%	0 (0.0059)	0.75 (0.0005)
D11	19524.7 (2.6)	5 (0)	1	2	3.57 (0.999)	0.21 (0.0001)	0%	1.57 (0.0068)	0.83 (0.0005)	2	3.57 (0.0068)	0.21 (0.0001)	0%	1.57 (0.0068)	0.83 (0.0005)
D12	19129.6 (2.5)	4.6 (0)	1	2	3.59 (1)	0.19 (0.0001)	0%	1.59 (0.006)	0.76 (0.0005)	2	3.59 (0.006)	0.19 (0.0001)	0%	1.59 (0.006)	0.76 (0.0005)
D13	19561.9 (2.6)	5 (0)	1	2.9	4.13 (1)	0.21 (0.0001)	0%	1.23 (0.0068)	0.84 (0.0005)	2.9	4.13 (0.0068)	0.21 (0.0001)	0%	1.23 (0.0068)	0.84 (0.0005)
D14	19174.4 (2.5)	4.7 (0)	1	2.9	4.15 (1)	0.2 (0.0001)	0%	1.25 (0.006)	0.78 (0.0005)	2.9	4.15 (0.006)	0.2 (0.0001)	0%	1.25 (0.006)	0.78 (0.0005)
D15	19561.9 (2.6)	5 (0)	1	0.9	2.13 (1)	0.21 (0.0001)	0%	1.23 (0.0068)	0.84 (0.0005)	0.9	2.13 (0.0068)	0.21 (0.0001)	0%	1.23 (0.0068)	0.84 (0.0005)
D16	19174.4 (2.5)	4.7 (0)	1	0.9	2.15 (1)	0.2 (0.0001)	0%	1.25 (0.006)	0.78 (0.0005)	0.9	2.15 (0.006)	0.2 (0.0001)	0%	1.25 (0.006)	0.78 (0.0005)
D17	19596.5 (2.6)	5.1 (0)	1	3.5	4.5 (1)	0.22 (0.0001)	0.2%	1 (0.0068)	0.85 (0.0006)	3.5	4.5 (0.0068)	0.22 (0.0001)	0.2%	1 (0.0068)	0.85 (0.0006)
D18	19215.6 (2.5)	4.7 (0)	1	3.5	4.52 (1)	0.2 (0.0001)	0%	1.02 (0.006)	0.79 (0.0005)	3.5	4.52 (0.006)	0.2 (0.0001)	0%	1.02 (0.006)	0.79 (0.0005)
D19	19596.5 (2.6)	5.1 (0)	1	1.5	2.5 (1)	0.22 (0.0001)	0.2%	1 (0.0068)	0.85 (0.0006)	1.5	2.5 (0.0068)	0.22 (0.0001)	0.2%	1 (0.0068)	0.85 (0.0006)
D20	19215.6 (2.5)	4.7 (0)	1	1.5	2.52 (1)	0.2 (0.0001)	0%	1.02 (0.006)	0.79 (0.0005)	1.5	2.52 (0.006)	0.2 (0.0001)	0%	1.02 (0.006)	0.79 (0.0005)
D21	19539.6 (2.7)	5 (0)	1	-5	-2.32 (1)	0.21 (0.0001)	0%	2.68 (0.0068)	0.84 (0.0006)	-5	-2.32 (0.0068)	0.21 (0.0001)	0%	2.68 (0.0068)	0.84 (0.0006)
D22	19146.4 (2.6)	4.6 (0)	1	-5	-2.3 (1)	0.2 (0.0001)	0%	2.7 (0.006)	0.77 (0.0005)	-5	-2.3 (0.006)	0.2 (0.0001)	0%	2.7 (0.006)	0.77 (0.0005)
D23	19539.6 (2.7)	5 (0)	0.33	-3	-0.32 (0.327)	0.21 (0.0001)	0%	2.68 (0.0068)	0.84 (0.0006)	-3	-0.32 (0.0068)	0.21 (0.0001)	0%	2.68 (0.0068)	0.84 (0.0006)
D24	19146.4 (2.6)	4.6 (0)	0.32	-3	-0.3 (0.321)	0.2 (0.0001)	0%	2.7 (0.006)	0.77 (0.0005)	-3	-0.3 (0.006)	0.2 (0.0001)	0%	2.7 (0.006)	0.77 (0.0005)
D25	19532.3 (2.7)	5 (0)	1	2	1.99 (1)	0.21 (0.0001)	94.5%	-0.01 (0.0068)	0.83 (0.0005)	2	1.99 (0.0068)	0.21 (0.0001)	94.5%	-0.01 (0.0068)	0.83 (0.0005)
D26	19139.6 (2.6)	4.6 (0)	1	2	2 (1)	0.2 (0.0001)	96.1%	0 (0.0059)	0.77 (0.0005)	2	2 (0.0059)	0.2 (0.0001)	96.1%	0 (0.0059)	0.77 (0.0005)
D27	19536.7 (2.7)	5 (0)	1	2.966	2.5 (1)	0.21 (0.0001)	41.8%	-0.46 (0.0068)	0.83 (0.0005)	2.58	2.5 (0.0068)	0.21 (0.0001)	92.8%	-0.08 (0.0068)	0.83 (0.0005)
D28	19144.9 (2.6)	4.6 (0)	1	2.966	2.51 (1)	0.2 (0.0001)	36%	-0.46 (0.0059)	0.77 (0.0005)	2.58	2.51 (0.0059)	0.2 (0.0001)	93.5%	-0.07 (0.0059)	0.77 (0.0005)
D29	19536.7 (2.7)	5 (0)	0.66	0.966	0.5 (0.662)	0.21 (0.0001)	41.8%	-0.46 (0.0068)	0.83 (0.0005)	0.58	0.5 (0.0068)	0.21 (0.0001)	92.8%	-0.08 (0.0068)	0.83 (0.0005)
D30	19144.9 (2.6)	4.6 (0)	0.75	0.966	0.51 (0.749)	0.2 (0.0001)	36%	-0.46 (0.0059)	0.77 (0.0005)	0.58	0.51 (0.0059)	0.2 (0.0001)	93.5%	-0.07 (0.0059)	0.77 (0.0005)
D31	19637.2 (2.6)	5.1 (0)	0.67	2	0.53 (0.669)	0.22 (0.0001)	0%	-1.47 (0.0069)	0.87 (0.0006)	2	0.53 (0.0069)	0.22 (0.0001)	0%	-1.47 (0.0069)	0.87 (0.0006)
D32	19264.2 (2.6)	4.8 (0)	0.7	2	0.5 (0.696)	0.21 (0.0001)	0%	-1.5 (0.006)	0.81 (0.0005)	2	0.5 (0.006)	0.21 (0.0001)	0%	-1.5 (0.006)	0.81 (0.0005)
D33	19669 (2.6)	5.2 (0)	0.99	2.5	0.92 (0.985)	0.23 (0.0002)	0%	-1.58 (0.0069)	0.89 (0.0006)	2	0.92 (0.0069)	0.23 (0.0002)	0.2%	-1.08 (0.0069)	0.89 (0.0006)
D34	19302.5 (2.6)	4.8 (0)	0.99	2.5	0.89 (0.993)	0.21 (0.0001)	0%	-1.61 (0.006)	0.82 (0.0005)	2	0.89 (0.006)	0.21 (0.0001)	0%	-1.11 (0.006)	0.82 (0.0005)
D35	19669 (2.6)	5.2 (0)	1	0.5	-1.08 (0.998)	0.23 (0.0002)	0%	-1.58 (0.0069)	0.89 (0.0006)	0	-1.08 (0.0069)	0.23 (0.0002)	0.2%	-1.08 (0.0069)	0.89 (0.0006)
D36	19302.5 (2.6)	4.8 (0)	1	0.5	-1.11 (1)	0.21 (0.0001)	0%	-1.61 (0.006)	0.82 (0.0005)	0	-1.11 (0.006)	0.21 (0.0001)	0%	-1.11 (0.006)	0.82 (0.0005)

S3 Table K: The mean treatment effect estimates, their standard errors, and goodness-of-fit statistics, for each of the 36 simulated scenarios assuming the mean structure from Model 2 ($y_{itk} = \beta_0 + \partial x_{tk} + \tau t + v_{0ik} + \epsilon_{ijk}$) with autoregressive (AR(1)) covariance structure between within subject measurement, together with the Monte Carlo standard error of each estimate in brackets. Each scenario was simulated 1000 times.

				Treatment Effect at 6 months						Time Averaged Treatment Effect					
Scenario	BIC	MSE	Proportion Significant Intervention Parameter	True Value	Estimate	Estimate SE	Coverage of 95% C.I.	Bias	Confidence Interval Width	True Value	Estimate	Estimate SE	Coverage of 95% C.I.	Bias	Confidence Interval Width
D1	19486.6 (2.7)	4.9 (0)	0.05	0	-0.01 (0.052)	0.3 (0.0002)	94.8%	-0.01 (0.0096)	1.19 (0.0007)	0	-0.01 (0.0096)	0.3 (0.0002)	94.8%	-0.01 (0.0096)	1.19 (0.0007)
D2	19081.9 (2.6)	4.6 (0)	0.05	0	0 (0.05)	0.28 (0.0002)	95%	0 (0.0088)	1.1 (0.0006)	0	0 (0.0088)	0.28 (0.0002)	95%	0 (0.0088)	1.1 (0.0006)
D3	19480.5 (2.7)	4.9 (0)	0.05	0	-0.01 (0.05)	0.3 (0.0002)	94.8%	-0.01 (0.0096)	1.18 (0.0007)	0	-0.01 (0.0096)	0.3 (0.0002)	94.8%	-0.01 (0.0096)	1.18 (0.0007)
D4	19076.5 (2.6)	4.6 (0)	0.05	0	0 (0.047)	0.28 (0.0002)	95.2%	0 (0.0087)	1.09 (0.0006)	0	0 (0.0087)	0.28 (0.0002)	95.2%	0 (0.0087)	1.09 (0.0006)
D5	19539.3 (2.7)	5 (0)	0.04	0	-0.01 (0.044)	0.31 (0.0002)	95.6%	-0.01 (0.0096)	1.2 (0.0007)	0	-0.01 (0.0096)	0.31 (0.0002)	95.6%	-0.01 (0.0096)	1.2 (0.0007)
D6	19147 (2.6)	4.6 (0)	0.04	0	0 (0.041)	0.28 (0.0002)	95.8%	0 (0.0087)	1.11 (0.0006)	0	0 (0.0087)	0.28 (0.0002)	95.8%	0 (0.0087)	1.11 (0.0006)
D7	19601.8 (2.6)	5.1 (0)	0.04	0	-0.01 (0.042)	0.31 (0.0002)	95.8%	-0.01 (0.0096)	1.23 (0.0007)	0	-0.01 (0.0096)	0.31 (0.0002)	95.8%	-0.01 (0.0096)	1.23 (0.0007)
D8	19220.6 (2.5)	4.7 (0)	0.04	0	0 (0.04)	0.29 (0.0002)	96%	0 (0.0087)	1.14 (0.0006)	0	0 (0.0087)	0.29 (0.0002)	96%	0 (0.0087)	1.14 (0.0006)
D9	19480.6 (2.7)	4.9 (0)	1	2	1.99 (0.999)	0.3 (0.0002)	94.9%	-0.01 (0.0096)	1.18 (0.0007)	2	1.99 (0.0096)	0.3 (0.0002)	94.9%	-0.01 (0.0096)	1.18 (0.0007)
D10	19076.6 (2.6)	4.6 (0)	1	2	2 (1)	0.28 (0.0002)	95.3%	0 (0.0087)	1.09 (0.0006)	2	2 (0.0087)	0.28 (0.0002)	95.3%	0 (0.0087)	1.09 (0.0006)
D11	19480.5 (2.7)	4.9 (0)	1	2	1.99 (1)	0.3 (0.0002)	95%	-0.01 (0.0096)	1.18 (0.0007)	2	1.99 (0.0096)	0.3 (0.0002)	95%	-0.01 (0.0096)	1.18 (0.0007)
D12	19076.6 (2.6)	4.6 (0)	1	2	2 (1)	0.28 (0.0002)	95.3%	0 (0.0087)	1.09 (0.0006)	2	2 (0.0087)	0.28 (0.0002)	95.3%	0 (0.0087)	1.09 (0.0006)
D13	19486.6 (2.7)	4.9 (0)	1	2.9	2.12 (1)	0.3 (0.0002)	26.5%	-0.78 (0.0096)	1.18 (0.0007)	2.9	2.12 (0.0096)	0.3 (0.0002)	26.5%	-0.78 (0.0096)	1.18 (0.0007)
D14	19083.7 (2.6)	4.6 (0)	1	2.9	2.12 (0.999)	0.28 (0.0002)	19.5%	-0.78 (0.0087)	1.09 (0.0006)	2.9	2.12 (0.0087)	0.28 (0.0002)	19.5%	-0.78 (0.0087)	1.09 (0.0006)
D15	19486.6 (2.7)	4.9 (0)	0.07	0.9	0.12 (0.067)	0.3 (0.0002)	26.5%	-0.78 (0.0096)	1.18 (0.0007)	0.9	0.12 (0.0096)	0.3 (0.0002)	26.5%	-0.78 (0.0096)	1.18 (0.0007)
D16	19083.8 (2.6)	4.6 (0)	0.07	0.9	0.12 (0.07)	0.28 (0.0002)	19.6%	-0.78 (0.0087)	1.09 (0.0006)	0.9	0.12 (0.0087)	0.28 (0.0002)	19.6%	-0.78 (0.0087)	1.09 (0.0006)
D17	19497.3 (2.7)	5 (0)	1	3.5	2.21 (1)	0.3 (0.0002)	1.2%	-1.29 (0.0096)	1.19 (0.0007)	3.5	2.21 (0.0096)	0.3 (0.0002)	1.2%	-1.29 (0.0096)	1.19 (0.0007)
D18	19096.5 (2.6)	4.6 (0)	1	3.5	2.19 (1)	0.28 (0.0002)	0.3%	-1.31 (0.0087)	1.1 (0.0006)	3.5	2.19 (0.0087)	0.28 (0.0002)	0.3%	-1.31 (0.0087)	1.1 (0.0006)
D19	19497.3 (2.7)	5 (0)	0.11	1.5	0.21 (0.105)	0.3 (0.0002)	1.2%	-1.29 (0.0096)	1.19 (0.0007)	1.5	0.21 (0.0096)	0.3 (0.0002)	1.2%	-1.29 (0.0096)	1.19 (0.0007)
D20	19096.5 (2.6)	4.6 (0)	0.11	1.5	0.19 (0.109)	0.28 (0.0002)	0.3%	-1.31 (0.0087)	1.1 (0.0006)	1.5	0.19 (0.0087)	0.28 (0.0002)	0.3%	-1.31 (0.0087)	1.1 (0.0006)
D21	19546.5 (2.7)	5 (0)	1	-5	-2.4 (1)	0.31 (0.0002)	0%	2.6 (0.0095)	1.22 (0.0008)	-5	-2.4 (0.0095)	0.31 (0.0002)	0%	2.6 (0.0095)	1.22 (0.0008)
D22	19153.5 (2.6)	4.6 (0)	1	-5	-2.36 (1)	0.29 (0.0002)	0%	2.64 (0.0089)	1.12 (0.0006)	-5	-2.36 (0.0089)	0.29 (0.0002)	0%	2.64 (0.0089)	1.12 (0.0006)
D23	19546.5 (2.7)	5 (0)	0.26	-3	-0.4 (0.259)	0.31 (0.0002)	0%	2.6 (0.0095)	1.22 (0.0008)	-3	-0.4 (0.0095)	0.31 (0.0002)	0%	2.6 (0.0095)	1.22 (0.0008)
D24	19153.5 (2.6)	4.6 (0)	0.25	-3	-0.36 (0.253)	0.29 (0.0002)	0%	2.64 (0.0089)	1.12 (0.0006)	-3	-0.36 (0.0089)	0.29 (0.0002)	0%	2.64 (0.0089)	1.12 (0.0006)
D25	19539.1 (2.7)	5 (0)	1	2	1.99 (0.999)	0.31 (0.0002)	95.5%	-0.01 (0.0096)	1.2 (0.0007)	2	1.99 (0.0096)	0.31 (0.0002)	95.5%	-0.01 (0.0096)	1.2 (0.0007)
D26	19146.8 (2.6)	4.6 (0)	1	2	2 (1)	0.28 (0.0002)	95.9%	0 (0.0087)	1.11 (0.0006)	2	2 (0.0087)	0.28 (0.0002)	95.9%	0 (0.0087)	1.11 (0.0006)
D27	19542.5 (2.7)	5 (0)	1	2.966	2.26 (1)	0.31 (0.0002)	35.7%	-0.71 (0.0096)	1.2 (0.0007)	2.58	2.26 (0.0096)	0.31 (0.0002)	81.1%	-0.32 (0.0096)	1.2 (0.0007)
D28	19150.6 (2.6)	4.6 (0)	1	2.966	2.26 (1)	0.28 (0.0002)	29.1%	-0.71 (0.0087)	1.12 (0.0006)	2.58	2.26 (0.0087)	0.28 (0.0002)	78.8%	-0.32 (0.0087)	1.12 (0.0006)
D29	19542.5 (2.7)	5 (0)	0.13	0.966	0.26 (0.129)	0.31 (0.0002)	35.7%	-0.71 (0.0096)	1.2 (0.0007)	0.58	0.26 (0.0096)	0.31 (0.0002)	81.1%	-0.32 (0.0096)	1.2 (0.0007)
D30	19150.6 (2.6)	4.6 (0)	0.14	0.966	0.26 (0.141)	0.28 (0.0002)	29.1%	-0.71 (0.0087)	1.12 (0.0006)	0.58	0.26 (0.0087)	0.28 (0.0002)	78.8%	-0.32 (0.0087)	1.12 (0.0006)
D31	19601.8 (2.6)	5.1 (0)	1	2	1.99 (0.999)	0.31 (0.0002)	95.7%	-0.01 (0.0096)	1.23 (0.0007)	2	1.99 (0.0096)	0.31 (0.0002)	95.7%	-0.01 (0.0096)	1.23 (0.0007)
D32	19220.6 (2.5)	4.7 (0)	1	2	2 (1)	0.29 (0.0002)	96%	0 (0.0087)	1.14 (0.0006)	2	2 (0.0087)	0.29 (0.0002)	96%	0 (0.0087)	1.14 (0.0006)
D33	19614.6 (2.6)	5.1 (0)	1	2.5	2.71 (1)	0.31 (0.0002)	90.9%	0.21 (0.0096)	1.23 (0.0007)	2	2.71 (0.0096)	0.31 (0.0002)	38.2%	0.71 (0.0096)	1.23 (0.0007)
D34	19235.4 (2.6)	4.7 (0)	1	2.5	2.74 (1)	0.29 (0.0002)	87.5%	0.24 (0.0087)	1.14 (0.0006)	2	2.74 (0.0087)	0.29 (0.0002)	27.5%	0.74 (0.0087)	1.14 (0.0006)
D35	19614.5 (2.6)	5.1 (0)	0.62	0.5	0.71 (0.617)	0.31 (0.0002)	90.8%	0.21 (0.0096)	1.23 (0.0007)	0	0.71 (0.0096)	0.31 (0.0002)	38.2%	0.71 (0.0096)	1.23 (0.0007)
D36	19235.4 (2.6)	4.7 (0)	0.72	0.5	0.74 (0.723)	0.29 (0.0002)	87.5%	0.24 (0.0087)	1.14 (0.0006)	0	0.74 (0.0087)	0.29 (0.0002)	27.5%	0.74 (0.0087)	1.14 (0.0006)

S3 Table L: The mean treatment effect estimates, their standard errors, and goodness-of-fit statistics, for each of the 36 simulated scenarios assuming the mean structure from Model 3 ($y_{itk} = \beta_0 + \partial x_{tk} + \kappa_t + v_{0ik} + \epsilon_{itk}$) with autoregressive (AR(1)) covariance structure between within subject measurement, together with the Monte Carlo standard error of each estimate in brackets. Each scenario was simulated 1000 times.

				Treatment Effect at 6 months						Time Averaged Treatment Effect					
Scenario	BIC	MSE	Proportion Significant Intervention Parameter	True Value	Estimate	Estimate SE	Coverage of 95% C.I.	Bias	Confidence Interval Width	True Value	Estimate	Estimate SE	Coverage of 95% C.I.	Bias	Confidence Interval Width
D1	19564.4 (2.7)	4.9 (0)	0.05	0	-0.01 (0.052)	0.3 (0.0002)	94.7%	-0.01 (0.0096)	1.19 (0.0007)	0	-0.01 (0.0096)	0.3 (0.0002)	94.7%	-0.01 (0.0096)	1.19 (0.0007)
D2	19159.5 (2.6)	4.6 (0)	0.05	0	0 (0.049)	0.28 (0.0002)	95%	0 (0.0088)	1.09 (0.0006)	0	0 (0.0088)	0.28 (0.0002)	95%	0 (0.0088)	1.09 (0.0006)
D3	19558.4 (2.7)	4.9 (0)	0.05	0	-0.01 (0.05)	0.3 (0.0002)	95%	-0.01 (0.0096)	1.18 (0.0007)	0	-0.01 (0.0096)	0.3 (0.0002)	95%	-0.01 (0.0096)	1.18 (0.0007)
D4	19154.2 (2.6)	4.6 (0)	0.05	0	0 (0.047)	0.28 (0.0002)	95.3%	0 (0.0087)	1.09 (0.0006)	0	0 (0.0087)	0.28 (0.0002)	95.3%	0 (0.0087)	1.09 (0.0006)
D5	19558.4 (2.7)	4.9 (0)	0.05	0	0 (0.05)	0.3 (0.0002)	94.9%	0 (0.0096)	1.18 (0.0007)	0	0 (0.0096)	0.3 (0.0002)	94.9%	0 (0.0096)	1.18 (0.0007)
D6	19154.2 (2.6)	4.6 (0)	0.05	0	0 (0.047)	0.28 (0.0002)	95.3%	0 (0.0087)	1.09 (0.0006)	0	0 (0.0087)	0.28 (0.0002)	95.3%	0 (0.0087)	1.09 (0.0006)
D7	19558.4 (2.7)	4.9 (0)	0.05	0	-0.01 (0.05)	0.3 (0.0002)	95%	-0.01 (0.0096)	1.18 (0.0007)	0	-0.01 (0.0096)	0.3 (0.0002)	95%	-0.01 (0.0096)	1.18 (0.0007)
D8	19154.2 (2.6)	4.6 (0)	0.05	0	0 (0.047)	0.28 (0.0002)	95.3%	0 (0.0087)	1.09 (0.0006)	0	0 (0.0087)	0.28 (0.0002)	95.3%	0 (0.0087)	1.09 (0.0006)
D9	19558.4 (2.7)	4.9 (0)	1	2	1.99 (1)	0.3 (0.0002)	95%	-0.01 (0.0096)	1.18 (0.0007)	2	1.99 (0.0096)	0.3 (0.0002)	95%	-0.01 (0.0096)	1.18 (0.0007)
D10	19154.2 (2.6)	4.6 (0)	1	2	2 (1)	0.28 (0.0002)	95.3%	0 (0.0087)	1.09 (0.0006)	2	2 (0.0087)	0.28 (0.0002)	95.3%	0 (0.0087)	1.09 (0.0006)
D11	19558.4 (2.7)	4.9 (0)	1	2	1.99 (1)	0.3 (0.0002)	95%	-0.01 (0.0096)	1.18 (0.0007)	2	1.99 (0.0096)	0.3 (0.0002)	95%	-0.01 (0.0096)	1.18 (0.0007)
D12	19154.2 (2.6)	4.6 (0)	1	2	2 (1)	0.28 (0.0002)	95.3%	0 (0.0087)	1.09 (0.0006)	2	2 (0.0087)	0.28 (0.0002)	95.3%	0 (0.0087)	1.09 (0.0006)
D13	19563.8 (2.7)	4.9 (0)	1	2.9	2.12 (0.999)	0.3 (0.0002)	26.3%	-0.78 (0.0096)	1.18 (0.0007)	2.9	2.12 (0.0096)	0.3 (0.0002)	26.3%	-0.78 (0.0096)	1.18 (0.0007)
D14	19160.5 (2.6)	4.6 (0)	1	2.9	2.12 (1)	0.28 (0.0002)	19.6%	-0.78 (0.0087)	1.09 (0.0006)	2.9	2.12 (0.0087)	0.28 (0.0002)	19.6%	-0.78 (0.0087)	1.09 (0.0006)
D15	19563.6 (2.7)	4.9 (0)	0.07	0.9	0.12 (0.067)	0.3 (0.0002)	26.3%	-0.78 (0.0096)	1.18 (0.0007)	0.9	0.12 (0.0096)	0.3 (0.0002)	26.3%	-0.78 (0.0096)	1.18 (0.0007)
D16	19160.5 (2.6)	4.6 (0)	0.07	0.9	0.12 (0.07)	0.28 (0.0002)	19.6%	-0.78 (0.0087)	1.09 (0.0006)	0.9	0.12 (0.0087)	0.28 (0.0002)	19.6%	-0.78 (0.0087)	1.09 (0.0006)
D17	19573.1 (2.7)	4.9 (0)	1	3.5	2.21 (1)	0.3 (0.0002)	1.2%	-1.29 (0.0096)	1.18 (0.0007)	3.5	2.21 (0.0096)	0.3 (0.0002)	1.2%	-1.29 (0.0096)	1.18 (0.0007)
D18	19171.7 (2.6)	4.6 (0)	1	3.5	2.19 (1)	0.28 (0.0002)	0.3%	-1.31 (0.0087)	1.1 (0.0006)	3.5	2.19 (0.0087)	0.28 (0.0002)	0.3%	-1.31 (0.0087)	1.1 (0.0006)
D19	19573.1 (2.7)	4.9 (0)	0.11	1.5	0.21 (0.106)	0.3 (0.0002)	1.2%	-1.29 (0.0096)	1.18 (0.0007)	1.5	0.21 (0.0096)	0.3 (0.0002)	1.2%	-1.29 (0.0096)	1.18 (0.0007)
D20	19171.7 (2.6)	4.6 (0)	0.11	1.5	0.19 (0.109)	0.28 (0.0002)	0.3%	-1.31 (0.0087)	1.1 (0.0006)	1.5	0.19 (0.0087)	0.28 (0.0002)	0.3%	-1.31 (0.0087)	1.1 (0.0006)
D21	19616.6 (2.6)	5 (0)	1	-5	-2.4 (1)	0.31 (0.0002)	0%	2.6 (0.0095)	1.21 (0.0008)	-5	-2.4 (0.0095)	0.31 (0.0002)	0%	2.6 (0.0095)	1.21 (0.0008)
D22	19221.9 (2.6)	4.6 (0)	1	-5	-2.36 (1)	0.29 (0.0002)	0%	2.64 (0.0089)	1.12 (0.0006)	-5	-2.36 (0.0089)	0.29 (0.0002)	0%	2.64 (0.0089)	1.12 (0.0006)
D23	19616.6 (2.6)	5 (0)	0.26	-3	-0.4 (0.259)	0.31 (0.0002)	0%	2.6 (0.0095)	1.21 (0.0008)	-3	-0.4 (0.0095)	0.31 (0.0002)	0%	2.6 (0.0095)	1.21 (0.0008)
D24	19221.9 (2.6)	4.6 (0)	0.26	-3	-0.36 (0.256)	0.29 (0.0002)	0%	2.64 (0.0089)	1.12 (0.0006)	-3	-0.36 (0.0089)	0.29 (0.0002)	0%	2.64 (0.0089)	1.12 (0.0006)
D25	19558.4 (2.7)	4.9 (0)	1	2	1.99 (1)	0.3 (0.0002)	95%	-0.01 (0.0096)	1.18 (0.0007)	2	1.99 (0.0096)	0.3 (0.0002)	95%	-0.01 (0.0096)	1.18 (0.0007)
D26	19154.2 (2.6)	4.6 (0)	1	2	2 (1)	0.28 (0.0002)	95.3%	0 (0.0087)	1.09 (0.0006)	2	2 (0.0087)	0.28 (0.0002)	95.3%	0 (0.0087)	1.09 (0.0006)
D27	19564.1 (2.7)	4.9 (0)	1	2.966	2.26 (1)	0.3 (0.0002)	34.4%	-0.71 (0.0096)	1.18 (0.0007)	2.58	2.26 (0.0096)	0.3 (0.0002)	80.2%	-0.32 (0.0096)	1.18 (0.0007)
D28	19161.1 (2.6)	4.6 (0)	1	2.966	2.26 (1)	0.28 (0.0002)	27.2%	-0.71 (0.0087)	1.09 (0.0006)	2.58	2.26 (0.0087)	0.28 (0.0002)	78.3%	-0.32 (0.0087)	1.09 (0.0006)
D29	19564.1 (2.7)	4.9 (0)	0.14	0.966	0.26 (0.136)	0.3 (0.0002)	34.4%	-0.71 (0.0096)	1.18 (0.0007)	0.58	0.26 (0.0096)	0.3 (0.0002)	80.2%	-0.32 (0.0096)	1.18 (0.0007)
D30	19161.1 (2.6)	4.6 (0)	0.15	0.966	0.26 (0.152)	0.28 (0.0002)	27.2%	-0.71 (0.0087)	1.09 (0.0006)	0.58	0.26 (0.0087)	0.28 (0.0002)	78.3%	-0.32 (0.0087)	1.09 (0.0006)
D31	19558.4 (2.7)	4.9 (0)	1	2	1.99 (1)	0.3 (0.0002)	95%	-0.01 (0.0096)	1.18 (0.0007)	2	1.99 (0.0096)	0.3 (0.0002)	95%	-0.01 (0.0096)	1.18 (0.0007)
D32	19154.2 (2.6)	4.6 (0)	1	2	2 (1)	0.28 (0.0002)	95.3%	0 (0.0087)	1.09 (0.0006)	2	2 (0.0087)	0.28 (0.0002)	95.3%	0 (0.0087)	1.09 (0.0006)
D33	19575.9 (2.7)	4.9 (0)	1	2.5	2.74 (1)	0.3 (0.0002)	88.1%	0.24 (0.0096)	1.18 (0.0007)	2	2.74 (0.0096)	0.3 (0.0002)	32.6%	0.74 (0.0096)	1.18 (0.0007)
D34	19174.8 (2.6)	4.6 (0)	1	2.5	2.77 (1)	0.28 (0.0002)	84.2%	0.27 (0.0087)	1.09 (0.0006)	2	2.77 (0.0087)	0.28 (0.0002)	22.2%	0.77 (0.0087)	1.09 (0.0006)
D35	19575.9 (2.7)	4.9 (0)	0.67	0.5	0.74 (0.672)	0.3 (0.0002)	88.1%	0.24 (0.0096)	1.18 (0.0007)	0	0.74 (0.0096)	0.3 (0.0002)	32.6%	0.74 (0.0096)	1.18 (0.0007)
D36	19174.9 (2.6)	4.6 (0)	0.77	0.5	0.77 (0.772)	0.28 (0.0002)	84.1%	0.27 (0.0087)	1.09 (0.0006)	0	0.77 (0.0087)	0.28 (0.0002)	22.2%	0.77 (0.0087)	1.09 (0.0006)

S3 Table M: The mean treatment effect estimates, their standard errors, and goodness-of-fit statistics, for each of the 36 simulated scenarios assuming the mean structure from Model 4 ($y_{itk} = \beta_0 + \partial x_{tk} + \tau t + \omega x_{tk} t + v_{0ik} + \epsilon_{itk}$) with autoregressive (AR(1)) covariance structure between within subject measurement, together with the Monte Carlo standard error of each estimate in brackets. Each scenario was simulated 1000 times.

				Treatment Effect at 6 months						Time Averaged Treatment Effect					
Scenario	BIC	MSE	Proportion Significant Intervention Parameter	True Value	Estimate	Estimate SE	Coverage of 95% C.I.	Bias	Confidence Interval Width	True Value	Estimate	Estimate SE	Coverage of 95% C.I.	Bias	Confidence Interval Width
D1	19493.7 (2.7)	4.9 (0)	0.07	0	-0.01 (0.073)	0.31 (0.0002)	94.6%	-0.01 (0.0097)	1.21 (0.0008)	0	-0.01 (0.0096)	0.3 (0.0002)	94.8%	-0.01 (0.0096)	1.19 (0.0007)
D2	19089 (2.6)	4.6 (0)	0.07	0	0 (0.068)	0.29 (0.0002)	94.8%	0 (0.0089)	1.12 (0.0006)	0	0 (0.0088)	0.28 (0.0002)	95%	0 (0.0088)	1.09 (0.0006)
D3	19487.6 (2.7)	4.9 (0)	0.07	0	-0.01 (0.073)	0.31 (0.0002)	95.1%	-0.01 (0.0097)	1.2 (0.0007)	0	-0.01 (0.0096)	0.3 (0.0002)	95%	-0.01 (0.0096)	1.18 (0.0007)
D4	19083.7 (2.6)	4.6 (0)	0.07	0	0 (0.067)	0.28 (0.0002)	95%	0 (0.0089)	1.11 (0.0006)	0	0 (0.0087)	0.28 (0.0002)	95.3%	0 (0.0087)	1.09 (0.0006)
D5	19515.2 (2.7)	5 (0)	1	0	0.35 (1)	0.31 (0.0002)	78.9%	0.35 (0.0097)	1.22 (0.0007)	0	-0.01 (0.0096)	0.3 (0.0002)	95.5%	-0.01 (0.0096)	1.19 (0.0007)
D6	19116.7 (2.6)	4.6 (0)	1	0	0.36 (0.999)	0.29 (0.0002)	76.1%	0.36 (0.0089)	1.12 (0.0006)	0	0 (0.0087)	0.28 (0.0002)	95.4%	0 (0.0087)	1.1 (0.0006)
D7	19609 (2.6)	5.1 (0)	0.06	0	-0.01 (0.061)	0.32 (0.0002)	95.8%	-0.01 (0.0097)	1.25 (0.0007)	0	-0.01 (0.0096)	0.31 (0.0002)	95.8%	-0.01 (0.0096)	1.23 (0.0007)
D8	19227.8 (2.5)	4.7 (0)	0.05	0	0 (0.052)	0.3 (0.0002)	95.8%	0 (0.0089)	1.16 (0.0006)	0	0 (0.0087)	0.29 (0.0002)	96%	0 (0.0087)	1.14 (0.0006)
D9	19487.6 (2.7)	4.9 (0)	0.98	2	1.99 (0.98)	0.31 (0.0002)	95%	-0.01 (0.0097)	1.2 (0.0007)	2	1.99 (0.0096)	0.3 (0.0002)	94.9%	-0.01 (0.0096)	1.18 (0.0007)
D10	19083.7 (2.6)	4.6 (0)	0.99	2	2 (0.994)	0.28 (0.0002)	95%	0 (0.0089)	1.11 (0.0006)	2	2 (0.0087)	0.28 (0.0002)	95.3%	0 (0.0087)	1.09 (0.0006)
D11	19487.6 (2.7)	4.9 (0)	0.98	2	1.99 (0.981)	0.31 (0.0002)	95.1%	-0.01 (0.0097)	1.2 (0.0007)	2	1.99 (0.0096)	0.3 (0.0002)	95%	-0.01 (0.0096)	1.18 (0.0007)
D12	19083.7 (2.6)	4.6 (0)	0.99	2	2 (0.993)	0.28 (0.0002)	94.9%	0 (0.0089)	1.11 (0.0006)	2	2 (0.0087)	0.28 (0.0002)	95.2%	0 (0.0087)	1.09 (0.0006)
D13	19491.6 (2.7)	4.9 (0)	0.96	2.9	2.03 (0.963)	0.31 (0.0002)	19.7%	-0.87 (0.0097)	1.21 (0.0007)	2.9	2.12 (0.0096)	0.3 (0.0002)	26.3%	-0.78 (0.0096)	1.18 (0.0007)
D14	19088.4 (2.6)	4.6 (0)	0.99	2.9	2.02 (0.986)	0.28 (0.0002)	13.3%	-0.88 (0.0089)	1.11 (0.0006)	2.9	2.12 (0.0087)	0.28 (0.0002)	19.6%	-0.78 (0.0087)	1.09 (0.0006)
D15	19491.5 (2.7)	4.9 (0)	0.33	0.9	0.03 (0.326)	0.31 (0.0002)	19.8%	-0.87 (0.0097)	1.21 (0.0007)	0.9	0.12 (0.0096)	0.3 (0.0002)	26.4%	-0.78 (0.0096)	1.18 (0.0007)
D16	19088.4 (2.6)	4.6 (0)	0.36	0.9	0.02 (0.358)	0.28 (0.0002)	13.3%	-0.88 (0.0089)	1.11 (0.0006)	0.9	0.12 (0.0087)	0.28 (0.0002)	19.6%	-0.78 (0.0087)	1.09 (0.0006)
D17	19498.6 (2.7)	4.9 (0)	0.98	3.5	2.05 (0.98)	0.31 (0.0002)	0.3%	-1.45 (0.0097)	1.21 (0.0007)	3.5	2.21 (0.0096)	0.3 (0.0002)	1.1%	-1.29 (0.0096)	1.18 (0.0007)
D18	19096.8 (2.6)	4.6 (0)	1	3.5	2.04 (0.995)	0.29 (0.0002)	0.1%	-1.46 (0.0089)	1.12 (0.0006)	3.5	2.19 (0.0087)	0.28 (0.0002)	0.3%	-1.31 (0.0087)	1.09 (0.0006)
D19	19498.6 (2.7)	4.9 (0)	0.69	1.5	0.05 (0.687)	0.31 (0.0002)	0.3%	-1.45 (0.0097)	1.21 (0.0007)	1.5	0.21 (0.0096)	0.3 (0.0002)	1.1%	-1.29 (0.0096)	1.18 (0.0007)
D20	19096.6 (2.6)	4.6 (0)	0.78	1.5	0.04 (0.779)	0.29 (0.0002)	0.1%	-1.46 (0.0089)	1.12 (0.0006)	1.5	0.19 (0.0087)	0.28 (0.0002)	0.3%	-1.31 (0.0087)	1.09 (0.0006)
D21	19531.4 (2.7)	5 (0)	1	-5	-2.09 (1)	0.31 (0.0002)	0%	2.91 (0.0097)	1.23 (0.0008)	-5	-2.39 (0.0095)	0.31 (0.0002)	0%	2.61 (0.0095)	1.2 (0.0007)
D22	19133.6 (2.6)	4.6 (0)	1	-5	-2.04 (1)	0.29 (0.0002)	0%	2.96 (0.0091)	1.13 (0.0006)	-5	-2.35 (0.0089)	0.28 (0.0002)	0%	2.65 (0.0089)	1.11 (0.0006)
D23	19531.4 (2.7)	5 (0)	1	-3	-0.09 (0.995)	0.31 (0.0002)	0%	2.91 (0.0097)	1.23 (0.0008)	-3	-0.39 (0.0095)	0.31 (0.0002)	0%	2.61 (0.0095)	1.2 (0.0007)
D24	19133.6 (2.6)	4.6 (0)	1	-3	-0.04 (1)	0.29 (0.0002)	0%	2.96 (0.0091)	1.13 (0.0006)	-3	-0.35 (0.0089)	0.28 (0.0002)	0%	2.65 (0.0089)	1.11 (0.0006)
D25	19515.2 (2.7)	5 (0)	1	2	2.35 (1)	0.31 (0.0002)	78.9%	0.35 (0.0097)	1.22 (0.0007)	2	1.99 (0.0096)	0.3 (0.0002)	95.5%	-0.01 (0.0096)	1.19 (0.0007)
D26	19116.4 (2.6)	4.6 (0)	1	2	2.36 (0.999)	0.29 (0.0002)	76%	0.36 (0.0089)	1.12 (0.0006)	2	2 (0.0087)	0.28 (0.0002)	95.4%	0 (0.0087)	1.1 (0.0006)
D27	19525.8 (2.7)	5 (0)	1	2.966	2.57 (1)	0.31 (0.0002)	75%	-0.39 (0.0097)	1.22 (0.0007)	2.58	2.26 (0.0096)	0.3 (0.0002)	80.8%	-0.32 (0.0096)	1.19 (0.0007)
D28	19129.3 (2.6)	4.6 (0)	1	2.966	2.58 (1)	0.29 (0.0002)	72.5%	-0.39 (0.0089)	1.13 (0.0006)	2.58	2.26 (0.0087)	0.28 (0.0002)	78.6%	-0.32 (0.0087)	1.11 (0.0006)
D29	19525.8 (2.7)	5 (0)	1	0.966	0.57 (0.998)	0.31 (0.0002)	75%	-0.39 (0.0097)	1.22 (0.0007)	0.58	0.26 (0.0096)	0.3 (0.0002)	80.8%	-0.32 (0.0096)	1.19 (0.0007)
D30	19129.3 (2.6)	4.6 (0)	1	0.966	0.58 (1)	0.29 (0.0002)	72.5%	-0.39 (0.0089)	1.13 (0.0006)	0.58	0.26 (0.0087)	0.28 (0.0002)	78.6%	-0.32 (0.0087)	1.11 (0.0006)
D31	19609 (2.6)	5.1 (0)	0.97	2	1.99 (0.974)	0.32 (0.0002)	95.7%	-0.01 (0.0098)	1.25 (0.0007)	2	1.99 (0.0096)	0.31 (0.0002)	95.7%	-0.01 (0.0096)	1.23 (0.0007)
D32	19227.8 (2.5)	4.7 (0)	0.99	2	2 (0.99)	0.3 (0.0002)	95.8%	0 (0.0089)	1.16 (0.0006)	2	2 (0.0087)	0.29 (0.0002)	96%	0 (0.0087)	1.14 (0.0006)
D33	19620.2 (2.6)	5.1 (0)	1	2.5	2.8 (1)	0.32 (0.0002)	84.7%	0.3 (0.0098)	1.26 (0.0007)	2	2.71 (0.0096)	0.31 (0.0002)	38%	0.71 (0.0096)	1.23 (0.0007)
D34	19240.7 (2.6)	4.7 (0)	1	2.5	2.83 (0.999)	0.3 (0.0002)	81.5%	0.33 (0.0089)	1.17 (0.0006)	2	2.74 (0.0087)	0.29 (0.0002)	27.4%	0.74 (0.0087)	1.14 (0.0006)
D35	19620.2 (2.6)	5.1 (0)	0.66	0.5	0.8 (0.655)	0.32 (0.0002)	84.7%	0.3 (0.0098)	1.26 (0.0007)	0	0.71 (0.0096)	0.31 (0.0002)	38%	0.71 (0.0096)	1.23 (0.0007)
D36	19240.8 (2.6)	4.7 (0)	0.76	0.5	0.83 (0.756)	0.3 (0.0002)	81.5%	0.33 (0.0089)	1.17 (0.0006)	0	0.74 (0.0087)	0.29 (0.0002)	27.4%	0.74 (0.0087)	1.14 (0.0006)

S3 Table N: The mean treatment effect estimates, their standard errors, and goodness-of-fit statistics, for each of the 36 simulated scenarios assuming the mean structure from Model 5 ($y_{itk} = \beta_0 + \partial x_{tk} + \kappa_t + \varphi_t + v_{0ik} + \epsilon_{itk}$) with autoregressive (AR(1)) covariance structure between within subject measurement, together with the Monte Carlo standard error of each estimate in brackets. Each scenario was simulated 1000 times.

				Treatment Effect at 6 months						Time Averaged Treatment Effect					
Scenario	BIC	MSE	Proportion Significant Intervention Parameter	True Value	Estimate	Estimate SE	Coverage of 95% C.I.	Bias	Confidence Interval Width	True Value	Estimate	Estimate SE	Coverage of 95% C.I.	Bias	Confidence Interval Width
D1	19635.8 (2.7)	4.9 (0)	0.06	0	0.03 (0.058)	0.68 (0.0003)	97.6%	0.03 (0.0201)	2.67 (0.0012)	0	-0.01 (0.0101)	0.32 (0.0002)	95.6%	-0.01 (0.0101)	1.25 (0.0007)
D2	19229.5 (2.6)	4.6 (0)	0.11	0	0.04 (0.108)	0.63 (0.0003)	96.8%	0.04 (0.0197)	2.47 (0.0011)	0	-0.01 (0.0093)	0.29 (0.0002)	95.2%	-0.01 (0.0093)	1.15 (0.0006)
D3	19629.7 (2.7)	4.9 (0)	0.06	0	0.03 (0.059)	0.68 (0.0003)	97.9%	0.03 (0.0201)	2.66 (0.0012)	0	-0.01 (0.0101)	0.32 (0.0002)	95.2%	-0.01 (0.0101)	1.24 (0.0007)
D4	19224.2 (2.6)	4.6 (0)	0.1	0	0.04 (0.104)	0.63 (0.0003)	96.9%	0.04 (0.0197)	2.47 (0.0011)	0	-0.01 (0.0092)	0.29 (0.0002)	95.1%	-0.01 (0.0092)	1.14 (0.0006)
D5	19629.7 (2.7)	4.9 (0)	0.06	0	0.03 (0.059)	0.68 (0.0003)	97.9%	0.03 (0.0201)	2.66 (0.0012)	0	-0.01 (0.0101)	0.32 (0.0002)	95.2%	-0.01 (0.0101)	1.24 (0.0007)
D6	19224.4 (2.6)	4.6 (0)	0.1	0	0.04 (0.104)	0.63 (0.0003)	96.8%	0.04 (0.0197)	2.47 (0.0011)	0	0 (0.0092)	0.29 (0.0002)	95%	0 (0.0092)	1.14 (0.0006)
D7	19629.7 (2.7)	4.9 (0)	0.06	0	0.03 (0.059)	0.68 (0.0003)	97.9%	0.03 (0.0201)	2.66 (0.0012)	0	-0.01 (0.0101)	0.32 (0.0002)	95.2%	-0.01 (0.0101)	1.24 (0.0007)
D8	19224.2 (2.6)	4.6 (0)	0.1	0	0.04 (0.104)	0.63 (0.0003)	96.9%	0.04 (0.0197)	2.47 (0.0011)	0	-0.01 (0.0092)	0.29 (0.0002)	95.1%	-0.01 (0.0092)	1.14 (0.0006)
D9	19629.7 (2.7)	4.9 (0)	0.39	2	2.03 (0.389)	0.68 (0.0003)	97.9%	0.03 (0.0201)	2.66 (0.0012)	2	1.99 (0.0101)	0.32 (0.0002)	95.2%	-0.01 (0.0101)	1.24 (0.0007)
D10	19224.2 (2.6)	4.6 (0)	0.46	2	2.04 (0.458)	0.63 (0.0003)	96.9%	0.04 (0.0197)	2.47 (0.0011)	2	1.99 (0.0092)	0.29 (0.0002)	95.1%	-0.01 (0.0092)	1.14 (0.0006)
D11	19629.7 (2.7)	4.9 (0)	0.39	2	2.03 (0.389)	0.68 (0.0003)	97.8%	0.03 (0.0201)	2.66 (0.0012)	2	1.99 (0.0101)	0.32 (0.0002)	95.1%	-0.01 (0.0101)	1.24 (0.0007)
D12	19224.2 (2.6)	4.6 (0)	0.46	2	2.04 (0.458)	0.63 (0.0003)	96.9%	0.04 (0.0197)	2.47 (0.0011)	2	1.99 (0.0092)	0.29 (0.0002)	95.1%	-0.01 (0.0092)	1.14 (0.0006)
D13	19633.6 (2.7)	4.9 (0)	0.3	2.9	2.05 (0.295)	0.68 (0.0003)	75.2%	-0.85 (0.0201)	2.67 (0.0012)	2.9	2.17 (0.0101)	0.32 (0.0002)	37%	-0.73 (0.0101)	1.24 (0.0007)
D14	19228.8 (2.6)	4.6 (0)	0.37	2.9	2.04 (0.373)	0.63 (0.0003)	71.2%	-0.86 (0.0197)	2.47 (0.0011)	2.9	2.17 (0.0092)	0.29 (0.0002)	29.7%	-0.73 (0.0092)	1.15 (0.0006)
D15	19633.6 (2.7)	4.9 (0)	0.1	0.9	0.05 (0.096)	0.68 (0.0003)	75.2%	-0.85 (0.0201)	2.67 (0.0012)	0.9	0.17 (0.0101)	0.32 (0.0002)	37%	-0.73 (0.0101)	1.24 (0.0007)
D16	19228.8 (2.6)	4.6 (0)	0.17	0.9	0.04 (0.167)	0.63 (0.0003)	71.2%	-0.86 (0.0197)	2.47 (0.0011)	0.9	0.17 (0.0092)	0.29 (0.0002)	29.7%	-0.73 (0.0092)	1.15 (0.0006)
D17	19640.6 (2.7)	4.9 (0)	0.32	3.5	2.06 (0.322)	0.68 (0.0003)	43.5%	-1.44 (0.0201)	2.67 (0.0012)	3.5	2.29 (0.0101)	0.32 (0.0002)	3.3%	-1.21 (0.0101)	1.24 (0.0007)
D18	19236.9 (2.6)	4.6 (0)	0.44	3.5	2.04 (0.435)	0.63 (0.0003)	37.6%	-1.46 (0.0197)	2.47 (0.0011)	3.5	2.28 (0.0093)	0.29 (0.0002)	1.5%	-1.22 (0.0093)	1.15 (0.0006)
D19	19640.6 (2.7)	4.9 (0)	0.2	1.5	0.06 (0.204)	0.68 (0.0003)	43.5%	-1.44 (0.0201)	2.67 (0.0012)	1.5	0.29 (0.0101)	0.32 (0.0002)	3.3%	-1.21 (0.0101)	1.24 (0.0007)
D20	19236.9 (2.6)	4.6 (0)	0.32	1.5	0.04 (0.324)	0.63 (0.0003)	37.6%	-1.46 (0.0197)	2.47 (0.0011)	1.5	0.28 (0.0093)	0.29 (0.0002)	1.5%	-1.22 (0.0093)	1.15 (0.0006)
D21	19672.8 (2.7)	5 (0)	0.77	-5	-1.98 (0.768)	0.69 (0.0003)	0.3%	3.02 (0.02)	2.69 (0.0012)	-5	-2.58 (0.0101)	0.32 (0.0002)	0%	2.42 (0.0101)	1.26 (0.0007)
D22	19273.1 (2.6)	4.6 (0)	0.89	-5	-1.93 (0.89)	0.64 (0.0003)	0.2%	3.07 (0.0197)	2.49 (0.0011)	-5	-2.55 (0.0094)	0.3 (0.0002)	0%	2.45 (0.0094)	1.16 (0.0006)
D23	19672.8 (2.7)	5 (0)	0.77	-3	0.02 (0.767)	0.69 (0.0003)	0.3%	3.02 (0.02)	2.69 (0.0012)	-3	-0.58 (0.0101)	0.32 (0.0002)	0%	2.42 (0.0101)	1.26 (0.0007)
D24	19273.1 (2.6)	4.6 (0)	0.89	-3	0.07 (0.888)	0.64 (0.0003)	0.2%	3.07 (0.0197)	2.49 (0.0011)	-3	-0.55 (0.0094)	0.3 (0.0002)	0%	2.45 (0.0094)	1.16 (0.0006)
D25	19629.6 (2.7)	4.9 (0)	0.39	2	2.03 (0.389)	0.68 (0.0003)	97.8%	0.03 (0.0201)	2.66 (0.0012)	2	1.99 (0.0101)	0.32 (0.0002)	95.1%	-0.01 (0.0101)	1.24 (0.0007)
D26	19224.3 (2.6)	4.6 (0)	0.46	2	2.04 (0.458)	0.63 (0.0003)	96.8%	0.04 (0.0197)	2.47 (0.0011)	2	1.99 (0.0092)	0.29 (0.0002)	95%	-0.01 (0.0092)	1.14 (0.0006)
D27	19634.7 (2.7)	4.9 (0)	0.31	2.966	2.25 (0.314)	0.68 (0.0003)	80.6%	-0.71 (0.0201)	2.67 (0.0012)	2.58	2.28 (0.0101)	0.32 (0.0002)	84.5%	-0.3 (0.0101)	1.24 (0.0007)
D28	19230.1 (2.6)	4.6 (0)	0.41	2.966	2.26 (0.407)	0.63 (0.0003)	77.5%	-0.71 (0.0197)	2.47 (0.0011)	2.58	2.28 (0.0092)	0.29 (0.0002)	83.2%	-0.3 (0.0092)	1.15 (0.0006)
D29	19634.7 (2.7)	4.9 (0)	0.07	0.966	0.25 (0.069)	0.68 (0.0003)	80.6%	-0.71 (0.0201)	2.67 (0.0012)	0.58	0.28 (0.0101)	0.32 (0.0002)	84.5%	-0.3 (0.0101)	1.24 (0.0007)
D30	19230.1 (2.6)	4.6 (0)	0.13	0.966	0.26 (0.128)	0.63 (0.0003)	77.5%	-0.71 (0.0197)	2.47 (0.0011)	0.58	0.28 (0.0092)	0.29 (0.0002)	83.2%	-0.3 (0.0092)	1.15 (0.0006)
D31	19629.7 (2.7)	4.9 (0)	0.39	2	2.03 (0.389)	0.68 (0.0003)	97.9%	0.03 (0.0201)	2.66 (0.0012)	2	1.99 (0.0101)	0.32 (0.0002)	95.2%	-0.01 (0.0101)	1.24 (0.0007)
D32	19224.2 (2.6)	4.6 (0)	0.46	2	2.04 (0.458)	0.63 (0.0003)	96.9%	0.04 (0.0197)	2.47 (0.0011)	2	1.99 (0.0092)	0.29 (0.0002)	95.1%	-0.01 (0.0092)	1.14 (0.0006)
D33	19646 (2.7)	4.9 (0)	0.57	2.5	3.06 (0.571)	0.68 (0.0003)	88.6%	0.56 (0.0201)	2.67 (0.0012)	2	2.68 (0.0101)	0.32 (0.0002)	43.3%	0.68 (0.0101)	1.24 (0.0007)
D34	19243.2 (2.6)	4.6 (0)	0.67	2.5	3.09 (0.665)	0.63 (0.0003)	84.2%	0.59 (0.0197)	2.48 (0.0011)	2	2.7 (0.0093)	0.29 (0.0002)	34.6%	0.7 (0.0093)	1.15 (0.0006)
D35	19645.9 (2.7)	4.9 (0)	0.11	0.5	1.06 (0.109)	0.68 (0.0003)	88.6%	0.56 (0.0201)	2.67 (0.0012)	0	0.68 (0.0101)	0.32 (0.0002)	43.2%	0.68 (0.0101)	1.24 (0.0007)
D36	19243.2 (2.6)	4.6 (0)	0.18	0.5	1.09 (0.184)	0.63 (0.0003)	84.2%	0.59 (0.0197)	2.48 (0.0011)	0	0.7 (0.0093)	0.29 (0.0002)	34.6%	0.7 (0.0093)	1.15 (0.0006)

S3 Table O: The mean treatment effect estimates, their standard errors, and goodness-of-fit statistics, for each of the 36 simulated scenarios assuming the mean structure from Model 6 ($y_{itk} = \beta_0 + \partial x_{tk} + \tau t + \psi d_{tk} + v_{0ik} + \epsilon_{itk}$) with autoregressive (AR(1)) covariance structure between within subject measurement, together with the Monte Carlo standard error of each estimate in brackets. Each scenario was simulated 1000 times.

				Treatment Effect at 6 months						Time Averaged Treatment Effect					
Scenario	BIC	MSE	Proportion Significant Intervention Parameter	True Value	Estimate	Estimate SE	Coverage of 95% C.I.	Bias	Confidence Interval Width	True Value	Estimate	Estimate SE	Coverage of 95% C.I.	Bias	Confidence Interval Width
D1	19493.5 (2.7)	4.9 (0)	0.12	0	-0.01 (0.118)	0.44 (0.0004)	91.1%	-0.01 (0.0156)	1.72 (0.0015)	0	-0.01 (0.0156)	0.44 (0.0004)	91.1%	-0.01 (0.0156)	1.72 (0.0015)
D2	19089 (2.6)	4.6 (0)	0.09	0	0 (0.094)	0.41 (0.0003)	96.8%	0 (0.0121)	1.6 (0.0012)	0	0 (0.0121)	0.41 (0.0003)	96.8%	0 (0.0121)	1.6 (0.0012)
D3	19487.4 (2.7)	4.9 (0)	0.12	0	-0.01 (0.116)	0.43 (0.0003)	91.2%	-0.01 (0.0154)	1.69 (0.0012)	0	-0.01 (0.0154)	0.43 (0.0003)	91.2%	-0.01 (0.0154)	1.69 (0.0012)
D4	19083.7 (2.6)	4.6 (0)	0.1	0	0 (0.095)	0.4 (0.0003)	96.4%	0 (0.0119)	1.57 (0.001)	0	0 (0.0119)	0.4 (0.0003)	96.4%	0 (0.0119)	1.57 (0.001)
D5	19539.1 (2.7)	5 (0)	0.77	0	-0.86 (0.772)	0.44 (0.0003)	51.3%	-0.86 (0.0154)	1.72 (0.0013)	0	-0.86 (0.0154)	0.44 (0.0003)	51.3%	-0.86 (0.0154)	1.72 (0.0013)
D6	19145.4 (2.6)	4.6 (0)	0.85	0	-0.88 (0.846)	0.41 (0.0003)	43%	-0.88 (0.0119)	1.61 (0.0011)	0	-0.88 (0.0119)	0.41 (0.0003)	43%	-0.88 (0.0119)	1.61 (0.0011)
D7	19608.8 (2.6)	5.1 (0)	0.11	0	-0.01 (0.108)	0.45 (0.0003)	92.6%	-0.01 (0.0155)	1.76 (0.0013)	0	-0.01 (0.0155)	0.45 (0.0003)	92.6%	-0.01 (0.0155)	1.76 (0.0013)
D8	19227.8 (2.5)	4.7 (0)	0.07	0	0 (0.073)	0.42 (0.0003)	97.5%	0 (0.0118)	1.65 (0.0011)	0	0 (0.0118)	0.42 (0.0003)	97.5%	0 (0.0118)	1.65 (0.0011)
D9	19487.4 (2.7)	4.9 (0)	1	2	1.99 (1)	0.43 (0.0003)	91.2%	-0.01 (0.0154)	1.69 (0.0012)	2	1.99 (0.0154)	0.43 (0.0003)	91.2%	-0.01 (0.0154)	1.69 (0.0012)
D10	19083.7 (2.6)	4.6 (0)	1	2	2 (1)	0.4 (0.0003)	96.4%	0 (0.0119)	1.57 (0.001)	2	2 (0.0119)	0.4 (0.0003)	96.4%	0 (0.0119)	1.57 (0.001)
D11	19487.3 (2.7)	4.9 (0)	1	2	1.99 (0.999)	0.43 (0.0003)	91.1%	-0.01 (0.0154)	1.69 (0.0012)	2	1.99 (0.0154)	0.43 (0.0003)	91.1%	-0.01 (0.0154)	1.69 (0.0012)
D12	19083.7 (2.6)	4.6 (0)	1	2	2 (1)	0.4 (0.0003)	96.4%	0 (0.0119)	1.57 (0.001)	2	2 (0.0119)	0.4 (0.0003)	96.4%	0 (0.0119)	1.57 (0.001)
D13	19487.4 (2.7)	4.9 (0)	1	2.9	2.89 (1)	0.43 (0.0003)	91.2%	-0.01 (0.0154)	1.69 (0.0012)	2.9	2.89 (0.0154)	0.43 (0.0003)	91.2%	-0.01 (0.0154)	1.69 (0.0012)
D14	19083.5 (2.6)	4.6 (0)	1	2.9	2.9 (0.999)	0.4 (0.0003)	96.3%	0 (0.0119)	1.57 (0.001)	2.9	2.9 (0.0119)	0.4 (0.0003)	96.3%	0 (0.0119)	1.57 (0.001)
D15	19487.4 (2.7)	4.9 (0)	0.7	0.9	0.89 (0.696)	0.43 (0.0003)	91.2%	-0.01 (0.0154)	1.69 (0.0012)	0.9	0.89 (0.0154)	0.43 (0.0003)	91.2%	-0.01 (0.0154)	1.69 (0.0012)
D16	19083.7 (2.6)	4.6 (0)	0.79	0.9	0.9 (0.786)	0.4 (0.0003)	96.4%	0 (0.0119)	1.57 (0.001)	0.9	0.9 (0.0119)	0.4 (0.0003)	96.4%	0 (0.0119)	1.57 (0.001)
D17	19487.4 (2.7)	4.9 (0)	1	3.5	3.49 (1)	0.43 (0.0003)	91.2%	-0.01 (0.0154)	1.69 (0.0012)	3.5	3.49 (0.0154)	0.43 (0.0003)	91.2%	-0.01 (0.0154)	1.69 (0.0012)
D18	19083.8 (2.6)	4.6 (0)	1	3.5	3.5 (0.999)	0.4 (0.0003)	96.3%	0 (0.0119)	1.57 (0.001)	3.5	3.5 (0.0119)	0.4 (0.0003)	96.3%	0 (0.0119)	1.57 (0.001)
D19	19487.4 (2.7)	4.9 (0)	0.97	1.5	1.49 (0.973)	0.43 (0.0003)	91.2%	-0.01 (0.0154)	1.69 (0.0012)	1.5	1.49 (0.0154)	0.43 (0.0003)	91.2%	-0.01 (0.0154)	1.69 (0.0012)
D20	19083.7 (2.6)	4.6 (0)	1	1.5	1.5 (0.999)	0.4 (0.0003)	96.4%	0 (0.0119)	1.57 (0.001)	1.5	1.5 (0.0119)	0.4 (0.0003)	96.4%	0 (0.0119)	1.57 (0.001)
D21	19487.4 (2.7)	4.9 (0)	1	-5	-5.01 (1)	0.43 (0.0003)	91.2%	-0.01 (0.0154)	1.69 (0.0012)	-5	-5.01 (0.0154)	0.43 (0.0003)	91.2%	-0.01 (0.0154)	1.69 (0.0012)
D22	19083.7 (2.6)	4.6 (0)	1	-5	-5 (1)	0.4 (0.0003)	96.4%	0 (0.0119)	1.57 (0.001)	-5	-5 (0.0119)	0.4 (0.0003)	96.4%	0 (0.0119)	1.57 (0.001)
D23	19487.4 (2.7)	4.9 (0)	1	-3	-3.01 (1)	0.43 (0.0003)	91.2%	-0.01 (0.0154)	1.69 (0.0012)	-3	-3.01 (0.0154)	0.43 (0.0003)	91.2%	-0.01 (0.0154)	1.69 (0.0012)
D24	19083.7 (2.6)	4.6 (0)	1	-3	-3 (1)	0.4 (0.0003)	96.4%	0 (0.0119)	1.57 (0.001)	-3	-3 (0.0119)	0.4 (0.0003)	96.4%	0 (0.0119)	1.57 (0.001)
D25	19539.1 (2.7)	5 (0)	1	2	1.14 (1)	0.44 (0.0003)	51.3%	-0.86 (0.0154)	1.72 (0.0013)	2	1.14 (0.0154)	0.44 (0.0003)	51.3%	-0.86 (0.0154)	1.72 (0.0013)
D26	19145.6 (2.6)	4.6 (0)	1	2	1.13 (0.999)	0.41 (0.0003)	43%	-0.87 (0.0119)	1.61 (0.0011)	2	1.13 (0.0119)	0.41 (0.0003)	43%	-0.87 (0.0119)	1.61 (0.0011)
D27	19547.9 (2.7)	5 (0)	1	2.966	1.86 (0.999)	0.44 (0.0003)	31.3%	-1.1 (0.0155)	1.73 (0.0013)	2.58	1.86 (0.0155)	0.44 (0.0003)	61.8%	-0.72 (0.0155)	1.73 (0.0013)
D28	19156 (2.6)	4.6 (0)	1	2.966	1.85 (1)	0.41 (0.0003)	21.5%	-1.12 (0.0119)	1.62 (0.0011)	2.58	1.85 (0.0119)	0.41 (0.0003)	57.4%	-0.73 (0.0119)	1.62 (0.0011)
D29	19547.9 (2.7)	5 (0)	0.38	0.966	-0.14 (0.381)	0.44 (0.0003)	31.3%	-1.1 (0.0154)	1.73 (0.0013)	0.58	-0.14 (0.0154)	0.44 (0.0003)	61.8%	-0.72 (0.0154)	1.73 (0.0013)
D30	19156 (2.6)	4.6 (0)	0.39	0.966	-0.15 (0.388)	0.41 (0.0003)	21.5%	-1.12 (0.0119)	1.62 (0.0011)	0.58	-0.15 (0.0119)	0.41 (0.0003)	57.4%	-0.73 (0.0119)	1.62 (0.0011)
D31	19608.8 (2.6)	5.1 (0)	1	2	1.99 (0.999)	0.45 (0.0003)	92.5%	-0.01 (0.0155)	1.76 (0.0013)	2	1.99 (0.0155)	0.45 (0.0003)	92.5%	-0.01 (0.0155)	1.76 (0.0013)
D32	19227.8 (2.5)	4.7 (0)	1	2	2 (1)	0.42 (0.0003)	97.6%	0 (0.0118)	1.65 (0.0011)	2	2 (0.0118)	0.42 (0.0003)	97.6%	0 (0.0118)	1.65 (0.0011)
D33	19618.3 (2.6)	5.1 (0)	1	2.5	2.13 (0.999)	0.45 (0.0003)	84.4%	-0.37 (0.0155)	1.76 (0.0013)	2	2.13 (0.0155)	0.45 (0.0003)	92.1%	0.13 (0.0155)	1.76 (0.0013)
D34	19239 (2.6)	4.7 (0)	1	2.5	2.16 (1)	0.42 (0.0003)	89.4%	-0.34 (0.0118)	1.65 (0.0011)	2	2.16 (0.0118)	0.42 (0.0003)	96.3%	0.16 (0.0118)	1.65 (0.0011)
D35	19618.3 (2.6)	5.1 (0)	0.85	0.5	0.13 (0.848)	0.45 (0.0003)	84.5%	-0.37 (0.0155)	1.76 (0.0013)	0	0.13 (0.0155)	0.45 (0.0003)	92.2%	0.13 (0.0155)	1.76 (0.0013)
D36	19239 (2.6)	4.7 (0)	0.87	0.5	0.16 (0.871)	0.42 (0.0003)	89.3%	-0.34 (0.0118)	1.65 (0.0011)	0	0.16 (0.0118)	0.42 (0.0003)	96.2%	0.16 (0.0118)	1.65 (0.0011)

S3 Table P: The mean treatment effect estimates, their standard errors, and goodness-of-fit statistics, for each of the 36 simulated scenarios assuming the mean structure from Model 7 ($y_{itk} = \beta_0 + \tau t + \psi d_{tk} + v_{0ik} + \epsilon_{itk}$) with autoregressive (AR(1)) covariance structure between within subject measurement, together with the Monte Carlo standard error of each estimate in brackets. Each scenario was simulated 1000 times.

				Treatment Effect at 6 months						Time Averaged Treatment Effect					
Scenario	BIC	MSE	Proportion Significant Intervention Parameter	True Value	Estimate	Estimate SE	Coverage of 95% C.I.	Bias	Confidence Interval Width	True Value	Estimate	Estimate SE	Coverage of 95% C.I.	Bias	Confidence Interval Width
D1	19486.4 (2.7)	4.9 (0)	0.09	0	-0.01 (0.091)	0.36 (0.0003)	90.9%	-0.01 (0.0126)	1.42 (0.0012)	0	-0.01 (0.0126)	0.36 (0.0003)	90.9%	-0.01 (0.0126)	1.42 (0.0012)
D2	19082 (2.6)	4.6 (0)	0.04	0	0 (0.041)	0.33 (0.0002)	95.9%	0 (0.0101)	1.31 (0.0009)	0	0 (0.0101)	0.33 (0.0002)	95.9%	0 (0.0101)	1.31 (0.0009)
D3	19480.3 (2.7)	4.9 (0)	0.08	0	-0.01 (0.081)	0.36 (0.0003)	91.9%	-0.01 (0.0124)	1.39 (0.001)	0	-0.01 (0.0124)	0.36 (0.0003)	91.9%	-0.01 (0.0124)	1.39 (0.001)
D4	19076.6 (2.6)	4.6 (0)	0.04	0	0 (0.039)	0.33 (0.0002)	96.1%	0 (0.0099)	1.29 (0.0009)	0	0 (0.0099)	0.33 (0.0002)	96.1%	0 (0.0099)	1.29 (0.0009)
D5	19532.1 (2.7)	5 (0)	0.75	0	-0.97 (0.748)	0.36 (0.0003)	25.1%	-0.97 (0.0125)	1.43 (0.0011)	0	-0.97 (0.0125)	0.36 (0.0003)	25.1%	-0.97 (0.0125)	1.43 (0.0011)
D6	19138.5 (2.6)	4.6 (0)	0.84	0	-0.98 (0.841)	0.34 (0.0002)	15.7%	-0.98 (0.0099)	1.33 (0.0009)	0	-0.98 (0.0099)	0.34 (0.0002)	15.7%	-0.98 (0.0099)	1.33 (0.0009)
D7	19601.7 (2.6)	5.1 (0)	0.08	0	-0.01 (0.075)	0.37 (0.0003)	92.5%	-0.01 (0.0125)	1.46 (0.0011)	0	-0.01 (0.0125)	0.37 (0.0003)	92.5%	-0.01 (0.0125)	1.46 (0.0011)
D8	19220.7 (2.5)	4.7 (0)	0.03	0	0 (0.025)	0.35 (0.0002)	97.4%	0 (0.0099)	1.36 (0.0009)	0	0 (0.0099)	0.35 (0.0002)	97.4%	0 (0.0099)	1.36 (0.0009)
D9	19522.3 (2.6)	5 (0)	0.2	2	0.4 (0.204)	0.36 (0.0003)	1.2%	-1.6 (0.0125)	1.42 (0.0011)	2	0.4 (0.0125)	0.36 (0.0003)	1.2%	-1.6 (0.0125)	1.42 (0.0011)
D10	19126.3 (2.6)	4.6 (0)	0.19	2	0.37 (0.187)	0.34 (0.0002)	0.1%	-1.63 (0.01)	1.32 (0.0009)	2	0.37 (0.01)	0.34 (0.0002)	0.1%	-1.63 (0.01)	1.32 (0.0009)
D11	19522.3 (2.6)	5 (0)	0.2	2	0.4 (0.204)	0.36 (0.0003)	1.2%	-1.6 (0.0125)	1.42 (0.0011)	2	0.4 (0.0125)	0.36 (0.0003)	1.2%	-1.6 (0.0125)	1.42 (0.0011)
D12	19126.3 (2.6)	4.6 (0)	0.19	2	0.37 (0.187)	0.34 (0.0002)	0.1%	-1.63 (0.01)	1.32 (0.0009)	2	0.37 (0.01)	0.34 (0.0002)	0.1%	-1.63 (0.01)	1.32 (0.0009)
D13	19522.3 (2.6)	5 (0)	0.93	2.9	1.3 (0.928)	0.36 (0.0003)	1.2%	-1.6 (0.0125)	1.42 (0.0011)	2.9	1.3 (0.0125)	0.36 (0.0003)	1.2%	-1.6 (0.0125)	1.42 (0.0011)
D14	19126.3 (2.6)	4.6 (0)	0.97	2.9	1.27 (0.971)	0.34 (0.0002)	0.1%	-1.63 (0.01)	1.32 (0.0009)	2.9	1.27 (0.01)	0.34 (0.0002)	0.1%	-1.63 (0.01)	1.32 (0.0009)
D15	19480.2 (2.7)	4.9 (0)	0.7	0.9	0.89 (0.701)	0.36 (0.0003)	91.9%	-0.01 (0.0124)	1.39 (0.001)	0.9	0.89 (0.0124)	0.36 (0.0003)	91.9%	-0.01 (0.0124)	1.39 (0.001)
D16	19076.6 (2.6)	4.6 (0)	0.79	0.9	0.9 (0.789)	0.33 (0.0002)	96.1%	0 (0.0099)	1.29 (0.0009)	0.9	0.9 (0.0099)	0.33 (0.0002)	96.1%	0 (0.0099)	1.29 (0.0009)
D17	19522.3 (2.6)	5 (0)	1	3.5	1.9 (0.997)	0.36 (0.0003)	1.2%	-1.6 (0.0125)	1.42 (0.0011)	3.5	1.9 (0.0125)	0.36 (0.0003)	1.2%	-1.6 (0.0125)	1.42 (0.0011)
D18	19126.3 (2.6)	4.6 (0)	1	3.5	1.87 (1)	0.34 (0.0002)	0.1%	-1.63 (0.01)	1.32 (0.0009)	3.5	1.87 (0.01)	0.34 (0.0002)	0.1%	-1.63 (0.01)	1.32 (0.0009)
D19	19480.3 (2.7)	4.9 (0)	0.97	1.5	1.49 (0.973)	0.36 (0.0003)	91.9%	-0.01 (0.0124)	1.39 (0.001)	1.5	1.49 (0.0124)	0.36 (0.0003)	91.9%	-0.01 (0.0124)	1.39 (0.001)
D20	19076.6 (2.6)	4.6 (0)	1	1.5	1.5 (0.999)	0.33 (0.0002)	96.1%	0 (0.0099)	1.29 (0.0009)	1.5	1.5 (0.0099)	0.33 (0.0002)	96.1%	0 (0.0099)	1.29 (0.0009)
D21	19522.8 (2.7)	5 (0)	1	-5	-3.41 (0.999)	0.36 (0.0003)	1.8%	1.59 (0.0124)	1.42 (0.0011)	-5	-3.41 (0.0124)	0.36 (0.0003)	1.8%	1.59 (0.0124)	1.42 (0.0011)
D22	19126.1 (2.6)	4.6 (0)	1	-5	-3.36 (0.999)	0.34 (0.0002)	0%	1.64 (0.01)	1.32 (0.0009)	-5	-3.36 (0.01)	0.34 (0.0002)	0%	1.64 (0.01)	1.32 (0.0009)
D23	19480.3 (2.7)	4.9 (0)	1	-3	-3.01 (1)	0.36 (0.0003)	91.9%	-0.01 (0.0124)	1.39 (0.001)	-3	-3.01 (0.0124)	0.36 (0.0003)	91.9%	-0.01 (0.0124)	1.39 (0.001)
D24	19076.6 (2.6)	4.6 (0)	1	-3	-3 (1)	0.33 (0.0002)	96.1%	0 (0.0099)	1.29 (0.0009)	-3	-3 (0.0099)	0.33 (0.0002)	96.1%	0 (0.0099)	1.29 (0.0009)
D25	19578.2 (2.7)	5 (0)	0.35	2	-0.59 (0.354)	0.37 (0.0003)	0%	-2.59 (0.0127)	1.46 (0.0012)	2	-0.59 (0.0127)	0.37 (0.0003)	0%	-2.59 (0.0127)	1.46 (0.0012)
D26	19192.2 (2.6)	4.7 (0)	0.45	2	-0.64 (0.452)	0.35 (0.0003)	0%	-2.64 (0.0102)	1.36 (0.001)	2	-0.64 (0.0102)	0.35 (0.0003)	0%	-2.64 (0.0102)	1.36 (0.001)
D27	19594.7 (2.6)	5.1 (0)	0.08	2.966	-0.02 (0.075)	0.38 (0.0003)	0%	-2.99 (0.0127)	1.48 (0.0012)	2.58	-0.02 (0.0127)	0.38 (0.0003)	0%	-2.6 (0.0127)	1.48 (0.0012)
D28	19211.8 (2.6)	4.7 (0)	0.03	2.966	-0.07 (0.034)	0.35 (0.0003)	0%	-3.04 (0.0102)	1.37 (0.001)	2.58	-0.07 (0.0102)	0.35 (0.0003)	0%	-2.65 (0.0102)	1.37 (0.001)
D29	19542 (2.7)	5 (0)	0.21	0.966	-0.4 (0.21)	0.37 (0.0003)	5.9%	-1.36 (0.0125)	1.43 (0.0011)	0.58	-0.4 (0.0125)	0.37 (0.0003)	24.5%	-0.98 (0.0125)	1.43 (0.0011)
D30	19150.1 (2.6)	4.6 (0)	0.21	0.966	-0.41 (0.213)	0.34 (0.0002)	0.9%	-1.38 (0.0099)	1.33 (0.0009)	0.58	-0.41 (0.0099)	0.34 (0.0002)	15.3%	-0.99 (0.0099)	1.33 (0.0009)
D31	19640.4 (2.6)	5.1 (0)	0.19	2	0.42 (0.194)	0.38 (0.0003)	1.9%	-1.58 (0.0126)	1.49 (0.0012)	2	0.42 (0.0126)	0.38 (0.0003)	1.9%	-1.58 (0.0126)	1.49 (0.0012)
D32	19266.1 (2.5)	4.7 (0)	0.16	2	0.38 (0.163)	0.35 (0.0003)	0.1%	-1.62 (0.0099)	1.39 (0.001)	2	0.38 (0.0099)	0.35 (0.0003)	0.1%	-1.62 (0.0099)	1.39 (0.001)
D33	19686.1 (2.6)	5.2 (0)	0.06	2.5	-0.09 (0.064)	0.39 (0.0003)	0%	-2.59 (0.0127)	1.53 (0.0013)	2	-0.09 (0.0127)	0.39 (0.0003)	0%	-2.09 (0.0127)	1.53 (0.0013)
D34	19320.2 (2.5)	4.8 (0)	0.04	2.5	-0.14 (0.04)	0.36 (0.0003)	0%	-2.64 (0.01)	1.42 (0.0011)	2	-0.14 (0.01)	0.36 (0.0003)	0%	-2.14 (0.01)	1.42 (0.0011)
D35	19617.6 (2.6)	5.1 (0)	0.28	0.5	-0.52 (0.276)	0.38 (0.0003)	24.3%	-1.02 (0.0125)	1.47 (0.0011)	0	-0.52 (0.0125)	0.38 (0.0003)	72.3%	-0.52 (0.0125)	1.47 (0.0011)
D36	19239.7 (2.6)	4.7 (0)	0.3	0.5	-0.52 (0.299)	0.35 (0.0002)	14.9%	-1.02 (0.0099)	1.37 (0.0009)	0	-0.52 (0.0099)	0.35 (0.0002)	70%	-0.52 (0.0099)	1.37 (0.0009)

S3 Table Q: The mean treatment effect estimates, their standard errors, and goodness-of-fit statistics, for each of the 36 simulated scenarios assuming the mean structure from Model 8 ($y_{itk} = \beta_0 + \kappa_t + \xi_d + v_{0ik} + \epsilon_{itk}$) with autoregressive (AR(1)) covariance structure between within subject measurement, together with the Monte Carlo standard error of each estimate in brackets. Each scenario was simulated 1000 times.

				Treatment Effect at 6 months						Time Averaged Treatment Effect					
Scenario	BIC	MSE	Proportion Significant Intervention Parameter	True Value	Estimate	Estimate SE	Coverage of 95% C.I.	Bias	Confidence Interval Width	True Value	Estimate	Estimate SE	Coverage of 95% C.I.	Bias	Confidence Interval Width
D1	19642.7 (2.7)	4.9 (0)	0.04	0	0 (0.044)	0.6 (0.0004)	93.3%	0 (0.0205)	2.33 (0.0014)	0	-0.02 (0.0175)	0.49 (0.0004)	91.3%	-0.02 (0.0175)	1.93 (0.0017)
D2	19236.5 (2.6)	4.6 (0)	0.04	0	0.01 (0.043)	0.55 (0.0003)	95.8%	0.01 (0.0175)	2.17 (0.0012)	0	0 (0.0135)	0.46 (0.0003)	96.6%	0 (0.0135)	1.79 (0.0013)
D3	19636.6 (2.7)	4.9 (0)	1	0	0 (1)	0.59 (0.0003)	93.6%	0 (0.0203)	2.31 (0.0012)	0	-0.02 (0.0171)	0.48 (0.0003)	91.6%	-0.02 (0.0171)	1.89 (0.0013)
D4	19231.2 (2.6)	4.6 (0)	1	0	0.02 (0.998)	0.55 (0.0003)	95.5%	0.02 (0.0174)	2.15 (0.0011)	0	0 (0.0131)	0.45 (0.0003)	96.5%	0 (0.0131)	1.76 (0.0011)
D5	19636.6 (2.7)	4.9 (0)	1	0	0 (1)	0.59 (0.0003)	93.6%	0 (0.0203)	2.31 (0.0012)	0	-0.02 (0.0171)	0.48 (0.0003)	91.6%	-0.02 (0.0171)	1.89 (0.0013)
D6	19231.2 (2.6)	4.6 (0)	1	0	0.02 (1)	0.55 (0.0003)	95.7%	0.02 (0.0174)	2.15 (0.0011)	0	0 (0.0131)	0.45 (0.0003)	96.7%	0 (0.0131)	1.76 (0.0011)
D7	19636.6 (2.7)	4.9 (0)	1	0	0 (1)	0.59 (0.0003)	93.6%	0 (0.0203)	2.31 (0.0012)	0	-0.02 (0.0171)	0.48 (0.0003)	91.6%	-0.02 (0.0171)	1.89 (0.0013)
D8	19231.2 (2.6)	4.6 (0)	1	0	0.02 (0.999)	0.55 (0.0003)	95.6%	0.02 (0.0174)	2.15 (0.0011)	0	0 (0.0131)	0.45 (0.0003)	96.6%	0 (0.0131)	1.76 (0.0011)
D9	19636.6 (2.7)	4.9 (0)	1	2	2 (1)	0.59 (0.0003)	93.6%	0 (0.0203)	2.31 (0.0012)	2	1.98 (0.0171)	0.48 (0.0003)	91.6%	-0.02 (0.0171)	1.89 (0.0013)
D10	19231.2 (2.6)	4.6 (0)	1	2	2.02 (1)	0.55 (0.0003)	95.7%	0.02 (0.0174)	2.15 (0.0011)	2	2 (0.0131)	0.45 (0.0003)	96.7%	0 (0.0131)	1.76 (0.0011)
D11	19636.6 (2.7)	4.9 (0)	1	2	2 (1)	0.59 (0.0003)	93.6%	0 (0.0203)	2.31 (0.0012)	2	1.98 (0.0171)	0.48 (0.0003)	91.6%	-0.02 (0.0171)	1.89 (0.0013)
D12	19231.3 (2.6)	4.6 (0)	1	2	2.02 (0.999)	0.55 (0.0003)	95.6%	0.02 (0.0174)	2.15 (0.0011)	2	2 (0.0132)	0.45 (0.0003)	96.6%	0 (0.0132)	1.76 (0.0011)
D13	19636.6 (2.7)	4.9 (0)	1	2.9	2.9 (1)	0.59 (0.0003)	93.6%	0 (0.0203)	2.31 (0.0012)	2.9	2.96 (0.0171)	0.48 (0.0003)	91.5%	0.06 (0.0171)	1.89 (0.0013)
D14	19231.2 (2.6)	4.6 (0)	1	2.9	2.92 (1)	0.55 (0.0003)	95.7%	0.02 (0.0174)	2.15 (0.0011)	2.9	2.97 (0.0131)	0.45 (0.0003)	96.9%	0.07 (0.0131)	1.76 (0.0011)
D15	19636.6 (2.7)	4.9 (0)	1	0.9	0.9 (1)	0.59 (0.0003)	93.6%	0 (0.0203)	2.31 (0.0012)	0.9	0.96 (0.0171)	0.48 (0.0003)	91.5%	0.06 (0.0171)	1.89 (0.0013)
D16	19231.3 (2.6)	4.6 (0)	1	0.9	0.92 (0.999)	0.55 (0.0003)	95.6%	0.02 (0.0174)	2.15 (0.0011)	0.9	0.97 (0.0132)	0.45 (0.0003)	96.8%	0.07 (0.0132)	1.76 (0.0011)
D17	19636.8 (2.7)	4.9 (0)	1	3.5	3.5 (0.999)	0.59 (0.0003)	93.5%	0 (0.0203)	2.31 (0.0012)	3.5	3.61 (0.0171)	0.48 (0.0003)	91.2%	0.11 (0.0171)	1.89 (0.0013)
D18	19231.2 (2.6)	4.6 (0)	1	3.5	3.52 (1)	0.55 (0.0003)	95.7%	0.02 (0.0174)	2.15 (0.0011)	3.5	3.62 (0.0131)	0.45 (0.0003)	96.3%	0.12 (0.0131)	1.76 (0.0011)
D19	19636.6 (2.7)	4.9 (0)	1	1.5	1.5 (1)	0.59 (0.0003)	93.6%	0 (0.0203)	2.31 (0.0012)	1.5	1.61 (0.0171)	0.48 (0.0003)	91.3%	0.11 (0.0171)	1.89 (0.0013)
D20	19231.2 (2.6)	4.6 (0)	1	1.5	1.52 (0.999)	0.55 (0.0003)	95.6%	0.02 (0.0174)	2.15 (0.0011)	1.5	1.62 (0.0132)	0.45 (0.0003)	96.2%	0.12 (0.0132)	1.76 (0.0011)
D21	19636.6 (2.7)	4.9 (0)	1	-5	-5 (1)	0.59 (0.0003)	93.6%	0 (0.0203)	2.31 (0.0012)	-5	-5.27 (0.0171)	0.48 (0.0003)	87.9%	-0.27 (0.0171)	1.89 (0.0013)
D22	19231.2 (2.6)	4.6 (0)	1	-5	-4.98 (1)	0.55 (0.0003)	95.7%	0.02 (0.0174)	2.15 (0.0011)	-5	-5.25 (0.0131)	0.45 (0.0003)	92.8%	-0.25 (0.0131)	1.76 (0.0011)
D23	19636.6 (2.7)	4.9 (0)	0.44	-3	-3 (0.439)	0.59 (0.0003)	93.5%	0 (0.0203)	2.31 (0.0012)	-3	-3.27 (0.0171)	0.48 (0.0003)	87.8%	-0.27 (0.0171)	1.89 (0.0013)
D24	19231.2 (2.6)	4.6 (0)	0.53	-3	-2.98 (0.531)	0.55 (0.0003)	95.7%	0.02 (0.0174)	2.15 (0.0011)	-3	-3.25 (0.0131)	0.45 (0.0003)	92.8%	-0.25 (0.0131)	1.76 (0.0011)
D25	19636.6 (2.7)	4.9 (0)	1	2	2 (1)	0.59 (0.0003)	93.6%	0 (0.0203)	2.31 (0.0012)	2	1.98 (0.0171)	0.48 (0.0003)	91.6%	-0.02 (0.0171)	1.89 (0.0013)
D26	19231.2 (2.6)	4.6 (0)	1	2	2.02 (1)	0.55 (0.0003)	95.7%	0.02 (0.0174)	2.15 (0.0011)	2	2 (0.0131)	0.45 (0.0003)	96.7%	0 (0.0131)	1.76 (0.0011)
D27	19636.6 (2.7)	4.9 (0)	1	2.966	2.97 (1)	0.59 (0.0003)	93.6%	0 (0.0203)	2.31 (0.0012)	2.58	2.61 (0.0171)	0.48 (0.0003)	91.7%	0.03 (0.0171)	1.89 (0.0013)
D28	19231.2 (2.6)	4.6 (0)	1	2.966	2.98 (1)	0.55 (0.0003)	95.7%	0.02 (0.0174)	2.15 (0.0011)	2.58	2.63 (0.0131)	0.45 (0.0003)	96.8%	0.05 (0.0131)	1.76 (0.0011)
D29	19636.6 (2.7)	4.9 (0)	1	0.966	0.97 (1)	0.59 (0.0003)	93.6%	0 (0.0203)	2.31 (0.0012)	0.58	0.61 (0.0171)	0.48 (0.0003)	91.7%	0.03 (0.0171)	1.89 (0.0013)
D30	19231.2 (2.6)	4.6 (0)	1	0.966	0.98 (1)	0.55 (0.0003)	95.7%	0.02 (0.0174)	2.15 (0.0011)	0.58	0.63 (0.0131)	0.45 (0.0003)	96.8%	0.05 (0.0131)	1.76 (0.0011)
D31	19636.6 (2.7)	4.9 (0)	1	2	2 (1)	0.59 (0.0003)	93.6%	0 (0.0203)	2.31 (0.0012)	2	1.98 (0.0171)	0.48 (0.0003)	91.6%	-0.02 (0.0171)	1.89 (0.0013)
D32	19231.2 (2.6)	4.6 (0)	1	2	2.02 (0.999)	0.55 (0.0003)	95.6%	0.02 (0.0174)	2.15 (0.0011)	2	2 (0.0132)	0.45 (0.0003)	96.6%	0 (0.0132)	1.76 (0.0011)
D33	19636.6 (2.7)	4.9 (0)	1	2.5	2.5 (1)	0.59 (0.0003)	93.6%	0 (0.0203)	2.31 (0.0012)	2	1.98 (0.0171)	0.48 (0.0003)	91.6%	-0.02 (0.0171)	1.89 (0.0013)
D34	19231.2 (2.6)	4.6 (0)	1	2.5	2.52 (1)	0.55 (0.0003)	95.7%	0.02 (0.0174)	2.15 (0.0011)	2	2 (0.0131)	0.45 (0.0003)	96.7%	0 (0.0131)	1.76 (0.0011)
D35	19636.6 (2.7)	4.9 (0)	1	0.5	0.5 (0.998)	0.59 (0.0003)	93.4%	0 (0.0203)	2.31 (0.0012)	0	-0.02 (0.0171)	0.48 (0.0003)	91.5%	-0.02 (0.0171)	1.89 (0.0013)
D36	19231.2 (2.6)	4.6 (0)	1	0.5	0.52 (1)	0.55 (0.0003)	95.7%	0.02 (0.0174)	2.15 (0.0011)	0	0 (0.0131)	0.45 (0.0003)	96.7%	0 (0.0131)	1.76 (0.0011)

S3 Table R: The mean treatment effect estimates, their standard errors, and goodness-of-fit statistics, for each of the 36 simulated scenarios assuming the mean structure from Model 9 ($y_{itk} = \beta_0 + \partial x_{tk} + \tau t + \psi d_{tk} + \zeta t^2 + v_{0ik} + \epsilon_{itk}$) with autoregressive (AR(1)) covariance structure between within subject measurement, together with the Monte Carlo standard error of each estimate in brackets. Each scenario was simulated 1000 times.

				Treatment Effect at 6 months						Time Averaged Treatment Effect					
Scenario	BIC	MSE	Proportion Significant Intervention Parameter	True Value	Estimate	Estimate SE	Coverage of 95% C.I.	Bias	Confidence Interval Width	True Value	Estimate	Estimate SE	Coverage of 95% C.I.	Bias	Confidence Interval Width
D1	19500.4 (2.7)	4.9 (0)	0.12	0	-0.01 (0.124)	0.46 (0.0004)	91.2%	-0.01 (0.0163)	1.78 (0.0016)	0	-0.01 (0.0163)	0.46 (0.0004)	91.2%	-0.01 (0.0163)	1.78 (0.0016)
D2	19096.2 (2.6)	4.6 (0)	0.1	0	0 (0.099)	0.42 (0.0003)	96%	0 (0.0126)	1.66 (0.0012)	0	0 (0.0126)	0.42 (0.0003)	96%	0 (0.0126)	1.66 (0.0012)
D3	19494.4 (2.7)	4.9 (0)	0.12	0	-0.01 (0.119)	0.45 (0.0003)	91.3%	-0.01 (0.016)	1.75 (0.0013)	0	-0.01 (0.016)	0.45 (0.0003)	91.3%	-0.01 (0.016)	1.75 (0.0013)
D4	19090.9 (2.6)	4.6 (0)	0.09	0	0 (0.089)	0.42 (0.0003)	96.5%	0 (0.0123)	1.63 (0.0011)	0	0 (0.0123)	0.42 (0.0003)	96.5%	0 (0.0123)	1.63 (0.0011)
D5	19494.6 (2.7)	4.9 (0)	0.12	0	-0.01 (0.119)	0.45 (0.0003)	91.4%	-0.01 (0.016)	1.75 (0.0013)	0	-0.01 (0.016)	0.45 (0.0003)	91.4%	-0.01 (0.016)	1.75 (0.0013)
D6	19091.1 (2.6)	4.6 (0)	0.09	0	0 (0.088)	0.42 (0.0003)	96.5%	0 (0.0123)	1.63 (0.0011)	0	0 (0.0123)	0.42 (0.0003)	96.5%	0 (0.0123)	1.63 (0.0011)
D7	19615.9 (2.6)	5.1 (0)	0.1	0	-0.02 (0.104)	0.46 (0.0003)	92.8%	-0.02 (0.016)	1.82 (0.0014)	0	-0.02 (0.016)	0.46 (0.0003)	92.8%	-0.02 (0.016)	1.82 (0.0014)
D8	19235.1 (2.5)	4.7 (0)	0.07	0	0 (0.073)	0.44 (0.0003)	97.6%	0 (0.0122)	1.71 (0.0011)	0	0 (0.0122)	0.44 (0.0003)	97.6%	0 (0.0122)	1.71 (0.0011)
D9	19494.1 (2.7)	4.9 (0)	1	2	1.99 (0.999)	0.45 (0.0003)	91.3%	-0.01 (0.016)	1.75 (0.0013)	2	1.99 (0.016)	0.45 (0.0003)	91.3%	-0.01 (0.016)	1.75 (0.0013)
D10	19090.9 (2.6)	4.6 (0)	1	2	2 (1)	0.42 (0.0003)	96.5%	0 (0.0123)	1.63 (0.0011)	2	2 (0.0123)	0.42 (0.0003)	96.5%	0 (0.0123)	1.63 (0.0011)
D11	19494.3 (2.7)	4.9 (0)	1	2	1.99 (1)	0.45 (0.0003)	91.4%	-0.01 (0.016)	1.75 (0.0013)	2	1.99 (0.016)	0.45 (0.0003)	91.4%	-0.01 (0.016)	1.75 (0.0013)
D12	19090.9 (2.6)	4.6 (0)	1	2	2 (1)	0.42 (0.0003)	96.5%	0 (0.0123)	1.63 (0.0011)	2	2 (0.0123)	0.42 (0.0003)	96.5%	0 (0.0123)	1.63 (0.0011)
D13	19494.3 (2.7)	4.9 (0)	1	2.9	2.89 (1)	0.45 (0.0003)	91.4%	-0.01 (0.016)	1.75 (0.0013)	2.9	2.89 (0.016)	0.45 (0.0003)	91.4%	-0.01 (0.016)	1.75 (0.0013)
D14	19090.9 (2.6)	4.6 (0)	1	2.9	2.9 (1)	0.42 (0.0003)	96.5%	0 (0.0123)	1.63 (0.0011)	2.9	2.9 (0.0123)	0.42 (0.0003)	96.5%	0 (0.0123)	1.63 (0.0011)
D15	19494.3 (2.7)	4.9 (0)	0.64	0.9	0.89 (0.644)	0.45 (0.0003)	91.3%	-0.01 (0.016)	1.75 (0.0013)	0.9	0.89 (0.016)	0.45 (0.0003)	91.3%	-0.01 (0.016)	1.75 (0.0013)
D16	19090.8 (2.6)	4.6 (0)	0.73	0.9	0.9 (0.734)	0.42 (0.0003)	96.3%	0 (0.0123)	1.63 (0.0011)	0.9	0.9 (0.0123)	0.42 (0.0003)	96.3%	0 (0.0123)	1.63 (0.0011)
D17	19494.3 (2.7)	4.9 (0)	1	3.5	3.49 (1)	0.45 (0.0003)	91.4%	-0.01 (0.016)	1.75 (0.0013)	3.5	3.49 (0.016)	0.45 (0.0003)	91.4%	-0.01 (0.016)	1.75 (0.0013)
D18	19090.9 (2.6)	4.6 (0)	1	3.5	3.5 (1)	0.42 (0.0003)	96.5%	0 (0.0123)	1.63 (0.0011)	3.5	3.5 (0.0123)	0.42 (0.0003)	96.5%	0 (0.0123)	1.63 (0.0011)
D19	19494.3 (2.7)	4.9 (0)	0.96	1.5	1.49 (0.956)	0.45 (0.0003)	91.3%	-0.01 (0.016)	1.75 (0.0013)	1.5	1.49 (0.016)	0.45 (0.0003)	91.3%	-0.01 (0.016)	1.75 (0.0013)
D20	19090.9 (2.6)	4.6 (0)	0.99	1.5	1.5 (0.993)	0.42 (0.0003)	96.5%	0 (0.0123)	1.63 (0.0011)	1.5	1.5 (0.0123)	0.42 (0.0003)	96.5%	0 (0.0123)	1.63 (0.0011)
D21	19494.3 (2.7)	4.9 (0)	1	-5	-5.01 (1)	0.45 (0.0003)	91.4%	-0.01 (0.016)	1.75 (0.0013)	-5	-5.01 (0.016)	0.45 (0.0003)	91.4%	-0.01 (0.016)	1.75 (0.0013)
D22	19090.9 (2.6)	4.6 (0)	1	-5	-5 (1)	0.42 (0.0003)	96.5%	0 (0.0123)	1.63 (0.0011)	-5	-5 (0.0123)	0.42 (0.0003)	96.5%	0 (0.0123)	1.63 (0.0011)
D23	19494.3 (2.7)	4.9 (0)	1	-3	-3.01 (1)	0.45 (0.0003)	91.4%	-0.01 (0.016)	1.75 (0.0013)	-3	-3.01 (0.016)	0.45 (0.0003)	91.4%	-0.01 (0.016)	1.75 (0.0013)
D24	19090.8 (2.6)	4.6 (0)	1	-3	-3 (0.998)	0.42 (0.0003)	96.3%	0 (0.0123)	1.63 (0.0011)	-3	-3 (0.0123)	0.42 (0.0003)	96.3%	0 (0.0123)	1.63 (0.0011)
D25	19494.6 (2.7)	4.9 (0)	1	2	1.99 (1)	0.45 (0.0003)	91.4%	-0.01 (0.016)	1.75 (0.0013)	2	1.99 (0.016)	0.45 (0.0003)	91.4%	-0.01 (0.016)	1.75 (0.0013)
D26	19091.2 (2.6)	4.6 (0)	1	2	2 (1)	0.42 (0.0003)	96.6%	0 (0.0123)	1.63 (0.0011)	2	2 (0.0123)	0.42 (0.0003)	96.6%	0 (0.0123)	1.63 (0.0011)
D27	19498.2 (2.7)	4.9 (0)	1	2.966	2.75 (1)	0.45 (0.0003)	88.6%	-0.21 (0.016)	1.75 (0.0013)	2.58	2.75 (0.016)	0.45 (0.0003)	90.2%	0.17 (0.016)	1.75 (0.0013)
D28	19095.4 (2.6)	4.6 (0)	1	2.966	2.78 (1)	0.42 (0.0003)	93.1%	-0.19 (0.0123)	1.63 (0.0011)	2.58	2.78 (0.0123)	0.42 (0.0003)	93.6%	0.2 (0.0123)	1.63 (0.0011)
D29	19498.2 (2.7)	4.9 (0)	0.4	0.966	0.75 (0.403)	0.45 (0.0003)	88.6%	-0.21 (0.016)	1.75 (0.0013)	0.58	0.75 (0.016)	0.45 (0.0003)	90.2%	0.17 (0.016)	1.75 (0.0013)
D30	19095.4 (2.6)	4.6 (0)	0.46	0.966	0.78 (0.457)	0.42 (0.0003)	93.1%	-0.19 (0.0123)	1.63 (0.0011)	0.58	0.78 (0.0123)	0.42 (0.0003)	93.6%	0.2 (0.0123)	1.63 (0.0011)
D31	19615.9 (2.6)	5.1 (0)	1	2	1.98 (1)	0.46 (0.0003)	92.8%	-0.02 (0.016)	1.82 (0.0014)	2	1.98 (0.016)	0.46 (0.0003)	92.8%	-0.02 (0.016)	1.82 (0.0014)
D32	19235.1 (2.5)	4.7 (0)	1	2	2 (1)	0.44 (0.0003)	97.6%	0 (0.0122)	1.71 (0.0011)	2	2 (0.0122)	0.44 (0.0003)	97.6%	0 (0.0122)	1.71 (0.0011)
D33	19625.1 (2.6)	5.1 (0)	1	2.5	2.19 (1)	0.47 (0.0004)	87.4%	-0.31 (0.0161)	1.82 (0.0014)	2	2.19 (0.0161)	0.47 (0.0004)	90.9%	0.19 (0.0161)	1.82 (0.0014)
D34	19246 (2.6)	4.7 (0)	1	2.5	2.23 (0.999)	0.44 (0.0003)	92.5%	-0.27 (0.0122)	1.71 (0.0011)	2	2.23 (0.0122)	0.44 (0.0003)	94.3%	0.23 (0.0122)	1.71 (0.0011)
D35	19625.1 (2.6)	5.1 (0)	0.82	0.5	0.19 (0.815)	0.47 (0.0004)	87.4%	-0.31 (0.0161)	1.82 (0.0014)	0	0.19 (0.0161)	0.47 (0.0004)	90.9%	0.19 (0.0161)	1.82 (0.0014)
D36	19245.9 (2.6)	4.7 (0)	0.84	0.5	0.22 (0.839)	0.44 (0.0003)	92.6%	-0.28 (0.0122)	1.71 (0.0011)	0	0.22 (0.0122)	0.44 (0.0003)	94.4%	0.22 (0.0122)	1.71 (0.0011)
