# Supplementary material for: Mixed effects approach to the analysis of the stepped wedge cluster randomised trial—Investigating the confounding effect of time through simulation
Source: PLoS One. 2018 Dec 13;13(12):e0208876. doi: 10.1371/journal.pone.0208876 (PMC6292598; doi:10.1371/journal.pone.0208876)
Supplement: S4 Appendix — (PDF) [file pone.0208876.s004.pdf]

S4 Appendix. Additional plots

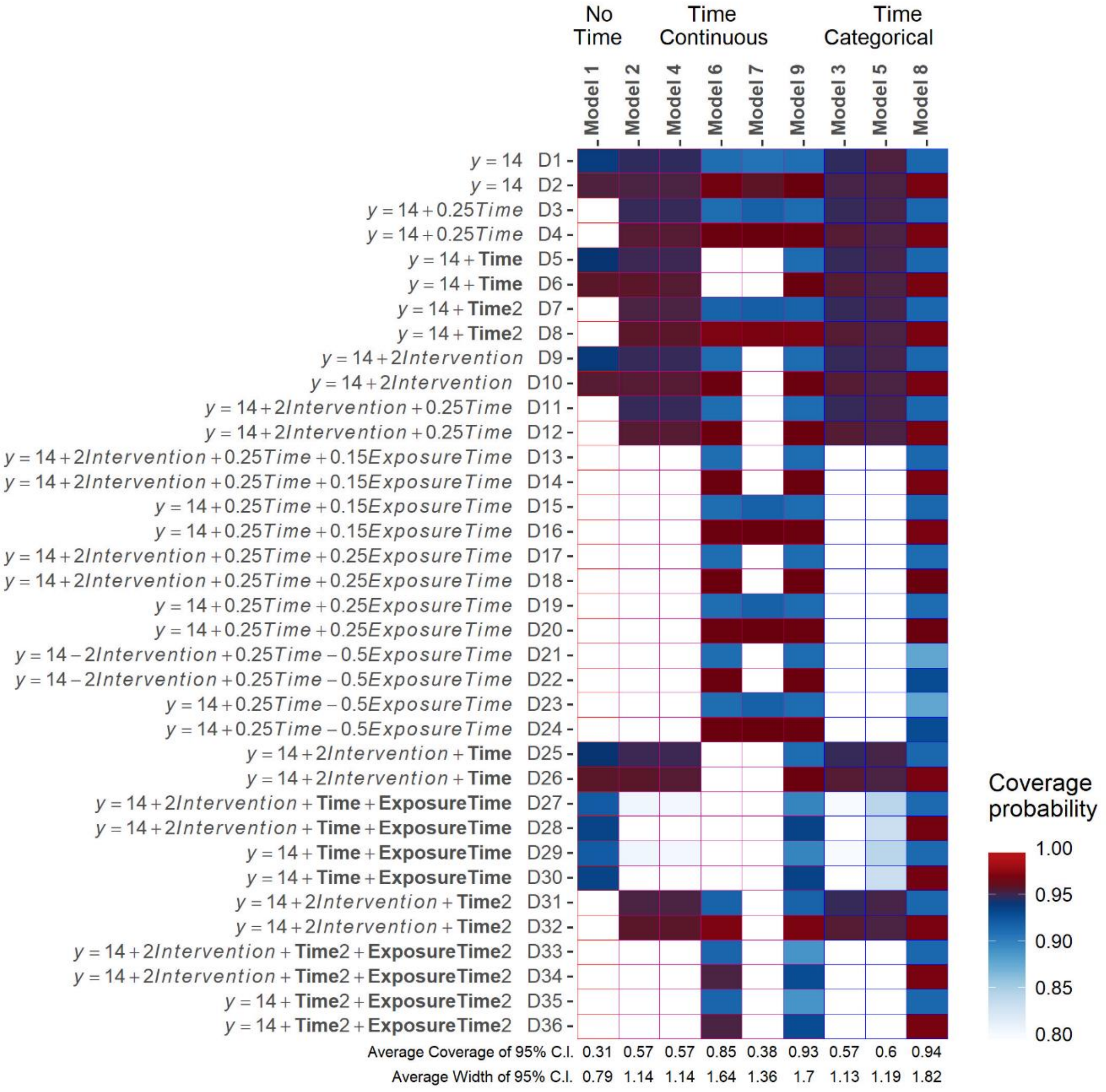

S4 Fig A. The heat map shows the coverage probability of the time-averaged intervention effect for the nine fitted models with CS correlation structure. Coverage probabilities between 0 and 0.95 are shown as blue, with dark blue assigned to coverage probabilities close to 0.95. Coverage probabilities over 0.95 are shown as red, with dark red indicative of coverage probabilities close to 0.95 and lighter red indicating coverage probabilities close to 1. Values at the bottom of each column show the average coverage probability for each fitted model and the average width of the confidence interval for the intervention effect. Odd-numbered scenarios are simulated with ρ = 0.-5 and even-numbered scenarios have ρ = 0.5.

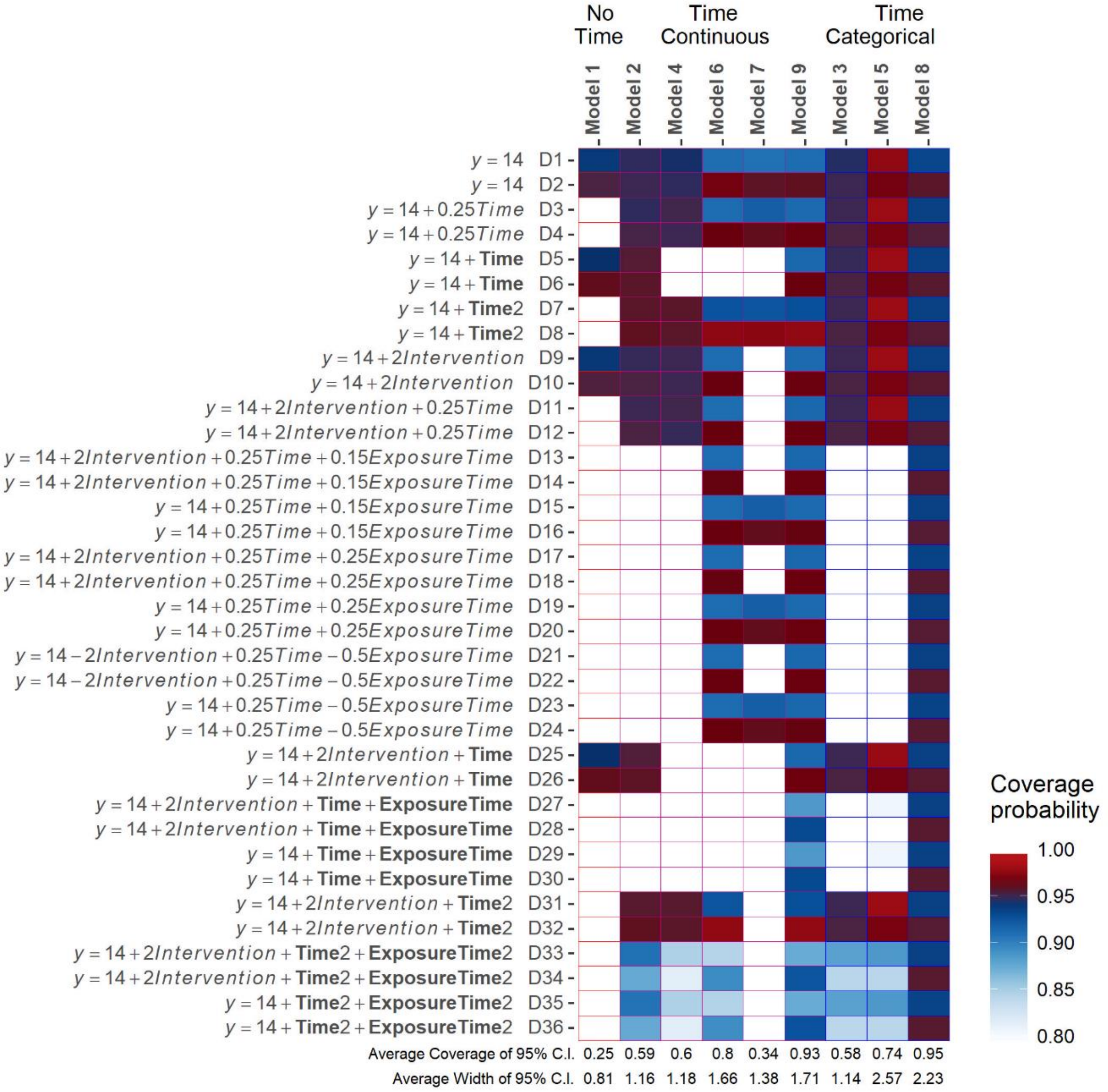

S4 Fig B. The heat map shows the coverage probability of the intervention effect at six months exposure for the nine fitted models with AR(1) correlation structure. Coverage probabilities between 0 and 0.95 are shown as blue, with dark blue assigned to coverage probabilities close to 0.95. Coverage probabilities over 0.95 are shown as red, with dark red indicative of coverage probabilities close to 0.95 and lighter red indicating coverage probabilities close to 1. Values at the bottom of each column show the average coverage probability for each fitted model and the average width of the confidence interval for the intervention effect. Odd-numbered scenarios are simulated with ρ = 0.-5 and even-numbered scenarios have ρ = 0.5.

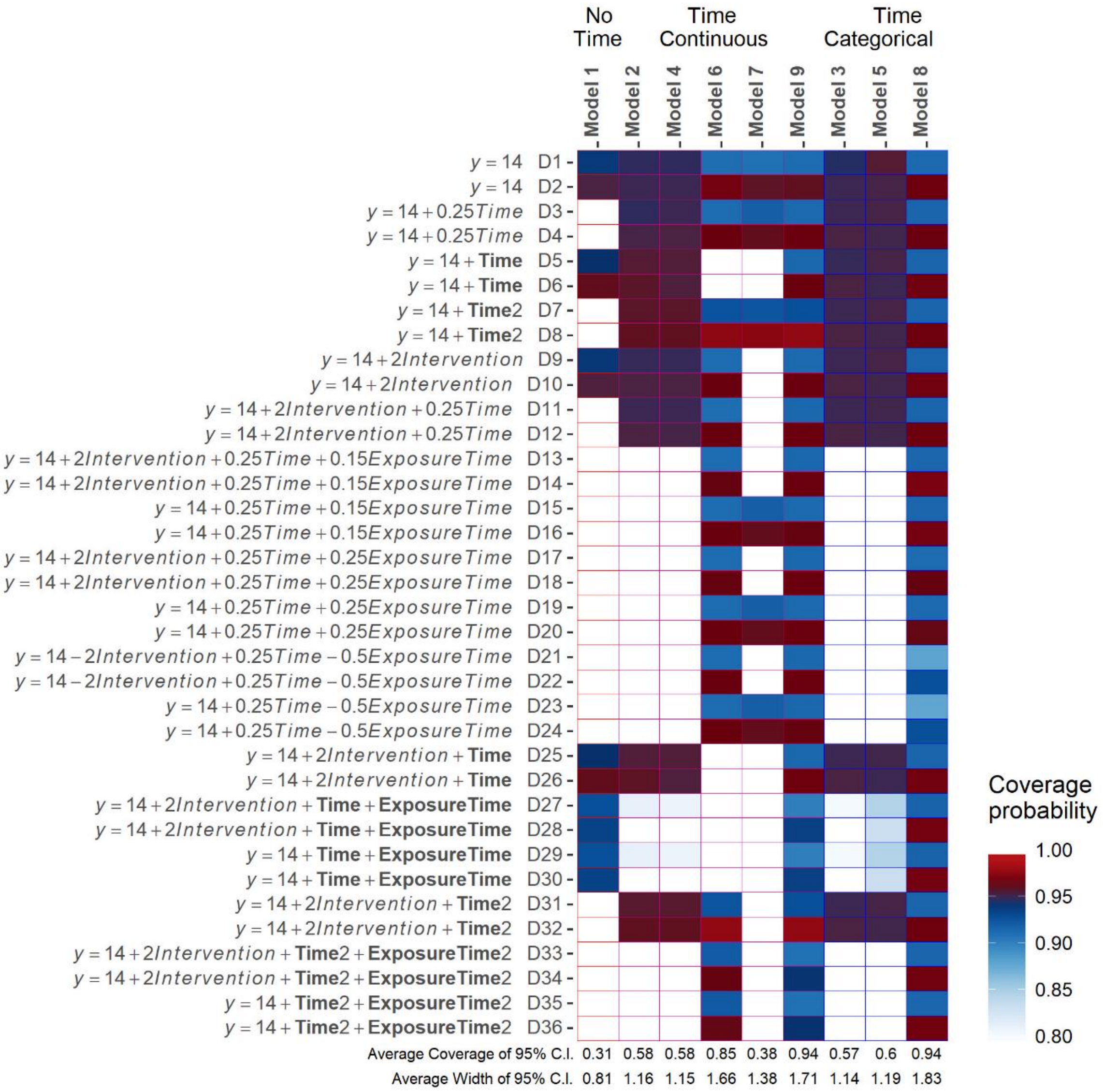

S4 Fig C. The heat map shows the coverage probability of the time-averaged intervention effect for the nine fitted models with AR(1) correlation structure. Coverage probabilities between 0 and 0.95 are shown as blue, with dark blue assigned to coverage probabilities close to 0.95. Coverage probabilities over 0.95 are shown as red, with dark red indicative of coverage probabilities close to 0.95 and lighter red indicating coverage probabilities close to 1. Values at the bottom of each column show the average coverage probability for each fitted model and the average width of the confidence interval for the intervention effect. Odd-numbered scenarios are simulated with ρ = 0.-5 and even-numbered scenarios have ρ = 0.5.

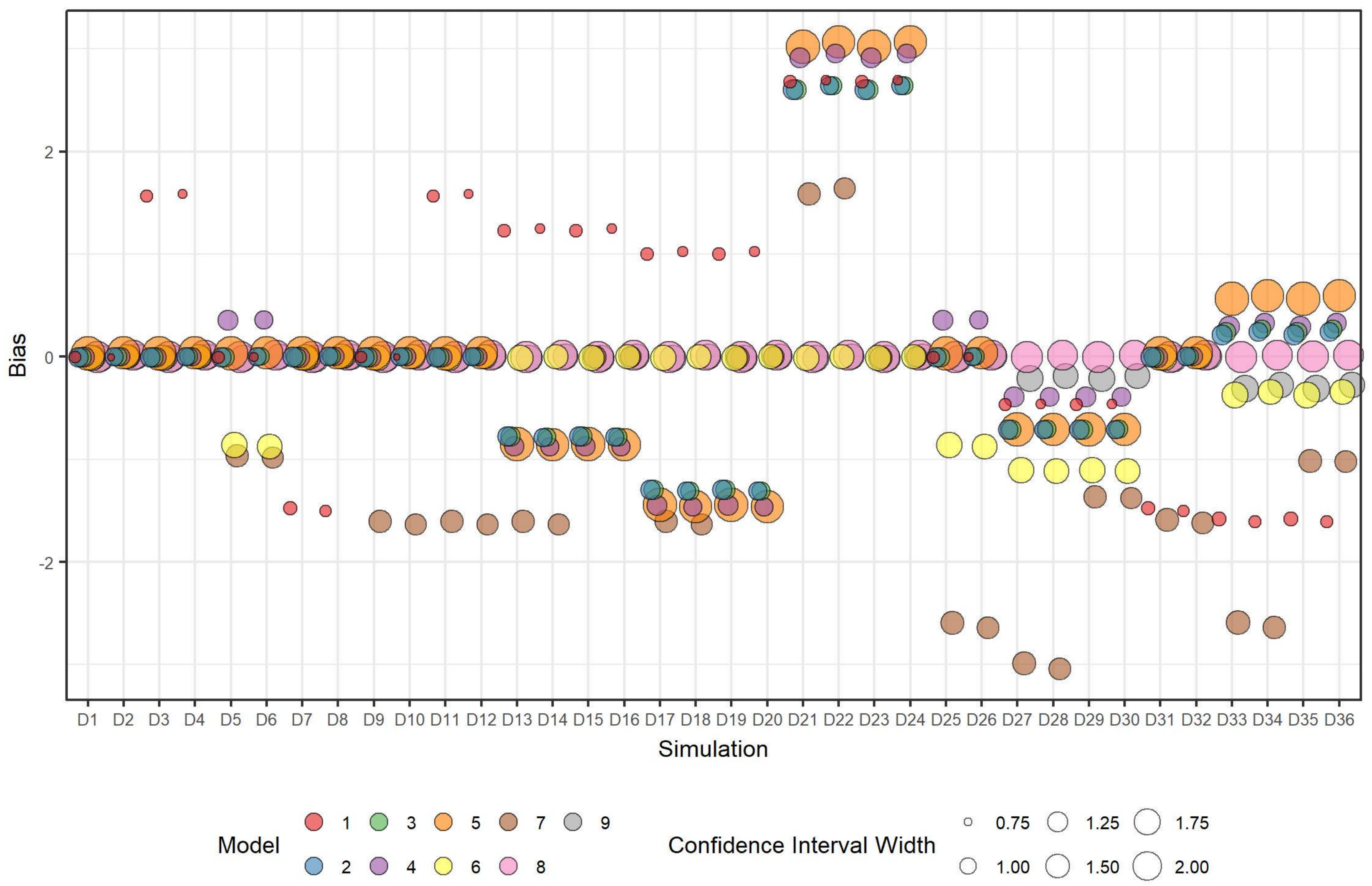

S4 Fig D. Plot of mean bias and mean 95% confidence interval width over all datasets within each scenario for each fitted model for the estimate of the intervention effect at six months exposure and where fitted models have assumed an AR(1) correlation structure.

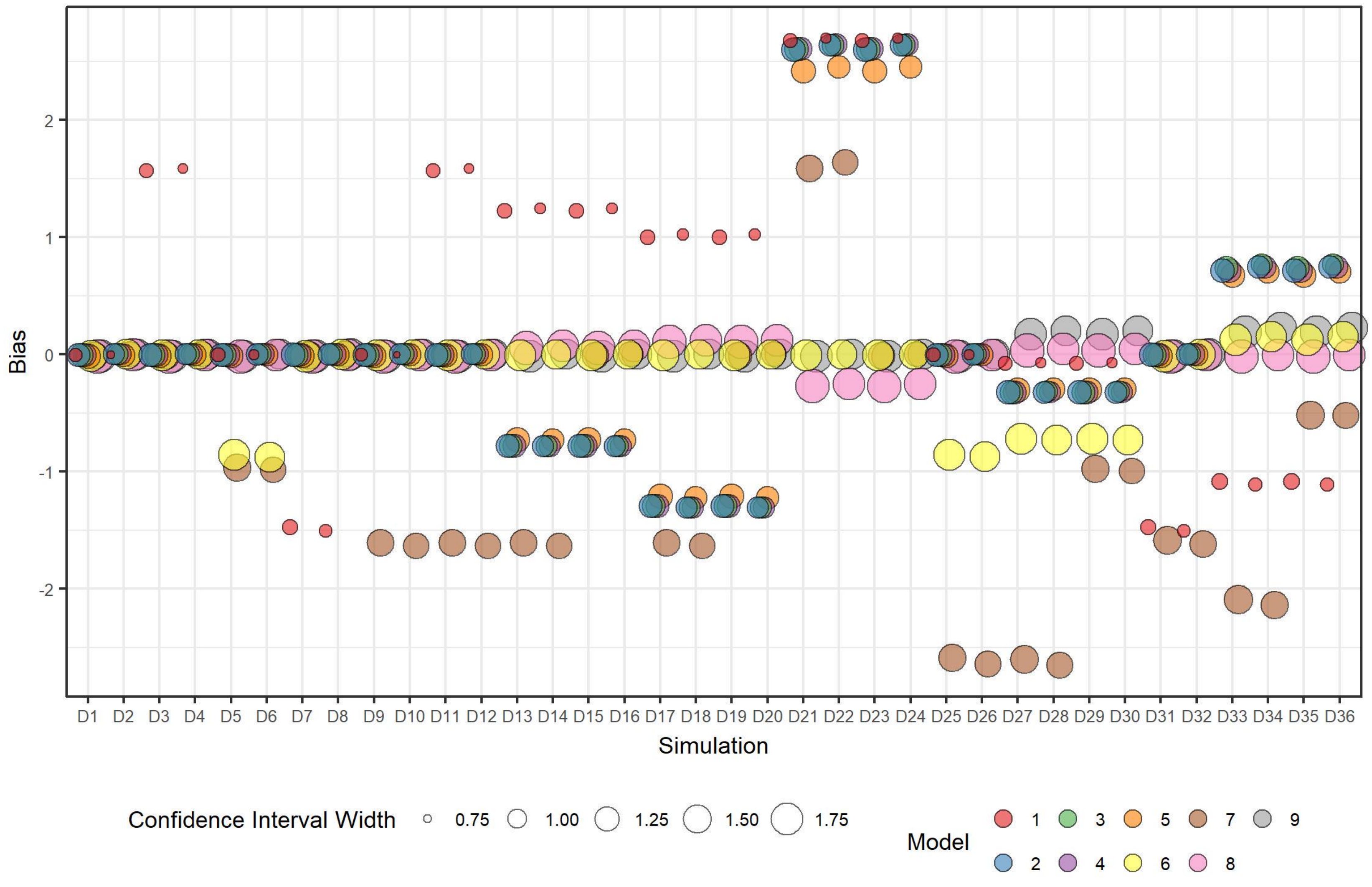

S4 Fig E. Plot of mean bias and mean 95% confidence interval width over all datasets within each scenario for each fitted model for the estimate of the time-averaged intervention effect and where fitted models have assumed an AR(1) correlation structure.

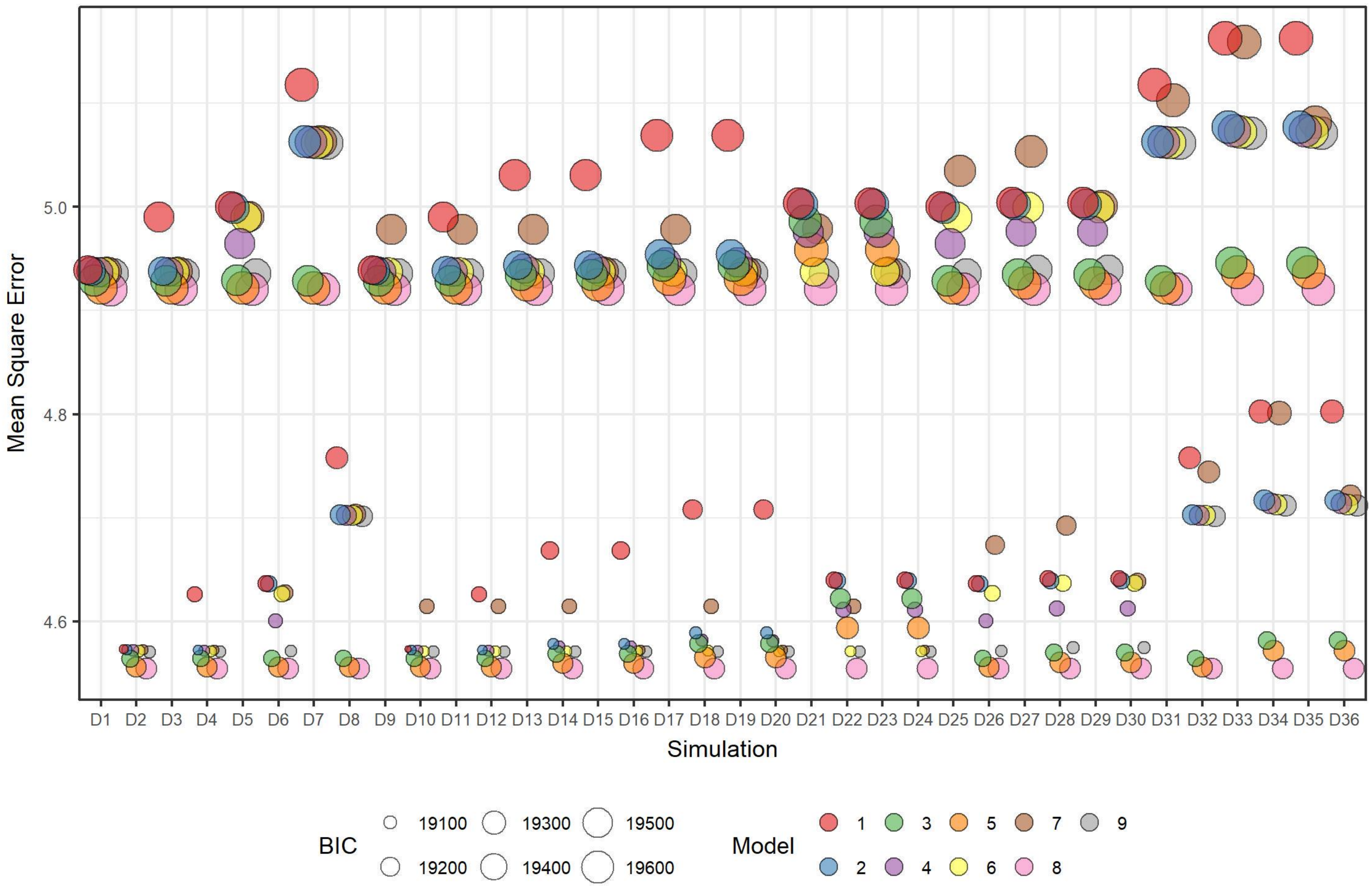

S4 Fig F. Plot of the mean of the MSE (mean square error) and mean BIC over all datasets within each scenario for each fitted model and where fitted models have assumed an AR(1) correlation structure.
